# Adenosine metabolic clearance maintains liver homeostasis by licensing arginine methylation of RIPK1

**DOI:** 10.1084/jem.20250603

**Published:** 2025-10-13

**Authors:** Ran Liu, Gengqiao Wang, Zhengting Jiang, Tianhao Zou, Chuanzheng Wang, Weimin Wang, Mao Cai, Shuhua Zhang, Guoliang Wang, Huan Cao, Di Zhang, Xueling Wang, Shenghe Deng, Tongxi Li, Jinyang Gu

**Affiliations:** 1 Center for Liver Transplantation, Union Hospital, Tongji Medical College, Huazhong University of Science and Technology, Wuhan, China; 2 Department of Hepatobiliary Surgery, The First Affiliated Hospital of Chongqing Medical University, Chongqing, China

## Abstract

Tumor necrosis factor α (TNFα) maintains homeostasis through promoting cell survival or cell death; however, how this process is regulated by metabolic pathways remains largely unknown. Here, we identify adenosine kinase (ADK), the key enzyme for catalyzing the conversion of adenosine to AMP, as an endogenous suppressor of RIPK1 kinase. ADK-mediated adenosine metabolic clearance is a prerequisite for transmethylation reactions on various cellular targets. We found that ADK licenses constitutive R606 symmetric dimethylation in RIPK1 death domain (DD), which is catalyzed by protein arginine methyltransferase 5. Upon TNFα stimulation, DD-mediated RIPK1 dimerization is inhibited by R606 methylation, preventing RIPK1 kinase activation and keeping cell death in check. Both hepatocyte-specific ADK knockout and systemic ADK inhibition cause spontaneous RIPK1-driven hepatocyte death, which leads to hepatic homeostasis disruption. Furthermore, ADK is reduced in hepatic ischemia–reperfusion, aggravating hepatic injury during liver surgery. Thus, this study reveals a mechanism of adenosine metabolism–dependent homeostasis maintenance that is implicated in both physiological and pathological conditions.

## Teaser

ADK removes adenosine to license PRMT5-mediated RIPK1 R606 symmetric dimethylation. The methylation inhibits death domain–mediated RIPK1 dimerization, which suppresses TNFα-induced cell death. ADK deficiency leads to RIPK1-driven hepatic tissue homeostasis disruption, including spontaneous liver injury in unstressed conditions and ischemia–reperfusion injury during liver surgery.

## Introduction

TNFα is an essential cytokine that maintains tissue homeostasis through different actions (Kalliolias and Ivashkiv, 2016; Brenner et al., 2015). Upon TNFR1 binding, TNFα promotes the expression of proinflammatory and prosurvival genes by activating MAPK and NF-κB signaling pathways (Brenner et al., 2015). Alternatively, TNFα triggers programmed cell death, including apoptosis and/or necroptosis (van Loo and Bertrand, 2023). Although TNFα facilitates immunologic defense against invading pathogens and immunologic surveillance against mutated host cells, deregulation of the TNFα pathway also leads to various inflammatory diseases such as steatohepatitis, ischemia–reperfusion injury (IRI), arthritis, and neurodegeneration (Wandrer et al., 2020; Scher et al., 2020; Venters et al., 2000; Wang et al., 2020). Because TNFα potentially results in two opposite outcomes, the pathway must be precisely regulated by multiple coordinators, among which receptor-interacting serine/threonine kinase 1 (RIPK1) is the critical node (Xu et al., 2021). Upon TNFα stimulation, RIPK1 and numerous other signaling proteins are recruited to TNFR1 and form the TNFR1 signaling complex called complex I (Micheau and Tschopp, 2003). In complex I, the kinase function of RIPK1 is normally inhibited by multiple posttranslational modifications, such as phosphorylation and ubiquitination, which constitute multilevel cell death–restraining checkpoints (Geng et al., 2017; Kist et al., 2021; Zhang et al., 2023a). However, when specific checkpoints are disabled under certain conditions, the death domain (DD) of RIPK1 promotes its dimerization; then in a dimer, trans-autophosphorylation leads to RIPK1 kinase activation that facilitates its interaction with FADD or RIPK3 and the subsequent assembly of apoptosis- or necroptosis-inducing complexes (Yuan et al., 2019), respectively, leading to cell death.

Recent advances suggest that metabolic pathways are involved in cell fate determination including the execution of cell death. The relationship between metabolism and cell death is widely explored in ferroptosis, a form of cell death driven by iron-dependent lipid peroxidation due to dysregulation of amino acids and polyunsaturated fatty acid metabolism (Stockwell et al., 2017). It is recognized that aberrant metabolism promotes cell death through mechanisms including energy exhaustion, oxidative stress, and release of damage-associated molecular patterns (Vringer and Tait, 2023; Yang et al., 2024). Although multiple cell death checkpoints in the TNFα pathway have been identified, they often rely on signal transduction or posttranslational modifications that are independent of metabolic processes (Tan et al., 2020; Xu et al., 2018; Zhang et al., 2023a). How metabolic activities orchestrate cell death checkpoints in the TNFα pathway remains an important yet unexplored question.

Adenosine kinase (ADK) is an evolutionarily conserved metabolic enzyme that converts adenosine to adenosine monophosphate (AMP) (Boison, 2006). Adenosine exists in all living systems and functions in both adenosine receptor–dependent and adenosine receptor–independent manners (Boison and Yegutkin, 2019). In mammals, there are four adenosine receptors including A_1_R, A_2_AR, A_2_BR, and A_3_R, and each of them activates different signaling pathways when stimulated by extracellular adenosine (Chen et al., 2013). Adenosine also regulates bioenergy and biochemistry in various manners independent of adenosine receptors (Boison, 2013), including promoting the proper operation of the methionine cycle. In the methionine cycle, S-adenosylmethionine (SAM) is converted to S-adenosylhomocysteine (SAH) after its methyl group is transferred to cellular targets; afterward, SAH is hydrolyzed to adenosine and homocysteine, which are then removed by ADK and recycled to methionine and SAM, respectively (Boison, 2013). SAH potently suppresses the transmethylation reaction by product inhibition, implying that ADK-mediated adenosine removal is indispensable for the activity of the methionine cycle and transmethylation reaction.

The pharmacological utilization of adenosine or its derivatives has been explored for a long time and exhibits efficacy in a wide range of diseases, such as arrhythmia and neurodegeneration, mainly through adenosine receptor–dependent mechanisms (Bennet and Drury, 1931; Eltzschig, 2009; Meng et al., 2019). Pharmacological effects of adenosine can also be achieved by ADK inhibition, which elevates endogenous adenosine level by blocking its clearance (Xu et al., 2017). Notably, clinical application of ADK inhibition–based therapy is severely hindered by its potential cytotoxicity (Boison, 2013). It has been observed that adenosine overdose triggers apoptosis in several cell types (Peyot et al., 2000; Zhao et al., 2002). Moreover, systemic ADK inhibition results in liver injury (Boison, 2013), which is further supported by the findings that either global or hepatocyte-specific ADK gene knockout leads to dramatic liver damage and premature death in mouse models (Boison et al., 2002; Li et al., 2023). However, the mechanism underlying adenosine overdose–induced cytotoxicity remains unclear.

In this study, we identified ADK as a suppressor of TNFα-induced cell death through the action of adenosine metabolic clearance. Both ADK knockout and adenosine treatment aggravate cell death in response to TNFα stimulation. Adenosine accumulation, caused by ADK depletion, decreases the intracellular SAM/SAH ratio and inhibits SAM-mediated methylation potential. We found that in steady state, RIPK1 is constitutively symmetrically dimethylated at R606, a critical residue in the DD that is involved in DD-mediated RIPK1 dimerization. We show that RIPK1 R606 methylation is mediated by protein arginine methyltransferase 5. Upon ADK depletion, adenosine is increased and prevents RIPK1 R606 methylation, which releases R606 to facilitate DD-mediated RIPK1 homodimerization and activation, leading to cell death in response to TNFα sensing. Importantly, RIPK1 kinase inactivation diminishes ADK knockout– or ADK inhibition–induced spontaneous hepatocyte death, as well as subsequent tissue homeostasis disruption and premature death. Furthermore, ADK is decreased in liver ischemia–reperfusion, which exacerbates RIPK1-dependent liver injury and worsens prognosis of patients that undergo liver surgery.

## Results

### Identification of ADK as a suppressor of TNFα-induced RIPK1-driven cell death

**Figure 1.**
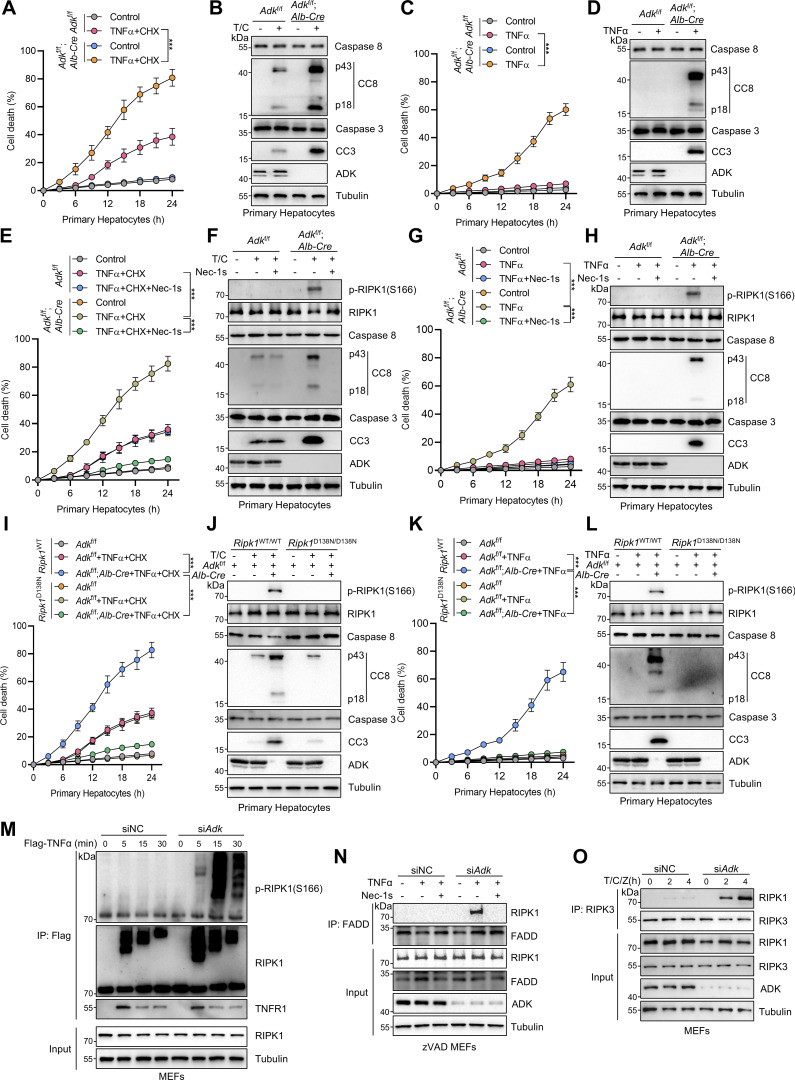
**Identification of ADK as a suppressor of TNFα-induced RIPK1-driven cell death. (A and B)** Primary hepatocytes from *Adk*^f/f^ or *Adk*^f/f^;*Alb-Cre* mice were treated with CHX (1 μM)/TNFα (10 ng/ml) (T/C) for the indicated time (A) or 12 h (B). Cell death was measured by the SYTOX Green positivity assay (A). The levels of cleaved caspase-8 (CC8), cleaved caspase-3 (CC3), and ADK were determined by immunoblotting (B). Scale bar: 50 μm. **(C and D)** Primary hepatocytes from *Adk*^f/f^ or *Adk*^f/f^;*Alb-Cre* mice were treated with TNFα (10 ng/ml) for the indicated time (C) or 12 h (D). Cell death was measured by the SYTOX Green positivity assay (C). The levels of CC8, CC3, and ADK were determined by immunoblotting (D). **(E and F)** Primary hepatocytes from *Adk*^f/f^ or *Adk*^f/f^;*Alb-Cre* mice were treated with CHX (1 μM)/TNFα (10 ng/ml) for the indicated time (E) or 12 h (F) in the presence or absence of Nec-1s (10 μM). Cell death was measured by the SYTOX Green positivity assay (E). The levels of p-RIPK1(S166), CC8, CC3, and ADK were determined by immunoblotting (F). **(G and H)** Primary hepatocytes from *Adk*^f/f^ or *Adk*^f/f^;*Alb-Cre* mice were treated with TNFα (10 ng/ml) for the indicated time (G) or 12 h (H) in the presence or absence of Nec-1s (10 μM). Cell death was measured by the SYTOX Green positivity assay (G). The levels of p-RIPK1(S166), CC8, CC3, and ADK were determined by immunoblotting (H). **(I and J)** Primary hepatocytes from *Adk*^f/f^, *Adk*^f/f^;*Alb-Cre*, and *Adk*^f/f^;*Alb-Cre*;*Ripk1*^D138N/D138N^ mice were treated with CHX (1 μM)/TNFα (10 ng/ml) for the indicated time (I) or 12 h (J). Cell death was measured by the SYTOX Green positivity assay (I). The levels of p-RIPK1(S166), CC8, CC3, and ADK were determined by immunoblotting (J). **(K and L)** Primary hepatocytes from *Adk*^f/f^, *Adk*^f/f^;*Alb-Cre*, and *Adk*^f/f^;*Alb-Cre*;*Ripk1*^D138N/D138N^ mice were treated with TNFα (10 ng/ml) for the indicated time (K) or 12 h (L). Cell death was measured by the SYTOX Green positivity assay (K). The levels of p-RIPK1(S166), CC8, CC3, and ADK were determined by immunoblotting (L). **(M)** MEFs transfected with siRNAs of negative control (siNC) or siRNAs targeting ADK (si*Adk*) were stimulated by Flag-TNFα (100 ng/ml) for the indicated time. The TNFR1 signaling complex was immunoprecipitated using an anti-Flag antibody. The complexes were analyzed by immunoblotting using anti-p-S166 RIPK1 antibody and other antibodies as indicated. **(N)** MEFs transfected with siNC or si*Adk* were preincubated with zVAD.fmk (10 μM) in the presence or absence of Nec-1s (10 μM) for 0.5 h and then stimulated with 10 ng/ml TNFα for 12 h. The complex II was isolated by FADD immunoprecipitation, and RIPK1 binding was revealed by immunoblotting. **(O)** MEFs transfected with siNC or si*Adk* were pretreated with CHX (C, 2 μg/ml) and zVAD.fmk (Z, 10 μM) for 0.5 h followed by 10 ng/ml TNFα (T) for the indicated time. The necrosome was isolated by immunoprecipitation of RIPK3, and RIPK1 binding was revealed by immunoblotting. Data are represented as the mean ± SD (A, C, E, G, I, and K). Data are representative of *n* = 3 independent experiments (A–O). Statistical significance was determined using two-way ANOVA with post hoc Bonferroni’s test (A, C, E, G, I, and K). ***P < 0.001. Source data are available for this figure: SourceData F1.

**Figure S1.**
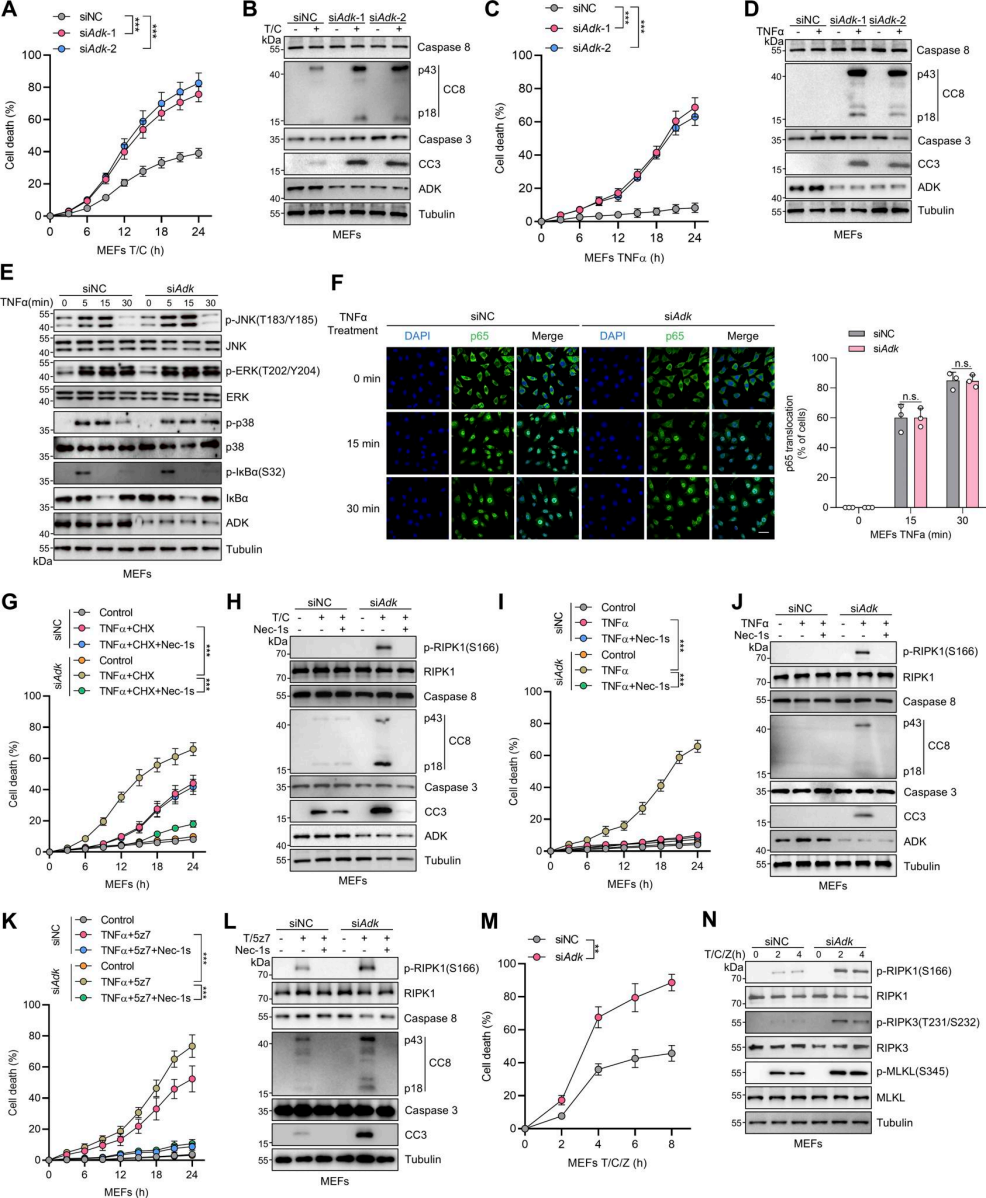
**ADK inhibits RIPK1 kinase–driven cell death induced by TNFα. (A and B)** MEFs transfected with siNC or si*Adk* were treated with CHX (1 μM)/TNFα (10 ng/ml) for the indicated time (A) or 12 h (B). Cell death was measured by the SYTOX Green positivity assay (A). The levels of CC8, CC3, and ADK were determined by immunoblotting (B). **(C and D)** MEFs transfected with siNC or si*Adk* were treated with TNFα (10 ng/ml) for the indicated time (C) or 12 h (D). Cell death was measured by the SYTOX Green positivity assay (C). The levels of CC8, CC3, and ADK were determined by immunoblotting (D). **(E)** MEFs transfected with siNC or si*Adk* were treated with TNFα (10 ng/ml) for the specified durations (min). Activation of MAPK or NF-κB pathways was analyzed with immunoblotting. **(F)** MEFs transfected with siNC or si*Adk* were treated with TNFα (10 ng/ml) for the specified durations. Then, the p65 nucleus translocation level of MEFs was detected by immunofluorescence. Scale bar: 20 μm. **(G and H)** MEFs transfected with siNC or si*Adk* were treated with CHX (1 μM)/TNFα (10 ng/ml) for the indicated time (G) or 12 h (H) in the presence or absence of Nec-1s (10 μM). Cell death was measured by the SYTOX Green positivity assay (G). The levels of p-RIPK1(S166), CC8, CC3, and ADK were determined by immunoblotting (H). **(I and J)** MEFs transfected with siNC or si*Adk* were treated with TNFα (10 ng/ml) for the indicated time (I) or 12 h (J) in the presence or absence of Nec-1s (10 μM). Cell death was measured by the SYTOX Green positivity assay (I). The levels of p-RIPK1(S166), CC8, CC3, and ADK were determined by immunoblotting (J). **(K and L)** MEFs transfected with siNC or si*Adk* were treated with 5z7 (500 nM)/TNFα (10 ng/ml) for the indicated time (K) or 12 h (L) in the presence or absence of Nec-1s (10 μM). Cell death was measured by the SYTOX Green positivity assay (K). The levels of p-RIPK1(S166), CC8, and CC3 were determined by immunoblotting (L). **(M and N)** MEFs transfected with siNC or si*Adk* were treated with CHX (2 μg/ml) and zVAD.fmk (Z, 10 μM) for 0.5 h followed by 10 ng/ml TNFα for the indicated time. Cell death was measured by the SYTOX Green positivity assay (M). The levels of p-S166 RIPK1, p-T231/S232 RIPK3, and p-S345 MLKL were determined by immunoblotting (N). Data are represented as the mean ± SD (A, C, F, G, I, K, and M). Data are representative of *n* = 3 independent experiments (A–N). Statistical significance was determined using two-way ANOVA with post hoc Bonferroni’s test (A, C, F, G, I, K, and M). **P < 0.01; ***P < 0.001. Source data are available for this figure: SourceData FS1.

In our previous study, we conducted siRNA screen to identify metabolic enzymes that regulate primary mouse hepatocyte apoptosis induced by combined TNFα and cycloheximide (CHX) treatment, a well-known paradigm through which apoptosis is induced and mediated by caspase-8 (Zhang et al., 2023c). We found that silencing ADK, which is highly enriched in hepatocytes (Boison, 2013; Li et al., 2023), promoted TNFα/CHX-triggered apoptosis (Zhang et al., 2023c). To confirm the role of ADK in TNFα-induced apoptosis, we generated hepatocyte-specific ADK knockout mice by crossing *Adk*^f/f^ mice with *Alb-Cre* Tg mice. Compared with isolated wild-type (WT) hepatocytes, ADK knockout hepatocytes underwent more drastic apoptosis under TNFα/CHX stimulation (Fig. 1, A and B). We also tested whether ADK regulates apoptosis induced by TNFα alone, a more physiologically relevant condition (Zhang et al., 2023c). TNFα alone did not trigger apparent apoptosis in WT hepatocytes (Zhang et al., 2023c) (Fig. 1, C and D); in contrast, TNFα induced substantial apoptosis in ADK knockout hepatocytes (Fig. 1, C and D). We then explored whether ADK affects the TNFα pathway in mouse embryonic fibroblasts (MEFs), a well-established cell line for investigating TNFα-induced cell death (Xu et al., 2018). Similar as that in hepatocytes, ADK knockdown sensitized MEFs to both TNFα/CHX- and TNFα-triggered apoptosis (Fig. S1, A–D). Thus, ADK potently suppresses TNFα-induced apoptosis.

We then investigated how ADK suppresses apoptosis triggered by TNFα. We first tested whether ADK regulates NF-κB and MAPK pathways, both of which are activated by TNFα and confer prosurvival signals (Xu et al., 2021). We observed that NF-κB and MAPK pathways were unaffected by ADK knockdown, as revealed by the levels of proteins involved in these pathways and the nuclear translocation of p65 (Fig. S1, E and F). TNFα can induce two types of apoptosis, including RIPK1 kinase–independent apoptosis (RIA) and RIPK1 kinase–dependent apoptosis (RDA), and RIA can be converted to RDA when RIPK1 is prone to activation (Xu et al., 2021; Dziedzic et al., 2018). Since ADK does not affect NF-κB or MAPK, we questioned whether ADK modulates RIPK1 activation and thus converts apoptosis modes. In line with the notion that TNFα/CHX typically induces RIA in normal conditions (Zhang et al., 2023c), specific RIPK1 kinase inhibitor Nec-1s did not limit TNFα/CHX-triggered apoptosis in WT hepatocytes; however, Nec-1s effectively protected ADK knockout hepatocytes from TNFα/CHX-triggered apoptosis (Fig. 1, E and F). Consistently, when stimulated by TNFα/CHX, RIPK1 activation, as indicated by S166 phosphorylation (Xu et al., 2018), was only detected in ADK knockout, but not WT, hepatocytes, which was also blocked by Nec-1s (Fig. 1 F). Similar results were obtained when hepatocytes were treated with TNFα alone (Fig. 1, G and H). Moreover, Nec-1s also blocked apoptosis and RIPK1 activation in ADK knockdown MEFs (Fig. S1, G–J). To consolidate these findings, we crossed hepatocyte ADK knockout mice with RIPK1 kinase-dead D138N mutation knockin mice (Zhang et al., 2023c). Consistently, genetic RIPK1 inactivation blocked TNFα/CHX- or TNFα-induced apoptosis and RIPK1 activation in ADK-deficient hepatocytes (Fig. 1, I–L). We found that ADK knockdown facilitated RIPK1 activation in complex I (Fig. 1 M). Furthermore, in response to TNFα, the interaction between RIPK1 and FADD, a marker of proapoptotic complex II formation (Wang et al., 2008), was enhanced by ADK knockdown, which was reversed by Nec-1s (Fig. 1 N).

The combination of TNFα and TAK1 inhibitor 5Z-7-oxozeaenol (5z7) is an established model to induce RDA in WT cells (Geng et al., 2017). We found that ADK-deficient cells exhibited increased sensitization to RDA and enhanced activation of caspases triggered by TNFα/5z7 stimulation (Fig. S1, K and L). Upon caspase inhibition mediated by zVAD.fmk (zVAD), RIPK1 kinase activation facilitates the formation of the RIPK1-RIPK3 complex (Cho et al., 2009), known as necrosome, where RIPK3 is activated and phosphorylates MLKL to promote necroptosis execution (Sun et al., 2012). ADK knockdown also sensitized cells to TNFα/CHX/zVAD-induced necroptosis (Fig. S1 M), which was evidenced by increased necroptosis markers, including p-T231/S232 RIPK3 and p-S345 MLKL (Fig. S1 N) (Sun et al., 2012), as well as necrosome formation (Fig. 1 O). Thus, ADK inhibits RIPK1 kinase–driven cell death induced by TNFα.

### ADK suppresses TNFα-induced cell death through its kinase activity–mediated adenosine metabolic clearance

**Figure 2.**
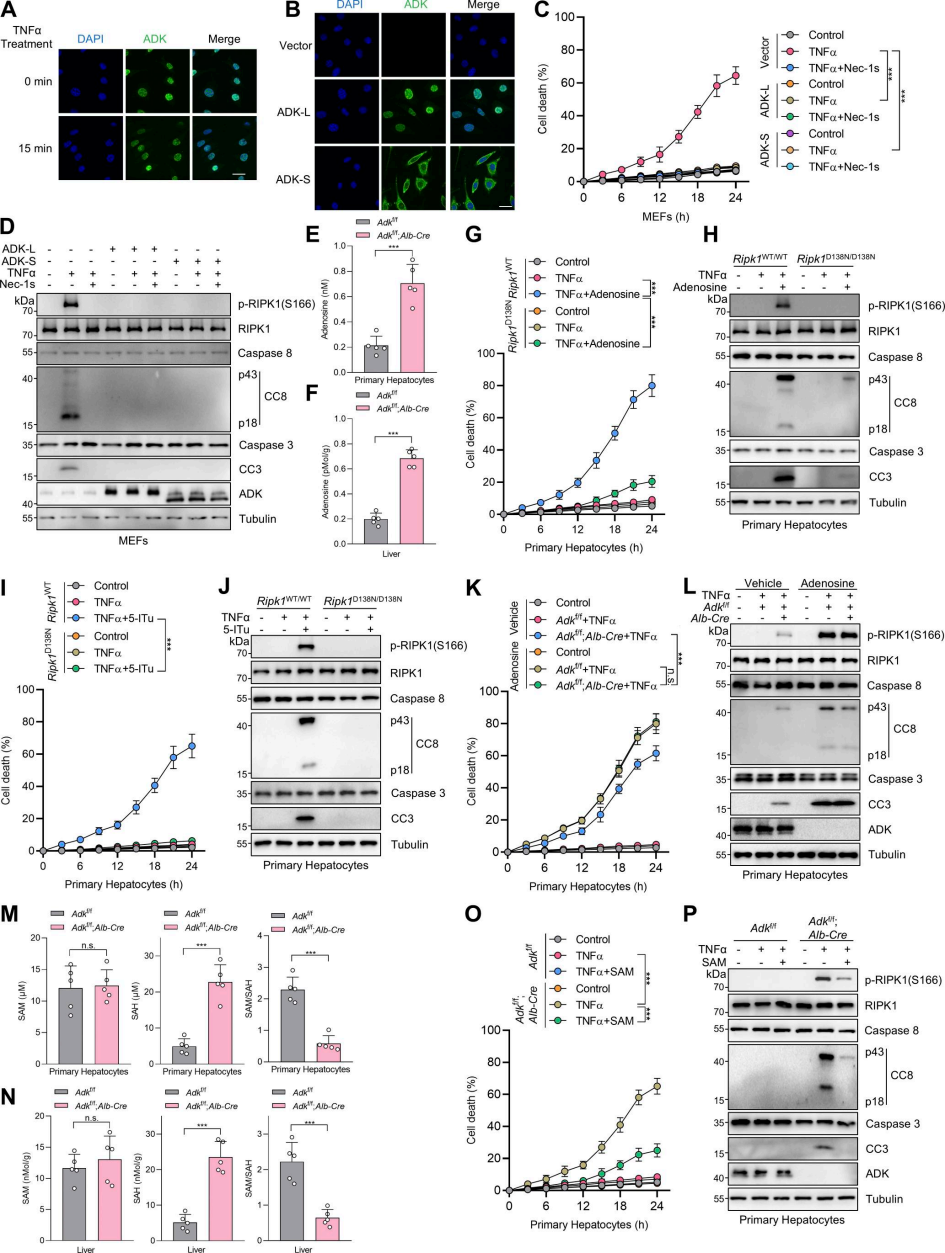
**ADK prevents TNFα-induced cell death by reducing adenosine levels, which in turn licenses the methionine cycle and transmethylation reactions. (A)** MEFs were treated with TNFα (10 ng/ml) for the specified durations (min). Then, the ADK subcellular localization was detected by immunofluorescence. **(B)** MEFs were transfected with lentivirus encoding ADK-L or ADK-S. Endogenous ADK was silenced with siRNA targeting 3′UTR. Then, the ADK subcellular localization was detected with immunofluorescence. **(C and D)** MEFs were transfected with lentivirus encoding ADK-L or ADK-S. Endogenous ADK was silenced with siRNA targeting 3′UTR. These cells were treated with TNFα (10 ng/ml) for the indicated time (C) or 12 h (D) in the presence or absence of Nec-1s (10 μM). Cell death was measured by the SYTOX Green positivity assay (C). The levels of p-RIPK1(S166), CC8, CC3, and ADK were determined by immunoblotting (D). **(E and F)** Adenosine concentrations in primary hepatocytes or liver tissue of *Adk*^f/f^ or *Adk*^f/f^;*Alb-Cre* mice were measured. **(G and H)** Primary hepatocytes from *Adk*^f/f^ or *Adk*^f/f^;*Alb-Cre* mice were treated with TNFα (10 ng/ml) for the indicated time (G) or 12 h (H) in the presence or absence of adenosine (1 mM). Cell death was measured by the SYTOX Green positivity assay (G). The levels of p-RIPK1(S166), CC8, and CC3 were determined with immunoblotting (H). **(I and J)** Primary hepatocytes from *Ripk1*^WT/WT^ or *Ripk1*^D138N/D138N^ mice were treated with TNFα (10 ng/ml) for the indicated time (I) or 12 h (J) in the presence or absence of 5-ITu (20 μM). Cell death was measured by the SYTOX Green positivity assay (I). The levels of p-RIPK1(S166), CC8, and CC3 were determined by immunoblotting (J). **(K and L)** Primary hepatocytes from *Adk*^f/f^ or *Adk*^f/f^;*Alb-Cre* mice were treated with TNFα (10 ng/ml) for the indicated time (K) or 12 h (L) in the presence or absence of adenosine (1 mM). Cell death was measured by the SYTOX Green positivity assay (K). The levels of p-RIPK1(S166), CC8, and CC3 were determined by immunoblotting (L). **(M and N)** Concentrations of SAM and SAH in primary hepatocytes or liver tissue of *Adk*^f/f^ or *Adk*^f/f^;*Alb-Cre* mice were measured. **(O and P)** Primary hepatocytes from *Adk*^f/f^ or *Adk*^f/f^;*Alb-Cre* mice were treated with TNFα (10 ng/ml) for the indicated time (O) or 12 h (P) in the presence or absence of SAM (100 μM). Cell death was measured by the SYTOX Green positivity assay (O). The levels of p-RIPK1(S166), CC8, CC3, and ADK were determined by immunoblotting (P). Data are represented as the mean ± SD (C, E–G, I, K, and M–O). Data are representative of *n* = 3 independent experiments (A–P). Statistical significance was determined using two-tailed unpaired Student’s *t* test (E, F, M, and N) or two-way ANOVA with post hoc Bonferroni’s test (C, G, I, K, and O). ***P < 0.001. Source data are available for this figure: SourceData F2.

We then examined how ADK prevents RIPK1-driven cell death. ADK consists of two transcript variants, and the longer one, ADK-L, has an extra nuclear localization signal region than the shorter one, ADK-S (Boison, 2013). We noticed that hepatocytes express more ADK-L than ADK-S, while MEFs exclusively express ADK-L from the ADK band positions in immunoblotting (Fig. 1 B and Fig. S1 B). Consistently, immunostaining revealed that the ADK protein locates in the nucleus of MEFs both in steady state and after TNFα stimulation (Fig. 2 A). To understand whether regulation of ADK on apoptosis is controlled by its subcellular location, we constructed reconstituted cells with either ADK-L or ADK-S. Interestingly, both nuclear ADK-L and cytoplasm ADK-S effectively suppressed RIPK1 activation and apoptosis in MEFs stimulated by TNFα (Fig. 2, B–D).

**Figure S2.**
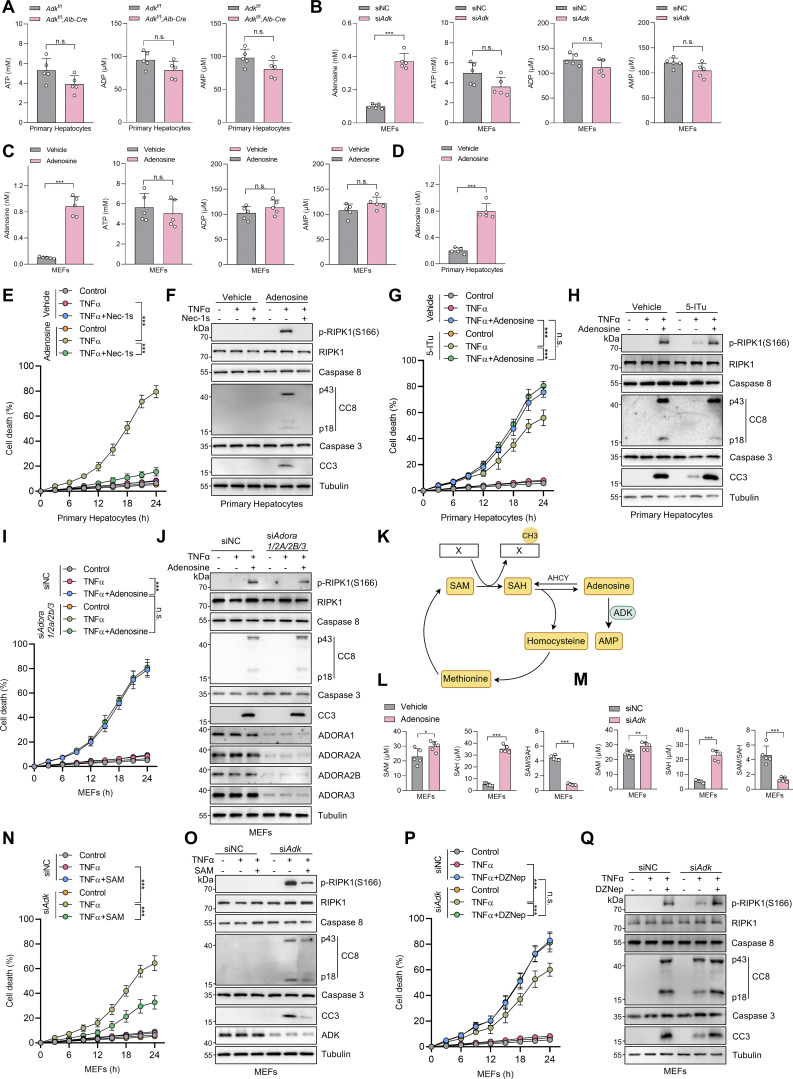
**Adenosine is indispensable for ADK in regulating TNFα-induced cell death. (A)** Primary hepatocytes were isolated from *Adk*^f/f^ or *Adk*^f/f^;*Alb-Cre* mice. Intracellular ATP, ADP, and AMP levels were measured. **(B)** MEFs were transfected with siNC or si*Adk*. Intracellular adenosine, ATP, ADP, and AMP levels were measured. **(C)** MEFs were treated with vehicle or adenosine (1 mM). After 1 h, intracellular adenosine, ATP, ADP, and AMP levels were measured. **(D)** Primary hepatocytes were isolated from WT mice and were treated with vehicle or adenosine (1 mM). After 1 h, intracellular adenosine level was measured. **(E and F)** Primary hepatocytes were treated with TNFα (10 ng/ml) for the indicated time (E) or 12 h (F) in the presence or absence of adenosine (1 mM) or Nec-1s (10 μM). Cell death was measured by the SYTOX Green positivity assay (E). The levels of p-RIPK1(S166), CC8, and CC3 were determined by immunoblotting (F). **(G and H)** Primary hepatocytes were treated with TNFα (10 ng/ml) for the indicated time (G) or 12 h (H) in the presence or absence of 5-ITu (20 μM) or adenosine (1 mM). Cell death was measured by the SYTOX Green positivity assay (G). The levels of p-RIPK1(S166), CC8, and CC3 were determined by immunoblotting (H). **(I and J)** MEFs transfected with siNC or si*Adora1/2a/2b/3* were treated with TNFα (10 ng/ml) for the indicated time (I) or 12 h (J) in the presence or absence of adenosine (1 mM). Cell death was measured by the SYTOX Green positivity assay (I). The levels of p-RIPK1(S166), CC8, CC3, and adenosine receptors were determined by immunoblotting (J). **(K)** Schematic diagram of ADK in the regulation of the methionine cycle. **(L)** MEFs were treated with vehicle or adenosine (1 mM). After 1 h, intracellular SAM and SAH were measured and their ratio was calculated. **(M)** MEFs were transfected with siNC or si*Adk*. Intracellular SAM and SAH were measured, and their ratio was calculated. **(N and O)** MEFs transfected with siNC or si*Adk* were treated with TNFα (10 ng/ml) for the indicated time (N) or 12 h (O) in the presence or absence of SAM (100 μM). Cell death was measured by the SYTOX Green positivity assay (N). The levels of p-RIPK1(S166), CC8, CC3, and ADK were determined by immunoblotting (O). **(P and Q)** MEFs transfected with siNC or si*Adk* were treated with TNFα (10 ng/ml) for the indicated time (P) or 12 h (Q) in the presence or absence of DZNep (10 μM). Cell death was measured by the SYTOX Green positivity assay (P). The levels of p-RIPK1(S166), CC8, CC3, and ADK were determined by immunoblotting (Q). Data are represented as mean ± SD (A–E, G, I, L, M, N, and P). Data are representative of *n* = 3 independent experiments (A–Q). Statistical significance was determined using unpaired two-tailed Student’s *t* test (A–D, L, and M) or two-way ANOVA with post hoc Bonferroni’s test (E, G, I, N, and P). *P < 0.05; **P < 0.01; ***P < 0.001. Source data are available for this figure: SourceData FS2.

Considering that TNFα-induced apoptosis is processed in the cytoplasm, the above findings suggest that ADK regulates the TNFα pathway with mediator(s) that can translocate between the nucleus and the cytoplasm. As a metabolic enzyme, ADK transfers a phosphate group from adenosine triphosphate (ATP) to adenosine, which yields adenosine diphosphate (ADP) and AMP (Kornberg and Pricer, 1951). Thus, ADK might repress TNFα-induced apoptosis by changing intracellular concentrations of adenosine, AMP, ADP, or ATP, all of which are small-molecule metabolites that can translocate between the nucleus and cytoplasm (Wright et al., 2016). To test this hypothesis, we measured concentrations of these metabolites in control or ADK-deficient cells. However, the intracellular levels of AMP, ADP, or ATP were nearly unaffected by ADK silencing (Fig. S2, A and B), which is consistent with previous studies (Fredholm et al., 2005; Boison, 2013). In contrast, ADK deficiency greatly elevated adenosine levels (Fig. 2, E and F; and Fig. S2 B). Notably, adenosine has a much lower intracellular concentration than the other three metabolites (Fig. 2, E and F; and Fig. S2, A and B), which might explain why adenosine concentration is much more sensitive to the change of ADK expression levels (Boison, 2013).

We then tested whether ADK prevents TNFα-induced cell death by adenosine clearance. Adding adenosine to cell culture medium increased intracellular adenosine concentration (Fig. S2, C and D) and sensitized cells to TNFα-induced apoptosis and RIPK1 activation (Fig. S2, E and F), which was reversed by RIPK1 kinase inactivation (Fig. S2, E and F). Consistently, 5-iodotubercidin (5-ITu), an ADK inhibitor (Bullough et al., 1994), also promoted RIPK1 kinase–driven TNFα-induced apoptosis (Fig. 2, I and J; and Fig. S2, G and H). When adenosine was pretreated, neither knockout nor inhibition of ADK further sensitized cells to TNFα-induced apoptosis (Fig. 2, K and L; and Fig. S2, G and H). Thus, these data suggest that ADK suppresses TNFα-induced cell death by preventing adenosine accumulation.

### Adenosine promotes TNFα-induced cell death by inhibiting SAM-dependent transmethylation

We subsequently investigated how adenosine facilitates RIPK1 activation and apoptosis. Since adenosine can be transported into or out of cells (Cheu et al., 2023), and activating adenosine receptors is the best-characterized function of adenosine (Boison, 2013), we first explored whether adenosine promotes RIPK1 activation by agonizing specific adenosine receptors. Efficient knockdown of all the four adenosine receptors did not prevent cells from the sensitization of adenosine-induced apoptosis (Fig. S2, I and J), suggesting that adenosine modulates TNFα-induced cell death in an adenosine receptor–independent manner.

As the levels of AMP, ADP, and ATP were not changed significantly by ADK deficiency, functions of intracellular adenosine including energy supply and biomacromolecule synthesis were unlikely involved. Thus, we paid attention to the methionine cycle, which is essential for transmethylation reaction and regulated by intracellular adenosine concentrations (Williams-Karnesky et al., 2013) (Fig. S2 K). To this end, we measured the concentrations of critical methionine cycle mediators, including SAM and SAH. The levels of SAH, but not SAM, were substantially increased upon ADK depletion or adenosine treatment, which resulted in sharply decreased SAM/SAH ratios (Fig. 2, M and N; and Fig. S2, L and M), suggestive of reduced transmethylation potential. Furthermore, supplementation of SAM reversed RIPK1 activation and apoptosis caused by ADK depletion or adenosine supplementation (Fig. 2, O and P; and Fig. S2, N and O). These results indicate that transmethylation reaction is involved in regulating RIPK1 activation. Consistent with this notion, transmethylation reaction inhibitor DZNep, which is known to block AHCY that catalyzes SAH hydrolysis (Jiang et al., 2023), not only sensitized cells to TNFα stimulation, but also abolished the function of ADK in suppressing RIPK1 activation and apoptosis (Fig. S2, P and Q). Thus, adenosine accumulation promotes TNFα-induced RIPK1 activation and apoptosis through abrogating SAM-dependent transmethylation reaction.

### ADK licenses constitutive RIPK1 symmetric dimethylation at R606

**Figure 3.**
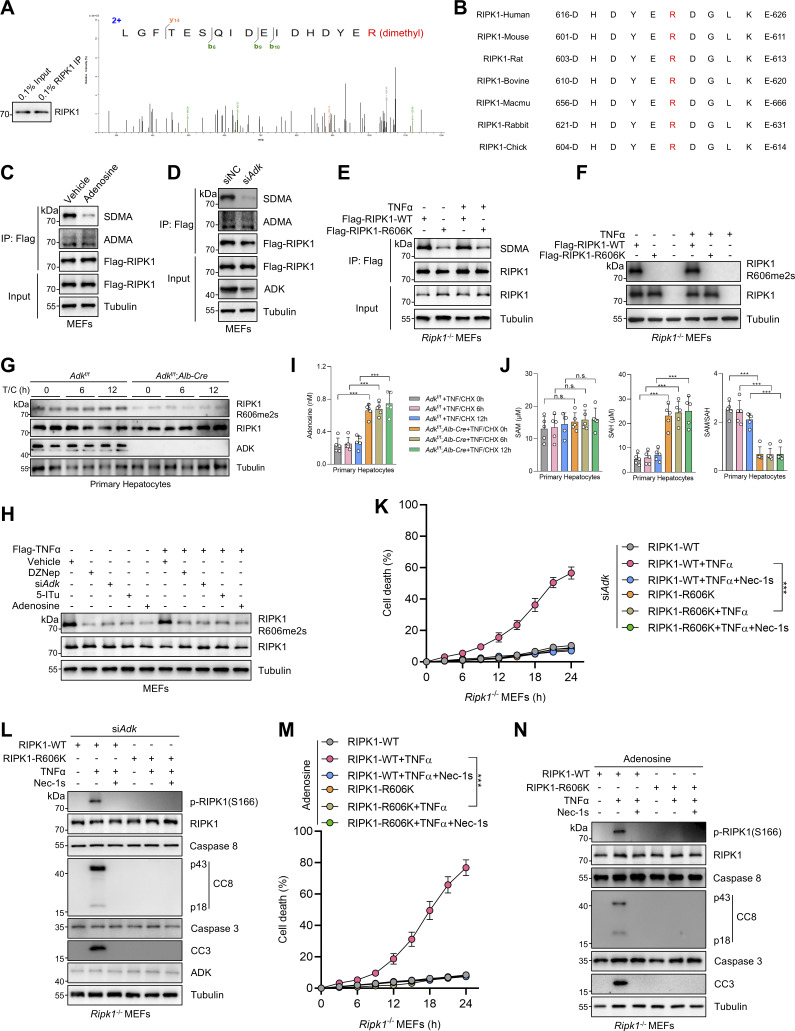
**ADK licenses constitutive RIPK1 symmetric dimethylation at R606, which inhibits TNFα-induced cell death. (A)** RIPK1 was immunoprecipitated from primary mouse hepatocytes and was analyzed with mass spectrometry. Left: Enrichment efficiency of RIPK1 immunoprecipitation was confirmed with immunoblotting. Right: The mass spectrum data revealed that endogenous mouse RIPK1 was dimethylated at R606. **(B)** Amino acid sequences at R606 in RIPK1 DD across various mammalian species were aligned. R606 was highlighted in red. **(C)***Ripk1*^−/−^ MEFs were reconstituted with Flag-RIPK1 by lentivirus. The cells were treated with or without adenosine (1 mM). RIPK1 was immunoprecipitated using anti-Flag antibody, and SDMA or ADMA levels were determined with immunoblotting. **(D)***Ripk1*^−/−^ MEFs were reconstituted with Flag-RIPK1 by lentivirus. The cells were transfected with siNC or si*Adk*. RIPK1 was immunoprecipitated using anti-Flag antibody, and SDMA or ADMA levels were determined with immunoblotting. **(E)***Ripk1*^−/−^ MEFs were reconstituted with WT or R606K mutant Flag-RIPK1 by lentivirus. The cells were treated with TNFα (10 ng/ml) for 15 min. The level of SDMA on RIPK1 was determined by immunoprecipitation and immunoblotting. **(F)***Ripk1*^−/−^ MEFs were reconstituted with WT or R606K mutant Flag-RIPK1 by lentivirus. The cells were treated with TNFα (10 ng/ml) for 15 min. The level of RIPK1 R606me2s was determined with immunoblotting. **(G)** Primary hepatocytes from *Adk*^f/f^ or *Adk*^f/f^;*Alb-Cre* mice were treated with CHX (1 μM)/TNFα (10 ng/ml) for the indicated time. RIPK1 R606me2s, RIPK1, and ADK levels were analyzed with immunoblotting. **(H)** MEFs transfected with or without si*Adk* were stimulated with Flag-TNFα (10 ng/ml) for 15 min after the pretreatment of DZNep (10 μM), 5-ITu (20 μM), or adenosine (1 mM) for 1 h. Complex I was immunoprecipitated using anti-Flag antibody, and RIPK1 R606me2s level in complex I or whole-cell lysates was determined with immunoblotting. **(I and J)** Primary hepatocytes from *Adk*^f/f^ or *Adk*^f/f^;*Alb-Cre* mice were treated with CHX (1 μM)/TNFα (10 ng/ml) for the indicated time. Adenosine levels in the cells were measured and compared (I). SAM, SAH levels, and their ratios in the cells were measured and compared (J). **(K and L)***Ripk1*^−/−^ MEFs were reconstituted with WT or R606K mutant Flag-RIPK1 by lentivirus. The cells were transfected with si*Adk* and subsequently treated with TNFα (10 ng/ml) for the indicated time (K) or 12 h (L) in the presence or absence of Nec-1s (10 μM). Cell death was measured by the SYTOX Green positivity assay (K). The levels of p-RIPK1(S166), CC8, CC3, and ADK were determined by immunoblotting (L). **(M and N)***Ripk1*^−/−^ MEFs were reconstituted with WT or R606K mutant Flag-RIPK1 by lentivirus. The cells were treated with adenosine (1 mM) and subsequently treated with TNFα (10 ng/ml) for the indicated time (M) or 12 h (N) in the presence or absence of Nec-1s (10 μM). Cell death was measured by the SYTOX Green positivity assay (M). The levels of p-RIPK1(S166), CC8, and CC3 were determined by immunoblotting (N). Data are represented as the mean ± SD (I–K and M). Data are representative of *n* = 3 independent experiments (A–N). Statistical significance was determined using two-way ANOVA with post hoc Bonferroni’s test (I–K and M). ***P < 0.001. Source data are available for this figure: SourceData F3.

We then sought to identify the key methylation targets that are involved in RIPK1 activation. As RIPK1 is modulated by various posttranslational modifications (Xu et al., 2018; Yan et al., 2022; Zhang et al., 2023a), we first assessed whether RIPK1 can be methylated. Mass spectrometry analysis revealed that endogenous RIPK1 in primary mouse hepatocytes was dimethylated at R606 (corresponding to human R621) (Fig. 3 A), which is highly conserved among species (Fig. 3 B). For dimethylated arginine, if one methyl group is added to each of the terminal guanidino nitrogens, the modification is denoted as symmetrically dimethylated arginine (SDMA); alternatively, if two methyl groups are added to the same nitrogens, it is denoted as asymmetrically dimethylated arginine (ADMA) (Bedford and Clarke, 2009). With antibodies that specifically recognize either SDMA or ADMA, we found that RIPK1 was modified by SDMA, which was repressed upon ADK deletion or adenosine treatment; in contrast, the ADMA signal is ambiguous at RIPK1 band position and unaffected by ADK deletion or adenosine treatment (Fig. 3, C and D). Mutation of arginine (R) to lysine (K) is commonly used as unmethylated mimetic because it preserves positive charge but cannot be modified by arginine methylation (Jiang et al., 2023). SDMA signal decreased in R606K RIPK1-reconstituted *Ripk1*^−/−^ MEFs than in that reconstituted with WT RIPK1 (Fig. 3 E). Notably, SDMA of RIPK1 was unaffected by TNFα treatment (Fig. 3 E), suggesting that RIPK1 arginine methylation was processed in steady state independently of the TNFα pathway. Furthermore, SAMA did not completely disappear in R606K RIPK1 (Fig. 3 E), which suggested that R606 was not the only dimethylated arginine of RIPK1. We generated an antibody that recognizes SDMA at RIPK1 R606 (RIPK1 R606me2s) and validated its specificity with RIPK1 knockout and R606K mutation (Fig. 3 F). With this antibody, we found that RIPK1 R606me2s modification levels were decreased upon ADK knockout or inhibition but were unaffected by TNFα (Fig. 3, G and H). Notably, adenosine level was increased, while SAM/SAH ratio levels were also decreased upon ADK knockout but were unaffected by TNFα/CHX stimulation (Fig. 3, I and J). Thus, RIPK1 is constitutively symmetrically dimethylated at R606 in steady state, which is prevented by adenosine accumulation.

### ADK suppresses TNFα-induced cell death by licensing RIPK1 R606me2s modification

We then explored whether R606 is the critical residue that underlies the regulation of ADK on TNFα-induced cell death. After ADK was depleted, compared with WT RIPK1-reconstituted MEFs, RIPK1 activation and apoptosis were reduced by R606K mutation (Fig. 3, K and L). Consistently, R606K mutation also abrogated the apoptosis sensitization mediated by adenosine treatment (Fig. 3, M and N). Thus, R606K mutation suppresses RIPK1 activation and abolished the cell death sensitization mediated by adenosine accumulation, which suggests that R606 is indispensable for RIPK1 activation and that either methylation or mutation of R606 inhibits its function in RIPK1-driven cell death.

### R606me2s suppresses DD-mediated RIPK1 dimerization

**Figure 4.**
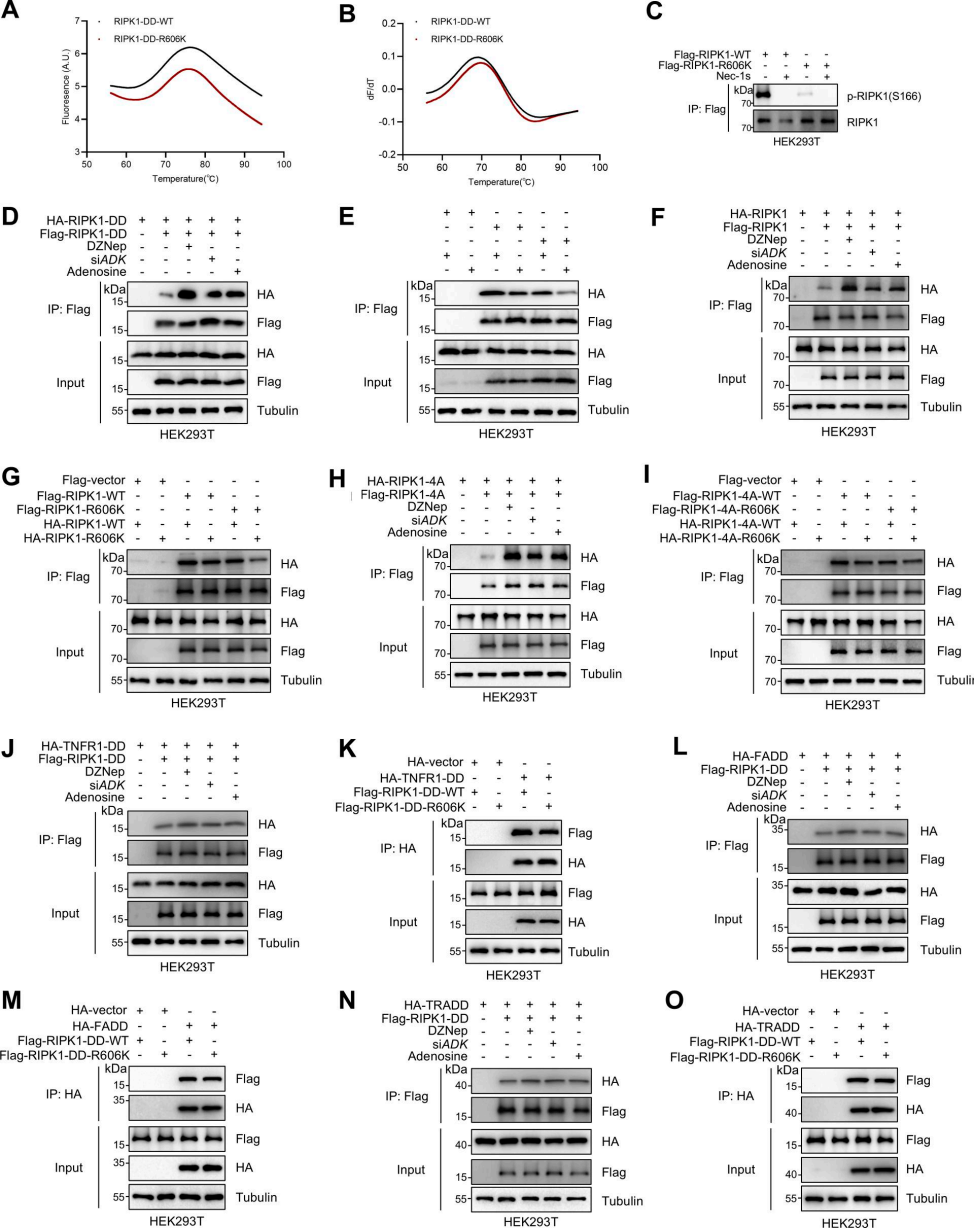
**RIPK1 R606me2s suppresses DD-mediated RIPK1 dimerization and the formation of complex I and complex II to inhibit subsequent cell death. (A and B)** Protein folding of RIPK1-DD-WT (50 µM) and RIPK1-DD-R606K (50 µM) was determined by the thermal shift assay. Data were collected in the presence of RIPK1-DD-WT or RIPK1-DD-R606K, leading to a rightward shift in the unfolding transition. The apparent melting temperature (Tm) is the peak in the derivative of the unfolding curve (dF/dT), which is used as an indicator of thermal stability. **(C)** HEK293T cells were transfected with expression vectors for Flag-RIPK1 WT or Flag-RIPK1-R606K in the presence or absence of Nec-1s (10 μM), respectively. The autophosphorylation of RIPK1 was determined with immunoblotting. **(D)** HEK293T cells were cotransfected with HA-tagged RIPK1-DD and Flag-tagged RIPK1-DD expression plasmids as indicated for 24 h. The cells were treated with DZNep (10 μM), si*ADK*, or adenosine (1 mM). The interaction between Flag-tagged and HA-tagged proteins was determined with immunoprecipitation and immunoblotting. DD, death domain. **(E)** HEK293T cells were cotransfected with Flag-tagged RIPK1-DD, Flag-tagged RIPK1-DD-R606K, HA-tagged RIPK1-DD, and HA-tagged RIPK1-DD-R606K expression plasmids as indicated for 24 h. The interaction between Flag-tagged and HA-tagged proteins was determined with immunoprecipitation and immunoblotting. DD, death domain. **(F)** HEK293T cells were cotransfected with HA-tagged RIPK1 and Flag-tagged RIPK1 expression plasmids as indicated for 24 h. The cells were treated with DZNep (10 μM), si*ADK*, or adenosine (1 mM). The interaction between Flag-tagged and HA-tagged proteins was determined with immunoprecipitation and immunoblotting. **(G)** HEK293T cells were cotransfected with Flag-tagged RIPK1, Flag-tagged RIPK1-R606K, HA-tagged RIPK1, and HA-tagged RIPK1-R606K expression plasmids as indicated for 24 h. The interaction between Flag-tagged and HA-tagged proteins was determined with immunoprecipitation and immunoblotting. **(H)** HEK293T cells were cotransfected with HA-tagged RIPK1-4A and Flag-tagged RIPK1-4A expression plasmids as indicated for 24 h. The cells were treated with DZNep (10 μM), si*ADK*, or adenosine (1 mM). The interaction between Flag-tagged and HA-tagged proteins was determined with immunoprecipitation and immunoblotting. 4A, amino acid sequence of IQIG in RIPK1 RHIM was mutated to AAAA. **(I)** HEK293T cells were cotransfected with Flag-tagged RIPK1-4A, Flag-tagged RIPK1-4A-R606K, HA-tagged RIPK1-4A, and HA-tagged RIPK1-4A-R606K expression plasmids as indicated for 24 h. The cells were lysed with 0.5% Nonidet P-40 buffer and divided equally into two parts. The interaction between Flag-tagged and HA-tagged proteins was determined with immunoprecipitation and immunoblotting. 4A, amino acid sequence of IQIG in RIPK1 RHIM was mutated to AAAA. **(J)** HEK293T cells were cotransfected with Flag-RIPK1 expression plasmids along with HA-tagged TNFR1-DD expression plasmids for 24 h. The cells were treated with DZNep (10 μM), si*ADK*, or adenosine (1 mM). The interaction between Flag-tagged and HA-tagged proteins was determined with immunoprecipitation and immunoblotting. **(K)** HEK293T cells were cotransfected with Flag-RIPK1-WT or Flag-RIPK1-R606K expression plasmids, as well as HA-tagged TNFR1-DD expression plasmids for 24 h. The interaction between Flag-tagged and HA-tagged proteins was determined with immunoprecipitation and immunoblotting. **(L)** HEK293T cells were cotransfected with Flag-RIPK1 expression plasmids along with HA-tagged FADD expression plasmids for 24 h. The cells were treated with DZNep (10 μM), si*ADK*, or adenosine (1 mM). The interaction between Flag-tagged and HA-tagged proteins was determined with immunoprecipitation and immunoblotting. **(M)** HEK293T cells were cotransfected with Flag-RIPK1-WT or Flag-RIPK1-R606K expression plasmids, as well as HA-tagged FADD expression plasmids for 24 h. The interaction between Flag-tagged and HA-tagged proteins was determined with immunoprecipitation and immunoblotting. **(N)** HEK293T cells were cotransfected with Flag-RIPK1 expression plasmids along with HA-tagged TRADD expression plasmids for 24 h. The cells were treated with DZNep (10 μM), si*ADK*, or adenosine (1 mM). The interaction between Flag-tagged and HA-tagged proteins was determined with immunoprecipitation and immunoblotting. **(O)** HEK293T cells were cotransfected with Flag-RIPK1-WT or Flag-RIPK1-R606K expression plasmids, as well as HA-tagged TRADD expression plasmids for 24 h. The interaction between Flag-tagged and HA-tagged proteins was determined with immunoprecipitation and immunoblotting. Data are representative of *n* = 3 independent experiments (A–O). Source data are available for this figure: SourceData F4.

We then investigated the mechanism underlying R606me2s inhibits RIPK1 activation. RIPK1 consists of an N-terminal kinase domain (KD), an intermediate domain (ID) that contains a RIP homotypic interaction motif (RHIM), and a C-terminal DD (Stanger et al., 1995). DD is a conserved domain shared by proteins including TNFR1, TRADD, RIPK1, and FADD, and mediates homotypic interactions among these proteins to promote cell death signaling (Park et al., 2007). As R606 locates in the DD of RIPK1, we first analyzed whether R606me2s affects the protein structure of RIPK1 DD. As revealed by thermal stability profiles, the melting temperatures of DD-WT and DD-R606K were similar (Fig. 4, A and B), which rules out that R606 methylation may significantly affect RIPK1 protein folding.

Homotypic interaction between DDs principally relies on electrostatic interaction mediated by specific charged residues; in contrast, hydrophobic interaction contributes little to DD interaction (Telliez et al., 2000; Jeong et al., 1999; Meng et al., 2018). Since symmetric dimethylation of arginine reduces the ability of its guanidine group to form hydrogen bond and increases its bulkiness and hydrophobicity (Yang and Bedford, 2013; Wu et al., 2021), we tested whether R606me2s suppresses DD-mediated dimerization of RIPK1, which is indispensable for RIPK1 activation (Meng et al., 2018). We found that R606K mutation inhibited RIPK1 autophosphorylation induced by RIPK1 overexpression (Fig. 4 C). The interaction between exogenously expressed Flag-tagged and HA-tagged DD of RIPK1 was promoted by DZNep, ADK knockdown, or adenosine treatment (Fig. 4 D), but inhibited by R606K mutation (Fig. 4 E), suggesting that R606 is an important positively charged residue that facilitates dimerization of RIPK1-DD and that either methylation or mutation of R606 suppresses this function. Consistently, the dimerization of full-length RIPK1 was also enhanced by methylation inhibition and suppressed by R606K mutation (Fig. 4, F and G). As homointeraction of both RHIM and DD might contribute to dimerization of full-length RIPK1, we also evaluated interaction between RIPK1 whose RHIM was disrupted by mutation of core IQIG to AAAA (4A) (Meng et al., 2018). We found that the dimerization of RIPK1 4A mutants was similarly facilitated by methylation inhibition and suppressed by R606K mutation (Fig. 4, H and I), suggesting that DD, but not RHIM, is the main methylated domain that promotes RIPK1 dimerization.

In response to TNFα, RIPK1 is recruited to TNFR1 by binding to its intracellular DD; after activation in a dimer, RIPK1 is released from complex I and binds to FADD via DD-mediated interaction and forms complex II that promotes apoptosis or necroptosis depending on whether caspase-8 is disabled (Meng et al., 2018; Laurien et al., 2020). Thus, besides dimerization of two RIPK1 proteins, DD-mediated heteroassociation between RIPK1 and other DD-containing proteins, especially TNFR1 and FADD, might also regulate TNFα-induced cell death. However, in overexpression experiments with HEK293T cells, blocking methylation or R606K mutation did not substantially regulate the interaction between RIPK1 DD and TNFR1 DD, FADD, or TRADD (Fig. 4, J–O).

**Figure 5.**
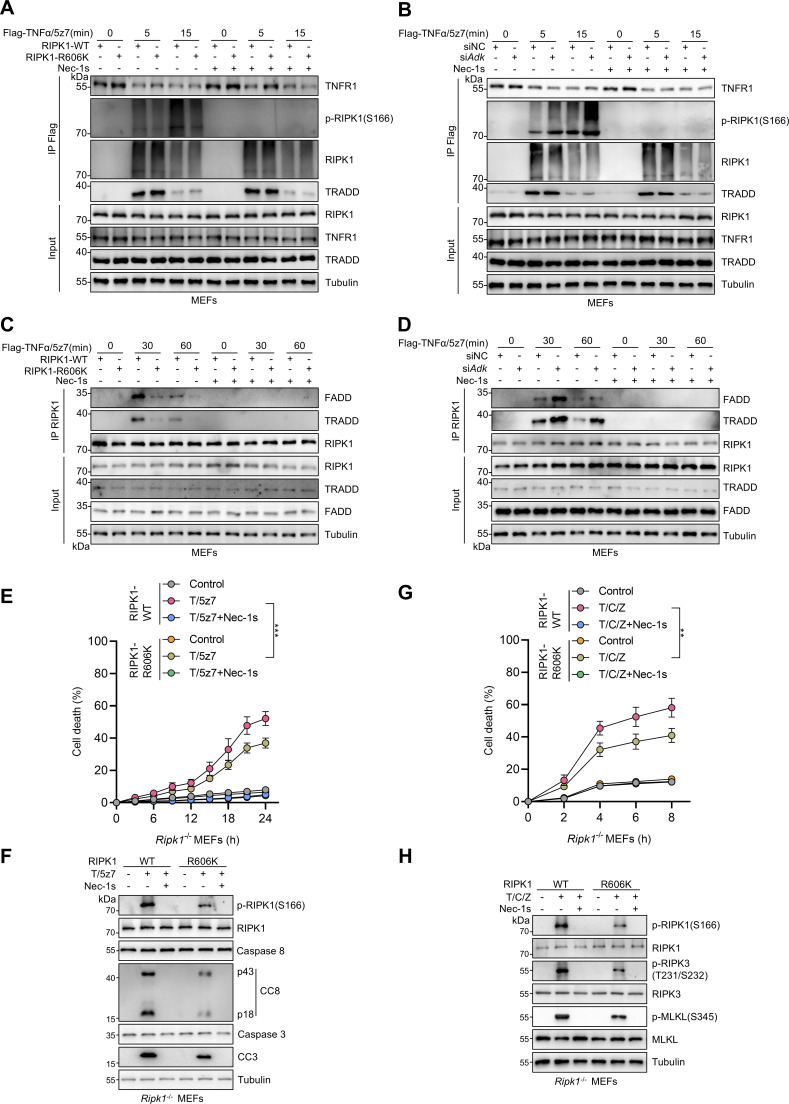
**RIPK1 R606me2s suppresses RIPK1 kinase activation and the formation of complex I and complex II to inhibit subsequent cell death. (A and B)** MEFs were reconstituted with WT or R606K mutant RIPK1 (A) or treated with negative control siRNAs or siRNAs targeting ADK (B). The cells were stimulated by Flag-TNFα (100 ng/ml) after pretreatment of 5z7 (500 nM) for 30 min. After stimulation for indicated periods, complex I was immunoprecipitated with Flag antibody. Relative levels of TNFR1, p-RIPK1 (S166), RIPK1, TRADD in complex I or whole-cell lysates were measured with immunoblotting. **(C and D)** MEFs were reconstituted with WT or R606K mutant RIPK1 (C) or treated with negative control siRNAs or siRNAs targeting ADK (D). The cells were stimulated by Flag-TNFα (10 ng/ml) after pretreatment of 5z7 (500 nM) for 30 min. After stimulation for indicated periods, RIPK1 was immunoprecipitated. Relative levels of RIPK1, FADD, TRADD in RIPK1 immunocomplex or whole-cell lysates were measured with immunoblotting. **(E and F)***Ripk1*^−/−^ MEFs were reconstituted with WT or R606K mutant Flag-RIPK1 by lentivirus. The cells were subsequently treated with 5z7 (500 nM)/TNFα (10 ng/ml) for the indicated time (E) or 12 h (F) in the presence or absence of Nec-1s (10 μM). Cell death was measured by the SYTOX Green positivity assay (E). The levels of p-RIPK1(S166), CC8, and CC3 were determined by immunoblotting (F). **(G and H)***Ripk1*^−/−^ MEFs were reconstituted with WT or R606K mutant Flag-RIPK1 by lentivirus. The cells were subsequently treated with CHX (C, 2 μg/ml) and zVAD.fmk (Z, 10 μM) for 0.5 h followed by 10 ng/ml TNFα (T) for the indicated time in the presence or absence of Nec-1s (10 μM). Cell death was measured by the SYTOX Green positivity assay (G). The levels of p-S166 RIPK1, p-T231/S232 RIPK3, and p-S345 MLKL were determined by immunoblotting (H). Data are represented as the mean ± SD (E and G). Data are representative of *n* = 3 independent experiments (A–H). Statistical significance was determined using two-way ANOVA with post hoc Bonferroni’s test (E and G). **P < 0.01; ***P < 0.001. Source data are available for this figure: SourceData F5.

We then tested the effects of RIPK1 R606me2s on endogenous RIPK1 kinase activation and its binding to TNFR1, FADD, and TRADD in MEFs. We stimulated RIPK1 KO MEFs reconstituted with WT or R606K mutant RIPK1 with or without Flag-TNFα/5z7, and immunoprecipitated complex I to examine the binding of RIPK1 to TNFR1. Interaction of RIPK1 with TNFR1 was similar in WT or R606K RIPK1-reconstituted MEFs; however, R606K mutation suppressed RIPK1 kinase activation within complex I (Fig. 5 A). In WT MEFs, knockdown of ADK had no effect on the recruitment of RIPK1 to TNFR1 but also promoted RIPK1 kinase activation in complex I (Fig. 5 B). In RIPK1 kinase–mediated apoptosis, RIPK1 kinase activity is essential for its binding with FADD or TRADD (Laurien et al., 2020). Consistently, in TNFα/5z7-induced RDA, the interaction between RIPK1 and TRADD or FADD was abrogated by RIPK1 kinase blockade using the specific RIPK1 kinase inhibitor Nec-1s (Fig. 5, C and D). Given that R606 methylation suppresses RIPK1 kinase activation, we observed that the interaction between RIPK1 and TRADD or FADD was suppressed upon R606K mutation but enhanced upon ADK knockdown (Fig. 5, C and D). However, in the overexpression system, we showed that R606K mutation had a minor effect on the interaction of RIPK1-DD with FADD or TRADD (Fig. 4, J–O), suggesting that RIPK1 R606me2s specifically inhibits RIPK1 dimerization and activation without significantly affecting its binding with other DD-containing proteins.

We also tested whether RIPK1 R606 plays a key role in RDA and necroptosis. When reconstituted MEFs were stimulated by TNFα/5z7 or TNFα/CHX/zVAD, compared with WT RIPK1, R606K mutation decreased RIPK1 activation and subsequent apoptosis or necroptosis (Fig. 5, E–H). Thus, ADK licenses RIPK1 R606me2s to suppress RIPK1 dimerization and kinase activation, which keeps TNFα-induced cell death in check.

### RIPK1 R606me2s is catalyzed by protein arginine methyltransferase 5

**Figure 6.**
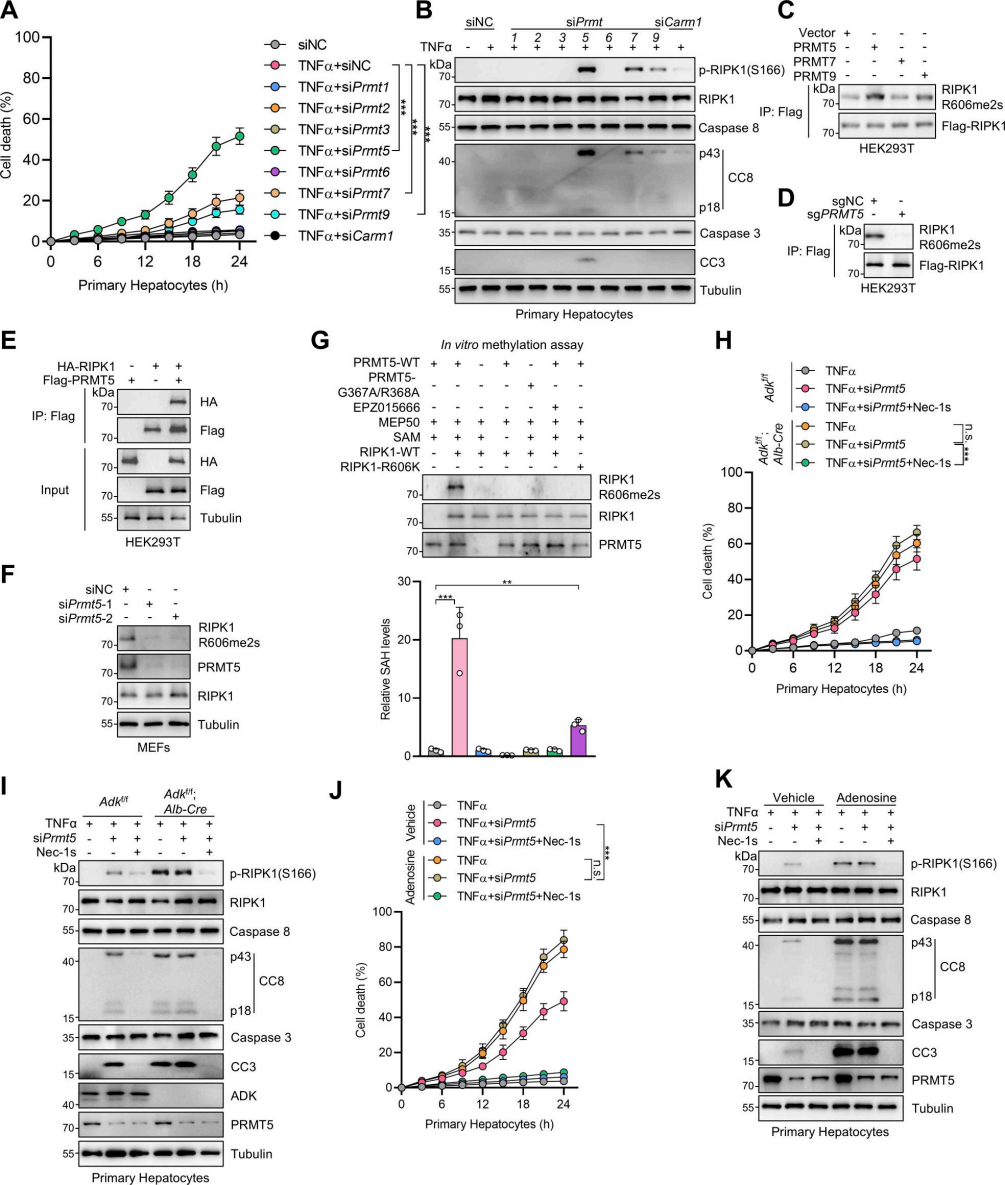
**RIPK1 R606 symmetric dimethylation is catalyzed by arginine methyltransferase PRMT5. (A and B)** Primary hepatocytes were transfected with siNC or siRNAs targeting PRMTs. The cells were treated with TNFα (10 ng/ml) for the indicated time (A) or 12 h (B). Cell death was measured by the SYTOX Green positivity assay (A). The levels of p-RIPK1(S166), CC8, and CC3 were determined by immunoblotting (B). **(C)** HEK293T cells were cotransfected with expression vectors for Flag-RIPK1, as well as PRMT5, PRMT7, or PRMT9. Flag-RIPK1 was immunoprecipitated using anti-Flag antibody, and R606me2s level was determined by immunoblotting. **(D)** HEK293T cells were transfected with expression vectors for Flag-RIPK1. Endogenous PRMT5 was deleted with the CRISPR/Cas9 system. Flag-RIPK1 was immunoprecipitated using anti-Flag antibody, and R606me2s level was determined by immunoblotting. **(E)** HEK293T cells were cotransfected with expression vectors for HA-RIPK1 and Flag-PRMT5. The interaction between HA-RIPK1 and Flag-PRMT5 was analyzed with immunoprecipitation and immunoblotting. **(F)** MEFs transfected with siNC or si*Prmt5* were treated with TNFα (10 ng/ml) for 15 min. The levels of RIPK1 R606me2s and PRMT5 were determined by immunoblotting. **(G)** WT or R606K mutant RIPK1 was purified from *PRMT5*^−/−^ HEK293T cells and was subjected to in vitro methylation assays for 1 h in the presence or absence of WT or G367A/R368A mutant PRMT5, EPZ015666, MEP50, SAM. The levels of RIPK1 R606me2s, PRMT5, and RIPK1 were determined by immunoblotting. The generation of SAH was analyzed via the MTase-Glo methyltransferase assay kit. **(H and I)** Primary hepatocytes from *Adk*^f/f^ or *Adk*^f/f^;*Alb-Cre* mice were treated with TNFα (10 ng/ml) for the indicated time (H) or 12 h (I) with or without si*Prmt5* in the presence or absence of Nec-1s (10 μM). Cell death was measured by the SYTOX Green positivity assay (H). The levels of p-RIPK1(S166), CC8, CC3, ADK, and PRMT5 were determined by immunoblotting (I). **(J and K)** Primary hepatocytes were treated with TNFα (10 ng/ml) for the indicated time (J) or 12 h (K) with or without si*Prmt5* in the presence or absence of adenosine (1 mM) or Nec-1s (10 μM). Cell death was measured by the SYTOX Green positivity assay (J). The levels of p-RIPK1(S166), CC8, CC3, and PRMT5 were determined by immunoblotting (K). Data are represented as the mean ± SD (A, G, H, and J). Data are representative of *n* = 3 independent experiments (A–K). Statistical significance was determined using two-way ANOVA with post hoc Bonferroni’s test (A, H, and J) or one-way ANOVA with post hoc Dunnett’s test (G). **P < 0.01; ***P < 0.001. Source data are available for this figure: SourceData F6.

**Figure S3.**
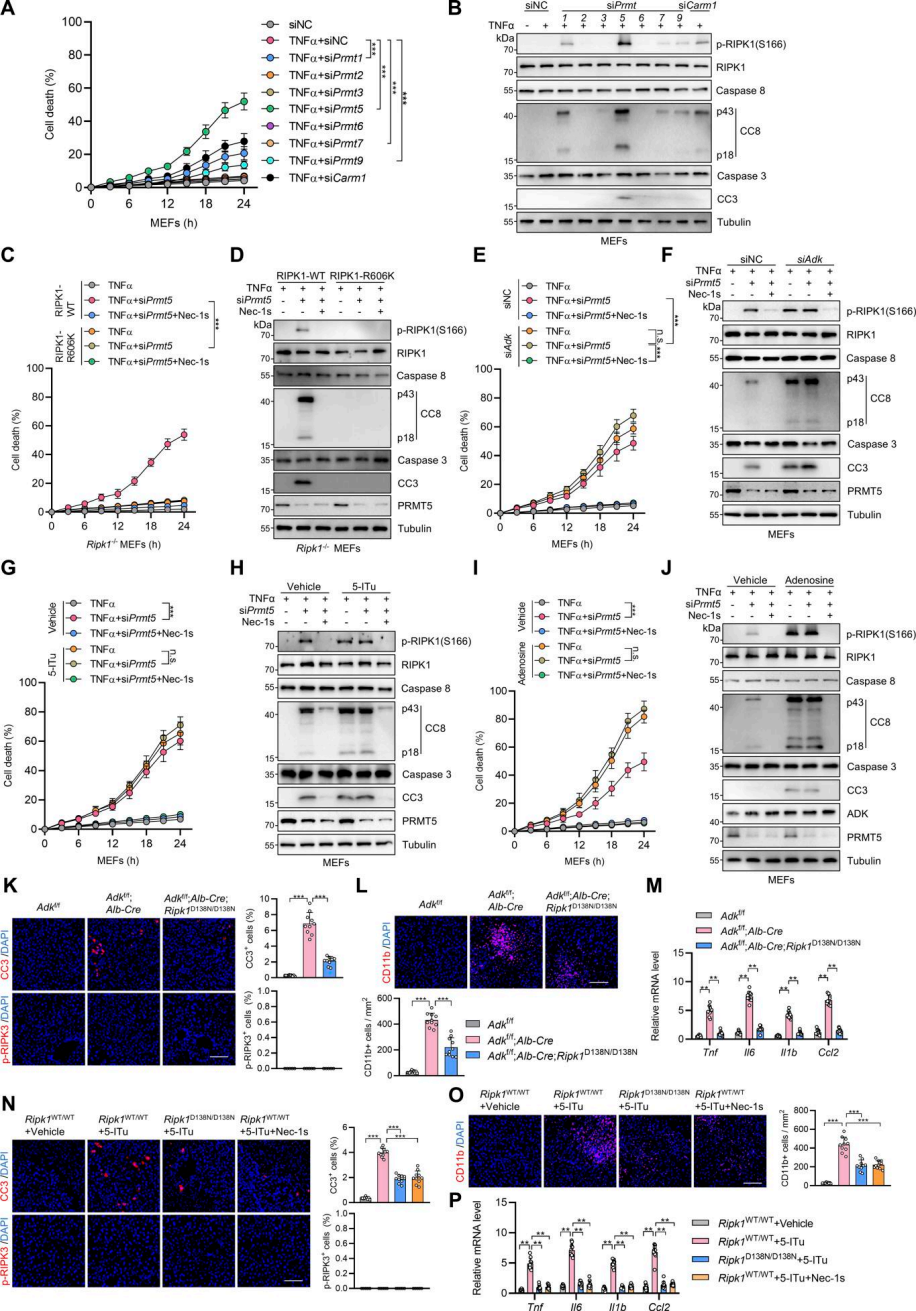
**Symmetric dimethylation of RIPK1 R606 is catalyzed by the arginine methyltransferase PRMT5. (A and B)** MEFs were transfected with siNC or siRNAs targeting PRMTs. The cells were treated with TNFα (10 ng/ml) for the indicated time (A) or 12 h (B). Cell death was measured by the SYTOX Green positivity assay (A). The levels of p-RIPK1(S166), CC8, and CC3 were determined by immunoblotting (B). **(C and D)***Ripk1*^−/−^ MEFs were reconstituted with WT or R606K mutant Flag-RIPK1 by lentivirus. The cells were transfected with si*Prmt5* and were subsequently treated with TNFα (10 ng/ml) for the indicated time (C) or 12 h (D) in the presence or absence of Nec-1s (10 μM). Cell death was measured by the SYTOX Green positivity assay (C). The levels of p-RIPK1(S166), CC8, CC3, and PRMT5 were determined by immunoblotting (D). **(E and F)** MEFs transfected with siNC or si*Adk* or si*Prmt5* were treated with TNFα (10 ng/ml) for the indicated time (E) or 12 h (F) in the presence or absence of Nec-1s (10 μM). Cell death was measured by the SYTOX Green positivity assay (E). The levels of p-RIPK1(S166), CC8, CC3, and PRMT5 were determined by immunoblotting (F). **(G and H)** MEFs transfected with siNC or si*Prmt5* were treated with TNFα (10 ng/ml) for the indicated time (G) or 12 h (H) in the presence or absence of 5-ITu (20 μM) or Nec-1s (10 μM). Cell death was measured by the SYTOX Green positivity assay (G). The levels of p-RIPK1(S166), CC8, CC3, and PRMT5 were determined by immunoblotting (H). **(I and J)** MEFs transfected with siNC or si*Prmt5* were treated with TNFα (10 ng/ml) for the indicated time (I) or 12 h (J) in the presence or absence of adenosine (1 mM) or Nec-1s (10 μM). Cell death was measured by the SYTOX Green positivity assay (I). The levels of p-RIPK1(S166), CC8, CC3, ADK, and PRMT5 were determined by immunoblotting (J). **(K–M)***Adk*^f/f^, *Adk*^f/f^;*Alb-Cre*, or *Adk*^f/f^;*Alb-Cre*;*Ripk1*^D138N/D138N^ mice were analyzed. In the unstressed conditions at the age of 7 wk, liver CC3 and p-RIPK3 immunostaining (K), and CD11b immunostaining (L) were conducted; mRNA levels of *Tnf*, *Il6*, *Il1β*, and *Ccl2* (M) were analyzed. Scale bar: 100 μm (K and L). **(N–P)** Vehicle, 5-ITu (100 mg/kg), and Nec-1s (20 mg/kg) were intraperitoneally injected to WT or *Ripk1*^D138N/D138N^ mice every day. 72 h after the first injection, liver CC3 and p-RIPK3 immunostaining (N) and CD11b immunostaining (O) were conducted; mRNA levels of *Tnf*, *Il6*, *Il1β*, and *Ccl2* (P) were analyzed. Scale bar: 100 μm (N and O). Data are represented as the mean ± SD (A, C, E, G, I, and K–P). Data are representative of *n* = 3 independent experiments (A–P). Statistical significance was determined using two-way ANOVA with post hoc Bonferroni’s test (A, C, E, G, I, M, and P) or one-way ANOVA with post hoc Dunnett’s test (K, L, N, and O). **P < 0.01; ***P < 0.001. Source data are available for this figure: SourceData FS3.

We then investigated the enzyme that catalyzes the formation of RIPK1 R606me2s. In mammals, arginine methylation is catalyzed by protein arginine methyltransferase (PRMT) family, which consists of nine members (Zheng et al., 2023). Since RIPK1 R606 methylation inhibits TNFα-induced apoptosis, the corresponding PRMT should also regulate RIPK1-driven cell death. Among the PRMT family, the expression of PRMT8 is limited in brain while the rest are expressed by a wide range of tissues (Bedford and Clarke, 2009). Therefore, we knocked down PRMTs, except for PRMT8, and stimulated the cells with TNFα to screen for PRMTs that regulate TNFα-induced cell death. We found that silencing PRMT1, PRMT5, PRMT7, PRMT9, and CARM1 (PRMT4) promotes RIPK1 activation and apoptosis in response to TNFα with PRMT5 exhibiting the most significant effect; in contrast, knocking down the rest PRMTs did not affect the TNFα pathway (Fig. 6, A and B; and Fig. S3, A and B). Among the identified PRMTs, PRMT5, PRMT7, and PRMT9 are able to catalyze the formation of SDMA (Bedford, 2007). We then screened for the PRMT that catalyzes RIPK1 R606me2s by ectopic expression in HEK293T cells. The overexpression of PRMT5 most significantly increased RIPK1 R606me2s (Fig. 6 C). Furthermore, knockout of *PRMT5* decreased R606me2s of exogenously expressed RIPK1 (Fig. 6 D). Consistently, exogenous PRMT5 interacted with exogenous RIPK1 (Fig. 6 E).

To confirm that PRMT5 is the endogenous PRMT for RIPK1, we knocked down PRMT5 in MEFs and found that silencing PRMT5 reduced RIPK1 R606me2s (Fig. 6 F). We then conducted in vitro methylation experiments to consolidate the function of PRMT5 to methylate RIPK1. From both immunoblotting and SAH production measurement, we confirmed that PRMT5 symmetrically dimethylated RIPK1 at R606, which was prevented by PRMT5 catalytic dead mutation or PRMT5 inhibitor EPZ015666 (Kryukov et al., 2016) (Fig. 6 G). Interestingly, although PRMT5 was unable to methylate R606K mutant, SAH production still occurred, indicating that PRMT5 methylates RIPK1 at additional residues (Fig. 6 G). RIPK1 R606K mutation abrogated the cell death sensitized by PRMT5 deficiency, suggesting that PRMT5 suppresses RIPK1-induced cell death by catalyzing R606me2s (Fig. S3, C and D).

We then tested whether PRMT5 is indispensable for adenosine metabolism to regulate TNFα-induced cell death. Consistent with the notion that ADK removes adenosine to license PRMT5-mediated RIPK1 R606 methylation, PRMT5 deficiency failed to promote TNFα-induced RIPK1 activation and apoptosis in the conditions of ADK knockdown, ADK inhibition, or adenosine treatment (Fig. 6, H–K and Fig. S3, E–J). Notably, in cells stimulated with TNFα, adenosine accumulation induced more RIPK1 activation and cell death than PRMT5 deficiency (Fig. 6, H–K and Fig. S3, E–J), which suggests that adenosine metabolism regulates TNFα-induced cell death in a manner that is largely but not completely dependent on PRMT5-mediated arginine methylation.

### ADK deficiency results in spontaneous RIPK1-driven liver damage

**Figure 7.**
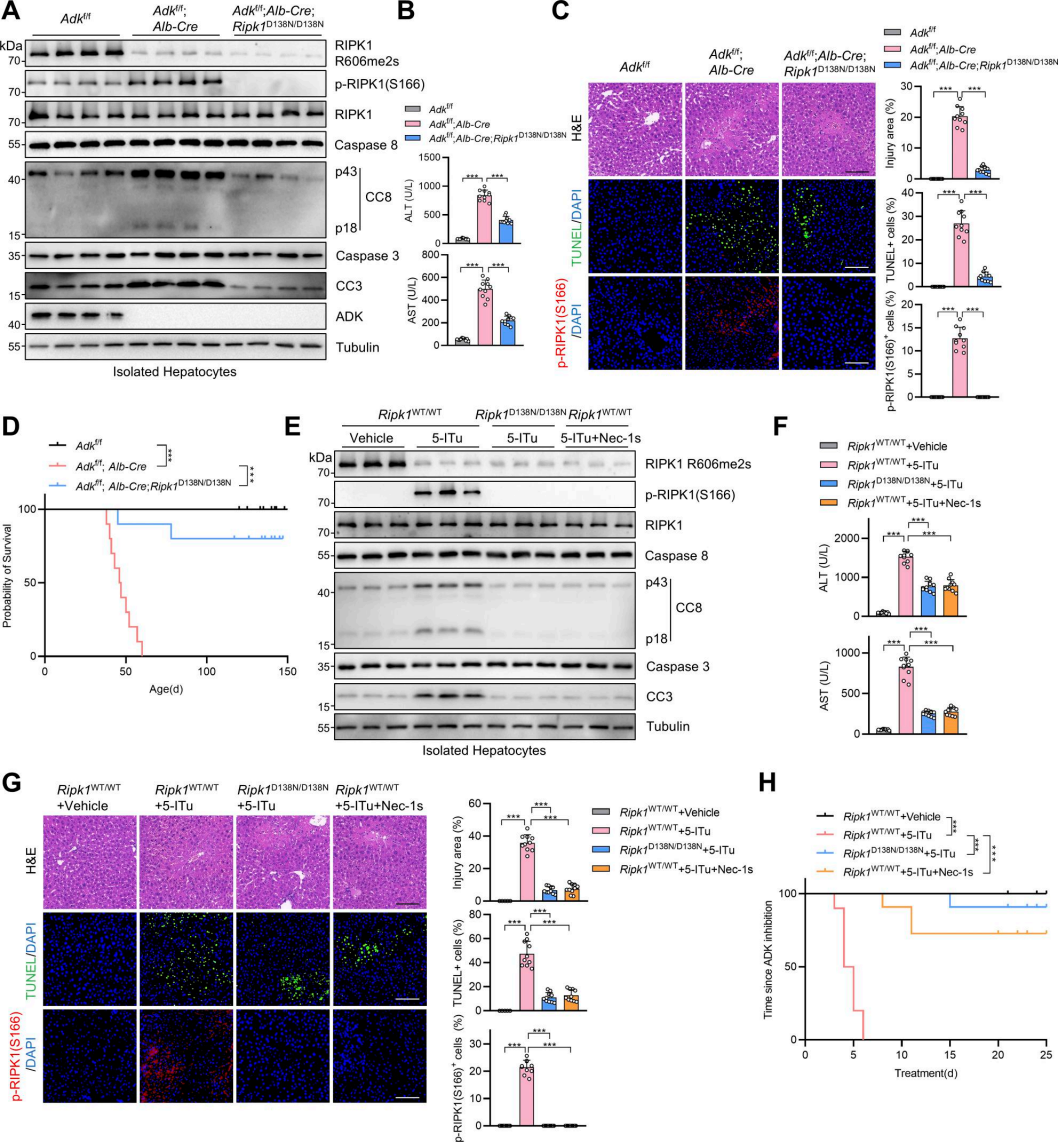
**ADK deficiency results in spontaneous RIPK1-driven liver damage. (A–D)** *Adk* ^f/f^, *Adk*^f/f^;*Alb-Cre*, or *Adk*^f/f^;*Alb-Cre*;*Ripk1*^D138N/D138N^ mice were analyzed. In the unstressed conditions at the age of 7 wk, the levels of p-RIPK1(S166), CC8, CC3, and ADK in hepatocytes isolated from the livers were determined by immunoblotting (A); liver H&E staining, TUNEL staining, and p-RIPK1(S166) immunostaining were performed (B); serum ALT/AST detection (C) was performed. Scale bar: 100 μm. The survival time of mice of different genotypes was analyzed (D). **(E–H)** Vehicle, 5-ITu (100 mg/kg), and Nec-1s (20 mg/kg) were intraperitoneally injected to 8-wk-old *Ripk1*^WT/WT^ or *Ripk1*^D138N/D138N^ mice every day. 72 h after the first injection, the levels of p-RIPK1(S166), CC8, CC3, and ADK in hepatocytes isolated from the livers were determined by immunoblotting (E); serum ALT/AST detection was performed (F); liver H&E staining, TUNEL staining, and p-RIPK1(S166) immunostaining were performed (G). Scale bar: 100 μm. The survival time of mice of different treatments was analyzed (H). Data are represented as the mean ± SD (B, C, F, and G). Data are representative of *n* = 3 independent experiments (A–H). Statistical significance was determined using one-way ANOVA with post hoc Dunnett’s test (B, C, F, and G) or two-sided log-rank test (D and H). ***P < 0.001. Source data are available for this figure: SourceData F7.

Keeping RIPK1 activity in check is essential for preventing hepatic tissue homeostasis disruption both during embryonic development and after birth (Xu et al., 2018; Zhang et al., 2023a). Thus, we subsequently characterized the physiologic role of ADK in suppressing RIPK1 and cell death. In support of the notion that ADK suppresses RIPK1 activation, we detected activated RIPK1 in livers of ADK knockout mice, which was blocked by RIPK1 kinase-dead mutation in *Adk*^f/f^;*Alb-Cre*;*Ripk1*^D138N/D138N^ mice (Fig. 7 A). In line with in vitro data, hepatic RIPK1 R606me2s was reduced upon ADK knockout but was unaffected by RIPK1 kinase activity (Fig. 7 A). Consequently, as revealed by serum alanine aminotransferase (ALT)/ aspartate aminotransferase (AST) detection (Fig. 7 B) and H&E staining and TdT-mediated dUTP Nick-End Labeling (TUNEL) assay, hepatocyte-specific ADK knockout led to spontaneous liver injury in unstressed conditions (Fig. 7 C). Of note, RIPK1 inactivation reversed hepatic cell death, as well as serum ALT and AST upregulation in ADK knockout mice, indicative of alleviated liver injury (Fig. 7, A–C). Interestingly, ADK knockout–induced cell death can be attributed to apoptosis as marked by CC3-positive signals, while necroptosis marker p-S231/S232 RIPK3 was absent in injured liver (Fig. S3 K), which is consistent with the silenced expression of RIPK3 in liver (Preston et al., 2022; Zhang et al., 2023b). Furthermore, more proinflammatory myeloid cells (CD11b^+^) infiltrated into livers of ADK knockout mice compared with WT mice, which was prevented by RIPK1 D138N mutation (Fig. S3 L). ADK deficiency also enhanced hepatic expression of multiple proinflammatory cytokines including *Tnf*, *Il6*, *Il1b*, and *Ccl2*, which was reversed by RIPK1 kinase inactivation (Fig. S3 M). Importantly, all of *Adk*^f/f^;*Alb-Cre* mice died during 38–60 days of age; in contrast, majority of *Adk*^f/f^;*Alb-Cre*;*Ripk1*^D138N/D138N^ mice survived over 120 days (Fig. 7 D).

ADK inhibition is proposed to treat multiple diseases by boosting endogenous adenosine levels; however, this therapeutic strategy is impeded by drug-induced liver injury of unknown reasons (Boison, 2013). We injected a large dose of 5-ITu into mice intraperitoneally to mimic the therapeutic regimen. We found that 5-ITu administration decreased hepatic RIPK1 R606me2s levels and promoted RIPK1 kinase activation and apoptosis (Fig. 7, E–G and Fig. S3, N–P). As a result, all of WT mice died within 1 wk after ADK inhibition (Fig. 7 H). Strikingly, due to RIPK1 kinase blockade, *Ripk1*^D138N/D138N^ mice, as well as *Ripk1*^WT/WT^ mice that were simultaneously injected with Nec-1s, exhibited mitigated liver damage (Fig. 7, E–G and Fig. S3, N–P), which enabled majority of them to survive >3 wk (Fig. 7 H). Thus, ADK maintains hepatic tissue homeostasis by licensing RIPK1 R606me2s and preventing RIPK1 kinase–driven liver injury in physiological conditions.

### ADK is reduced in liver ischemia–reperfusion and aggravates liver injury

**Figure 8.**
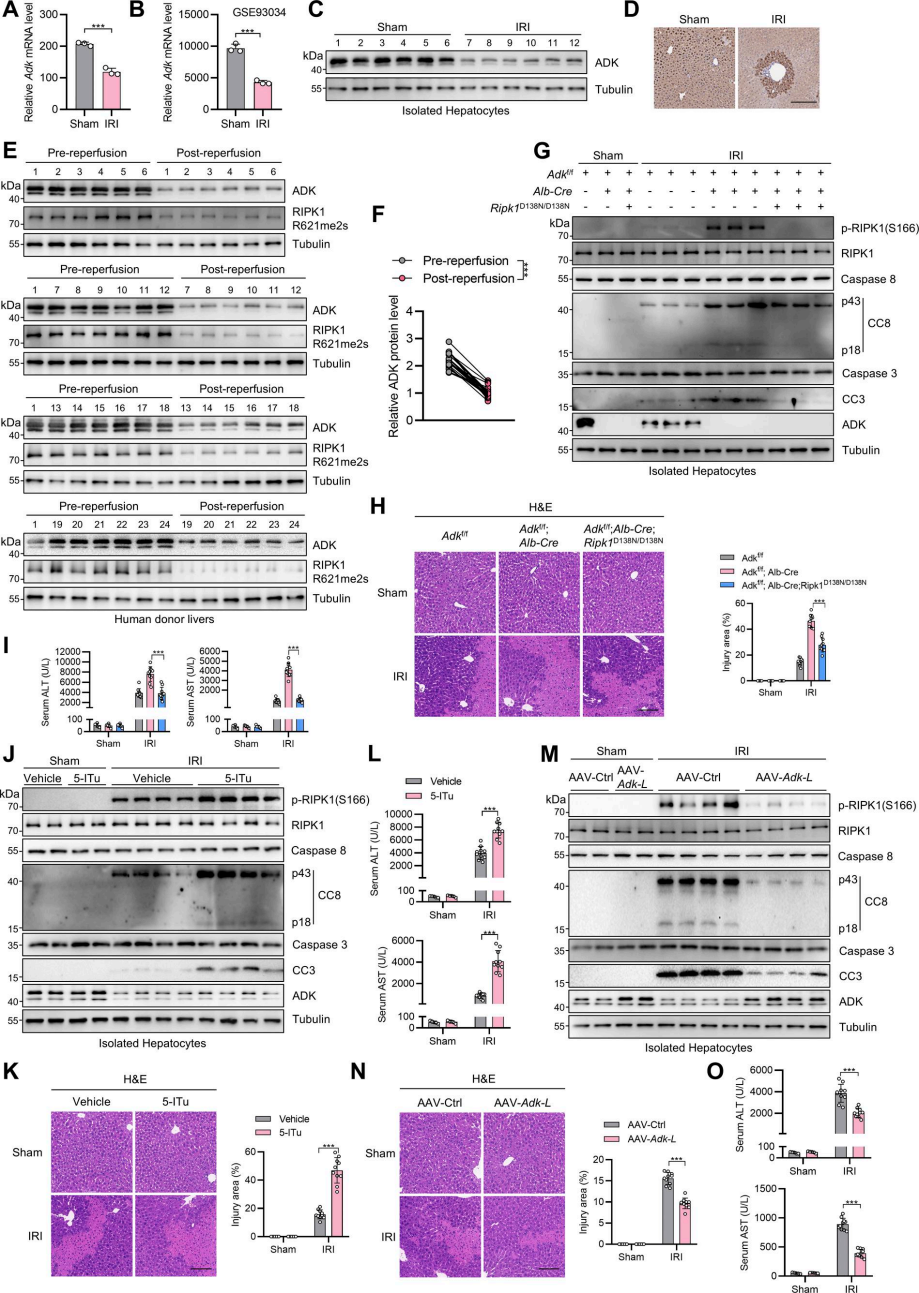
**ADK expression reduction promotes liver IRI. (A)** RNA-seq revealed the mRNA level of ADK after IRI in mouse liver tissue. **(B)** ADK mRNA level in mouse liver IRI transcriptome dataset GSE93034 was shown. **(C and D)** Protein levels of ADK after IRI in mouse livers were detected by immunoblotting and immunohistochemistry. Scale bar: 200 μm (D). **(E and F)** ADK protein levels were detected in donor livers before reperfusion or after reperfusion by immunoblotting (E). Comparison of protein expression levels of ADK before and after reperfusion (F). *n* = 24 for donor livers (F). **(G–I)***Adk*^f/f^, *Adk*^f/f^;*Alb-Cre*, or *Adk*^f/f^;*Alb-Cre*;*Ripk1*^D138N/D138N^ mice underwent 1-h ischemia/6-h reperfusion operation at the age of 5 wk. The levels of p-RIPK1(S166), CC8, CC3, and ADK in isolated hepatocytes were determined by immunoblotting (G). Liver H&E staining (H) and serum ALT/AST detection (I) were performed. Scale bar: 100 μm (H). **(J–L)** Vehicle or 5-ITu (20 mg/kg) was injected intraperitoneally to WT mice at the age of 8 wk. 1 h after the injection, the mice underwent 1-h ischemia/6-h reperfusion operation. The levels of p-RIPK1(S166), CC8, CC3, and ADK were determined by immunoblotting (J). Liver H&E staining (K) and serum ALT/AST detection (L) were performed. Scale bar: 100 μm (K). **(M–O)** AAV8 that was expressing *Adk-L* or control was injected to WT mice through tail vein. 1 mo after the injection, the mice underwent 1-h ischemia/6-h reperfusion operation. The levels of p-RIPK1(S166), CC8, CC3, and ADK were determined by immunoblotting (M). Liver H&E staining (N) and serum ALT/AST detection (O) were performed. Scale bar: 100 μm (N). Data are represented as the mean ± SD (A, B, H, I, K, L, N, and O). Data are representative of *n* = 3 independent experiments (A–O). Statistical significance was determined using unpaired two-tailed Student’s *t* test (A and B), paired two-tailed Student’s t test (F), or two-way ANOVA with post hoc Bonferroni’s test (H, J, K, L, N, and O). ***P < 0.001. Source data are available for this figure: SourceData F8.

We then assessed whether ADK-mediated RIPK1 kinase suppression is implicated in human diseases. In RNA-sequencing (RNA-seq) data derived from mouse livers that experienced IRI, an inevitable pathology during clinical liver partial resection or transplantation operation that can be life-threatening (Hirao et al., 2022), we found that ADK expression decreased sharply in IRI-challenged livers compared with sham-operated livers (Fig. 8 A). This observation was consistent with a public dataset (Kleinschmidt et al., 2017) in the Gene Expression Omnibus (Fig. 8 B), and was validated in our experimental system (Fig. 8, C and D). Consistently, hepatic ADK expression was also reduced in clinical donor livers after reperfusion compared with that before reperfusion during human liver transplantation surgery (Fig. 8, E and F).

**Figure S4.**
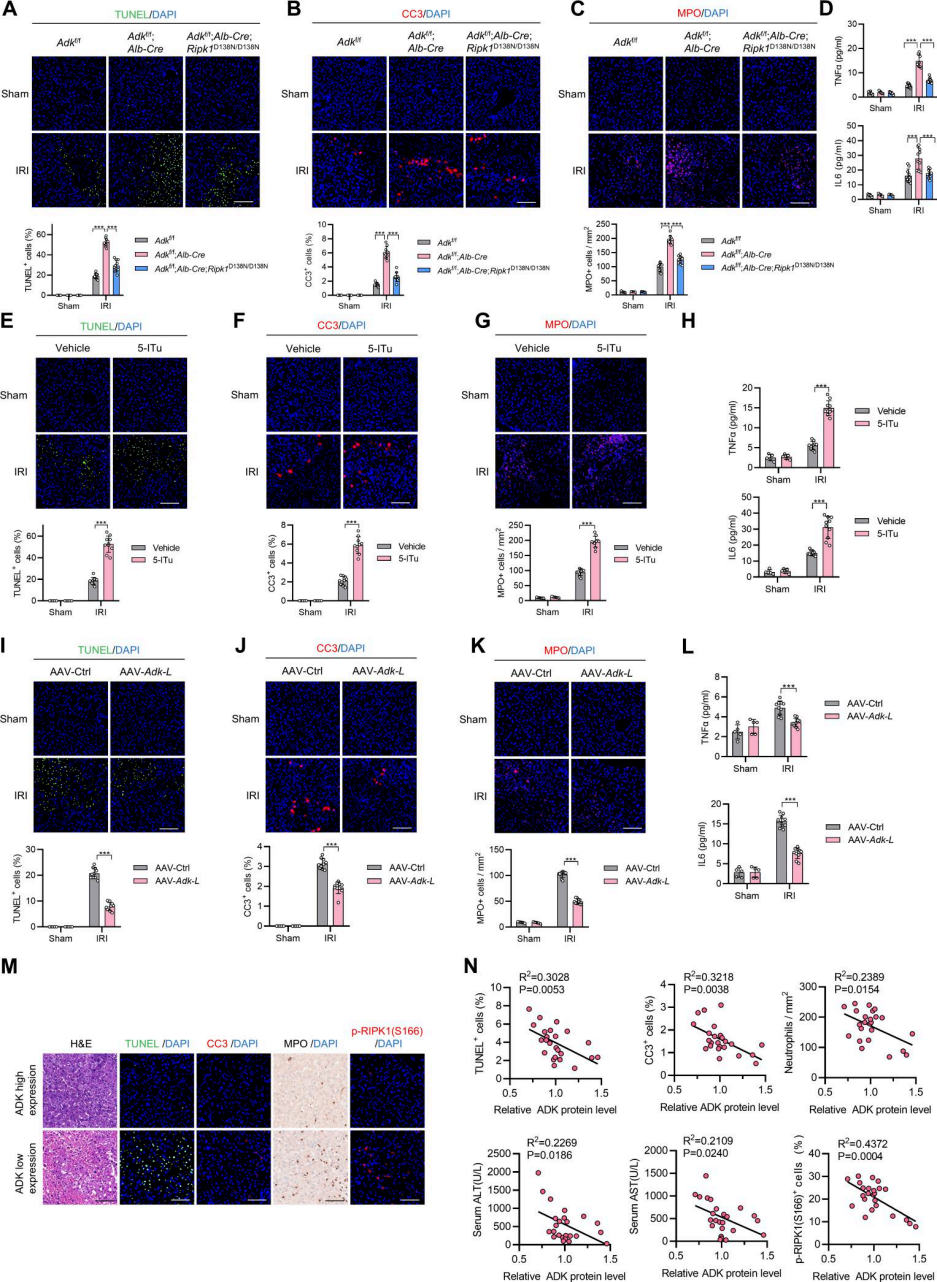
**ADK maintains tissue homeostasis by preventing RIPK1 kinase activation–driven liver injury in physiological conditions and IRI. (A–D)** *Adk* ^f/f^, *Adk*^f/f^;*Alb-Cre*, or *Adk*^f/f^;*Alb-Cre*;*Ripk1*^D138N/D138N^ mice underwent 1-h ischemia/6-h reperfusion operation at the age of 6 wk. Liver TUNEL staining (A), CC3 immunostaining (B), and MPO immunostaining (C) were performed. Serum levels of proinflammatory cytokines TNFα and IL-6 were detected with ELISA (D). Scale bar: 100 μm (A–C). **(E–H)** Vehicle or 5-ITu (20 mg/kg) was injected intraperitoneally to WT mice at the age of 8 wk. 1 h after the injection, the mice underwent 1-h ischemia/6-h reperfusion operation. Liver TUNEL staining (E), CC3 immunostaining (F), and MPO immunostaining (G) were performed. Serum levels of proinflammatory cytokines TNFα and IL-6 were detected with ELISA (H). Scale bar: 100 μm (E–G). **(I–L)** AAV8 that was expressing *Adk-L* or control was injected to WT mice through a tail vein. 1 mo after the injection, the mice underwent 1-h ischemia/6-h reperfusion operation. Liver TUNEL staining (I), CC3 immunostaining (J), and MPO immunostaining (K) were performed. Serum levels of proinflammatory cytokines TNFα and IL-6 were detected with ELISA (L). Scale bar: 100 μm (I–K). **(M)** Postreperfusion clinical donor liver injury and inflammation was analyzed with H&E staining, TUNEL staining, CC3 staining, MPO staining, and p-RIPK1 (S166) staining. The donor livers were divided into postreperfusion ADK high- or low-expression groups according to immunoblotting data in Fig. 8 E. The representative images of the two groups are shown. Scale bar: 100 μm. **(N)** Levels of TUNEL^+^ cells, CC3^+^ cells, neutrophils, and p-RIPK1 (S166)–positive cells in postreperfusion clinical donor livers were quantified from staining images in Fig. S4 M. Serum levels of ALT and AST of donor liver recipients on the first day after liver transplantation were collected from their medical records. Postreperfusion ADK expression level was analyzed according to immunoblotting data in Fig. 8 E. The correlations between levels of postreperfusion TUNEL^+^ cells, CC3^+^ cells, neutrophils, ALT, AST, p-RIPK1 (S166)–positive cells, and ADK expression level were analyzed. Data are represented as the mean ± SD (A–N). Data are representative of *n* = 3 independent experiments (A–N). Statistical significance was determined using two-way ANOVA with post hoc Bonferroni’s test (A–L) or Pearson’s correlation test (N). ***P < 0.001.

**Figure S5.**
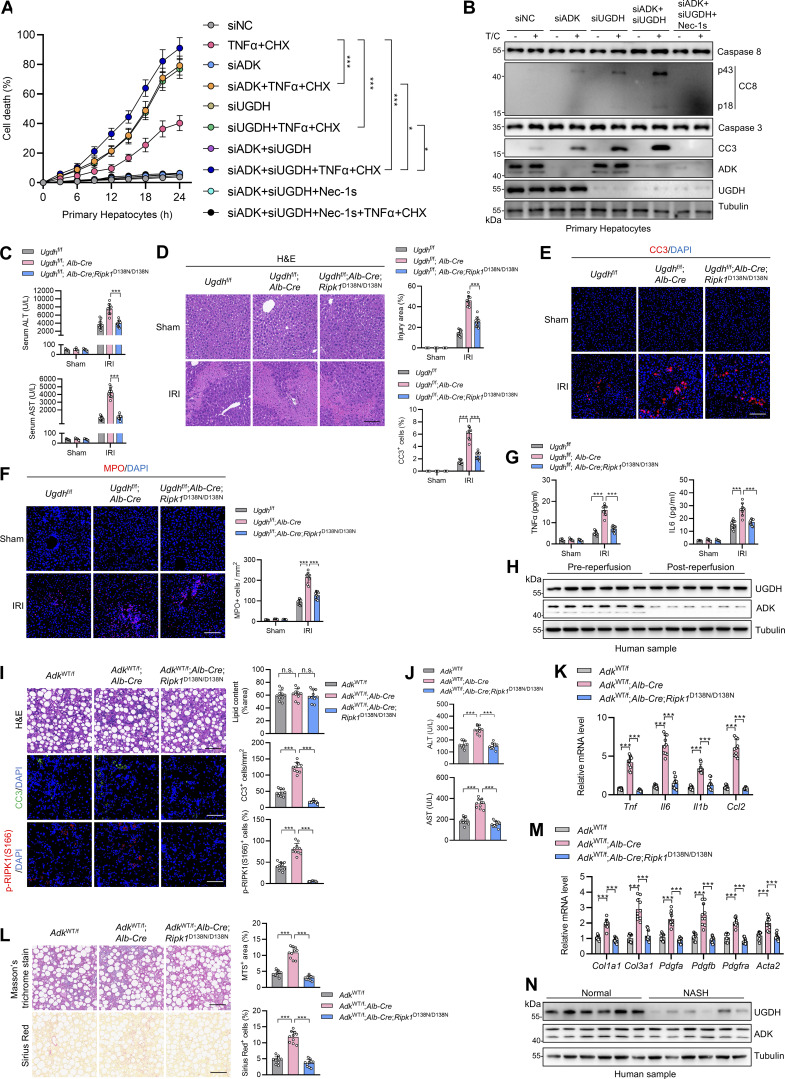
**ADK expression reduction aggravates liver IRI. (A and B)** Primary hepatocytes from WT mice were treated with CHX (1 μM)/TNFα (10 ng/ml) for the indicated time (A) or 12 h (B). Cell death was measured by the SYTOX Green positivity assay (A). The levels of p-RIPK1(S166), CC8, and CC3 were determined by immunoblotting (B). **(C–G)***Ugdh*^f/f^, *Ugdh*^f/f^;*Alb-Cre*, *Ugdh*^f/f^;*Alb-Cre*;*Ripk1*^D138N/D138N^ mice were subjected to 1-h ischemia/6-h reperfusion model. Serum ALT/AST detection (C), H&E staining (D), CC3 immunostaining (E), MPO immunostaining (F), and serum TNFα and IL-6 level detection (G) were performed. Scale bar: 100 μm (D–F). **(H)** Protein expression level of UGDH and ADK in clinical donor liver IRI samples was analyzed with immunoblotting. **(I–M)***Adk*^WT/f^, *Adk*^WT/f^;*Alb-Cre*, *Adk*^WT/f^;*Alb-Cre*;*Ripk1*^D138N/D138N^ mice were fed with choline-deficient high-fat diet (60% fat, 0.1% methionine, and no added choline, A06071302; Research Diets) for 6 wk to construct the NASH model. Liver H&E staining, CC3 immunostaining, and p-RIPK1 (S166) immunostaining were performed (I), serum ALT/AST was measured (J), proinflammatory gene expression was analyzed (K), liver fibrosis was analyzed with Masson’s trichrome staining and Sirius red staining (L), and fibrotic gene expression was analyzed (M). Scale bar: 100 μm (I and L). **(N)** Protein expression level of UGDH and ADK in clinical NASH samples was analyzed with immunoblotting. Data are represented as the mean ± SD of *n* = 3 (A, C–G, and I–M). Data are representative of *n* = 3 independent experiments (A–N). Statistical significance was determined using two-way ANOVA with post hoc Bonferroni’s test (C–G, K, and M) or one-way ANOVA with post hoc Dunnett’s test (I, J, and L). *P < 0.05; ***P < 0.001. Source data are available for this figure: SourceData FS5.

In line with cellular models, hepatic RIPK1 R621me2s was reduced along with decreased ADK expression in human liver IRI samples (Fig. 8 E), which suggests that ADK reduction might aggravate liver IRI triggered by suppressing RIPK1 R621me2s and activating RIPK1. To test this hypothesis, we performed IRI operation on mice of different genotypes. Compared with WT littermates, hepatocyte ADK knockout mice exhibited enhanced RIPK1 activation, leading to more severe cell death, inflammation, and liver injury (Fig. 8, G–I and Fig. S4, A–D). All the above alterations caused by ADK knockout were reversed by RIPK1 kinase-dead mutation (Fig. 8, G–I and Fig. S4, A–D). Similar to genetic knockout, blocking ADK activity with 5-ITu also exacerbated IRI, suggesting that ADK metabolic function is protective in this acute liver injury (Fig. 8, J–L and Fig. S4, E–H). To further link ADK expression levels with IRI, we intravenously injected adeno-associated virus serotype 8 (AAV8) expressing thyroxine-binding globulin promoter–driven ADK-L into WT mice to overexpress ADK specifically in hepatocytes. As expected, ADK overexpression suppressed RIPK1 activation and the subsequent cell death, inflammation, and liver injury (Fig. 8, M–O and Fig. S4, I–L). In clinical patient-derived samples, the donor livers that expressed lower levels of ADK experienced more drastic cell death, inflammation, and RIPK1 kinase activation (Fig. S4, M and N), suggesting that reduced ADK expression also aggravates liver injury by facilitating RIPK1 activation during donor liver transplantation operation. We previously showed that UGDH suppresses RIPK1 kinase activity via its metabolic product UDP-GlcA (Zhang et al., 2023c). The expression of UGDH decreases in nonalcoholic steatohepatitis (NASH), which promotes RIPK1 activation and inflammation. We then explored the relationship between UGDH and ADK in the context of RIPK1 inhibition. Knockdown of either UGDH or ADK in primary hepatocytes promoted TNFα/CHX-induced cell death, and knockdown of both of them further increased cell death (Fig. S5, A and B), suggesting that UGDH and ADK function in independent manners. In mouse models, hepatocyte-specific UGDH knockout exacerbated cell death and inflammation in liver IRI, which was partially reversed by RIPK1 kinase inactivation (Fig. S5, C–G). However, in clinical samples, the UGDH protein level was unchanged during IRI, in contrast to decreased ADK (Fig. S5 H). We also showed that hepatocyte-specific ADK knockout aggravated cell death, inflammation, and fibrosis in the NASH model (Fig. S5, I–M). However, in human samples, the ADK protein level was unchanged during NASH, distinguished from downregulated UGDH (Fig. S5 N). Thus, our data support the regulatory role of UGDH and ADK on RIPK1 kinase activity in disease models. But given that the expression levels of UGDH and ADK reduced exclusively in NASH and liver IRI, respectively, they are two independent pathways that function differentially in distinct diseases. Collectively, these data demonstrate that reduction in ADK expression levels during liver IRI aggravates liver damage by facilitating RIPK1-driven apoptosis and inflammation.

### R606K mutation of RIPK1 protects mice from RIPK1-driven liver damage

**Figure 9.**
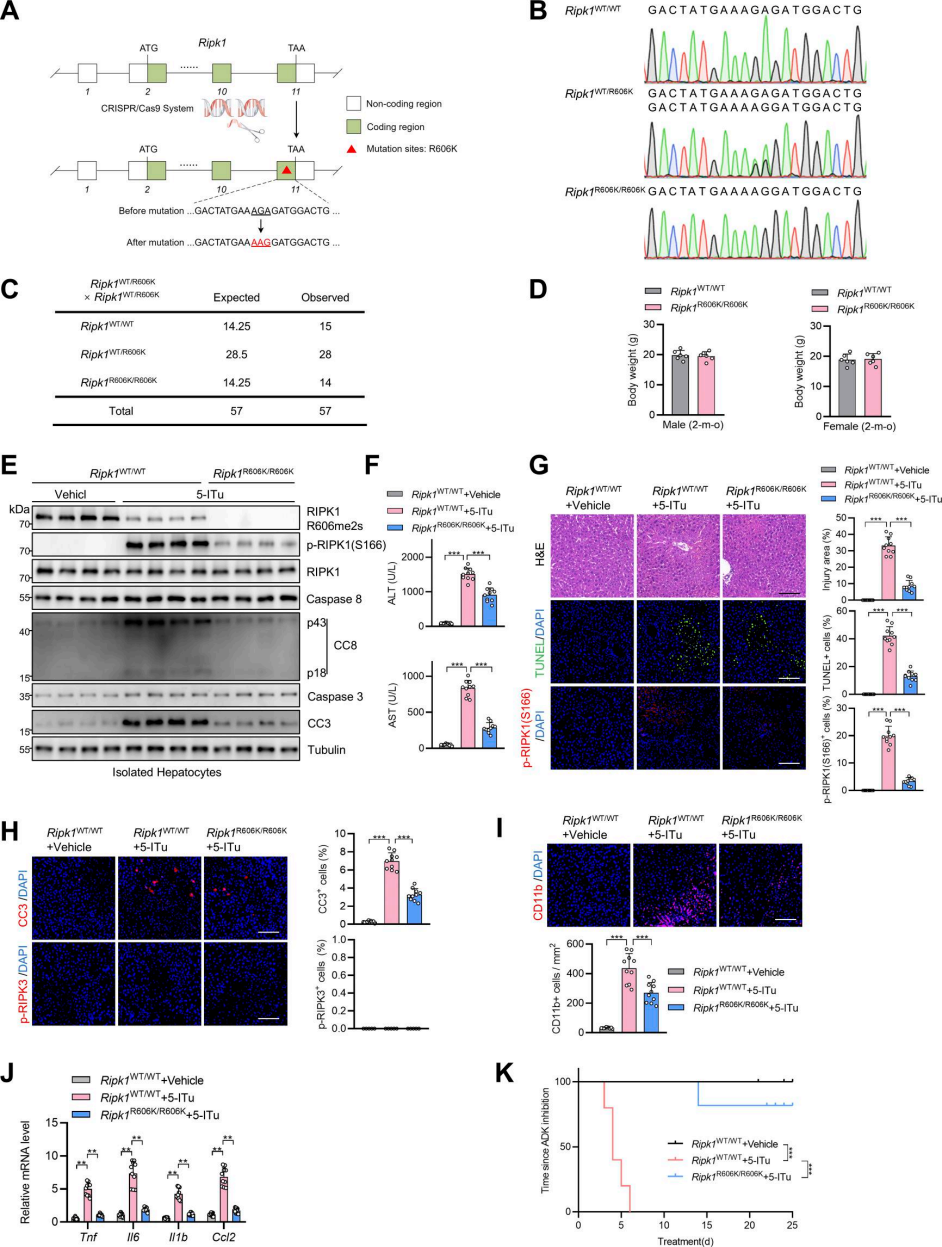
**RIPK1 R606K mutation protects mice against ADK inhibition–induced RIPK1-driven liver injury. (A and B)** Illustration of the generation of *Ripk1*^R606K/R606K^ mice (A) and the genotype as determined by Sanger sequencing (B) are shown. **(C)** Number of offspring from intercrossing of *Ripk1*^WT/R606K^ mice. **(D)** Body weight of male and female mice of *Ripk1*^WT/WT^ or *Ripk1*^R606K/R606K^ genotypes at the age of 2 mo. **(E–K)** Vehicle or 5-ITu (100 mg/kg) was intraperitoneally injected to *Ripk1*^WT/WT^ or *Ripk1*^R606K/R606K^ mice every day. 72 h after the first injection, the levels of p-RIPK1(S166), CC8, CC3, and ADK in hepatocytes isolated from the livers were determined by immunoblotting (E); serum ALT/AST detection was performed (F); liver H&E staining, TUNEL staining, and p-RIPK1(S166) immunostaining were performed (G); liver CC3 and p-RIPK3 immunostaining (H) and CD11b immunostaining (I) were conducted; mRNA levels of *Tnf*, *Il6*, *Il1β*, *Ccl2* (J) were analyzed. The survival time of mice of different groups was analyzed (K). Scale bar: 100 μm (G–I). Data are represented as the mean ± SD (D and F–J). Data are representative of *n* = 3 independent experiments (A–K). Statistical significance was determined using two-tailed unpaired Student’s *t* test (D), one-way ANOVA with post hoc Dunnett’s test (F–I), two-way ANOVA with post hoc Bonferroni’s test (J), or log-rank test (K). **P < 0.01; ***P < 0.001. Source data are available for this figure: SourceData F9.

**Figure 10.**
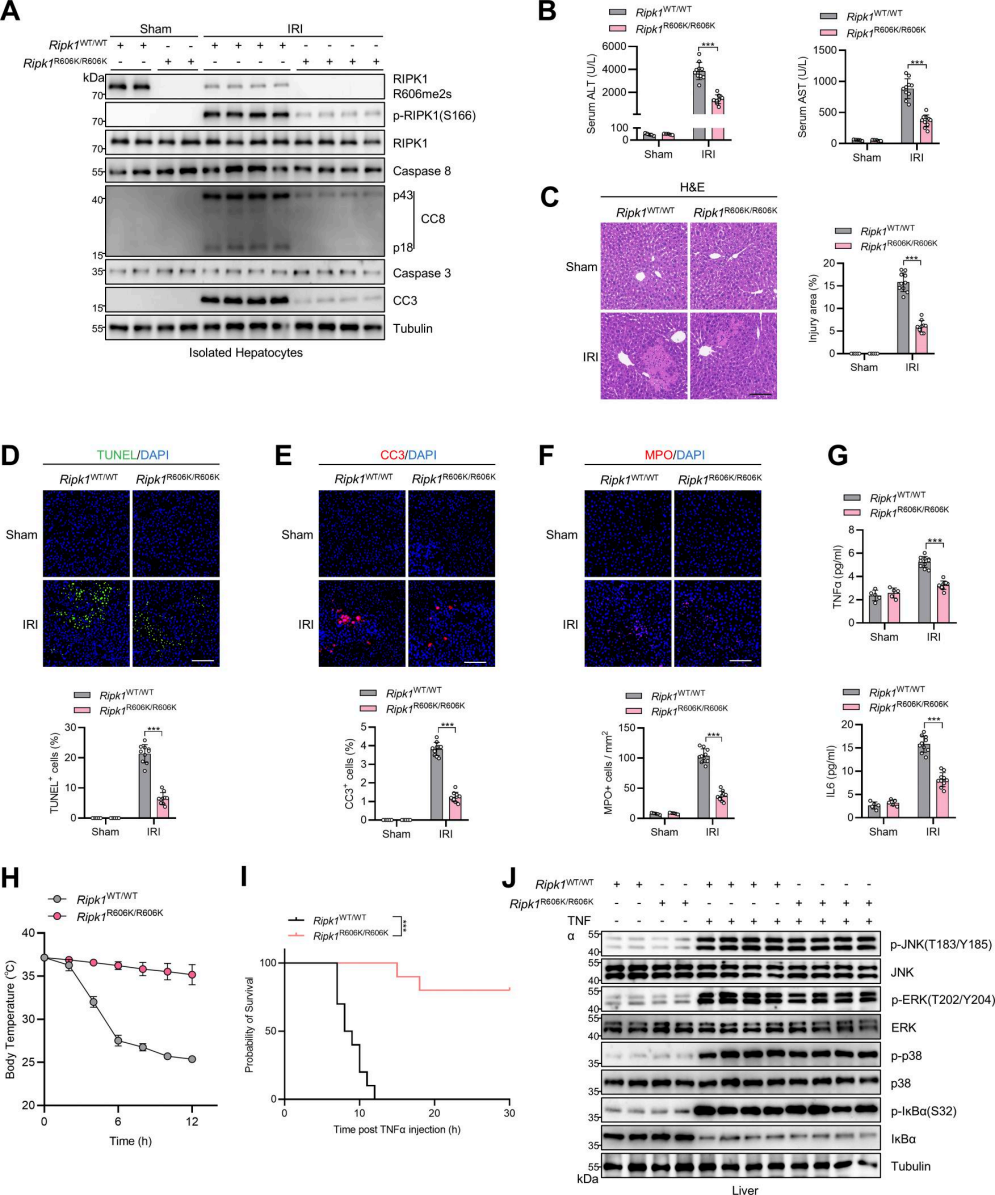
***Ripk1*** ^**R606K/R606K**^ **mice exhibited mitigated RIPK1-driven liver IRI and SIRS. (A–G)** *Ripk1* ^WT/WT^ or *Ripk1*^R606K/R606K^ mice underwent 1-h ischemia/6-h reperfusion operation. The levels of RIPK1 R606me2s, p-RIPK1(S166), CC8, CC3 in isolated hepatocytes were determined by immunoblotting (A). Serum ALT/AST detection (B) and liver H&E staining (C) were performed; liver TUNEL staining (D), CC3 immunostaining (E), MPO immunostaining (F), and serum proinflammatory cytokine detection (G) were performed. Scale bar: 100 μm (C–F). **(H–J)** 500 μg/kg TNFα was intravenously injected to *Ripk1*^WT/WT^ or *Ripk1*^R606K/R606K^ mice. Body temperature (H) and survival time (I) were compared. NF-κB and MAPK activation in liver was analyzed with immunostaining (J). Data are represented as the mean ± SD (B–H). Data are representative of *n* = 3 independent experiments (A–J). Statistical significance was determined using two-way ANOVA with post hoc Bonferroni’s test (B–H) or log-rank test (I). ***P < 0.001. Source data are available for this figure: SourceData F10.

To investigate the physiological significance of R606me2s modification of RIPK1, we generated *Ripk1*^R606K/R606K^ knockin mice with CRISPR/Cas9 technology (Fig. 9, A and B). *Ripk1*^R606K/R606K^ mice were born in normal Mendelian ratios (Fig. 9 C) and grew normally with no significant difference with littermate control *Ripk1*^WT/WT^ mice (Fig. 9 D). We exposed these mice to large-dose 5-ITu administration. Consistent with the notion that R606K mutation abrogated the dimerization of RIPK1 DD, *Ripk1*^R606K/R606K^ mice showed decreased RIPK1 activation and apoptosis (Fig. 9 E). As a result, compared with *Ripk1*^WT/WT^ mice, liver injury and inflammation were attenuated in *Ripk1*^R606K/R606K^ mice (Fig. 9, F–J), which prolonged their survival (Fig. 9 K). We then subjected *Ripk1*^R606K/R606K^ mice to liver IRI operation. In the liver IRI model, R606K mutation also inhibited RIPK1 kinase activation and subsequent apoptosis (Fig. 10 A). Consequently, compared with *Ripk1*^WT/WT^ mice, *Ripk1*^R606K/R606K^ mice exhibited alleviated liver damage as revealed by decreased serum ALT/AST (Fig. 10 B), reduced injury area (Fig. 10 C), and cell death (Fig. 10, D and E). Moreover, inflammation was also mitigated in *Ripk1*^R606K/R606K^ mice as determined by reduced neutrophil infiltration (Fig. 10 F) and proinflammatory cytokine (Fig. 10 G). RIPK1 kinase activity is essential for TNFα-induced systematic inflammatory response syndrome (SIRS) (Duprez et al., 2011; Dondelinger et al., 2019). In our SIRS model, compared with WT mice, RIPK1 R606K mutation prevented the hypothermia and lethality caused by lethal dose of TNFα (Fig. 10, H and I); in contrast, NF-κB and MAPK activation was unaffected by RIPK1 R606K mutation (Fig. 10 J). Thus, these in vivo data are generally consistent with in vitro experiments and suggest that RIPK1 R606me2s plays an important role in maintaining liver homeostasis by suppressing RIPK1 dimerization and kinase activation.

## Discussion

In this study, we establish a following model for how adenosine metabolism maintains hepatic tissue homeostasis in physiological or pathological conditions. ADK-mediated intracellular adenosine removal is prerequisite for keeping a relatively high SAM/SAH ratio that facilitates transmethylation reactions. Among them, PRMT5 constitutively catalyzes symmetric dimethylation at RIPK1 R606 in the DD. When cells sense TNFα, RIPK1 is recruited to complex I and RIPK1 R606me2s suppresses electrostatic interaction–mediated DD associations to prevent RIPK1 dimerization, which keeps cell death in check. If adenosine clearance fails due to ADK abrogation, deficiency of RIPK1 R606me2s promotes RIPK1 dimerization and activation that leads to enhanced TNFα-induced cell death and subsequent tissue homeostasis disruption.

RIPK1 is the major signaling node that orchestrates the TNFα signaling pathway. The RIPK1 scaffold function promotes cell survival, but its kinase function induces cell death; thus, the kinase activity of RIPK1 is normally suppressed by multiple checkpoints to prevent its detrimental consequences and maintain tissue homeostasis (Xu et al., 2021). Typically, genetic evidence reveals that the disablement of one of the checkpoints leads to spontaneous cell death and tissue homeostasis disruption. For instance, hepatocyte-specific knockout of TAK1, a protein kinase that phosphorylates RIPK1 at S321 to inhibit its activation, leads to spontaneous hepatocyte apoptosis, inflammation, liver fibrosis, and hepatocellular carcinoma, which are reversed by RIPK1 kinase-dead mutation (Geng et al., 2017; Tan et al., 2020). Knockout of TBK1, another protein kinase that suppresses RIPK1 activation by phosphorylation at T190, results in embryonic lethality and neurodegeneration, which can also be prevented by RIPK1 inactivation (Xu et al., 2018). Hepatocyte-specific knockout of EGLN1-3, which performs hydroxylation of RIPK1 at multiple proline residues, especially P196, causes liver vascular malformation, hepatocyte apoptosis, and inflammation (Zhang et al., 2023a). Notably, the above checkpoints are composed of metabolism-independent posttranslational modifications of RIPK1. We previously showed that UGDH is a metabolic enzyme that suppresses RIPK1 activation (Zhang et al., 2023c). The catalytic product of UGDH, UDP-gluconate, directly binds to the KD of RIPK1 and thus reduces its catalytic ability. We found that knockdown of either UGDH or ADK in primary hepatocytes promotes TNFα/CHX-induced cell death and knockdown of both of them further increased cell death, suggesting that UGDH and ADK function in independent manners. This is consistent with the notion that cell death checkpoints can act mutually dependently or independently. Disruption of two independent cell death checkpoints can exhibit additive or synergistic effects, such as UGDH/ADK in this study or TBK1/TAK1 in a previous study (Xu et al., 2018). Nevertheless, although UGDH suppresses TNFα-induced cell death and is highly enriched in hepatocytes, hepatocyte-specific UGDH knockout does not trigger spontaneous abnormalities (Zhang et al., 2023c), suggesting that UGDH-mediated metabolism does not play a critical role in maintaining homeostasis in physiological conditions. In contrast to UGDH, hepatocyte-specific knockout of ADK leads to spontaneous hepatocyte apoptosis, inflammation, and premature death, which are protected by RIPK1 kinase inactivation. Thus, we present novel evidence that the metabolic pathway can constitute a cell death checkpoint that restrains the kinase activity of RIPK1 and maintains tissue homeostasis.

The three functional domains of RIPK1, including N-terminal KD, ID, and C-terminal DD, play different roles in the TNFα pathway (Xu et al., 2021). Notably, checkpoints of RIPK1 usually involve posttranslational modifications in RIPK1 KD or ID. Modifications in KD of RIPK1, including phosphorylation at S25 and T190 or hydroxylation at P196, suppress RIPK1 kinase activity (Dondelinger et al., 2019; Xu et al., 2018; Zhang et al., 2023a). Modifications in ID of RIPK1, including phosphorylation at S321, ubiquitination at K376, SUMOylation at K550, also determine whether RIPK1 is activated in response to TNFα stimulation by regulating interactions between RIPK1 and other important signaling proteins (Jaco et al., 2017; Geng et al., 2017; Ea et al., 2006; Yan et al., 2022). In contrast, the function of RIPK1 DD in the TNFα pathway is elucidated only recently (Meng et al., 2018; Imai et al., 2024; Rodriguez et al., 2024), and modifications in the DD of RIPK1 and their role in TNFα pathway remain largely unclear. We find that a critical residue R606 in RIPK1 DD is constitutively symmetrically dimethylated and the methylation suppresses TNFα-induced cell death by preventing DD interaction–mediated RIPK1 dimerization, which deepens and broadens our understanding of how RIPK1 is regulated. DD interaction requires electrostatic interaction facilitated by specific charged residues, while hydrophobic interaction is minimally involved because most hydrophobic residues are buried inside the protein (Meng et al., 2018; Telliez et al., 2000; Huang et al., 1996; Jeong et al., 1999). On the other hand, methylation inhibits the ability of arginine to form electrostatic interactions, especially hydrogen bond, while it increases its size and hydrophobicity (Telliez et al., 2000; Jeong et al., 1999; Meng et al., 2018). Thus, our findings are rational and generally consistent with previous reports on arginine methylation and establish arginine methylation as a novel and critical hierarchy that determines the outcome of the TNFα pathway.

ADK removes adenosine by catalyzing its conversion to AMP (Boison, 2013; Eltzschig, 2009). As adenosine exhibits therapeutic effects in diseases including arrhythmia and neurodegeneration, the strategy of inhibiting ADK to increase adenosine level for therapy has been proposed, which was abrogated by side effects, especially drug-induced liver injury (Boison, 2013). Our findings that excessive adenosine accumulation inhibits RIPK1 R606 dimethylation and promotes TNFα-induced RIPK1 kinase–driven cell death elucidate the mechanism underlying toxicity of adenosine overdose. Pharmacological inhibition of RIPK1 only alleviates adverse effect of intracellular adenosine that is independent on adenosine receptors, while it does not abolish its therapeutic effects that are largely dependent on adenosine receptors, which implies that the RIPK1 kinase block can be utilized to prevent adenosine-induced cell death, while it preserves relevant therapeutic benefits. Our findings also suggest RIPK1 inhibition is potentially applicable to treat diseases that are associated with ADK reduction, such as liver IRI.

Taken together, our results establish that ADK-mediated adenosine clearance licenses RIPK1 R606 symmetric dimethylation to prevent RIPK1 kinase–driven cell death. The findings elucidate a critical mechanism for tissue homeostasis maintenance in physiological conditions that involves a novel RIPK1 posttranslational modification, as well as a new hierarchy of RIPK1 regulation, and suggest RIPK1 inhibition as a feasible strategy to precisely treat diseases that are induced by adenosine overload–triggered cell death.

## Materials and methods

### Human liver samples

Liver samples for pretransplantation analysis were extracted from the left hepatic lobe after cold preservation but prior to the transplantation process. In contrast, posttransplantation samples were gathered from the identical lobe following approximately 2 h of portal reperfusion, just before the closure of the abdominal cavity. These samples were either preserved in formalin for histological examination or snap-frozen in liquid nitrogen for subsequent immunoblotting analysis. The livers used in this study were procured through the China Organ Transplant Response System, with the collection period spanning from August 2022 to October 2023. The research included a total of 24 donor livers. In the context of human sample, adherence to the ethical guidelines of the Declaration of Helsinki was maintained, and the studies were sanctioned by the Ethics Committee of Union Hospital, Tongji Medical College, Huazhong University of Science and Technology (approval no. UHCT-2023-0870). Prior to participation, written consent was obtained from both the human sample donors and the recipients, or their respective family members, ensuring informed agreement.

### Animals

*Adk* ^f/f^ (T013684) mice were obtained from GemPharmatech, China*. Alb-Cre* (NM-KI-220458) mice were obtained from the Shanghai Model Organisms Center, China. *Ripk1*^D138N/D138N^ mice were generated as previously described (Liu et al., 2024; Zhang et al., 2023c). Littermate control was employed for these gene knockout or knockin mice. All mice were in the C57BL/6J background. All animals were housed in a specific pathogen–free environment with no more than five mice per cage. They were subjected to controlled light conditions (12-h light and 12-h dark cycle), temperature (24 ± 2°C), and humidity (50 ± 10%) levels. The animals were provided with ad libitum access to food and water throughout the duration of the experiments. The study adhered to regulations concerning animal welfare. General welfare checks were conducted daily. Before each individual experiment, the animals were thoroughly evaluated for suitability based on preset criteria approved by local animal welfare authorities. Mice were euthanized by isoflurane overdose followed by cervical dislocation prior to sample harvest. All procedures involving animals were conducted following the protocols approved by the Institutional Animal Care and Use Committee of Huazhong University of Science and Technology, with the specific approval code 2023–3548.

### Cell lines

HEK293T and MEFs were cultured in high-glucose DMEM containing 100 U/ml penicillin and 100 mg/ml streptomycin supplemented with 10% FBS. The cells were maintained at 37°C and 5% CO_2_. Primary hepatocytes were isolated as detailed below.

### Mouse IRI model

The mouse 70% warm IRI model was employed using male mice at 6 wk of age. Prior to surgery, mice were anesthetized with 50 mg/kg sodium pentobarbital to ensure the absence of toe-clamping pain reflex and corneal reflex. The abdominal skin was then sterilized using a 75% ethanol and Betadine solution, repeated three times. The surgical procedure involved a midline incision of the abdomen, layer by layer, after which the left and middle branches of the portal vein were identified, isolated, and ligated to induce partial ischemia of the liver for 1 h. Reperfusion of the liver was visually confirmed upon releasing the ligations of the left and middle branches of the portal vein. 6 h after reperfusion, mice were reanesthetized for blood collection from the orbital vein. Following blood sampling, the liver was perfused with saline through the portal vein, and the left and middle lobes were resected for subsequent analysis.

### Chemical treatment in vitro

The following chemicals were utilized for treatment in cell culture: TNFα (P6020; Beyotime), CHX (HY-12320; MedChemExpress), Nec-1s (HY-14622A; MedChemExpress), zVAD.fmk (HY-16658B; MedChemExpress), adenosine (ST1074; Beyotime), 5-ITu (HY-15424; MedChemExpress), SAM (HY-B0617; MedChemExpress), DZNep (HY-10442; MedChemExpress), 5z7 (HY-12686; MedChemExpress).

### Administration of 5-ITu and Nec-1s in vivo

5-ITu (HY-15424; MedChemExpress) and Nec-1s (HY-14622A; MedChemExpress) were dissolved in dimethyl sulfoxide and transferred to 40% polyethylene glycol solution. 5-ITu and Nec-1s were administered intraperitoneally as a dose of 100 and 20 mg/kg body weight every day, respectively.

### Primary hepatocyte isolation

Following the administration of sodium pentobarbital anesthesia at a dosage of 50 mg/kg, and verification of the lack of both pinch and corneal reflexes, the mice underwent a thorough disinfection of the abdominal area using a sequence of 75% ethanol and Betadine solution, which was repeated thrice. This was followed by a midline incision to access the portal vein. In preparation for the extraction of primary hepatocytes from the mouse liver, the organ was subjected to a complete enzymatic digestion via portal vein perfusion. The perfusion process commenced with the use of Liver Perfusion Medium (17701-038; Life Technologies), which was then succeeded by Liver Digestion Medium (17703-034; Life Technologies), both at a steady rate of 2 ml/min for a duration of 5 min each. Upon completion of the digestion, the liver was swiftly removed and placed into chilled, sterile phosphate-buffered saline (PBS) solution. The hepatic tissue was then diced into small pieces, and the subsequent cell suspension was passed through a 70-μm mesh cell strainer (352350; Falcon) to separate the cells. The purification of primary hepatocytes was achieved through a series of centrifugation steps at 50 × *g* for 3 min, with this procedure being conducted three times to ensure cell isolation.

### Immunoblotting

For the purpose of immunoblotting analysis, a set of primary antibodies targeting specific proteins was utilized: cleaved caspase-8 (8592; Cell Signaling Technology), cleaved caspase-3 (9664; Cell Signaling Technology), ADK (ab307357; Abcam), tubulin (HC101-01; TransGen), p-RIPK1(S166) (53286; Cell Signaling Technology), RIPK1 (3493; Cell Signaling Technology), FADD (ab124812; Abcam), p-RIPK3 (91702; Cell Signaling Technology), RIPK3 (95702; Cell Signaling Technology), p-MLKL (37333; Cell Signaling Technology), MLKL (ab243142; Abcam), p-JNK (4671; Cell Signaling Technology), JNK (24164-1-AP; Proteintech), p-ERK (4370; Cell Signaling Technology), ERK (9102; Cell Signaling Technology), p-p38 (9216; Cell Signaling Technology), p38 (14064-1-AP; Proteintech), p-IκBα (2859; Cell Signaling Technology), IκBα (4814; Cell Signaling Technology), SDMA (13222; Cell Signaling Technology), ADMA (13522; Cell Signaling Technology), Flag (F1804; Sigma-Aldrich), HA (sc-7392; Santa Cruz), PRMT5 (79998; Cell Signaling Technology), TRADD (3694; Cell Signaling Technology). Secondary antibodies used in this study included the following: HRP-conjugated AffiniPure Goat Anti-Mouse IgG(H+L) (SA00001-1; Proteintech), HRP-conjugated AffiniPure Goat Anti-Rabbit IgG(H+L) (SA00001-2; Proteintech). Signal detection was carried out using Immobilon ECL Ultra Western HRP Substrate (Millipore). Following incubation in Restore Western Blot Stripping Buffer (Thermo Fisher Scientific ), the membranes were reprobed.

### Analysis of cytotoxicity and viability

To evaluate the cytotoxic effects of various treatments, primary hepatocytes were exposed to SYTOX Green (Invitrogen) at a concentration of 5 mM within their culture medium. The fluorescence intensity of SYTOX Green was quantified employing a fluorescence enzyme marker (BioTek). A negative control was established to ascertain the baseline fluorescence intensity of SYTOX Green. Cell death percentage was calculated as (fluorescence signal from treated samples − fluorescence signal from negative control)/(maximum fluorescence signal − fluorescence signal from negative control). Cells subjected to Triton X-100 served as a positive control, facilitating the induction of maximum fluorescence intensity.

### Immunoprecipitation

The cells were first washed twice with precooled PBS. Following this, cell lysis was performed using 1 ml of a 1% NP-40 lysis buffer (P0013F; Beyotime Biotechnology), which was supplemented with phosphatase and protease inhibitors. The lysate was then gently mixed at 4°C for 30 min prior to centrifugation at 12,000 × *g* for 15 min at 4°C. The supernatant was collected and transferred to a fresh centrifuge tube. Subsequently, the target antibody was introduced, and the mixture was incubated overnight at 4°C with gentle rocking. On the subsequent day, Protein A/G Beads (HY-K0202; MedChemExpress) were added to the tube, mixed, and incubated for 2 h at 4°C with continuous agitation. After incubation, the beads underwent three washes with the 1% NP-40 buffer. Ultimately, the beads were collected with magnetic grate and the protein sample was eluted by heating in 50 μl of 1× SDS sample buffer. The sample was then subjected to immunoblotting analysis or mass spectrometry analysis.

### TUNEL staining

After the removal of paraffin from the sections, they were treated with 20 μg/ml DNase-free proteinase K for a duration of 30 min at room temperature, followed by triple rinsing with PBS. Subsequently, TUNEL staining was applied using the In Situ Cell Death Detection Kit POD (Roche). The staining solution was mixed as per the manufacturer’s instructions, and the sections were then incubated for 60 min at 37°C in a light-protected environment. After incubation, the nuclei were stained with DAPI and the sections were rinsed three times with PBS. The quantification of TUNEL-positive cells in relation to the overall cell population was carried out utilizing Fiji software.

### Serum biochemical analysis

Blood samples were obtained, and serum was subsequently separated by gradient centrifugation. Thereafter, the levels of ALT and AST in the serum were quantified using ALT Assay Kit (Nanjing Jiancheng Bioengineering Institute, C009-2-1) and the AST Assay Kit (Nanjing Jiancheng Bioengineering Institute, C010-1-1), respectively. These assays adhered to the protocols provided with the kits and employed colorimetric techniques for the detection of enzyme activities.

### Immunofluorescence and immunohistochemistry

Liver tissues were preserved in a 4% paraformaldehyde solution and then encapsulated in paraffin. Prior to the execution of immunohistochemistry and immunofluorescence assays, the paraffin-embedded sections were subjected to deparaffinization and antigen retrieval processes using a 0.01 M sodium citrate buffer. To inhibit intrinsic peroxidase activity, the sections were exposed to a 3% solution of H_2_O_2_ for a period of 15 min, succeeded by a series of three times of PBS rinses. Subsequently, the sections were immersed in a 5% solution of goat serum for a duration of 1 h to prevent nonspecific binding. Thereafter, the sections were treated with primary antibodies specific to the proteins of interest and incubated at a temperature of 4°C overnight. Following this incubation, the sections underwent three PBST rinses, which is a solution of PBS with Tween-20, and were then treated with the corresponding secondary antibodies for a period of 2 h at room temperature. In the case of immunohistochemistry, the sections were colored using 3,3′-diaminobenzidine for visualization, and subsequently counterstained with hematoxylin. Once the staining processes were complete, the sections were mounted and secured. Microscopic images were captured using a microscope fitted with either a 20× or 40× objective lens.

### Enzyme-linked immunosorbent assay

The levels of TNFα, CCL2, and LIF in the serum were quantified using specific ELISA kits following the protocols provided by the manufacturers: mouse TNFα (KE10002; Proteintech), mouse IL-6 (KE10007; Proteintech). Optical density was recorded with a microplate reader, and the serum protein concentrations were calculated by referencing the standard curves supplied with the respective assay kits.

### In vitro methylation assay

To achieve in vitro methylation of RIPK1, a 50 μl reaction mixture containing 250 nM RIPK1 protein purified from *PRMT5*^−/−^ HEK293T cells and 40 μM SAM (final concentration) in PBS buffer was prepared. The reaction was initiated by introducing 1 μg of recombinant PRMT5 and 1 μg of recombinant WDR77 (MEP50) and incubated at 30°C for a duration of 1.5 h. The process was halted by adding 10 μl of 4×SDS loading buffer. The mixture was then subjected to analysis via 10% SDS-PAGE, and methylation levels were detected through immunoblotting using an RIPK1 R606me2s antibody. Additionally, the formation of SAH was evaluated using the MTase-Glo Methyltransferase Assay (V7601) kit, adhering to the manufacturer’s guidelines.

### Lentivirus production and infection

In the process of generating lentiviruses for gene overexpression, HEK293T cells were cotransfected with the pLenti-puromycin vector encoding target proteins, along with pMD2.G and psPAX2 plasmids, for a period of 48 h. The supernatant was collected and filtered before centrifugation. The concentrated lentivirus was applied to target cells, supplemented with polybrene at a concentration of 8 mg/ml. After a 6-h incubation with the lentiviral particles, the medium was refreshed. The cells underwent selection with puromycin 24 h after infection.

### Construction of plasmids and transfection

Polymerase chain reaction (PCR)-amplified mouse RIPK1 and mouse ADK were cloned into pLenti/puro(+)-Flag. pLenti/puro (+)-Flag RIPK1 R606K was generated using the QuikChange site-directed mutagenesis kit (Stratagene). Transfection was performed using Lipofectamine 3000 transfection reagent (Invitrogen) according to the manufacturer’s instructions.

### Measurement of metabolite concentration

For measurement of adenosine concentration, the adenosine assay (MET-5090; Cell Biolabs) was utilized. For measurement of SAM and SAH concentration, SAM and SAH ELISA Combo Kit (MET-5151-C; Cell Biolabs) was utilized. For measurement of AMP, ADP, and ATP concentrations, AMP-Glo assay (V5011; Promega), ADP-Glo assay (V6930; Promega), and CellTiter-Glo assay (G7570; Promega) were utilized. All procedures were conducted according to the manufacturer’s instructions.

### Mass spectrometry

Primary mouse hepatocytes were isolated from WT 8-wk-old male mice. This was followed by the procedure of RIPK1 immunoprecipitation. The precipitated complexes were then subjected to five successive washes using NP-40 and PBS. An appropriate amount of TEAB was added to adjust pH 8.0. 5 μl of suspension was used for SDS-PAGE testing. For digestion, the protein solution was reduced with 5 mM dithiothreitol for 30 min at 56°C and alkylated with 11 mM iodoacetamide for 15 min at room temperature in darkness. The protein sample was then diluted by adding 100 mM TEAB to urea concentration <2 M. Finally, trypsin was added at 1:50 trypsin-to-protein mass ratio for the first digestion overnight and 1:100 trypsin-to-protein mass ratio for a second 4-h digestion. Finally, the peptides were desalted by a C18 SPE column. The tryptic peptides were dissolved in solvent A, directly loaded onto a homemade reversed-phase analytical column (25 cm length, 100 μm i.d.). The mobile phase consisted of solvent A (0.1% formic acid, 2% acetonitrile/in water) and solvent B (0.1% formic acid, 90% acetonitrile/in water). Peptides were separated with the following gradient: 0–68 min, 6%-23%B; 68–82 min, 23%-32%B; 82–86 min, 32%-80%B; 86–90 min, 80%B, and all at a constant flow rate of 500 nl/min on an EASY-nLC 1200 UPLC system (Thermo Fisher Scientific). The separated peptides were analyzed in Orbitrap Exploris 480 with a nano-electrospray ion source. The electrospray voltage applied was 2,300 V. Precursors and fragments were analyzed at the Orbitrap detector. The full MS scan resolution was set to 60,000 for a scan range of 400–1,200 m/z. The MS/MS scan was fixed first mass as 110 m/z at a resolution of 15,000 with the TurboTMT set as off. Up to 25 most abundant precursors were then selected for further MS/MS analyses with 20-s dynamic exclusion. The HCD fragmentation was performed at a normalized collision energy of 27%. Automatic gain control target was set at 100%, with an intensity threshold of 50,000 ions/s and a maximum injection time of Auto.

### Construction and injection of AAV8

The AAV8 delivery system was employed to overexpress *Adk-L* in the livers of WT mice (pAAV-TBGp-MCS-Flag-SV40 PolyA). AAV8 vectors were generated through the transfection of three plasmids (pAAV flanked by the AAV inverted terminal repeat sequences, pAAV8 trans-plasmid with the AAV rep and cap genes, and the pAAV helper plasmid) into HEK293T cells. The AAV titers were quantified at 1 × 10^12^ viral genomes per milliliter (v.g./ml). Subsequently, mice were administered 2 × 10^11^ v.g. in 200 μl of virus via the tail vein. The AAV8 used in this study was procured from GeneChem (China).

### Generation of RIPK1 R606me2s antibody

The RIPK1 R606me2s antibody is a custom-produced rabbit polyclonal antibody developed by Thermo Fisher Scientific utilizing the 2-rabbit 90-day protocol. In this process, two rabbits were immunized with an initial injection of 0.25 mg and booster injections of 0.10 mg (administered on days 14, 42, and 56) of a synthesized peptide corresponding to RIPK1 R606me2s. Serum was collected from the rabbits on day 70 and underwent purification through positive selection for affinity to the methylated peptide, followed by negative selection to ensure no affinity to the nonmethylated peptide. The procedure yielded a final purified specific antibody at a concentration of 0.5 mg/ml.

### Generation of *Ripk1*^R606K^ mutant mice

The CRISPR/Cas9 system, comprising Cas9 nuclease, single-guide RNA (sgRNA), and donor vector, was microinjected into fertilized oocytes of C57BL/6J mice to facilitate homologous recombination. Positive F0-generation mice were subsequently validated through PCR amplification and Sanger sequencing. To establish a stably heritable F1 mouse model, confirmed F0 founders were outcrossed with WT C57BL/6J mice, yielding heterozygous *Ripk1*^WT/C257S^ mutant offspring. Genetic stabilization was achieved through successive backcrossing with the C57BL/6J background strain. The target sequence was 5′-CAT​GAC​TAT​GAA​AGA​GAT​GGA​CTG​AAA-3′. The donor vector sequence was 5′-AGG​CAG​TGG​AAA​AAC​TGT​GCC​CGC​AAG​CTG​GGC​TTC​ACT​GAG​TCT​CAG​ATC​GAT​GAA​ATC​GAC​CAT​GAC​TAT​GAA​AAG​GAT​GGA​CTG​AAA​GAG​AAA​GTT​TAC​CAA​ATG​CTT​CAG​AAG​TGG​CTG​ATG​CGG​GAA​GGC​ACC​AAA​GGG​GCC​ACA-3′, where the point mutation is underlined.

### Enzyme-linked immunosorbent assay

Serum TNFα and IL-6 concentrations were determined with the following ELISA kit according to the manufacturer’s instructions: mouse TNFα (KE10002; Proteintech), mouse IL-6 (KE10091; Proteintech).

### RNA isolation and quantitative reverse transcription–PCR

RNA was isolated utilizing the RNeasy Plus Mini kit (74134; Qiagen). Following this, complementary DNA (cDNA) was generated through the reverse transcription process of RNA, employing qPCR Master Mix (Q311-02; Vazyme). Quantitative real-time PCR (qRT-PCR) assays were executed on a Bio-Rad CFX96 platform, utilizing SYBR Green–based qPCR reagents (1725275; Bio-Rad). The composition of the qRT-PCR mixture included 0.8 μl of each primer (forward and reverse), 10 μl of SYBR Green dye, 4.4 μl of the cDNA sample, and 4 μl of DEPC-treated water. The primers used were as follows: mouse *Tnf* (forward: 5′-CCC​TCA​CAC​TCA​GAT​CAT​CTT​CT-3′, reverse: 5′-GCT​ACG​ACG​TGG​GCT​ACA​G-3′), mouse *Il6* (forward: 5′-CCA​AGA​GGT​GAG​TGC​TTC​CC-3′, reverse: 5′-CTG​TTG​TTC​AGA​CTC​TCT​CCC​T-3′), mouse *Il1b* (forward: 5′-GCA​ACT​GTT​CCT​GAA​CTC​AAC​T-3′, reverse: 5′-ATC​TTT​TGG​GGT​CCG​TCA​ACT-3′), mouse *Ccl2* (forward: 5′-TTA​AAA​ACC​TGG​ATC​GGA​ACC​AA-3′, reverse: 5′-GCA​TTA​GCT​TCA​GAT​TTA​CGG​GT-3′).

### siRNA transfection

For gene knockdown, the siRNAs targeting the following sequences were mixed and transfected to ensure knockdown efficiency and avoid off-target effects: si*Adk*-1 (3′UTR: 5′-GTG​CTA​CTT​CTA​GGA​CCT​T-3′, 5′-CCT​GAT​AAT​TGT​CAG​ATA​A-3′, 5′-GAC​CTT​TAG​TCT​CTG​AAA​T-3′, 5′-CCT​GCT​TAC​TGA​TGG​TAC​T-3′), si*Adk*-2 (CDS: 5′-CCT​TGA​TAA​GTA​TTC​TCT​G-3′, 5′-GTA​TTG​AAA​GTG​GCT​CGC​T-3′, 5′-GCC​GCC​AAT​TGT​TAC​AAG​A-3′, 5′-GTA​TTG​AAA​GTG​GCT​CGC​T-3′), si*Prmt1* (5′-CGC​AAC​TCC​ATG​TTT​CAC​A-3′, 5′-GCT​GAG​GAC​ATG​ACA​TCC​A-3′, 5′-CCG​ACA​ATA​TAA​AGA​CTA​C-3′, 5′-GCA​AGT​GAA​GAG​GAA​CGA​C-3′), si*Prmt2* (5′-CGG​GTT​CTG​TTG​TGT​TAC​A-3′, 5′-GCT​GTG​TAT​ATA​GGT​GTT​C-3′, 5′-GCC​AAA​GTC​GAA​TCA​TAT​C-3′, 5′-GAG​GAG​TAC​TTC​GAC​AGC​T-3′), si*Prmt3* (5′-GCA​CAG​AAA​GTT​ACA​GAG​A-3′, 5′-CGC​ACA​GAA​AGT​TAC​AGA​G-3′, 5′-CCT​ACG​GTT​GAA​TAT​ATG​A-3′, 5′-CCT​CAT​TGT​GAC​CCT​GAC​T-3′), si*Carm1* (5′-CCA​CGA​TTT​CTG​TTC​TTT​C-3′, 5′-GCC​ATG​AAG​ATG​TGT​GTG​T-3′, 5′-CCC​GAC​CAA​CAC​CAT​GCA​C-3′, 5′-GTT​GCT​TTC​ATT​GGC​TCC​A-3′), si*Prmt5*-1 (5′-GCA​CAG​TTT​GAG​ATG​CCT​T-3′, 5′-CCC​ATC​AAA​TAC​TCT​CAA​T-3′, 5′-CCA​GAA​CAT​CTG​TGT​GCG​T-3′, 5′-CCT​CTT​GTG​AAT​GCG​TCT​C-3′), si*Prmt5*-2 (5′-GAA​GCA​GCT​CTG​AGT​TCT​C-3′, 5′-GCG​TTT​CAA​GAG​GGA​GTT​C-3′, 5′-CAG​CTC​TGA​GTT​CTC​TTC​C-3′, 5′-CTC​CAG​TAC​TTG​GAA​TAC​T-3′), si*Prmt6* (5′-CGC​ATA​CTT​CTG​CGC​TAC​A-3′, 5′-GTG​GAA​CAA​GAT​ACG​GAC​A-3′, 5′-CCA​GCT​GTA​CTA​CGA​GTG​C-3′, 5′-CGC​ATA​CTT​CTG​CGC​TAC​A-3′), si*Prmt7* (5′-GCC​TCG​GTT​TGG​AGA​AAT​C-3′, 5′-GCC​GAC​ATG​CTA​CAT​GAC​A-3′, 5′-GCC​TCA​AGA​GAT​CCT​GAC​T-3′, 5′-AGG​TGT​TTA​CAG​TTG​AGA​G-3′), si*Prmt9* (5′-AGC​ACC​AAC​CCT​ATG​ATG​C-3′, 5′-CGG​GGA​TGC​CAC​TCA​TCT​T-3′, 5′-GCG​AGA​CTT​ATC​CAT​GCC​G-3′, 5′-GCA​CTG​TCT​AGG​TGA​CCA​G-3′), si*Adora1* (5′-CCT​CAT​CTA​CAT​TGC​CAT​C-3′, 5′-CCA​TTG​CTC​CTC​ATG​GTT​C-3′, 5′-CCC​GGA​AAT​GTA​CTG​GTG​A-3′, 5′-GCT​ACA​CAT​CTT​GAA​CTG​C-3′), si*Adora2a* (5′-CGA​AGG​GCA​TCA​TTG​CGA​T-3′, 5′-GAG​GAC​GTA​GTA​CCC​ATG​A-3′, 5′-GCA​GAA​CGT​CAC​AAA​CTT​C-3′, 5′-CCA​CAG​CAA​TTC​CGT​TGT​C-3′), si*Adora2b* (5′-CCC​ATG​AGC​TAC​ATG​GTG​T-3′, 5′-CCC​GCT​CAG​GTA​TAA​AGG​T-3′, 5′-CGT​GGC​TAA​CAA​ATA​CAT​T-3′, 5′-CCC​ACC​AAC​TAC​TTT​CTG​G-3′), si*Adora3* (5′-CCT​CAG​ATT​CTT​TGG​ACT​C-3′, 5′-GCA​AGT​CAA​GAT​GCA​CTT​C-3′, 5′-CGT​GGT​CAG​TTT​GGA​TTA​C-3′, 5′-CCT​ATT​GTC​TAC​GCC​TGC​A-3′). Transfection was performed using Lipofectamine 3000 transfection reagent (Invitrogen) according to the manufacturer’s instructions.

### Statistics

Data are presented as the mean value plus or minus the standard deviation. Each experiment was conducted a minimum of three times to ensure reliability. Statistical evaluations were carried out utilizing GraphPad Prism software, version 8.0 (specifically v8.4.1). The Shapiro–Wilk test was applied to ascertain whether the data samples adhered to a normal distribution. In cases where the data were normally distributed, two-tailed Student’s *t* test was implemented for comparing two groups. For assessing differences among several groups with a defined control, a one-way ANOVA coupled with Dunnett’s posttests was the chosen method. When analyzing data with multiple variables, a two-way ANOVA was employed, followed by Bonferroni’s post hoc tests for multiple comparisons. For datasets that did not conform to a normal distribution, nonparametric tests were utilized; the Mann–Whitney U test was applied for comparing just two groups, while the Kruskal–Wallis test, succeeded by Dunnett’s posttests, was used for evaluating multiple group differences. P < 0.05 was considered statistically significant, denoted as *, P < 0.01 as **, P < 0.001 as ***, and nonsignificant results as n.s.

### Online supplemental material

Fig. S1 shows the regulation of ADK on RIPK1 activation and TNFα-induced cell death, related to Fig. 1. Fig. S2 shows the regulation of adenosine on RIPK1 activation and TNFα-induced cell death, related to Fig. 2. Fig. S3 shows the regulation of PRMT5 on RIPK1 activation and TNFα-induced cell death, related to Fig. 6. Fig. S4 shows the regulation of ADK on liver IRI, related to Fig. 8. Fig. S5 shows the relationship between ADK and UGDH in the TNFα pathway.

## Supplementary Material

SourceData F1 is the source file for Fig. 1.

SourceData F2 is the source file for Fig. 2.

SourceData F3 is the source file for Fig. 3.

SourceData F4 is the source file for Fig. 4.

SourceData F5 is the source file for Fig. 5.

SourceData F6 is the source file for Fig. 6.

SourceData F7 is the source file for Fig. 7.

SourceData F8 is the source file for Fig. 8.

SourceData F9 is the source file for Fig. 9.

SourceData F10 is the source file for Fig. 10.

SourceData FS1 is the source file for Fig. S1.

SourceData FS2 is the source file for Fig. S2.

SourceData FS3 is the source file for Fig. S3.

SourceData FS5 is the source file for Fig. S5.

## Data Availability

The mass spectrometry data of RIPK1 arginine dimethylation are deposited in the PRIDE database (https://www.ebi.ac.uk/pride) under the accession number PXD064410. All other data are available in the article.
