# Supplementary material for: Adenosine metabolic clearance maintains liver homeostasis by licensing arginine methylation of RIPK1
Source: J Exp Med. 2025 Oct 13;223(1):e20250603. doi: 10.1084/jem.20250603 (PMC12517274; doi:10.1084/jem.20250603)

Uncropped blots_Figure 1

Panel B

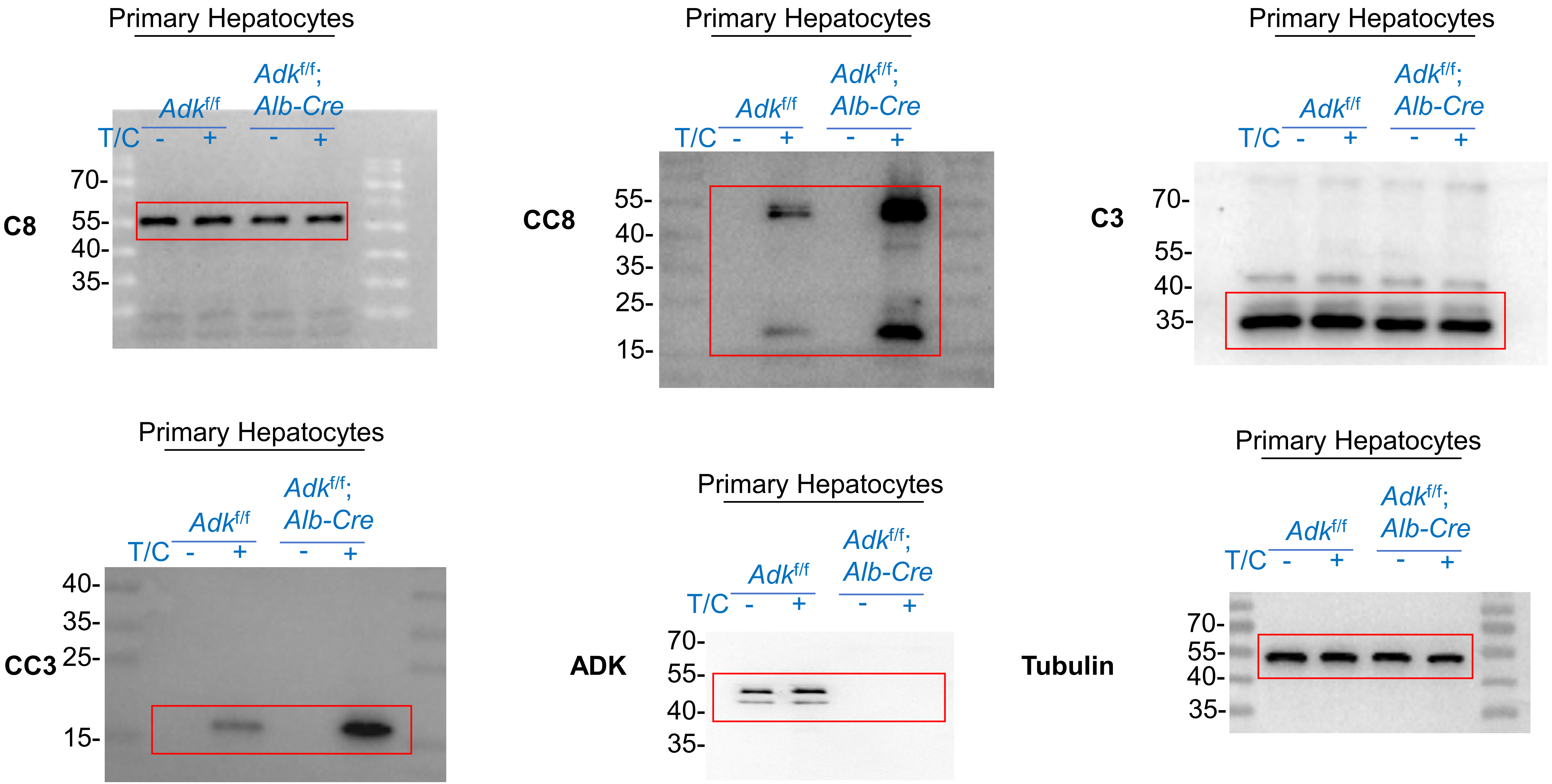

Uncropped blots_Figure 1

Panel D

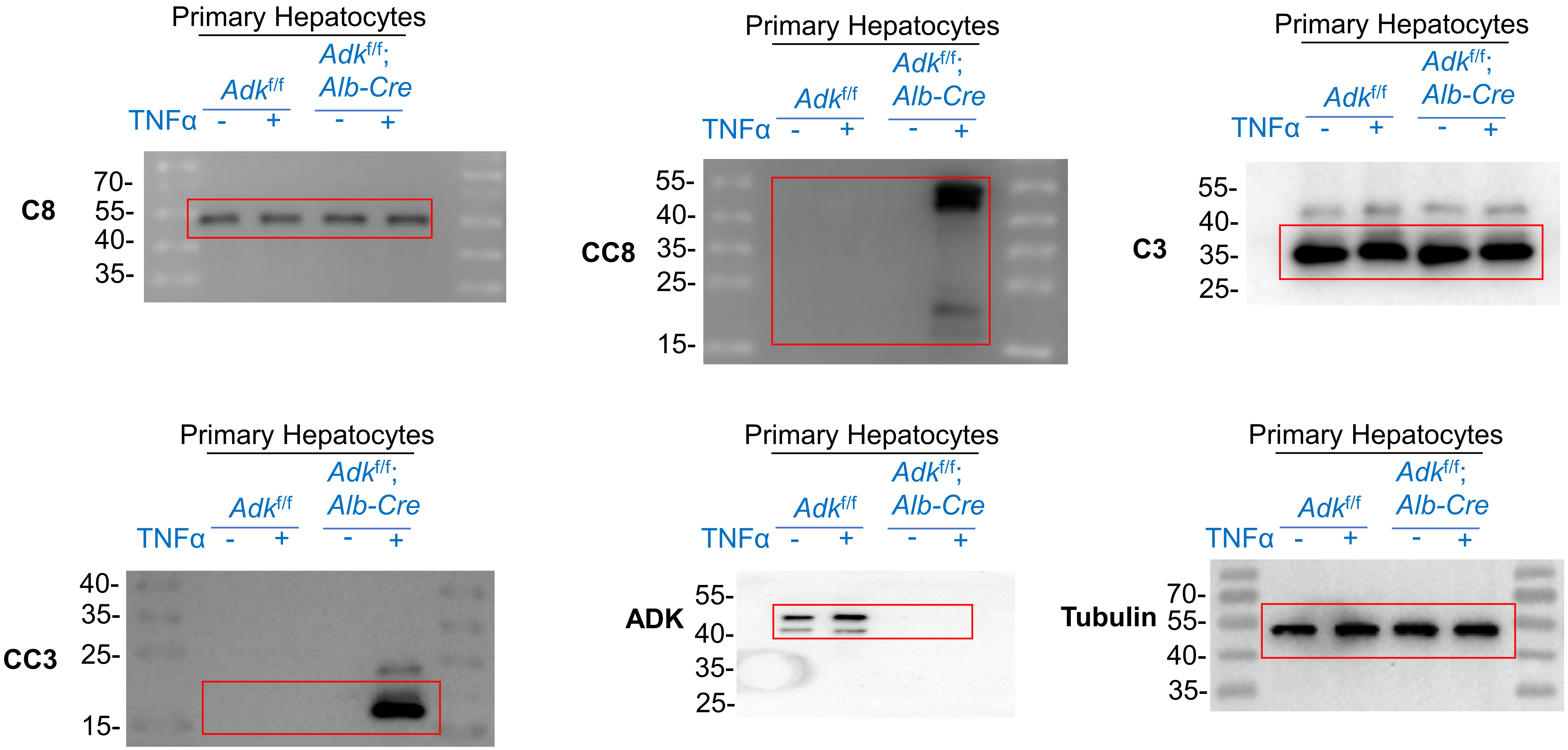

Uncropped blots_Figure 1

Panel F

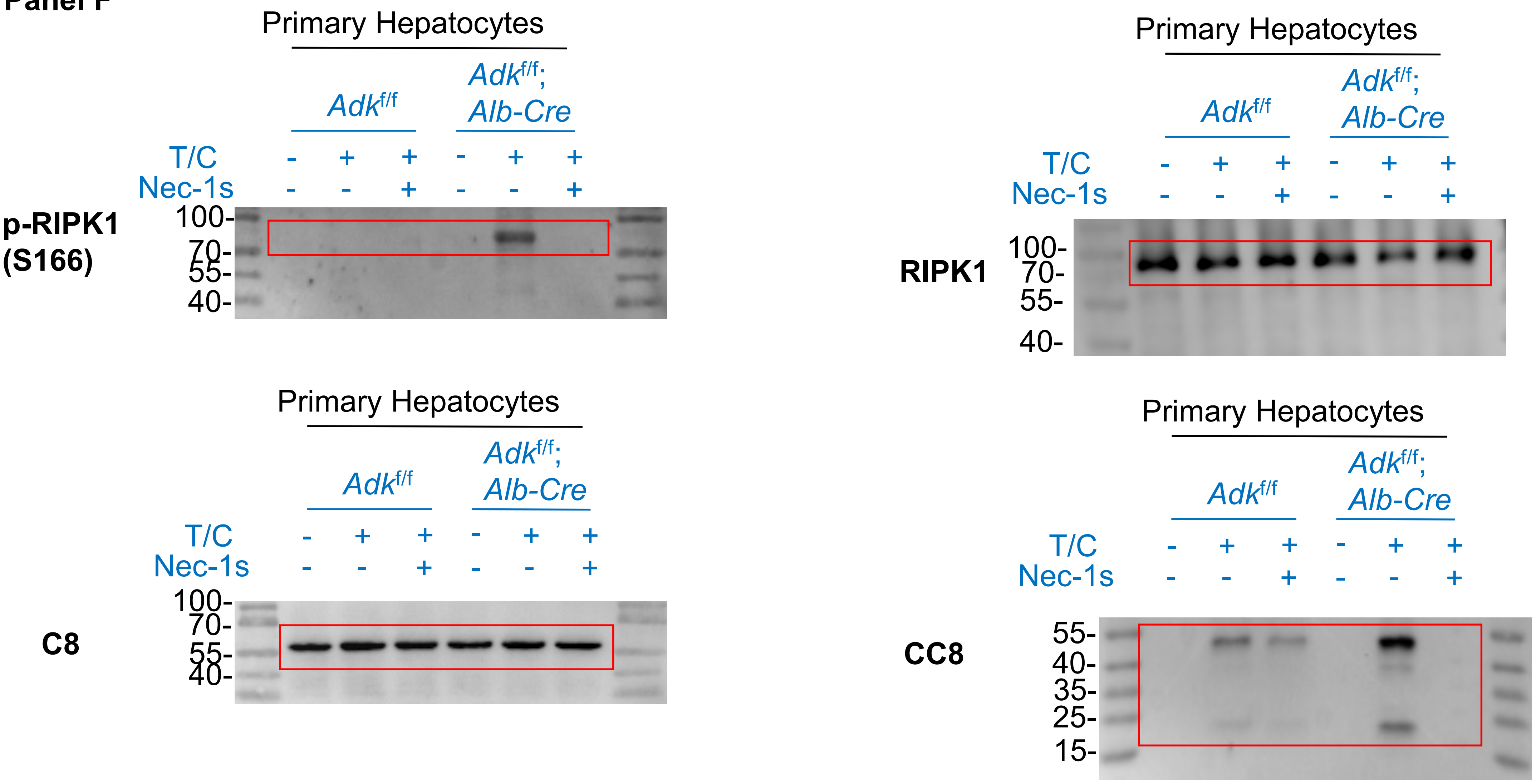

Uncropped blots_Figure 1

Panel F

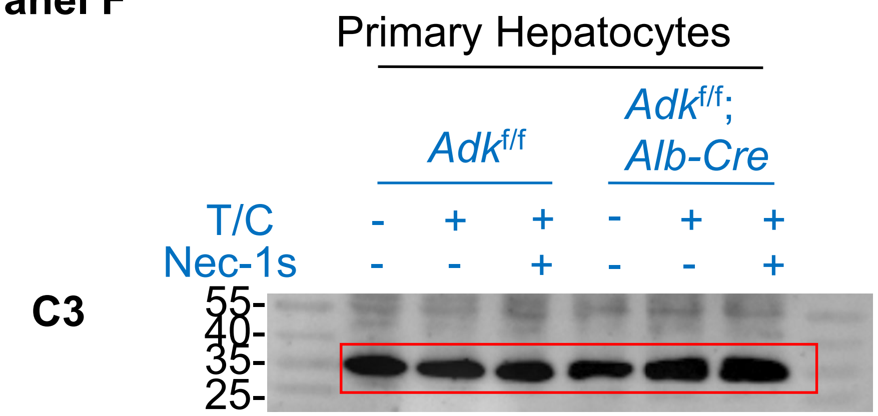

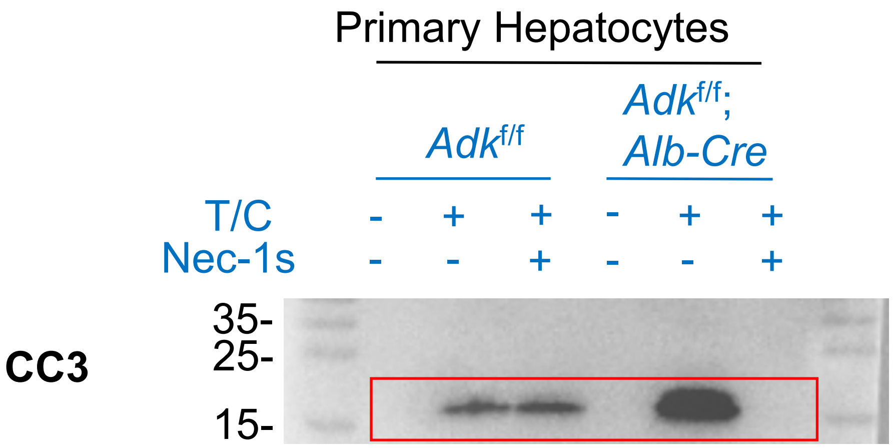

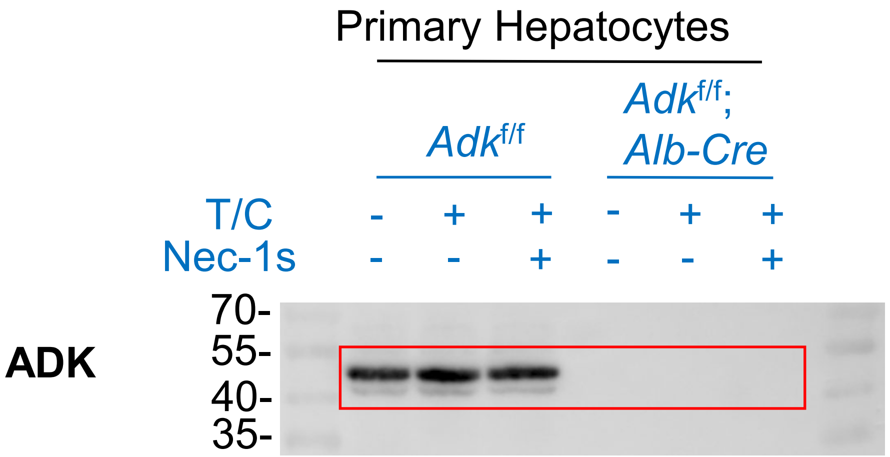

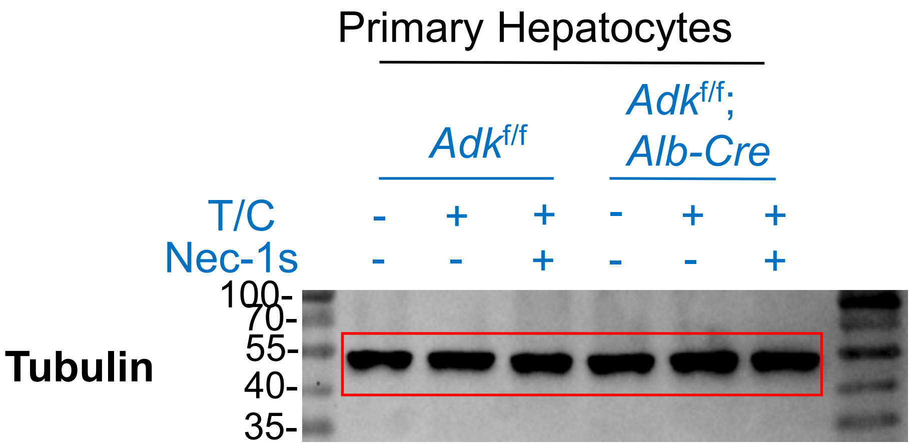

Uncropped blots_Figure 1

Panel H

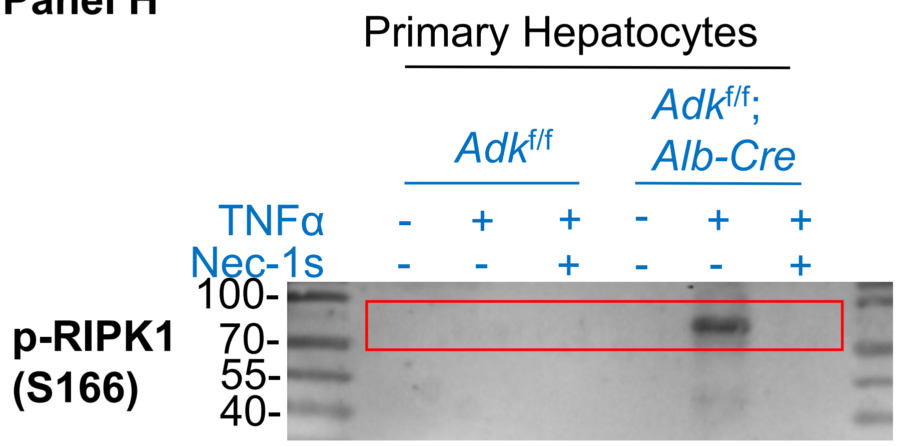

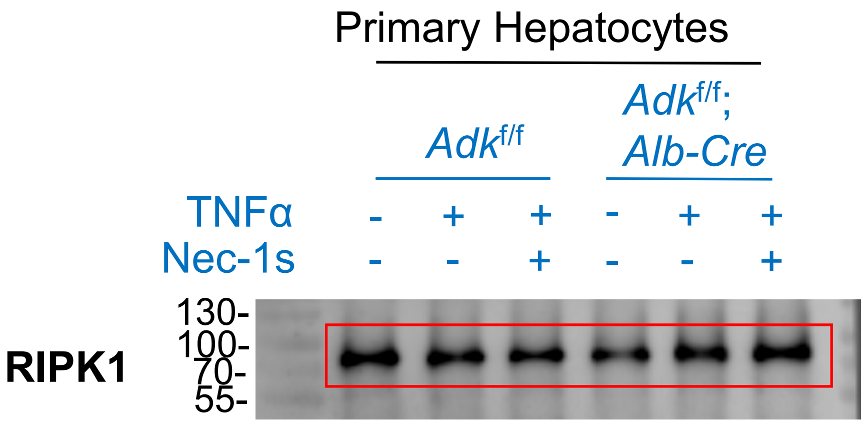

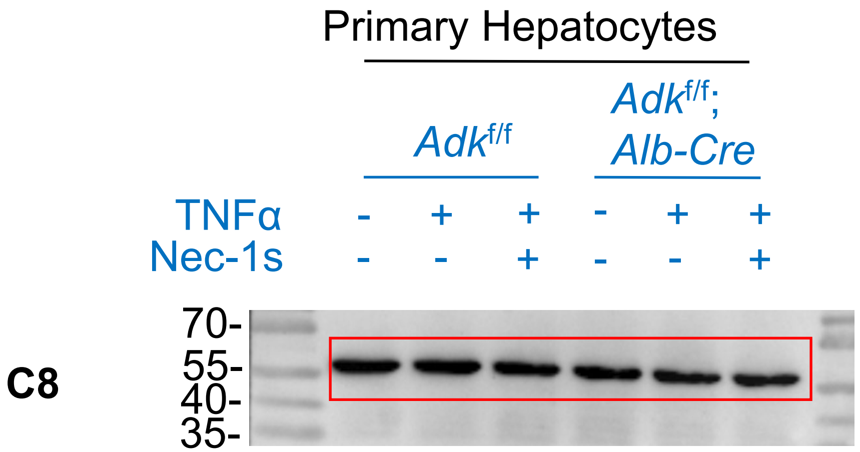

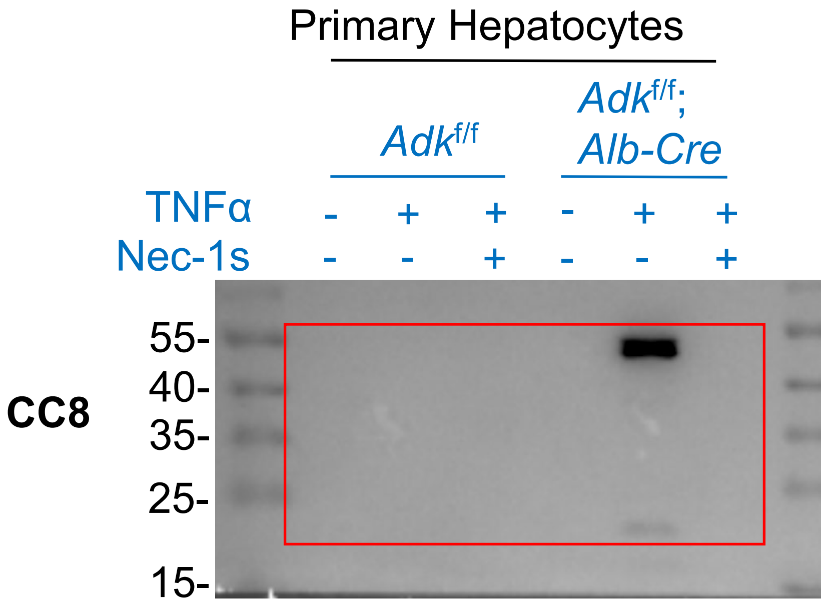

Uncropped blots_Figure 1

Panel H

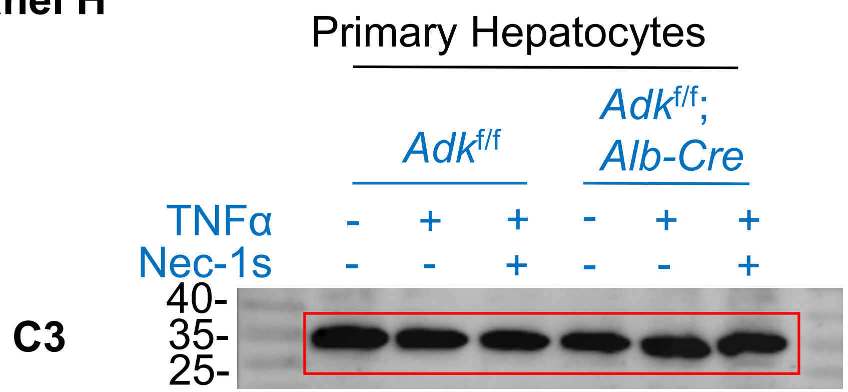

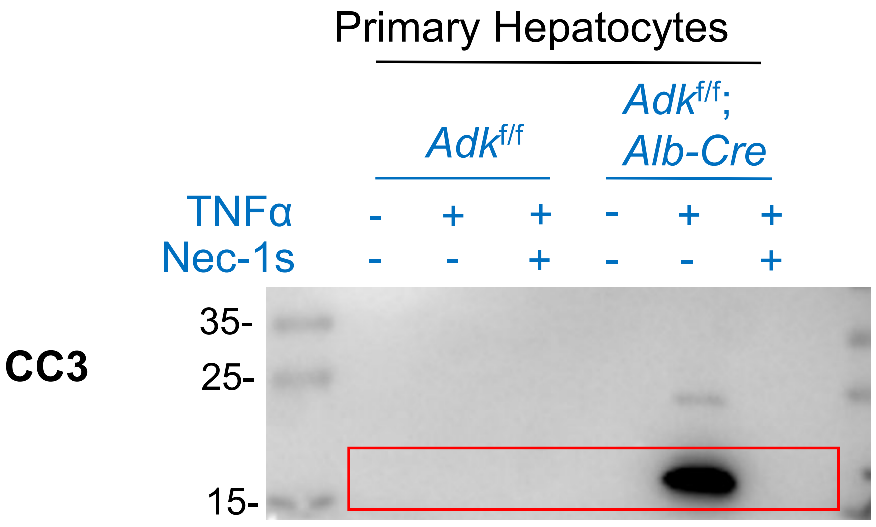

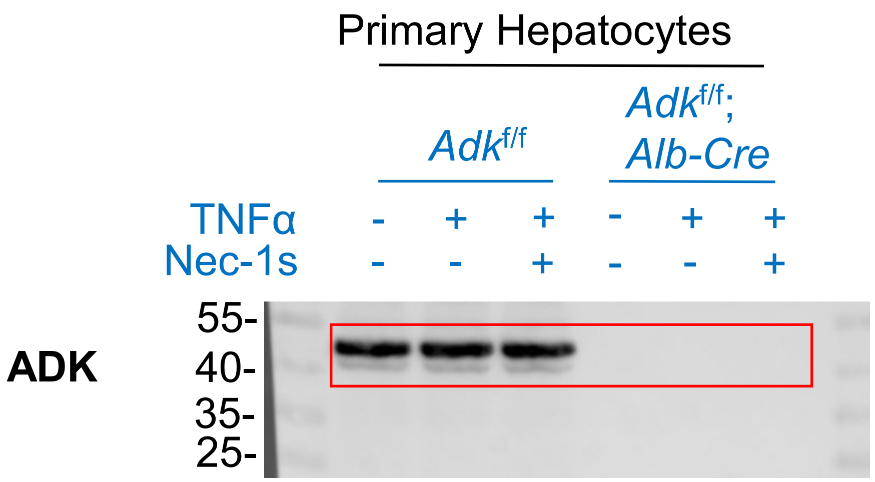

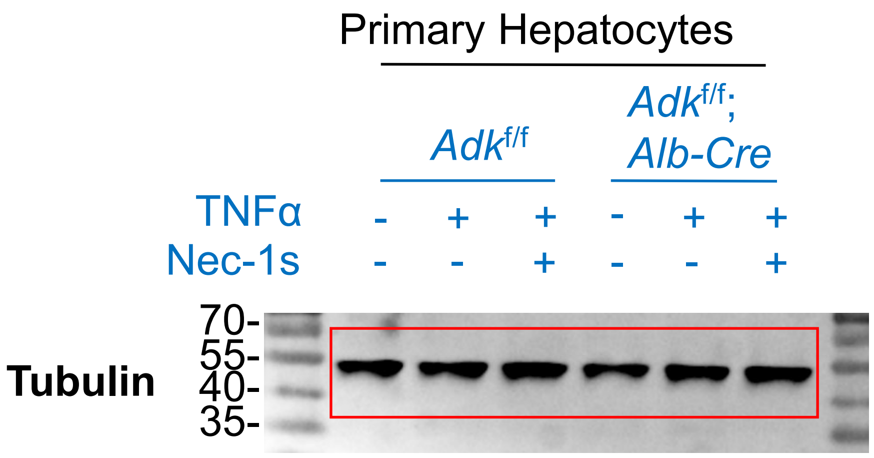

Uncropped blots_Figure 1

Panel J

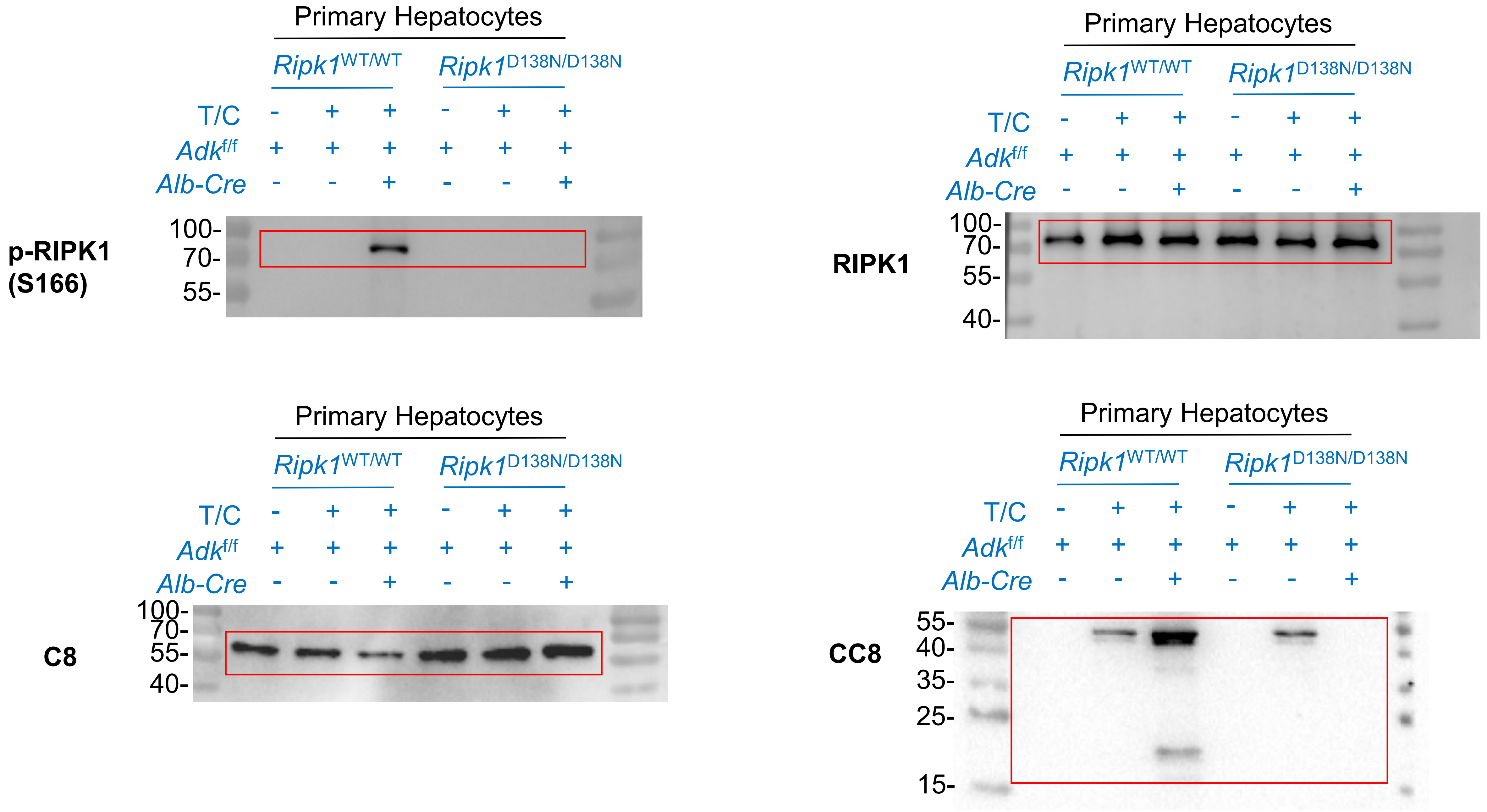

Uncropped blots_Figure 1

Panel J

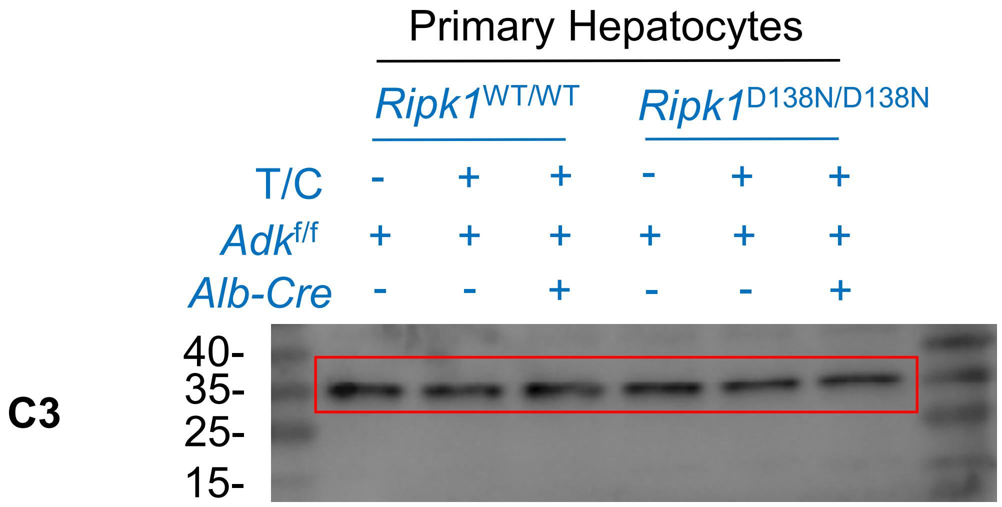

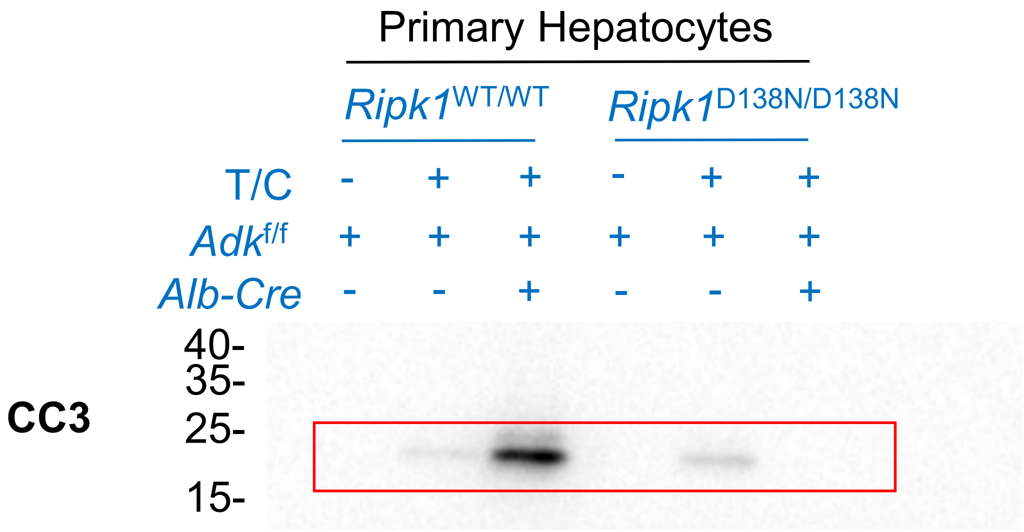

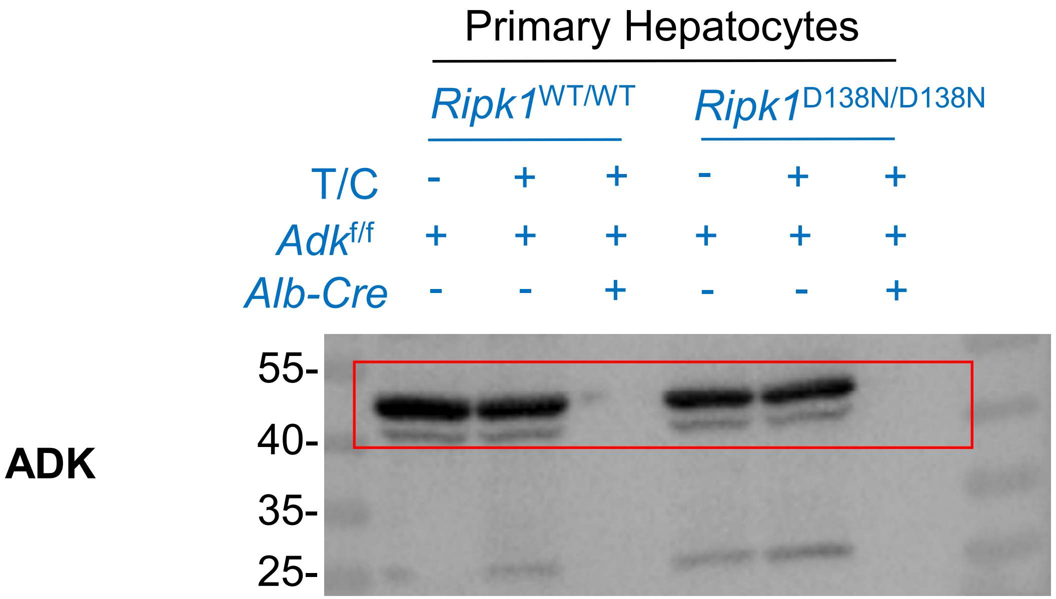

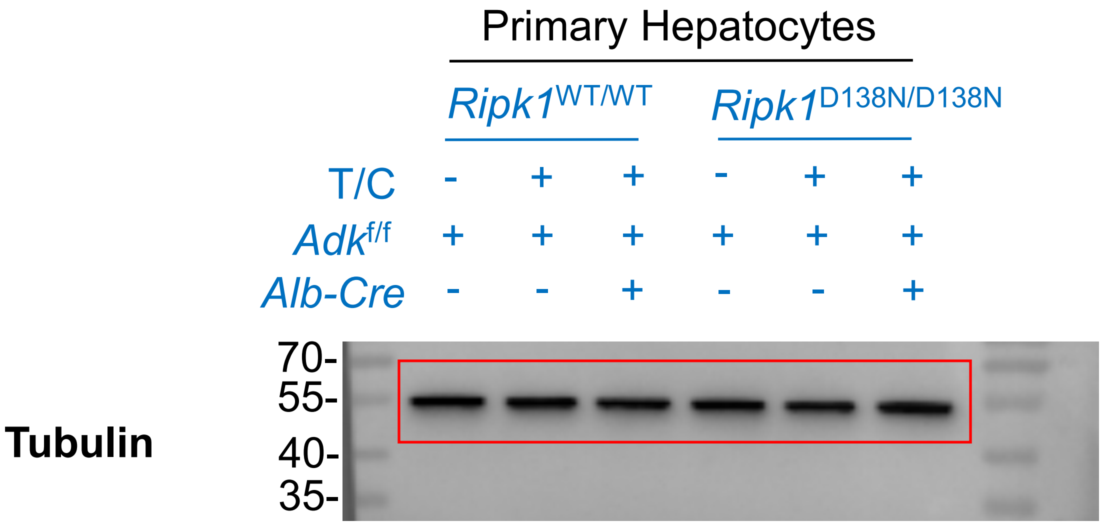

Uncropped blots_Figure 1

Panel L

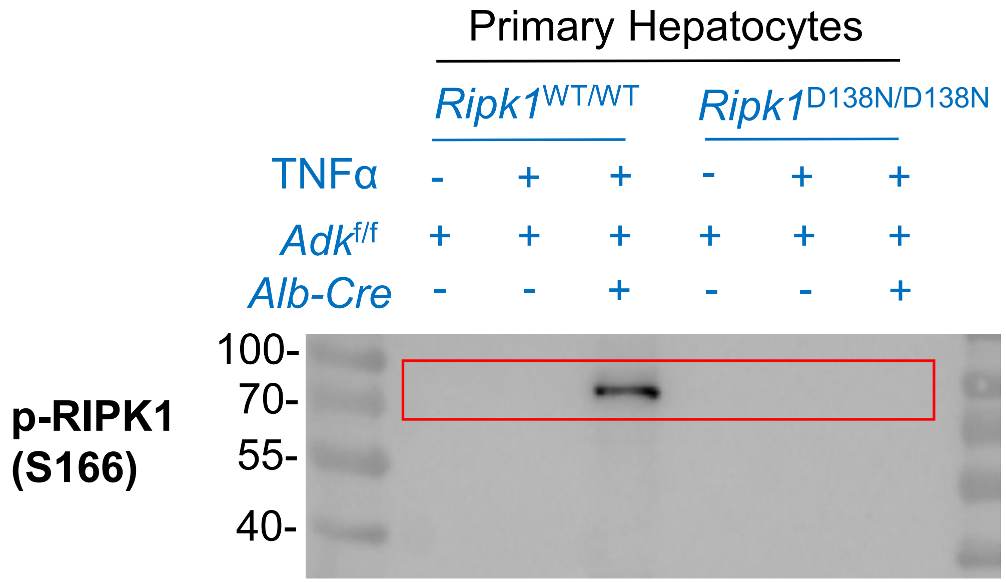

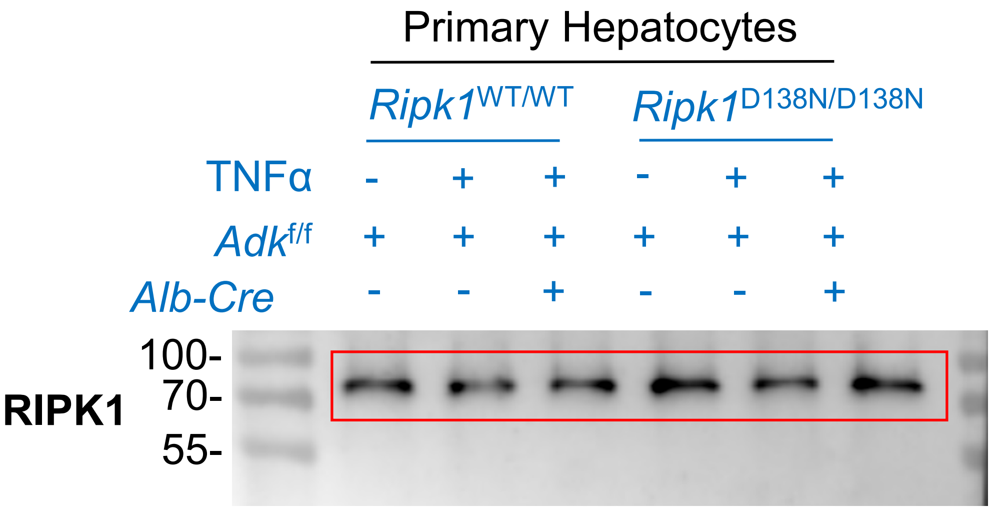

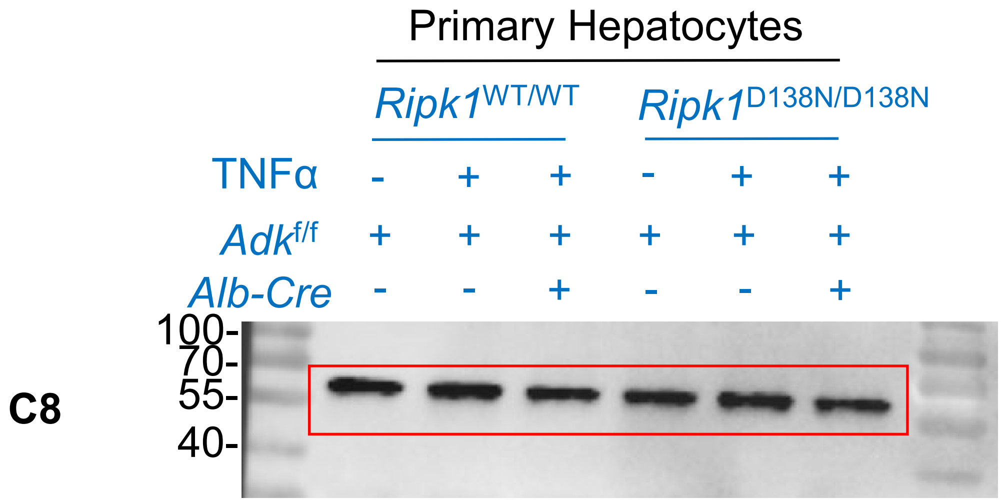

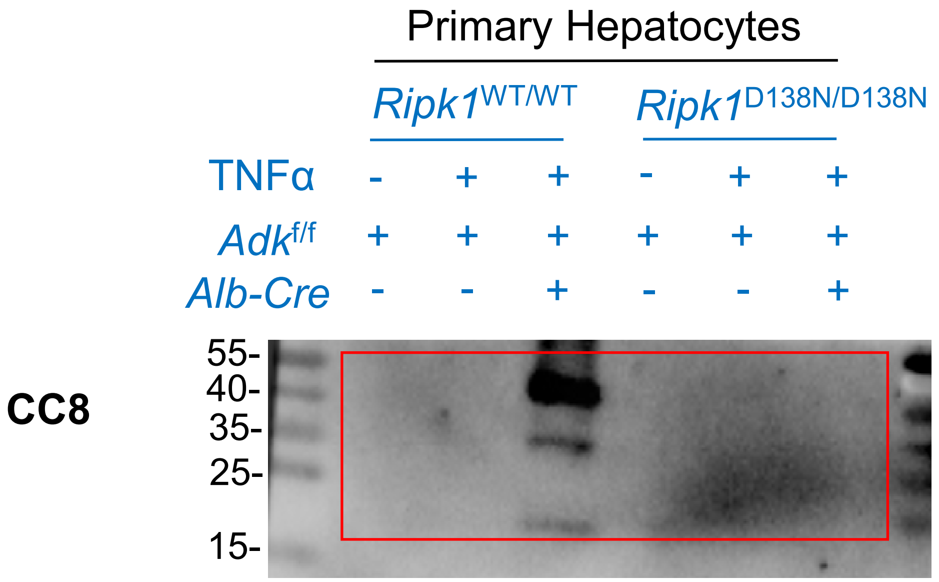

Uncropped blots_Figure 1

Panel L

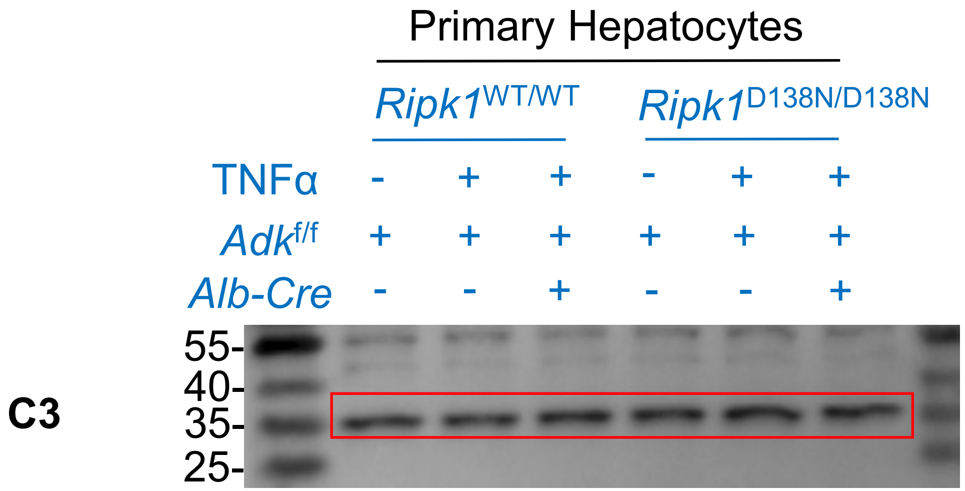

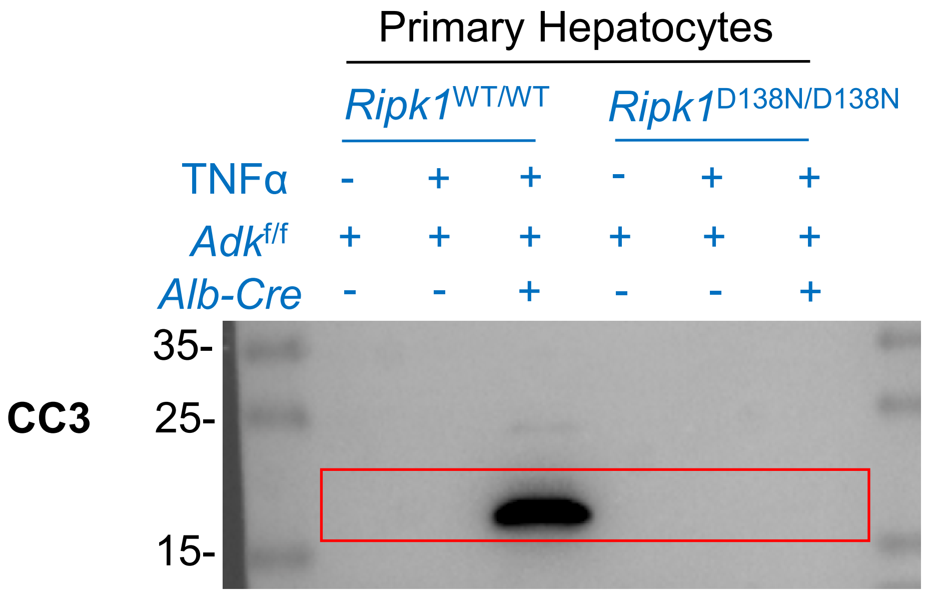

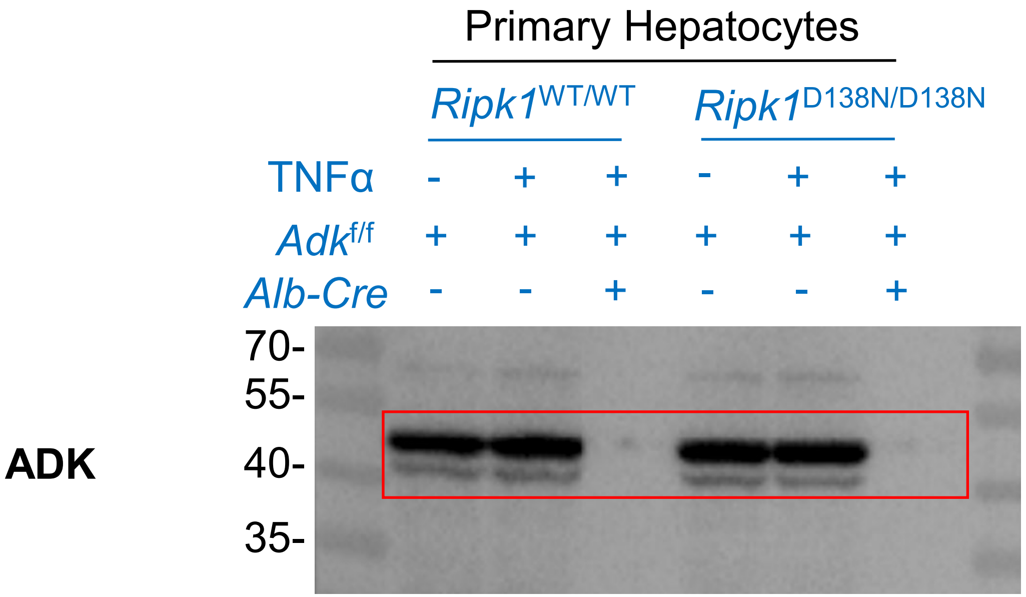

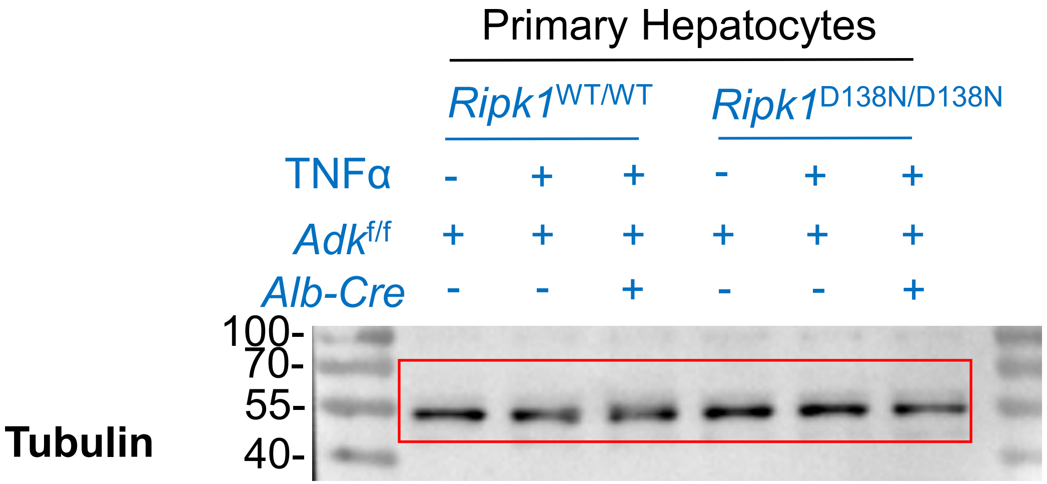

Uncropped blots_Figure 1

Panel M

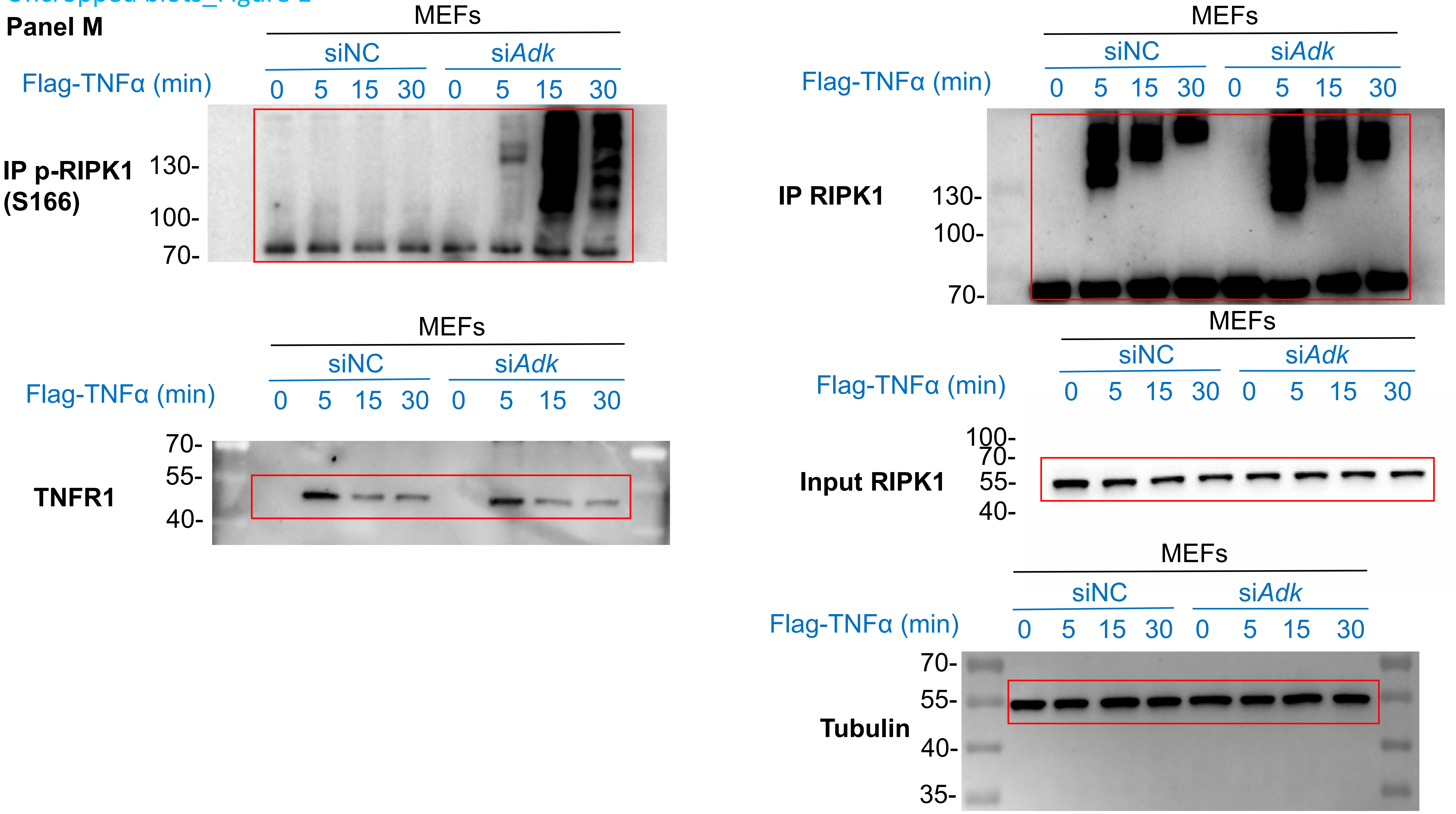

Uncropped blots_Figure 1

Panel N

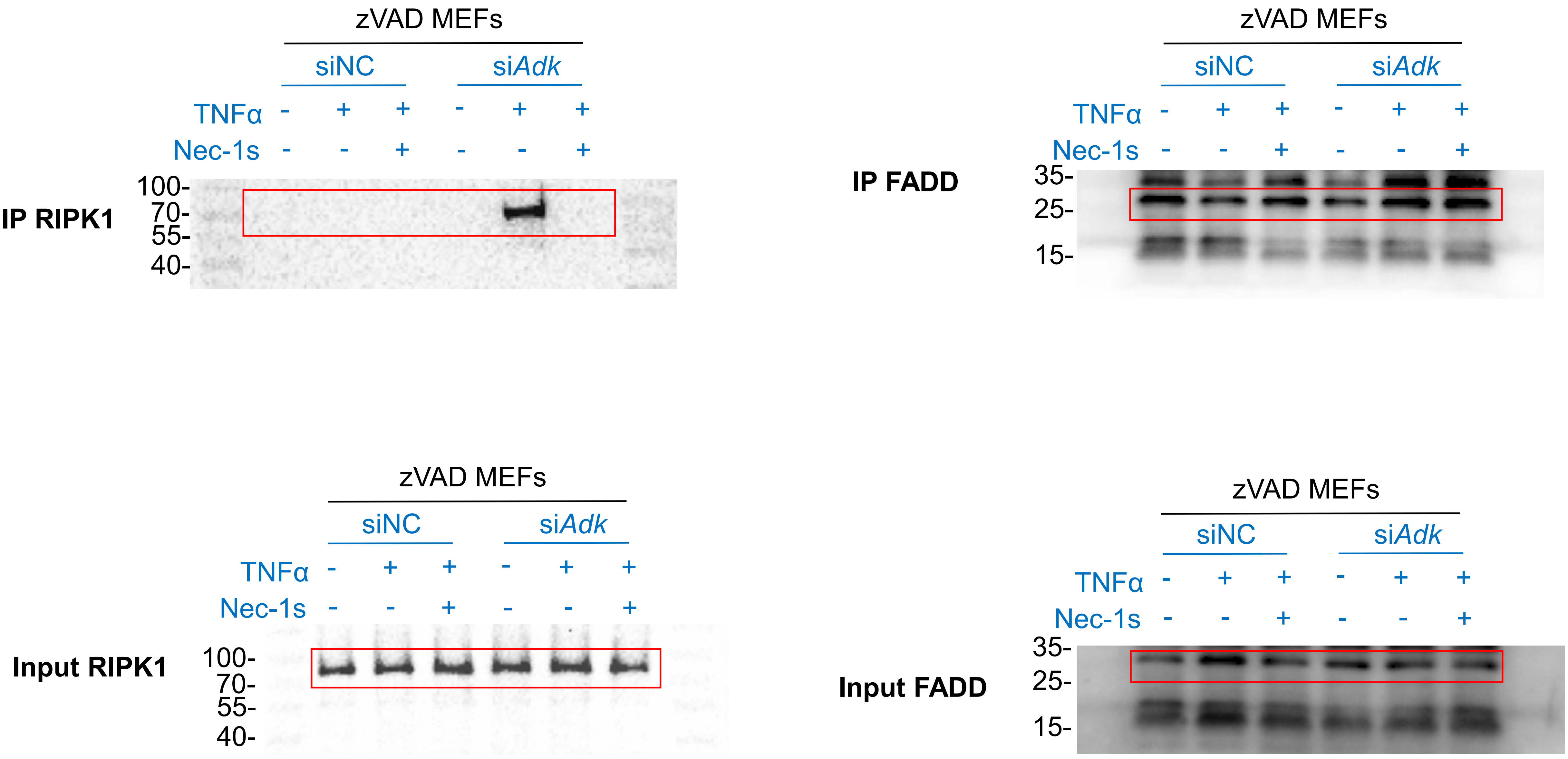

Uncropped blots_Figure 1

Panel N

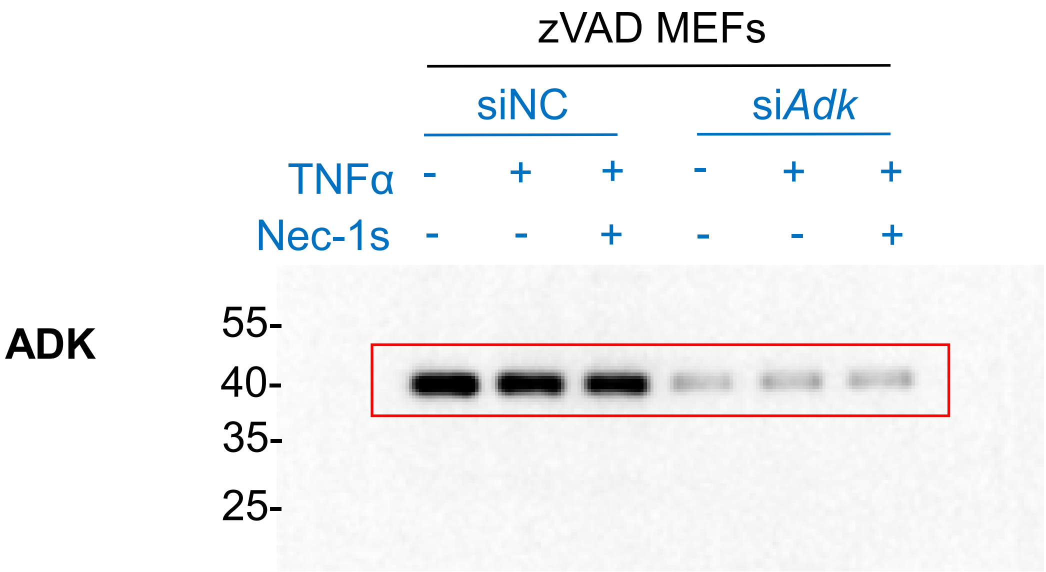

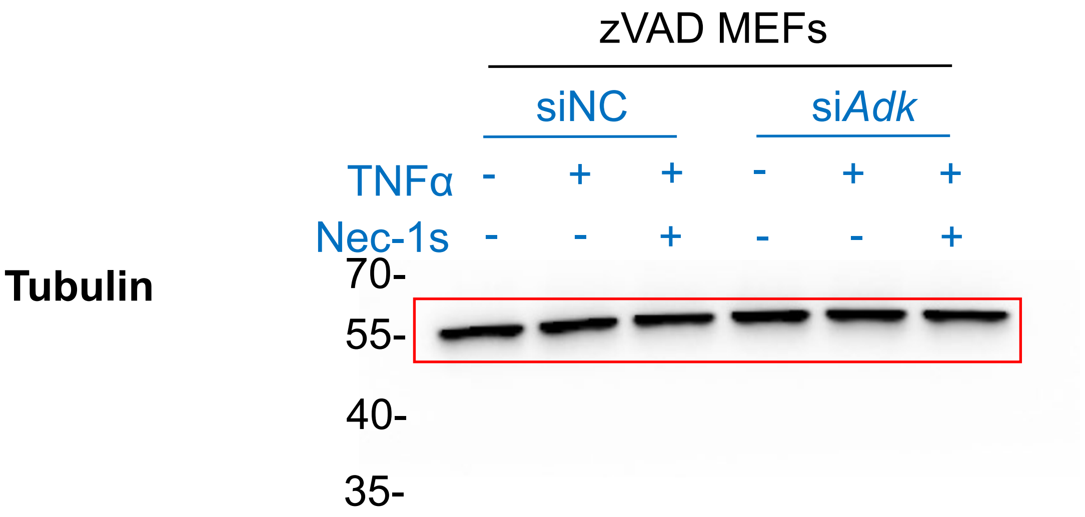

Uncropped blots_Figure 1

Panel O

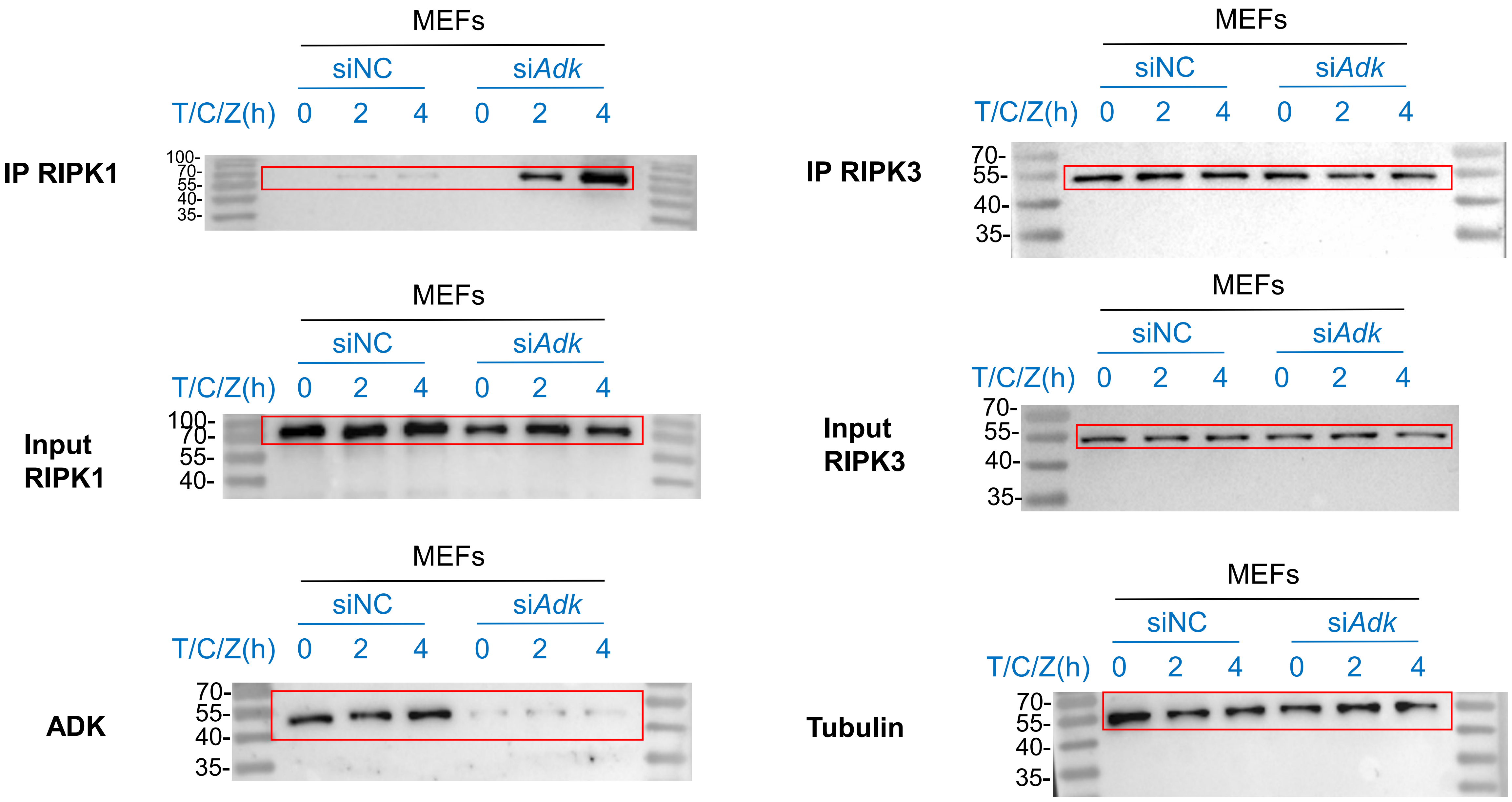

Supplement: SourceData F1 — is the source file for Fig. 1. [file jem_20250603_sourcedataf1.pdf]
