# Supplementary material for: Adenosine metabolic clearance maintains liver homeostasis by licensing arginine methylation of RIPK1
Source: J Exp Med. 2025 Oct 13;223(1):e20250603. doi: 10.1084/jem.20250603 (PMC12517274; doi:10.1084/jem.20250603)

Uncropped blots_Figure 2

Panel D

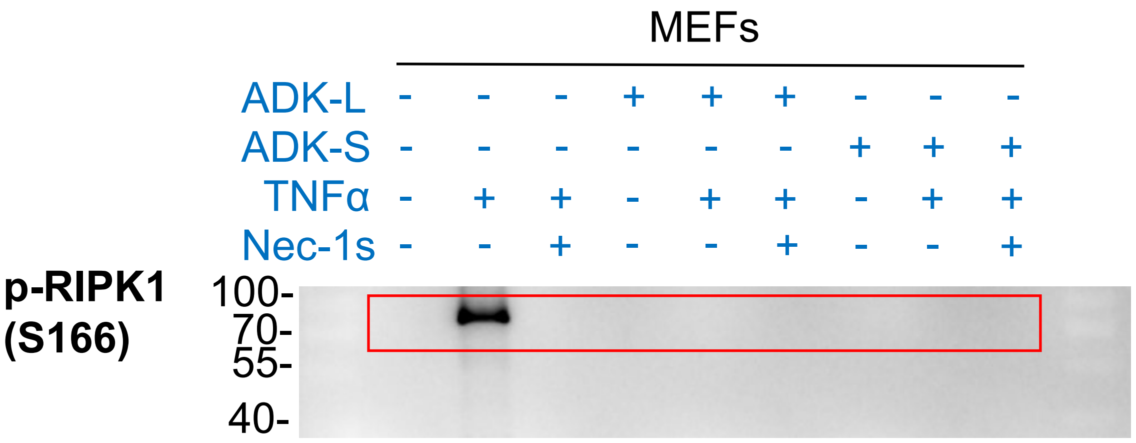

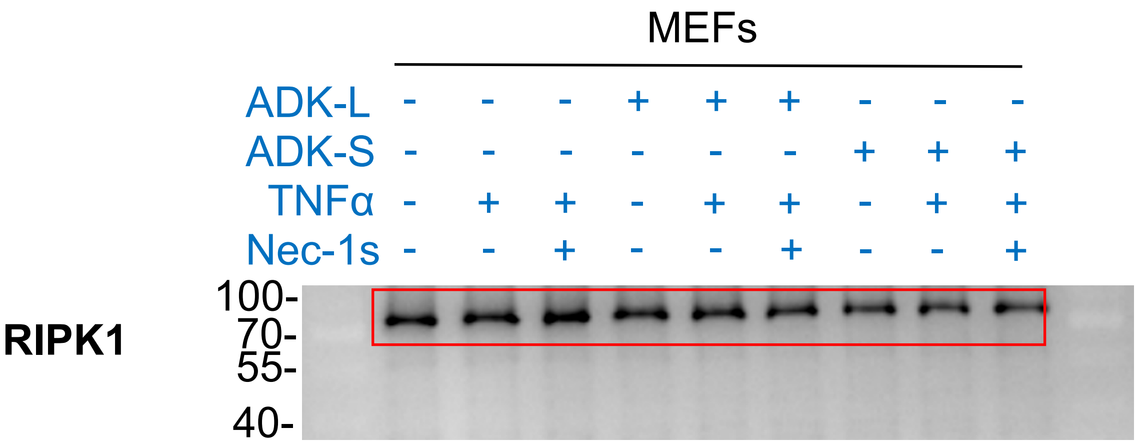

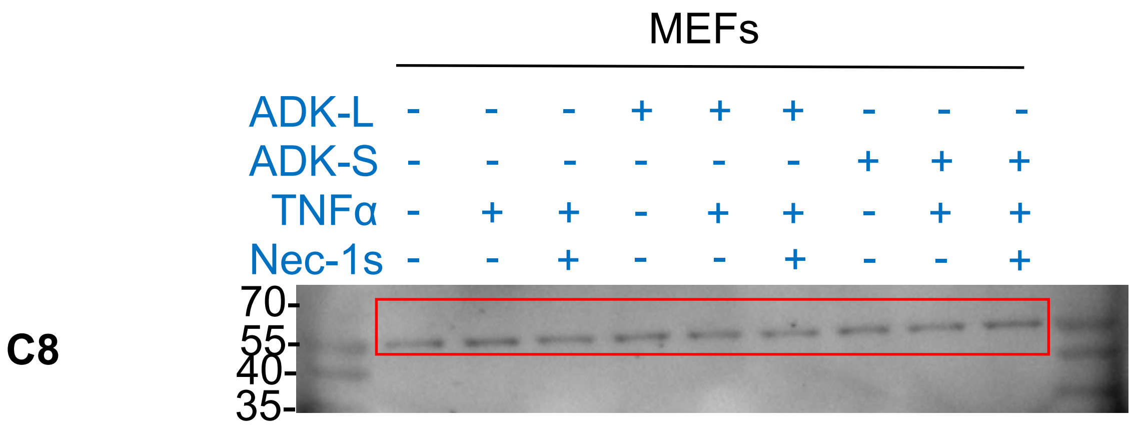

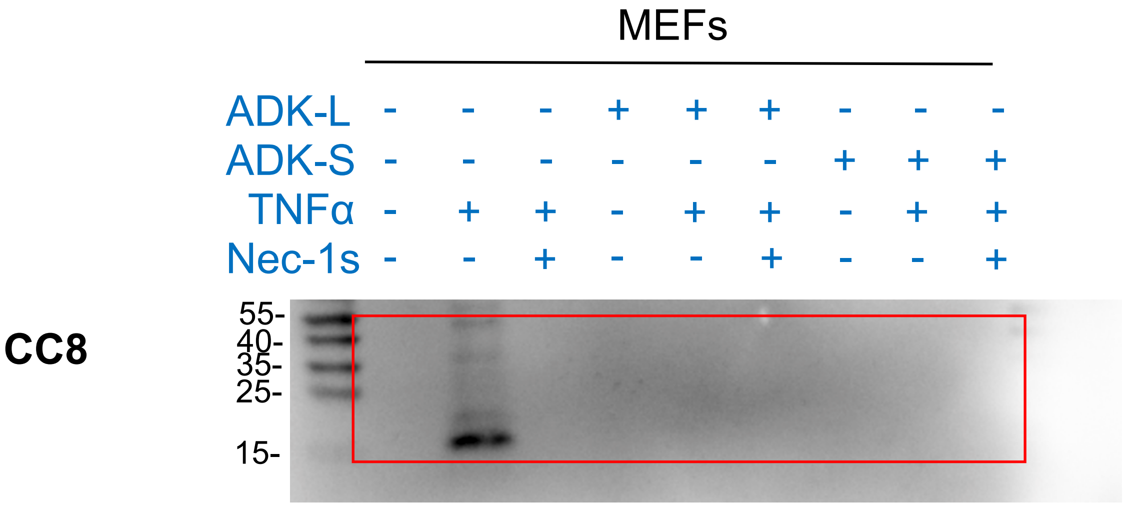

Uncropped blots_Figure 2

Panel D

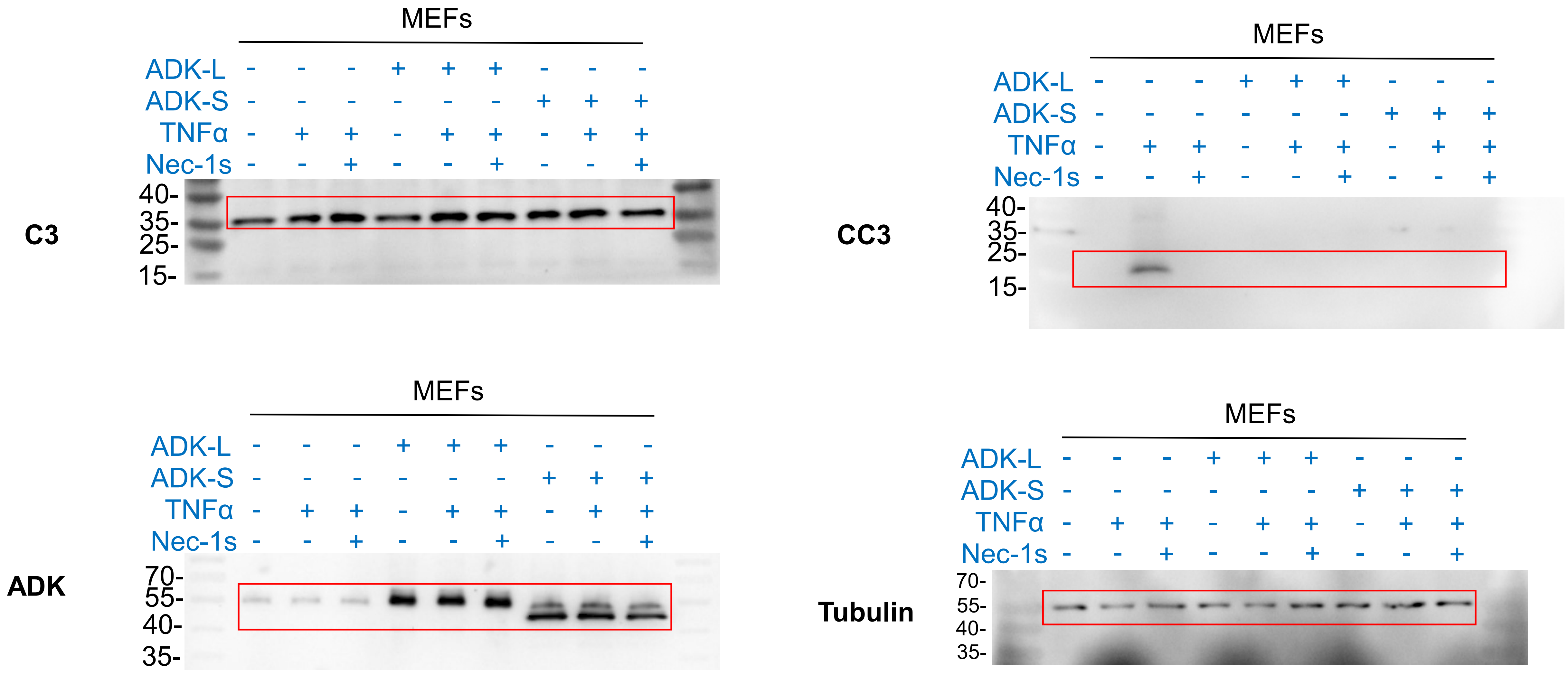

Uncropped blots_Figure 2

Panel H

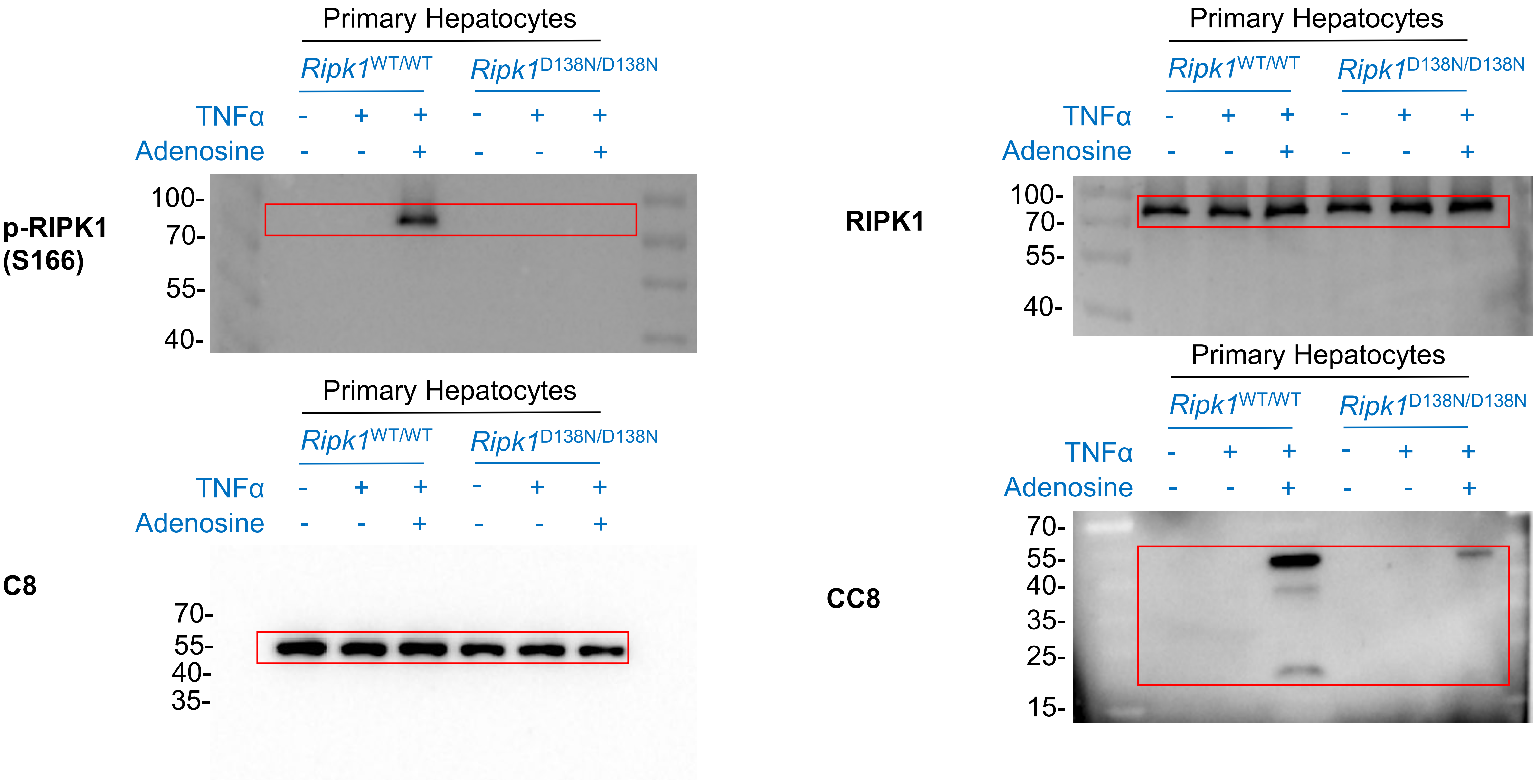

Uncropped blots_Figure 2

Panel H

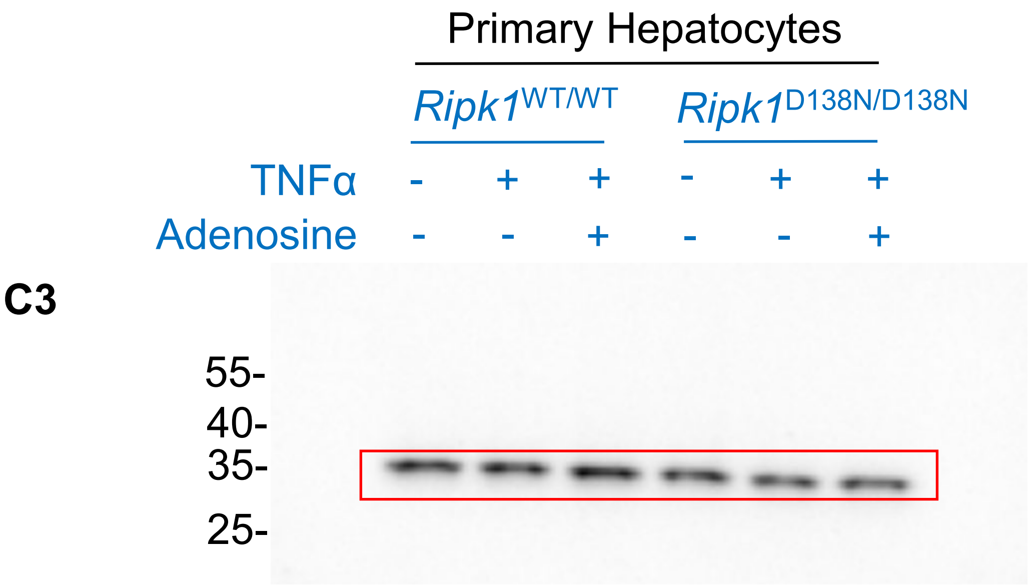

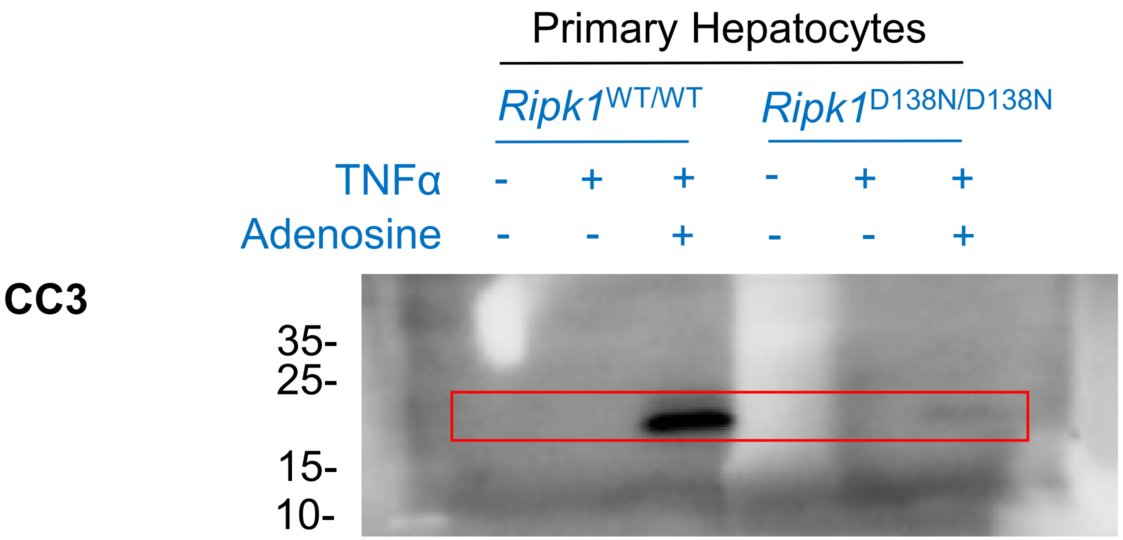

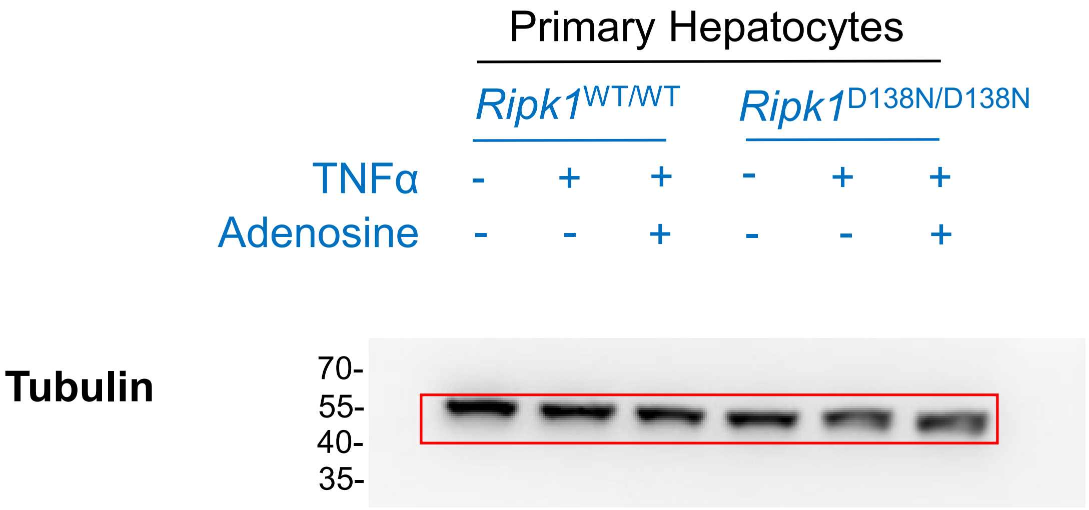

Uncropped blots_Figure 2

Panel J

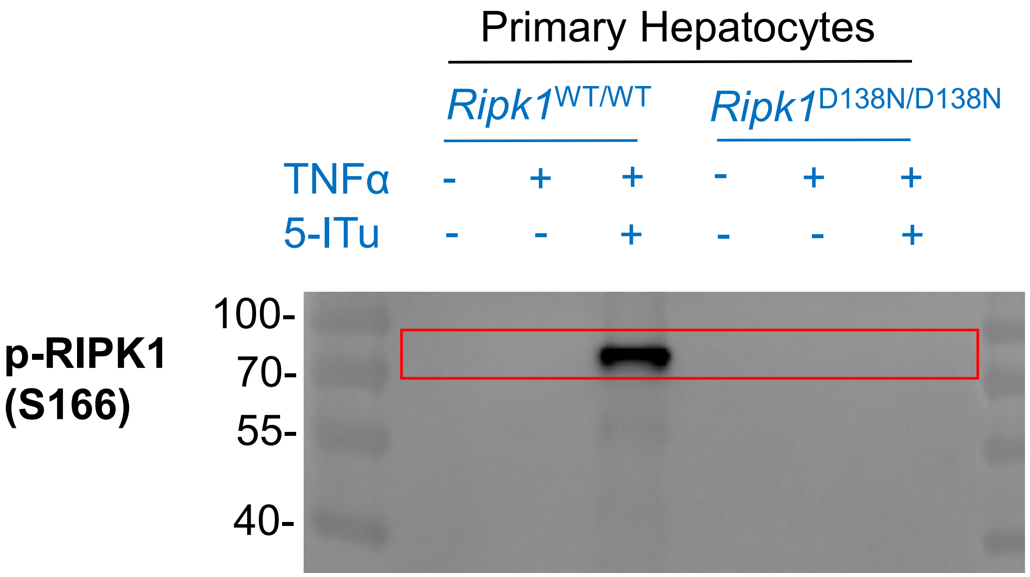

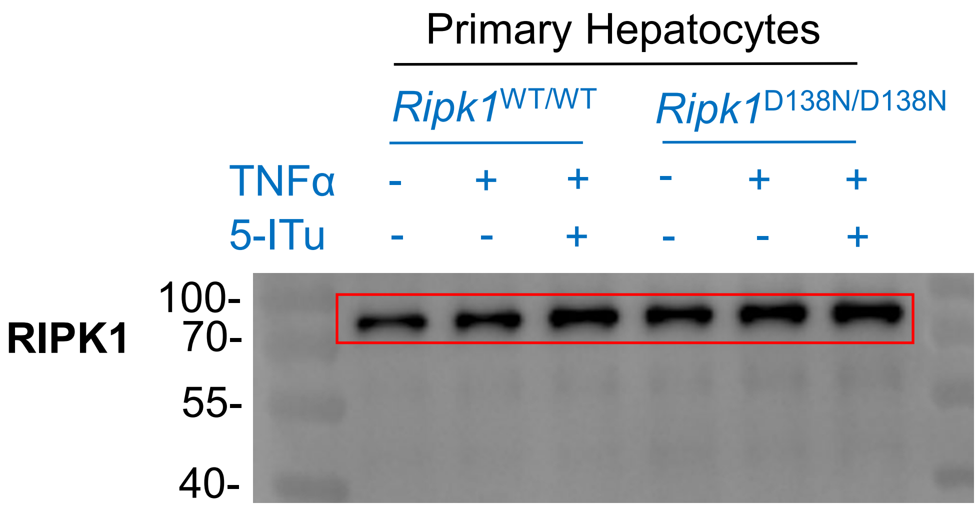

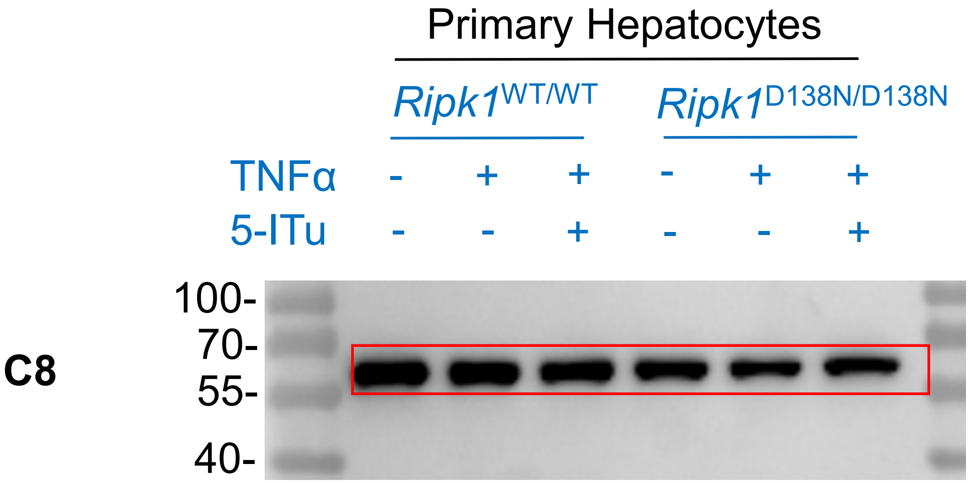

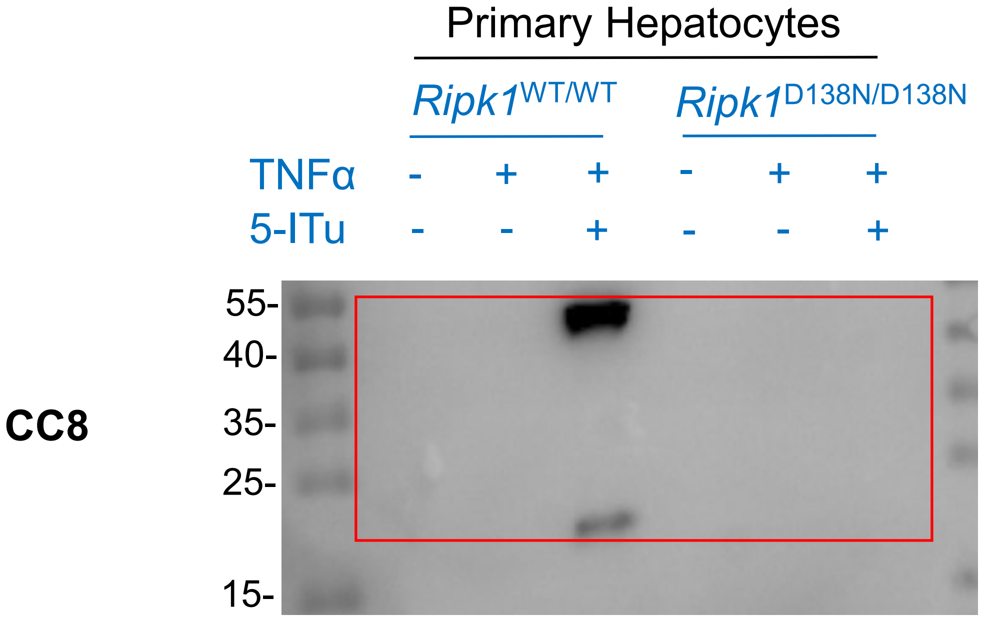

Uncropped blots_Figure 2

Panel J

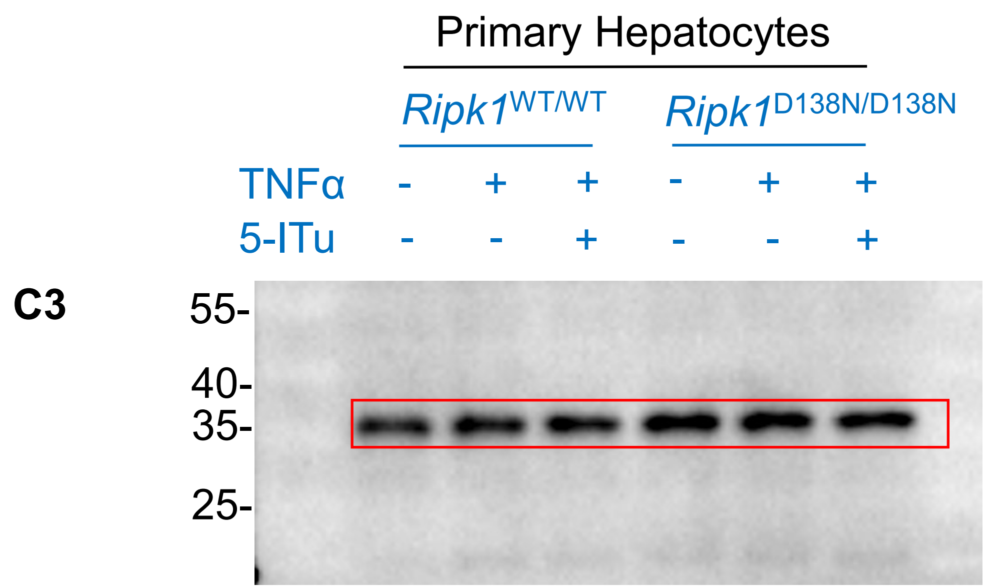

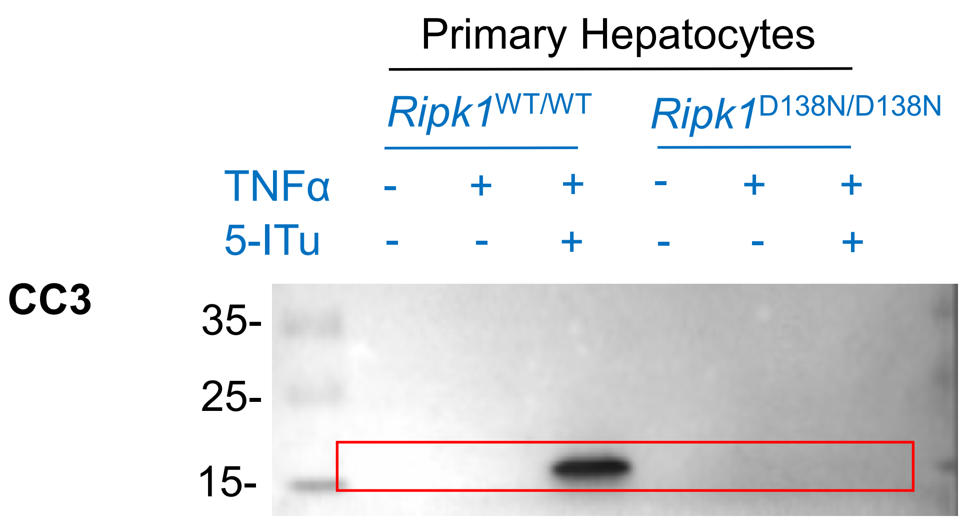

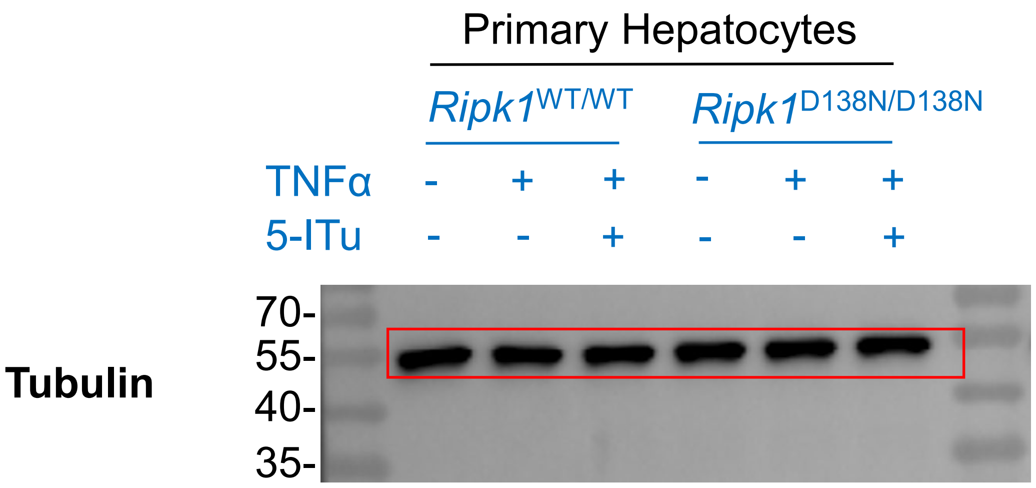

Uncropped blots_Figure 2

Panel L

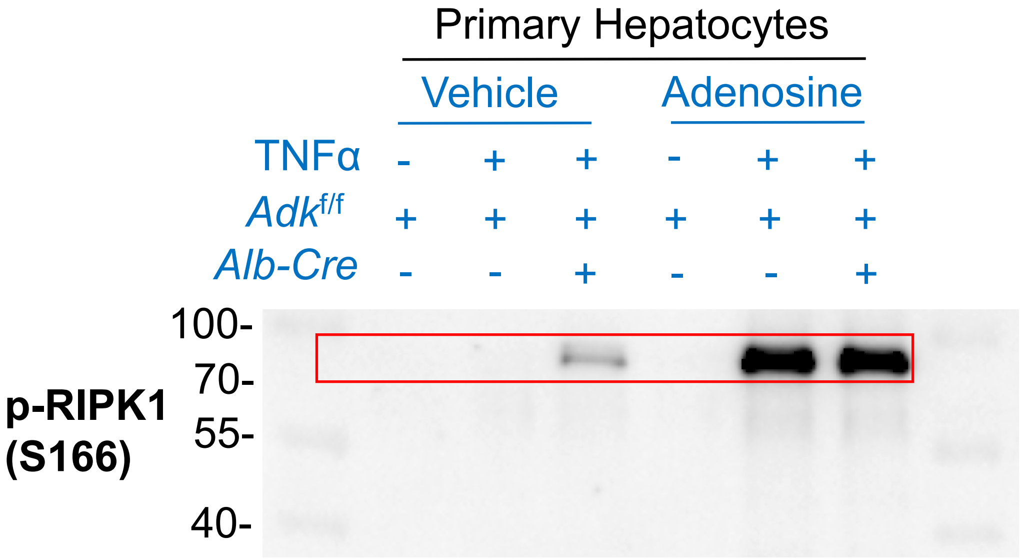

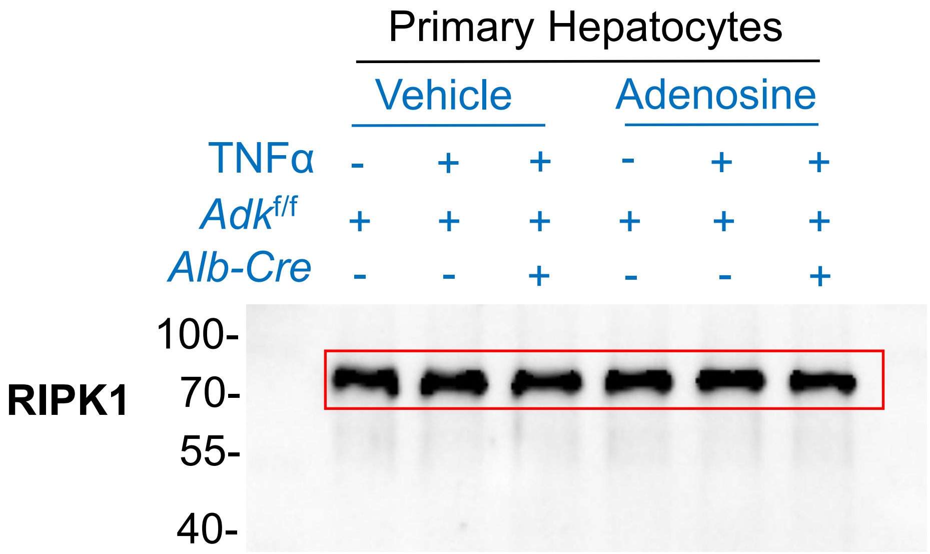

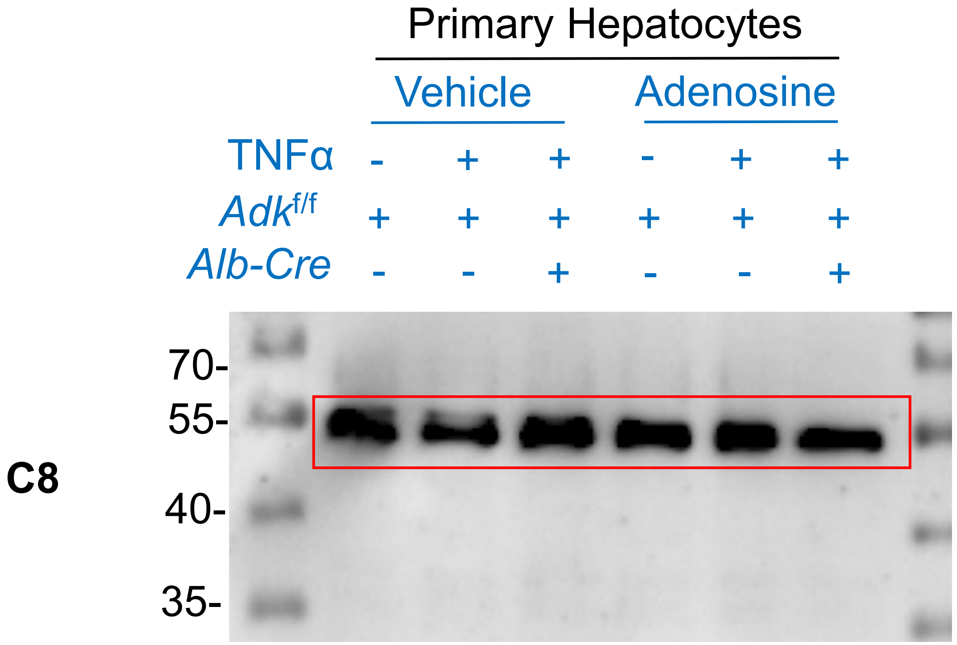

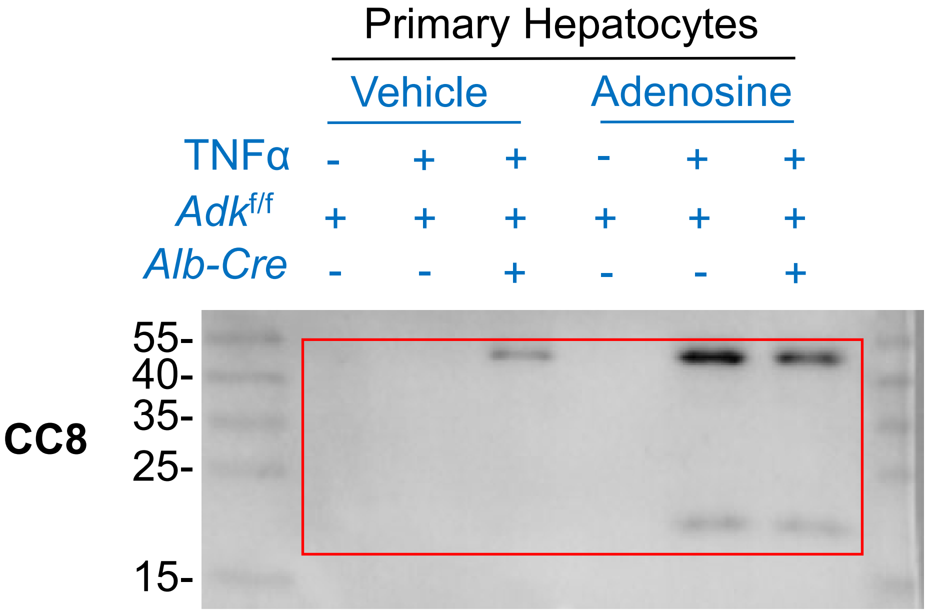

Uncropped blots_Figure 2

Panel L

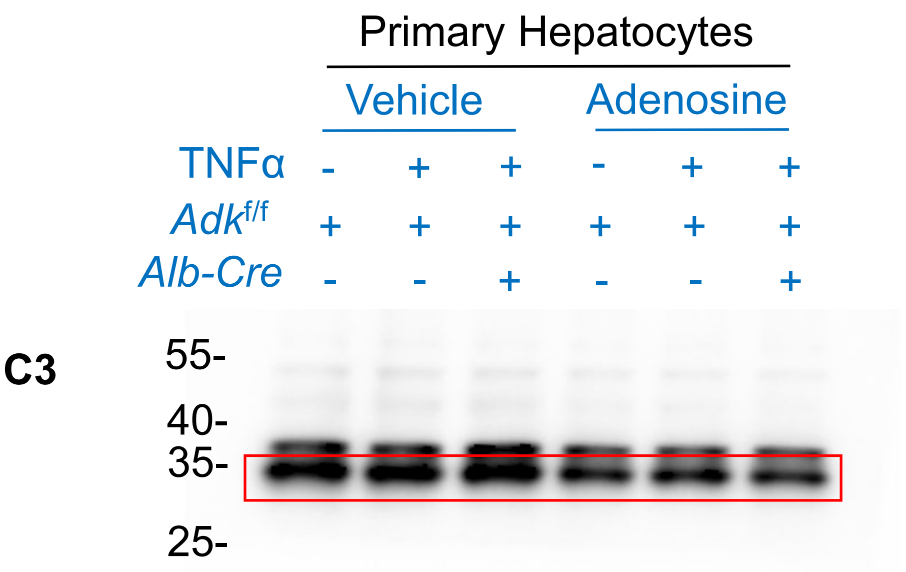

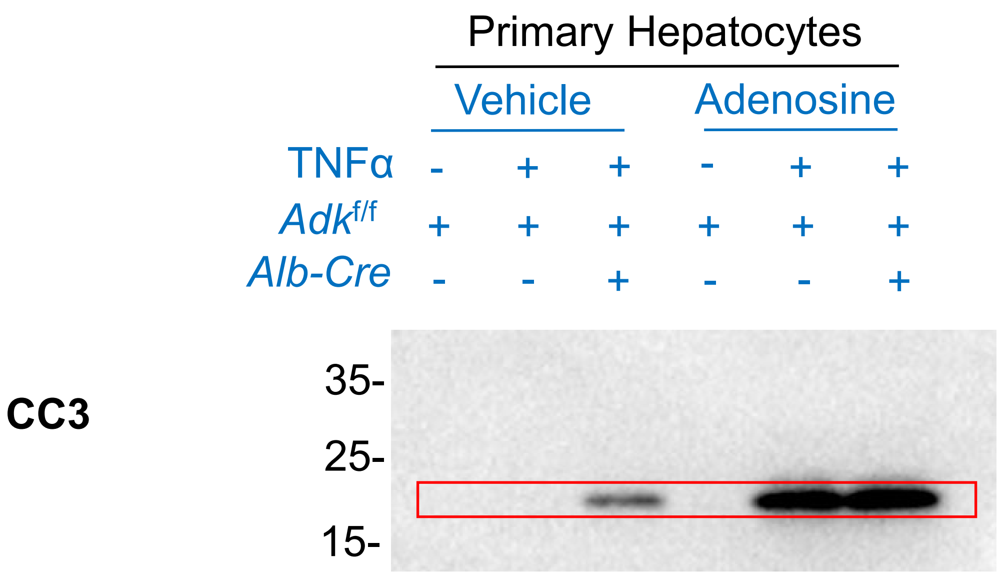

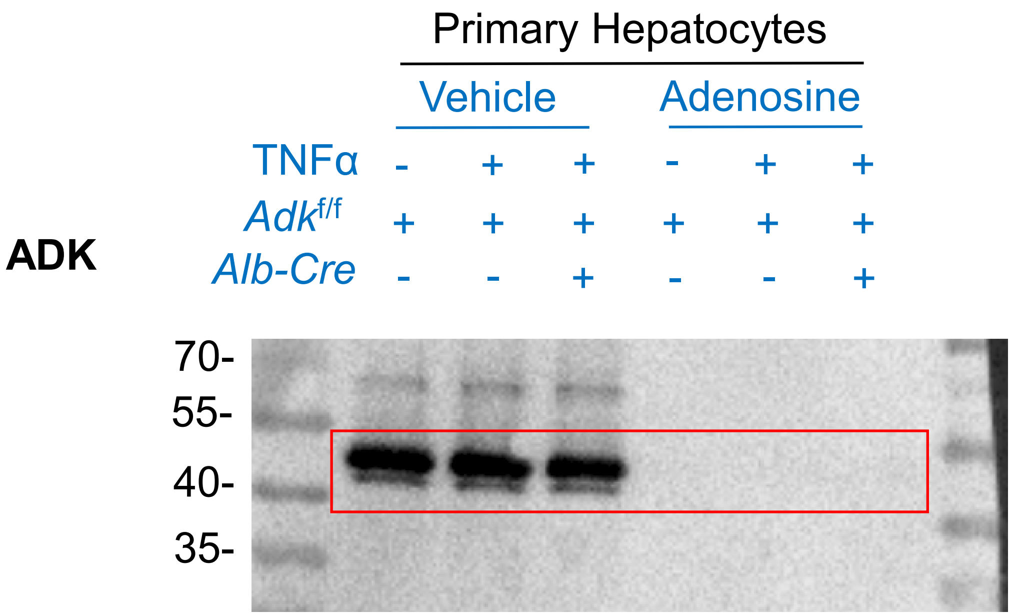

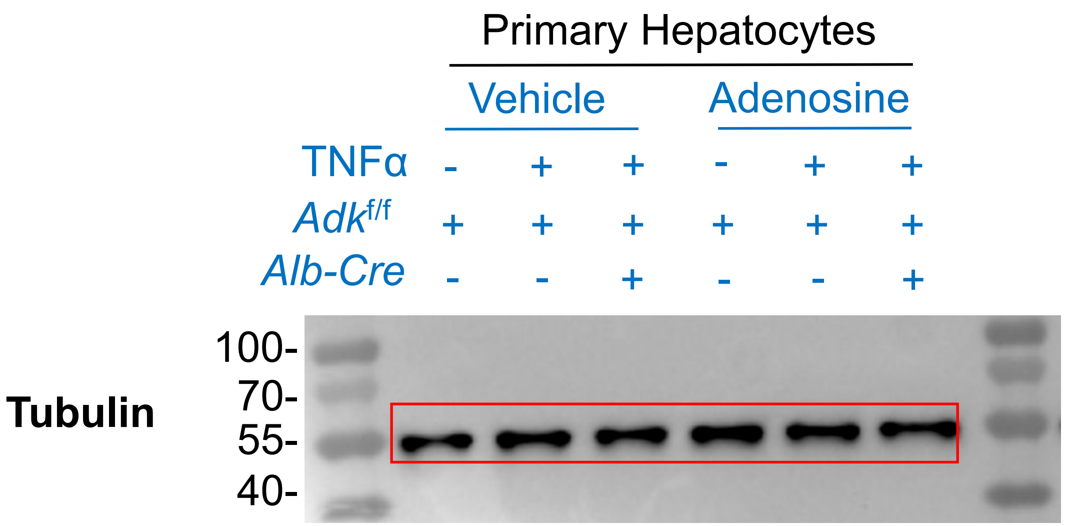

Uncropped blots_Figure 2

Panel P

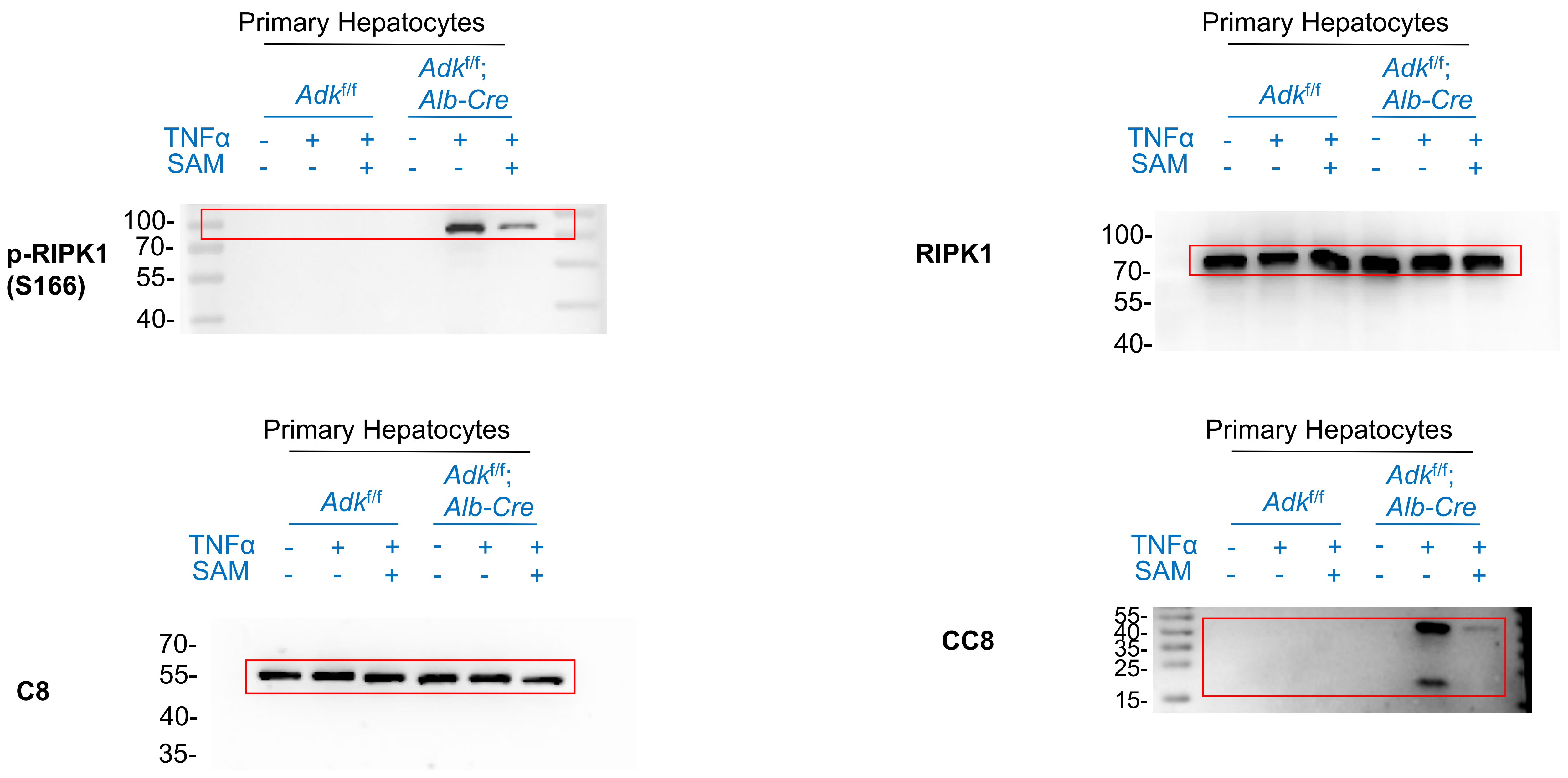

Uncropped blots_Figure 2

Panel P

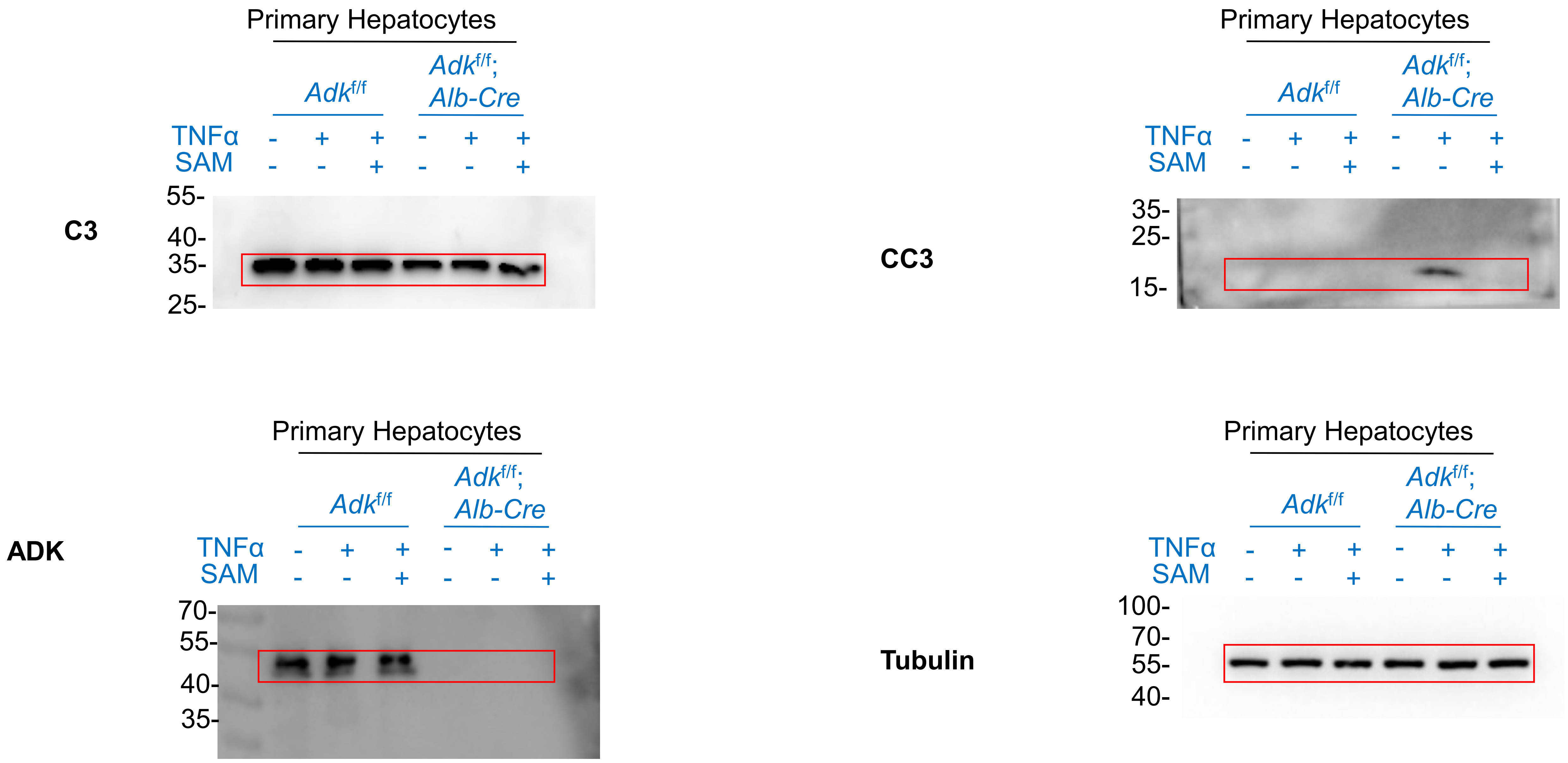

Supplement: SourceData F2 — is the source file for Fig. 2. [file jem_20250603_sourcedataf2.pdf]
