# Supplementary material for: Adenosine metabolic clearance maintains liver homeostasis by licensing arginine methylation of RIPK1
Source: J Exp Med. 2025 Oct 13;223(1):e20250603. doi: 10.1084/jem.20250603 (PMC12517274; doi:10.1084/jem.20250603)

Uncropped blots_Figure 3

Panel A

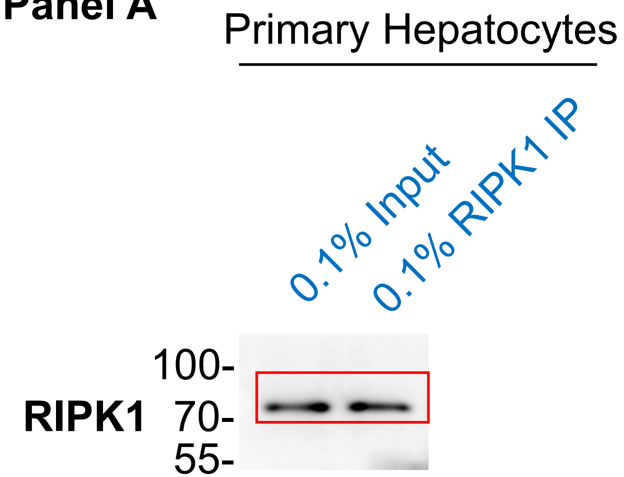

Panel C

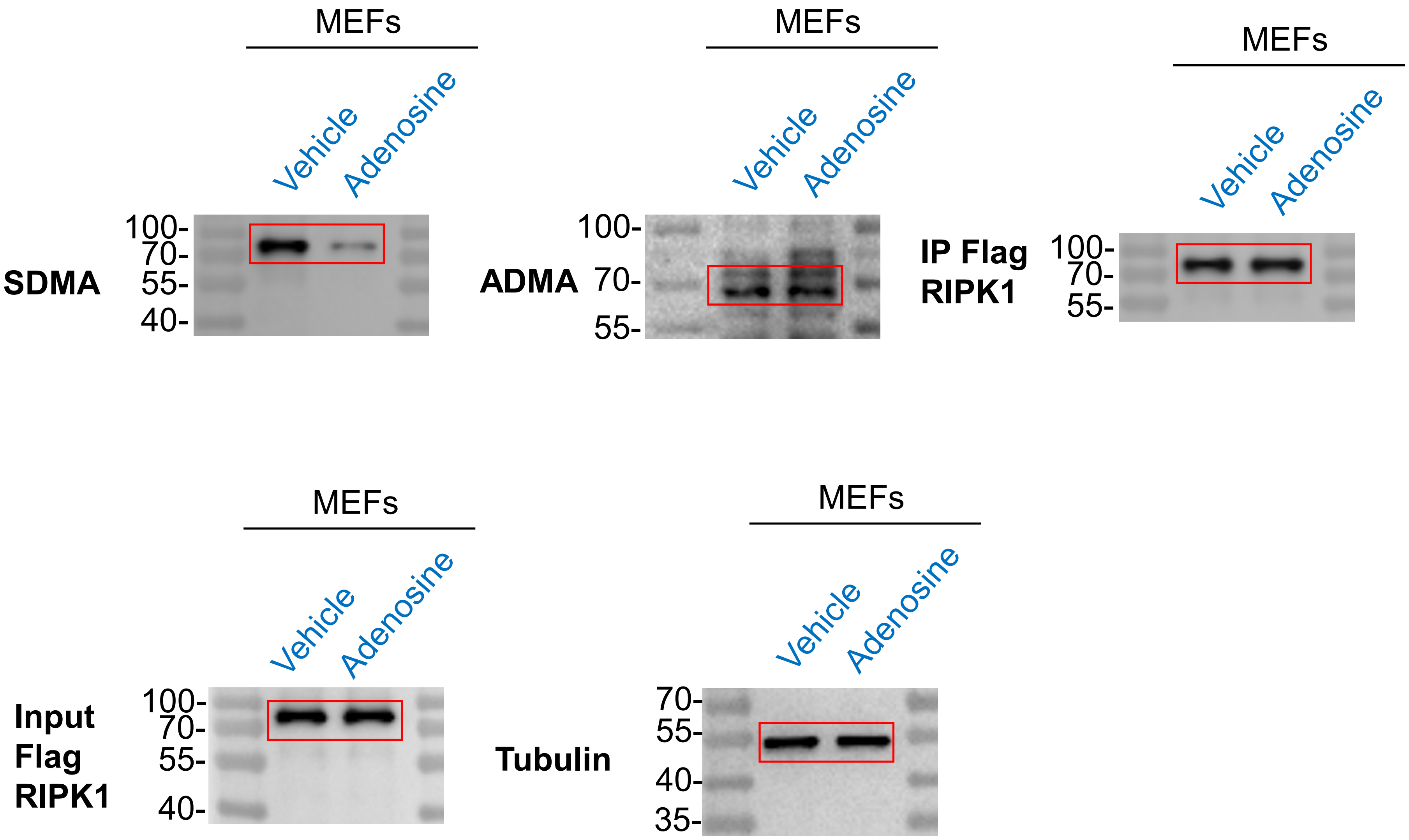

Uncropped blots_Figure 3

Panel D

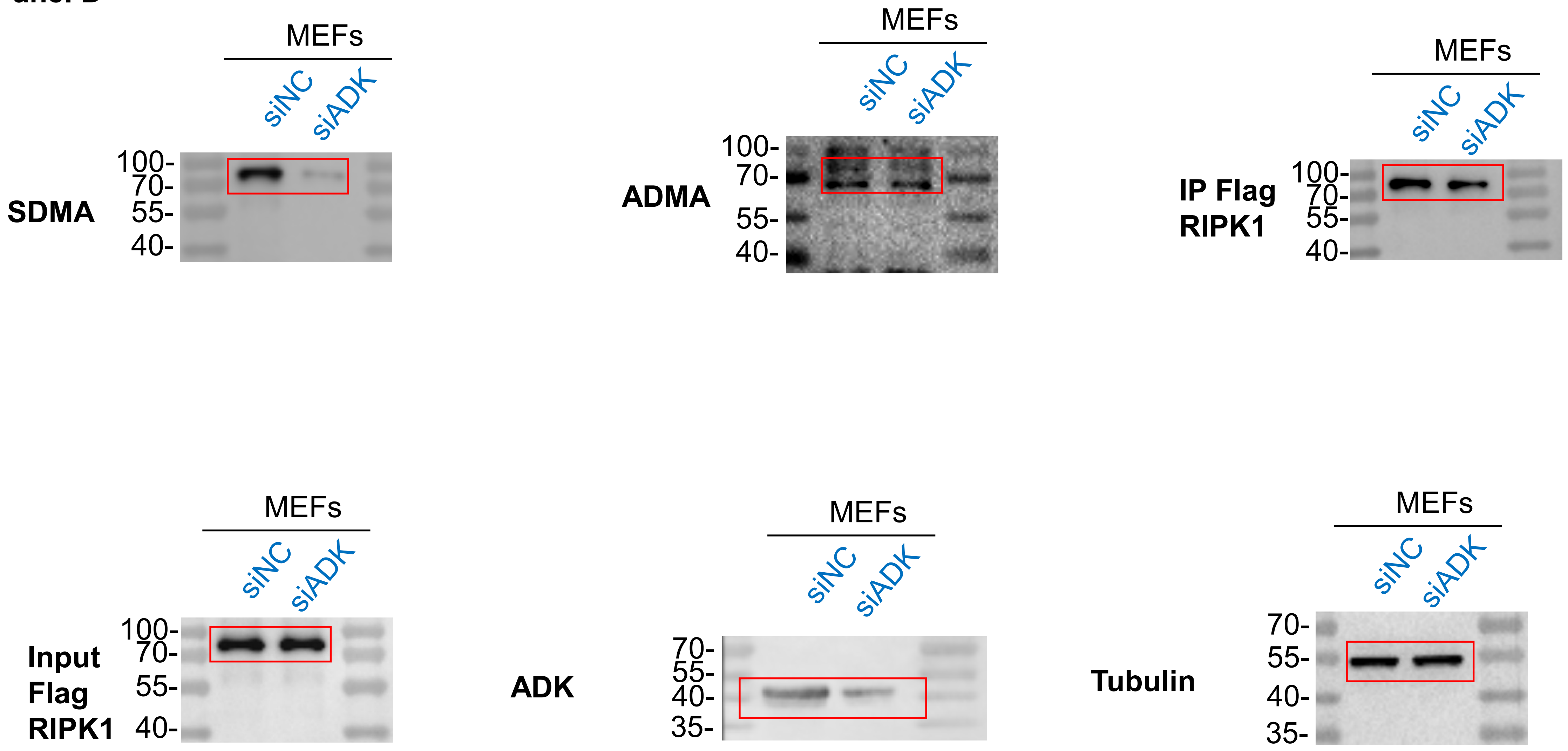

Uncropped blots_Figure 3

Panel E

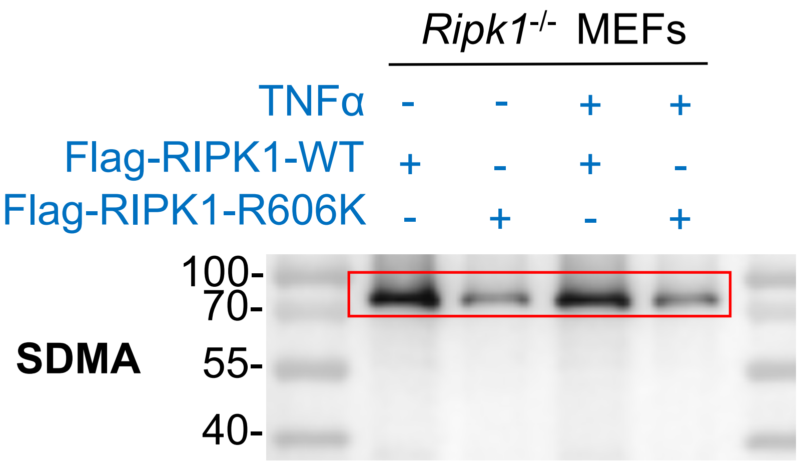

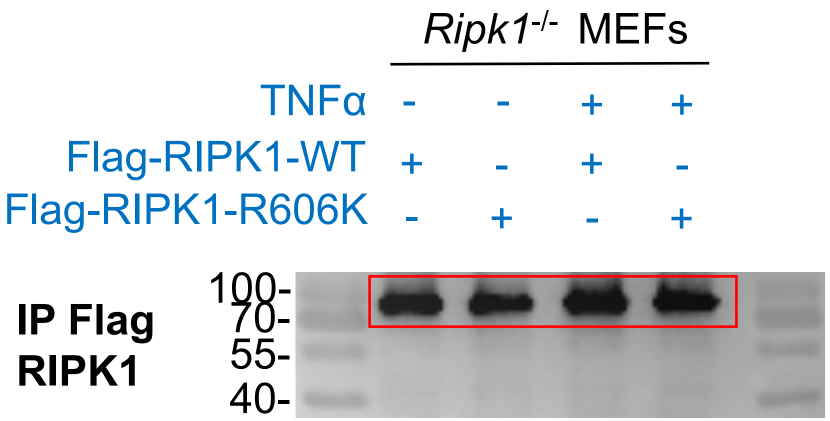

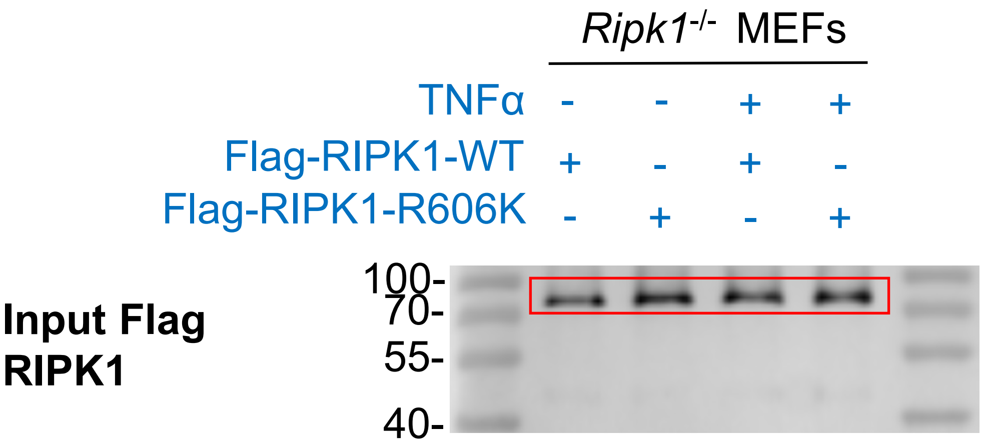

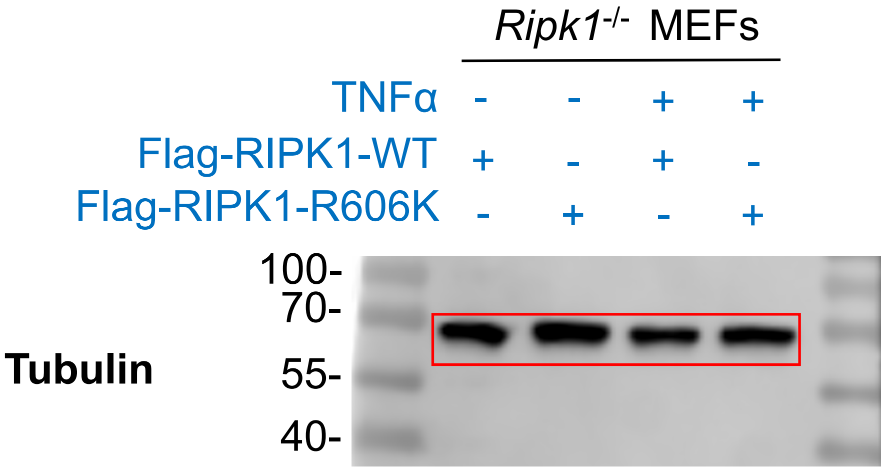

Uncropped blots_Figure 3

Panel F

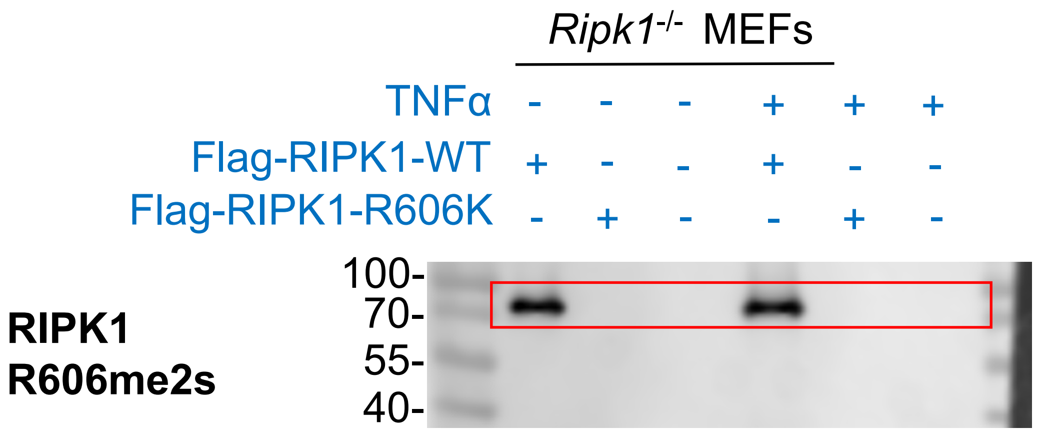

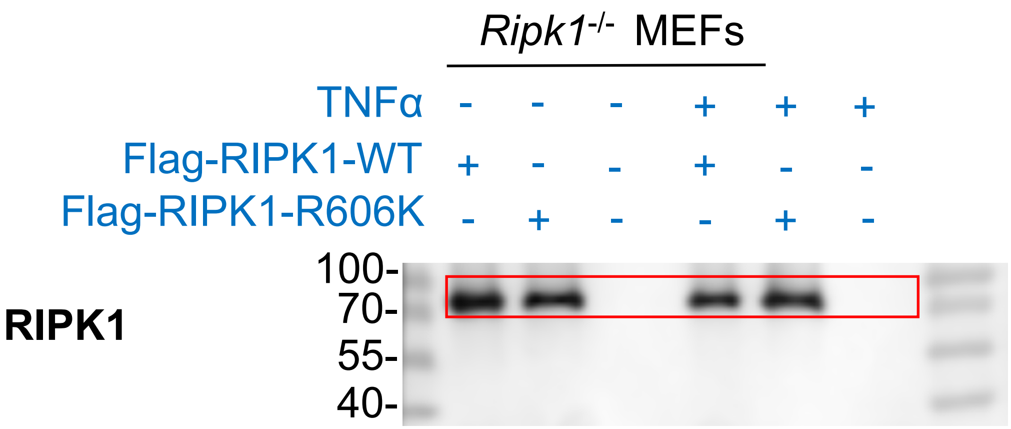

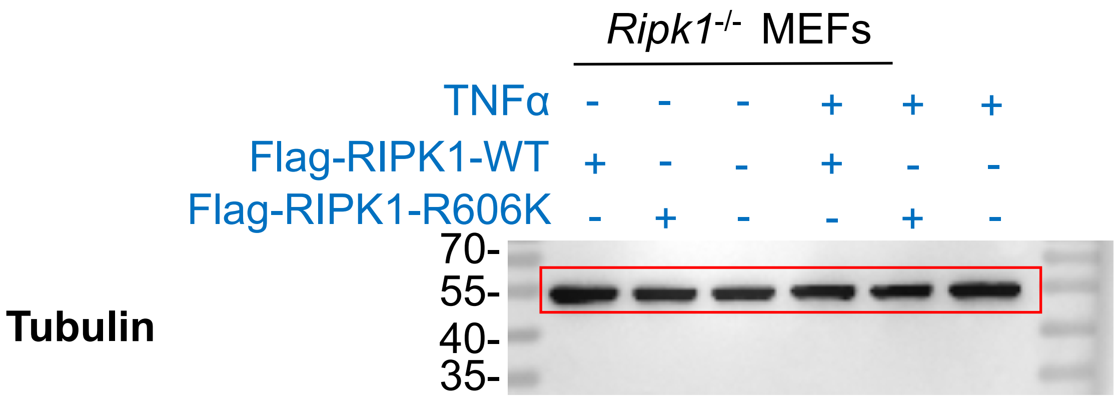

Uncropped blots_Figure 3

Panel G

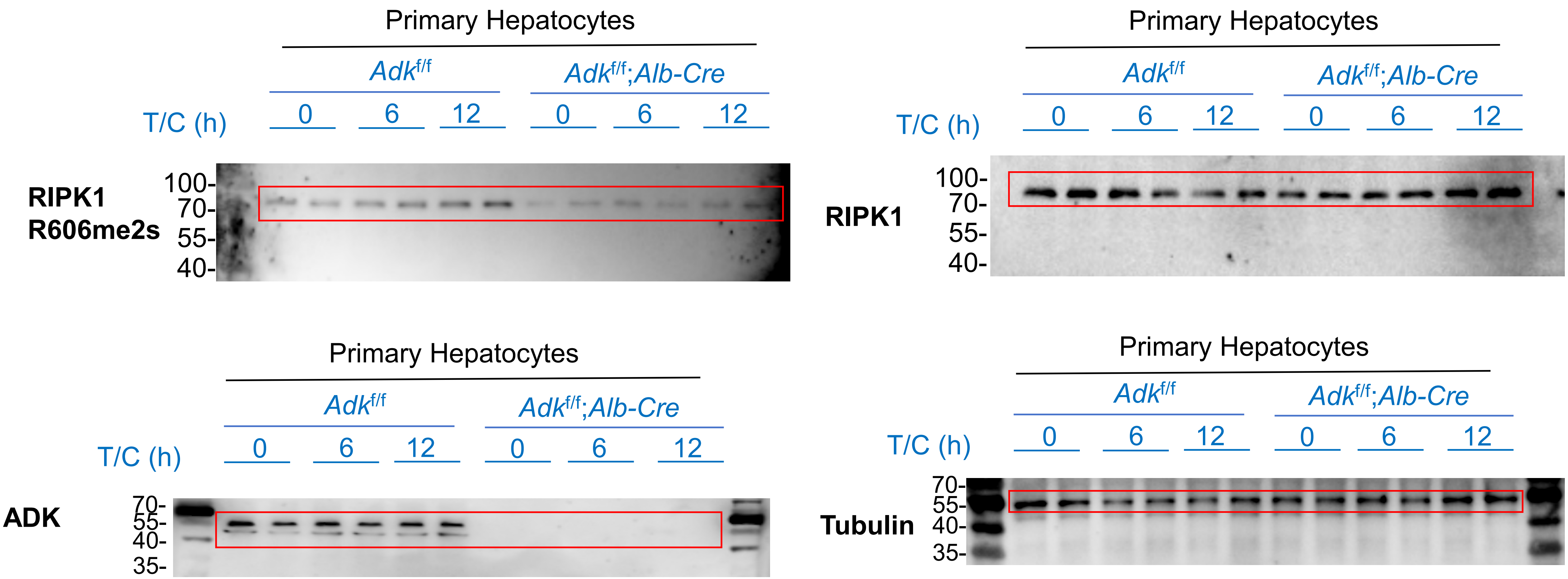

Uncropped blots_Figure 3

Panel H

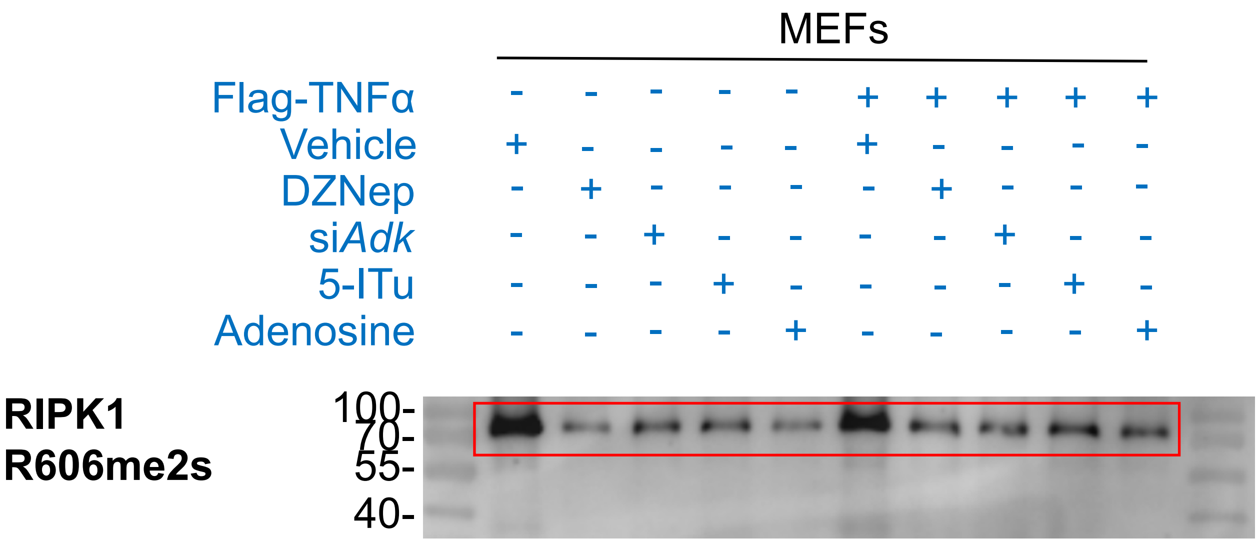

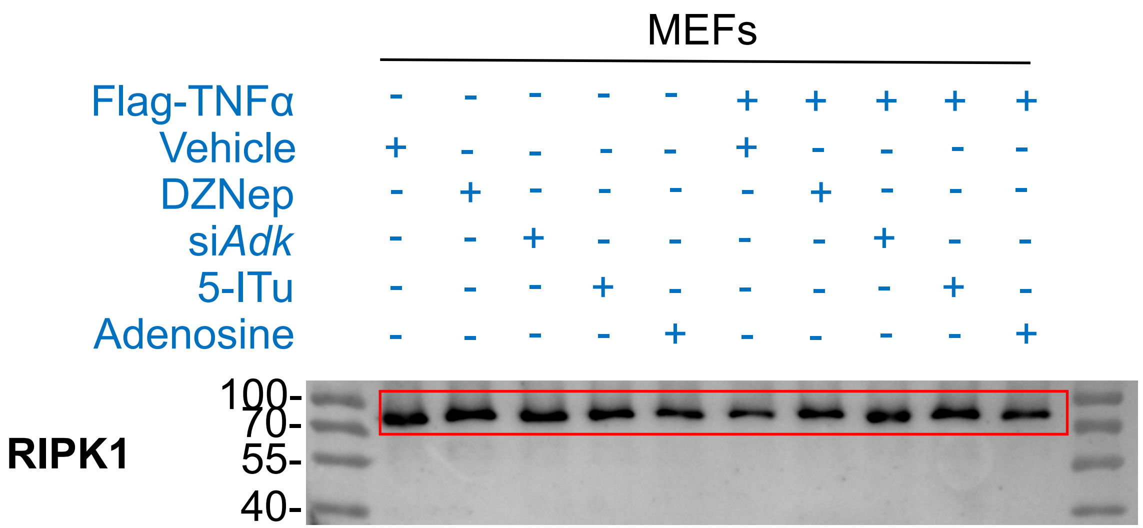

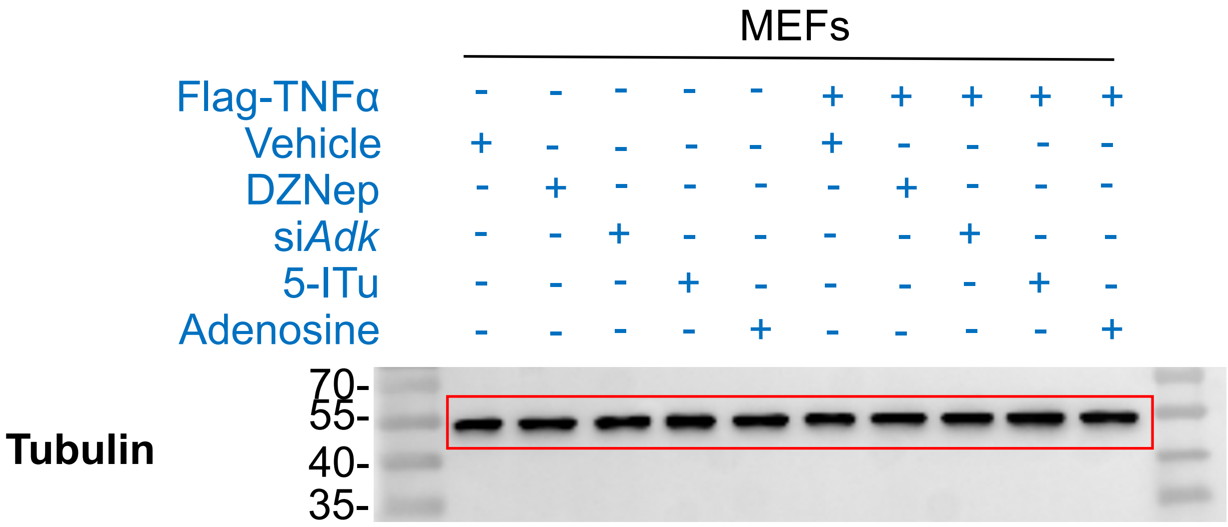

Uncropped blots_Figure 3

Panel L

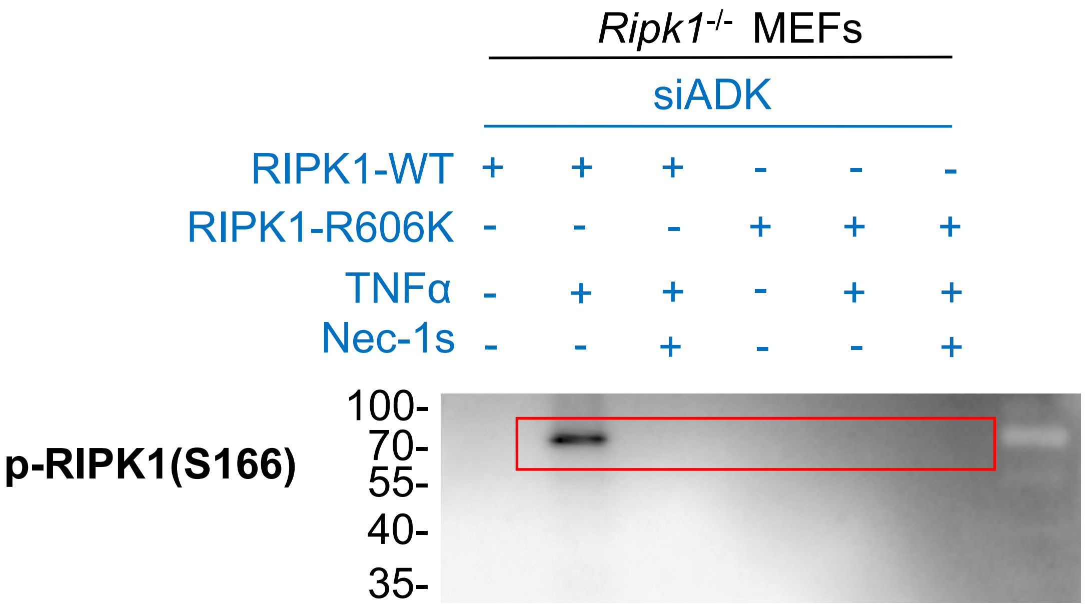

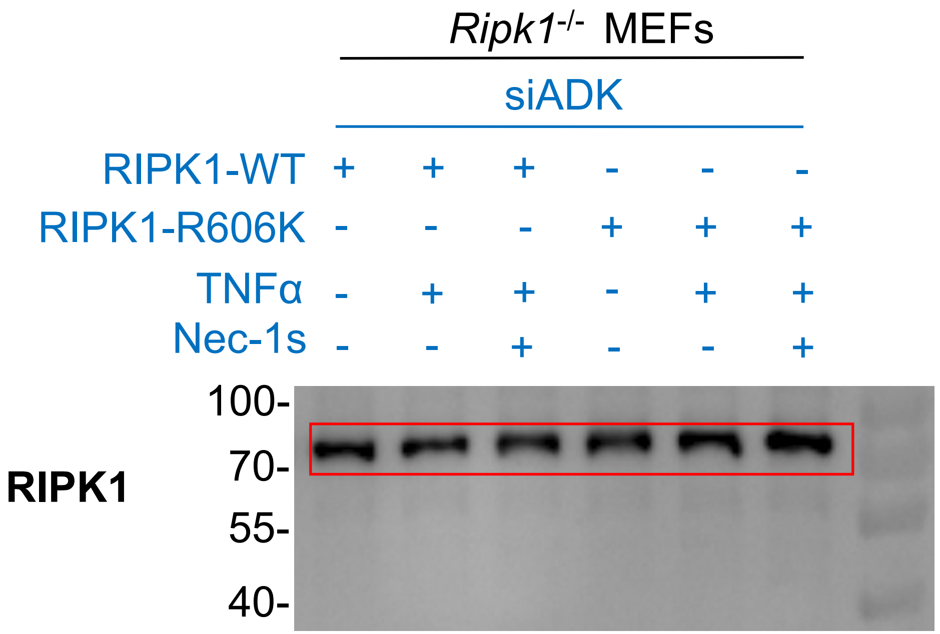

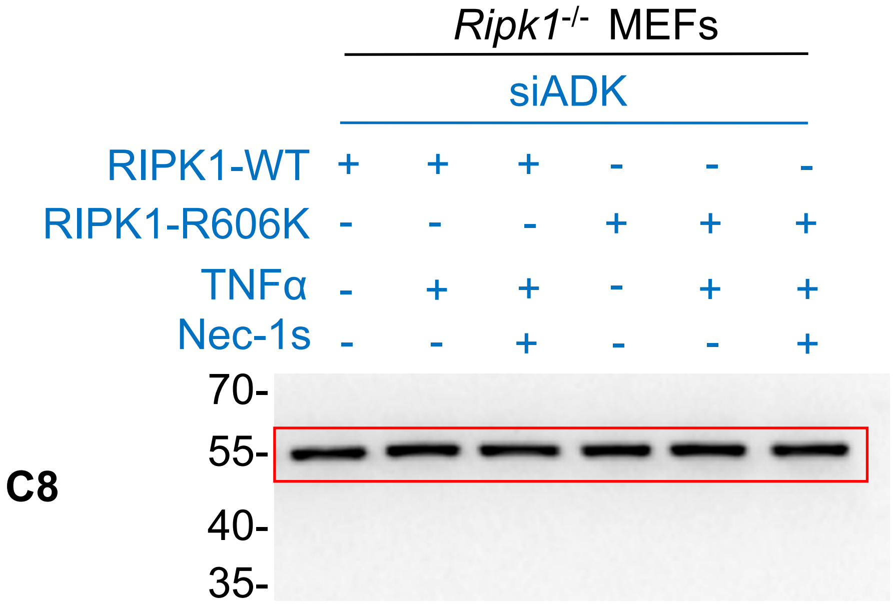

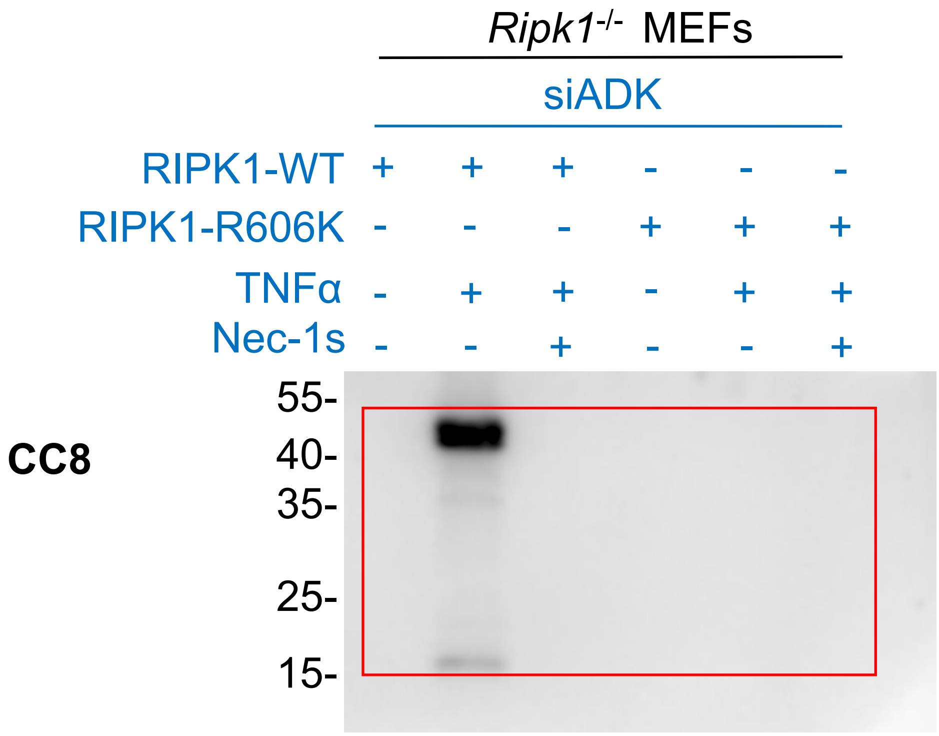

Uncropped blots_Figure 3

Panel L

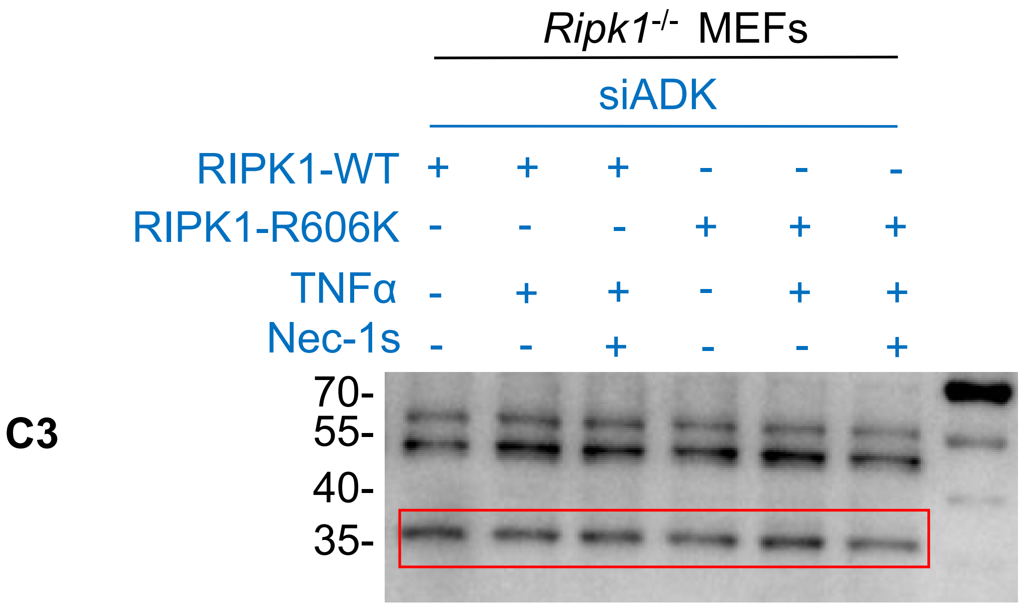

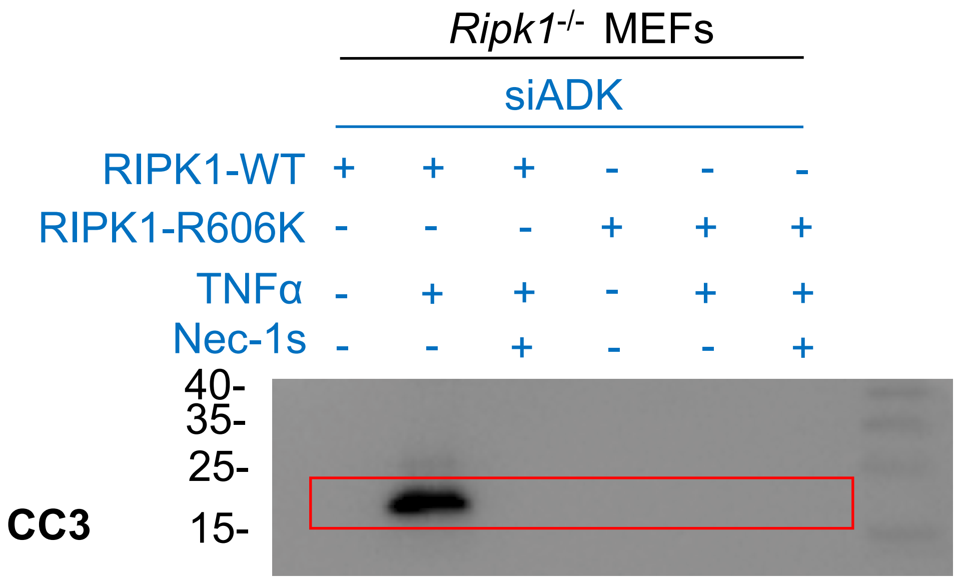

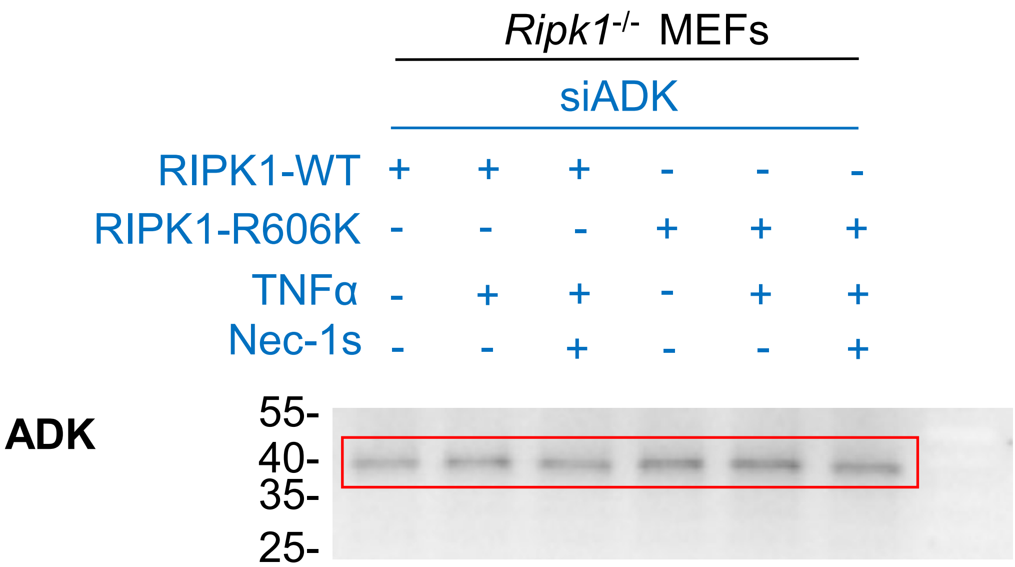

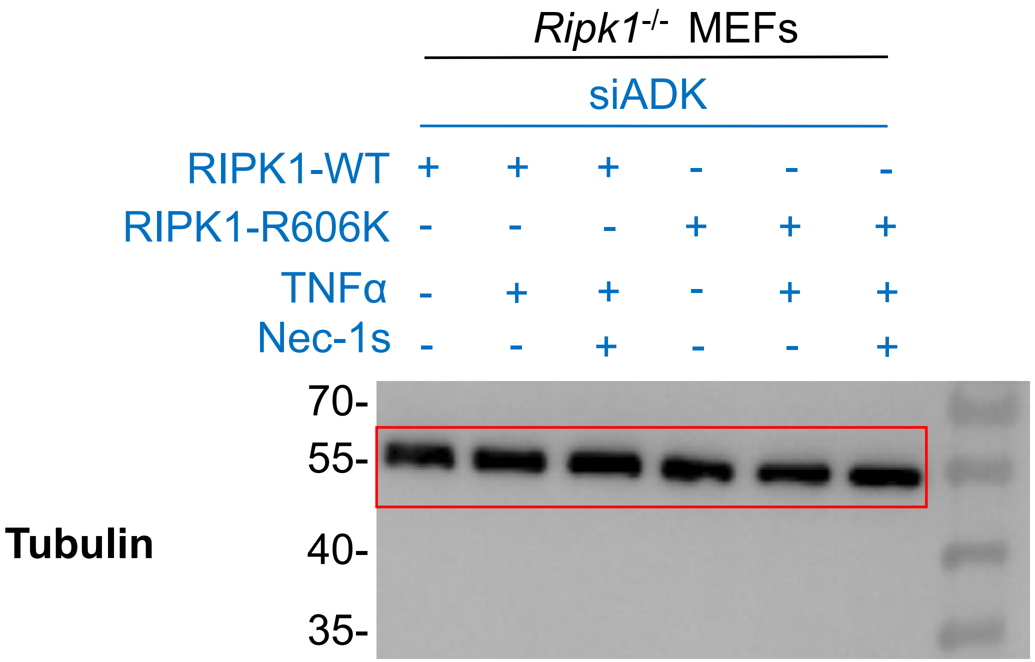

Uncropped blots_Figure 3

Panel N

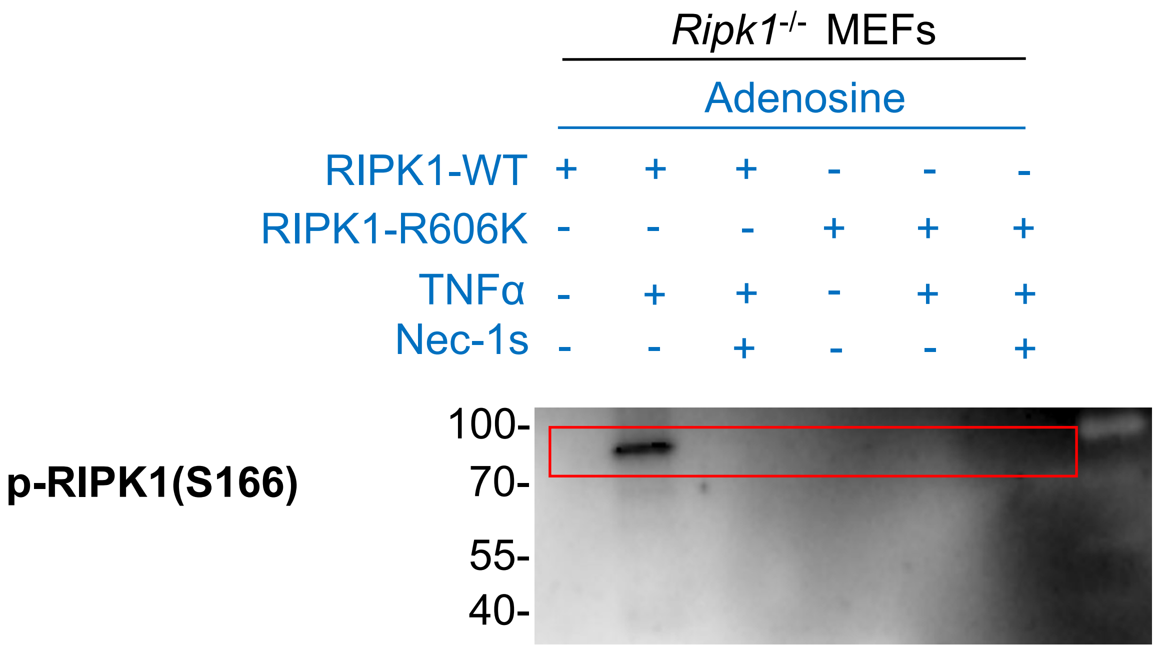

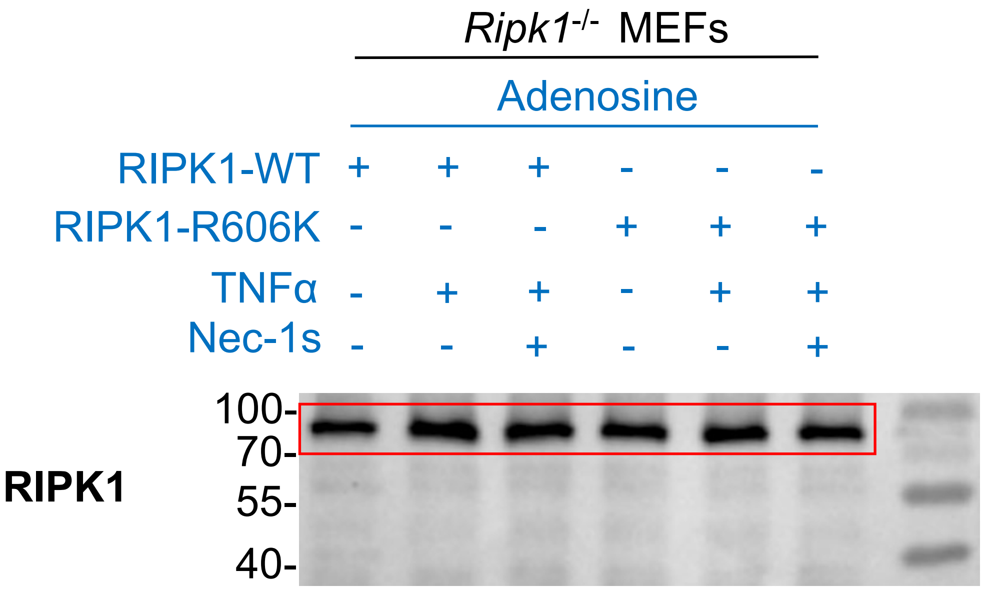

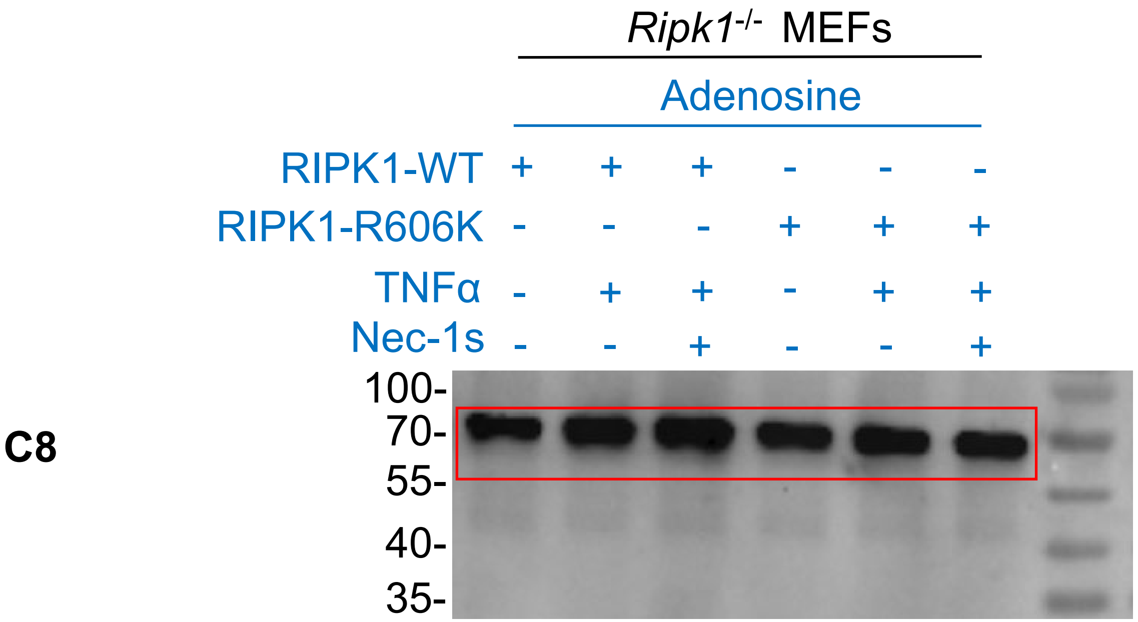

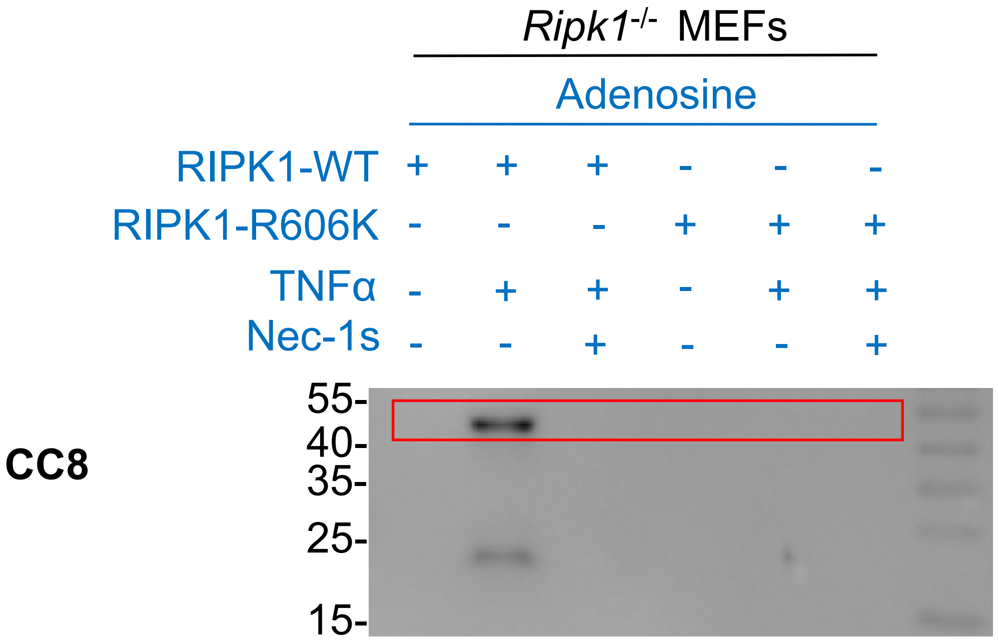

Uncropped blots_Figure 3

Panel N

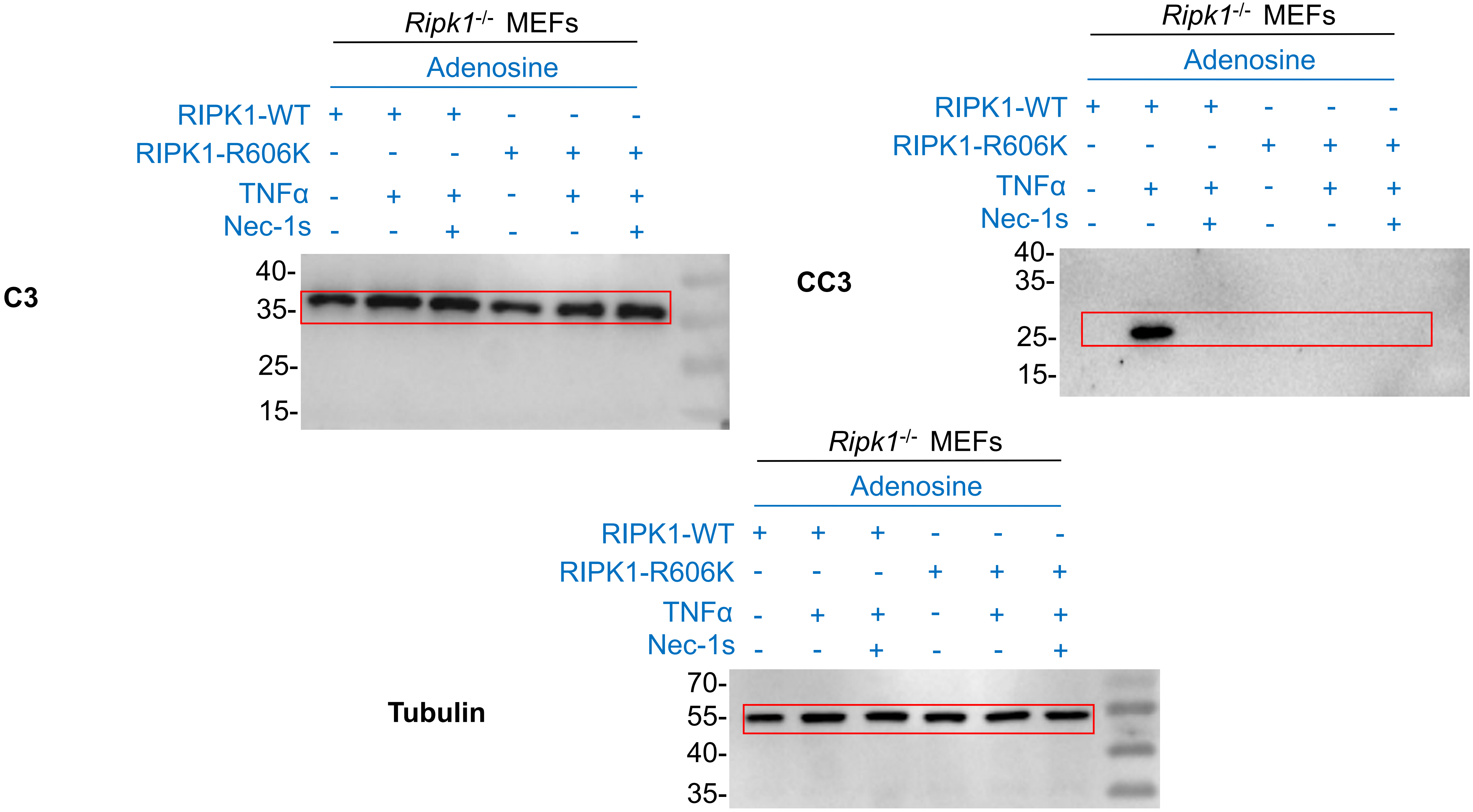

Supplement: SourceData F3 — is the source file for Fig. 3. [file jem_20250603_sourcedataf3.pdf]
