# Supplementary material for: Adenosine metabolic clearance maintains liver homeostasis by licensing arginine methylation of RIPK1
Source: J Exp Med. 2025 Oct 13;223(1):e20250603. doi: 10.1084/jem.20250603 (PMC12517274; doi:10.1084/jem.20250603)

Uncropped blots_Figure 4

Panel C

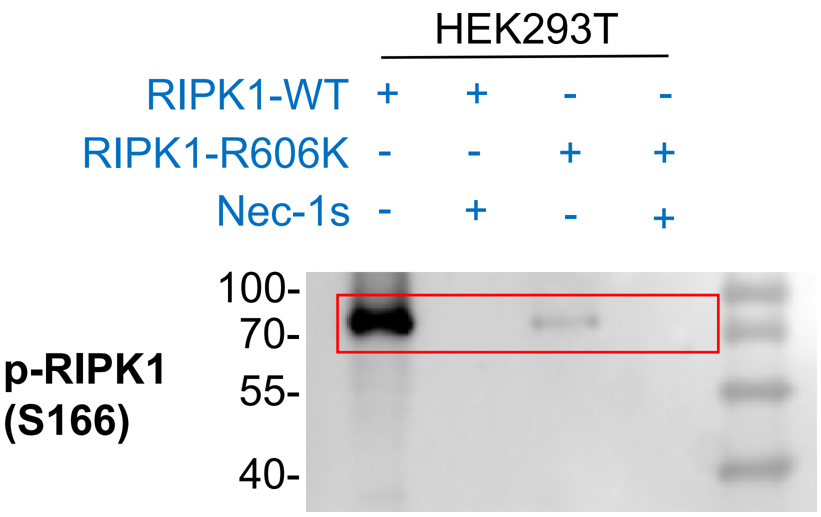

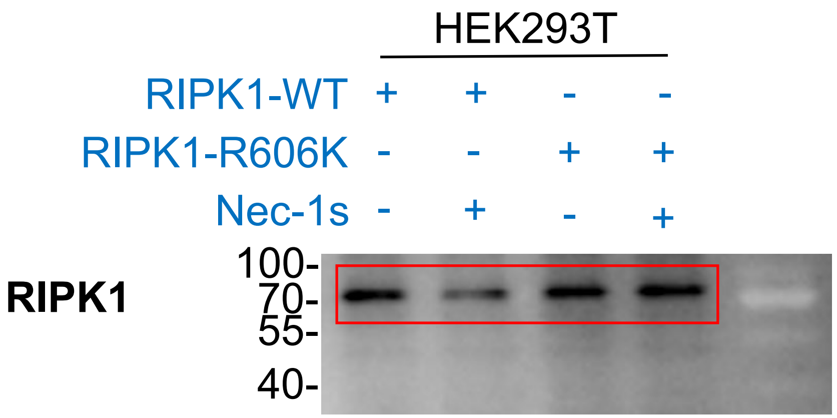

Uncropped blots_Figure 4

Panel D

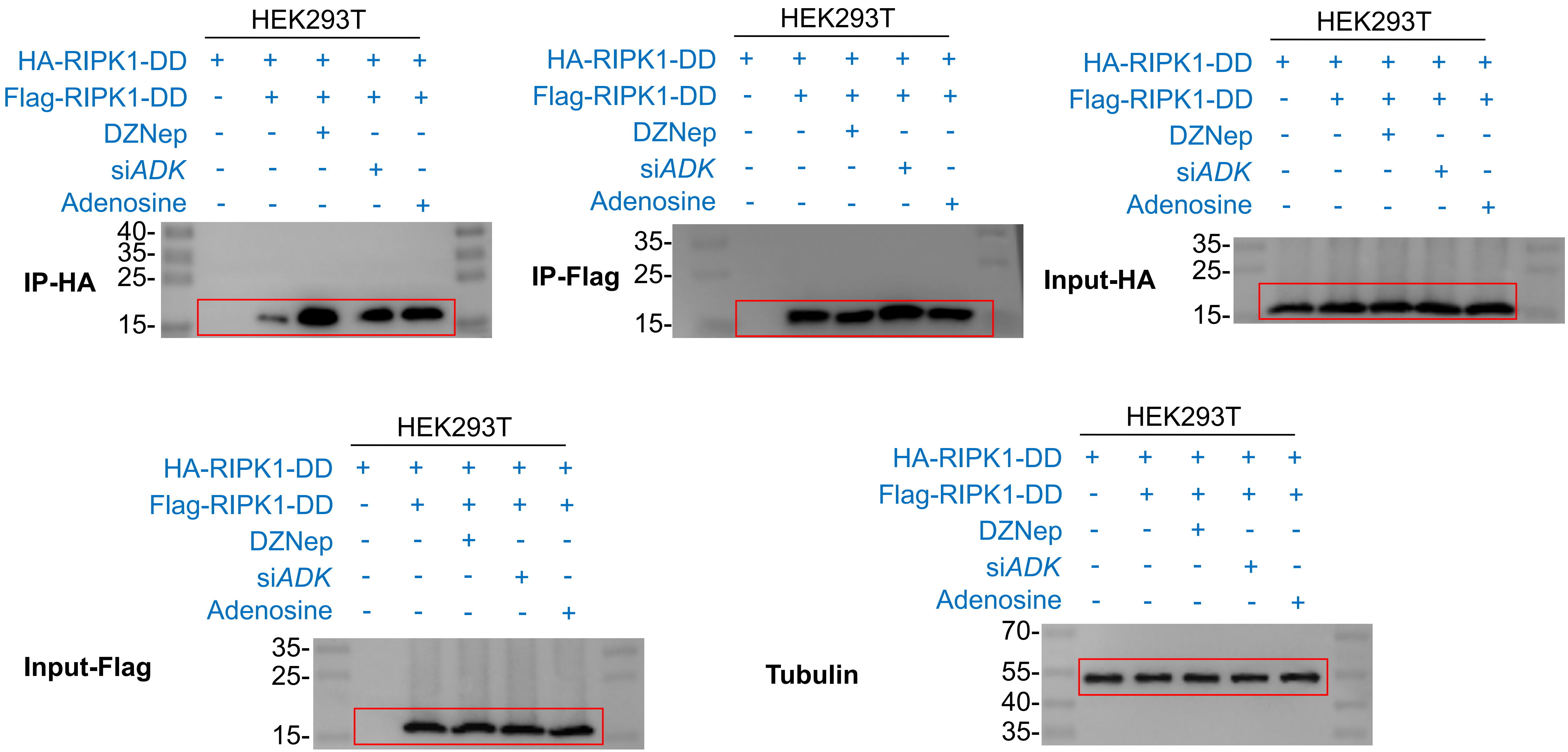

Uncropped blots_Figure 4

Panel E

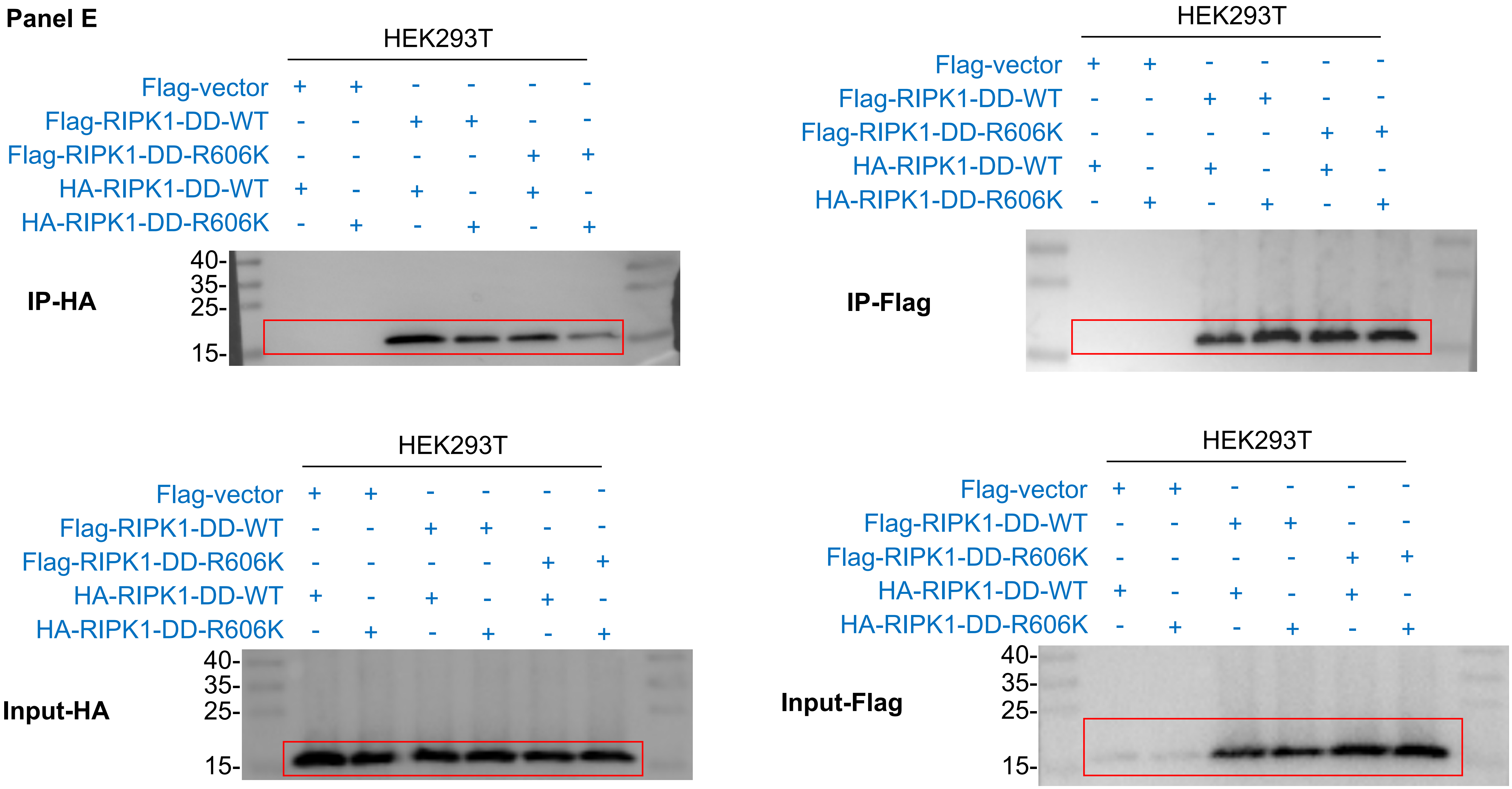

Uncropped blots_Figure 4

Panel E

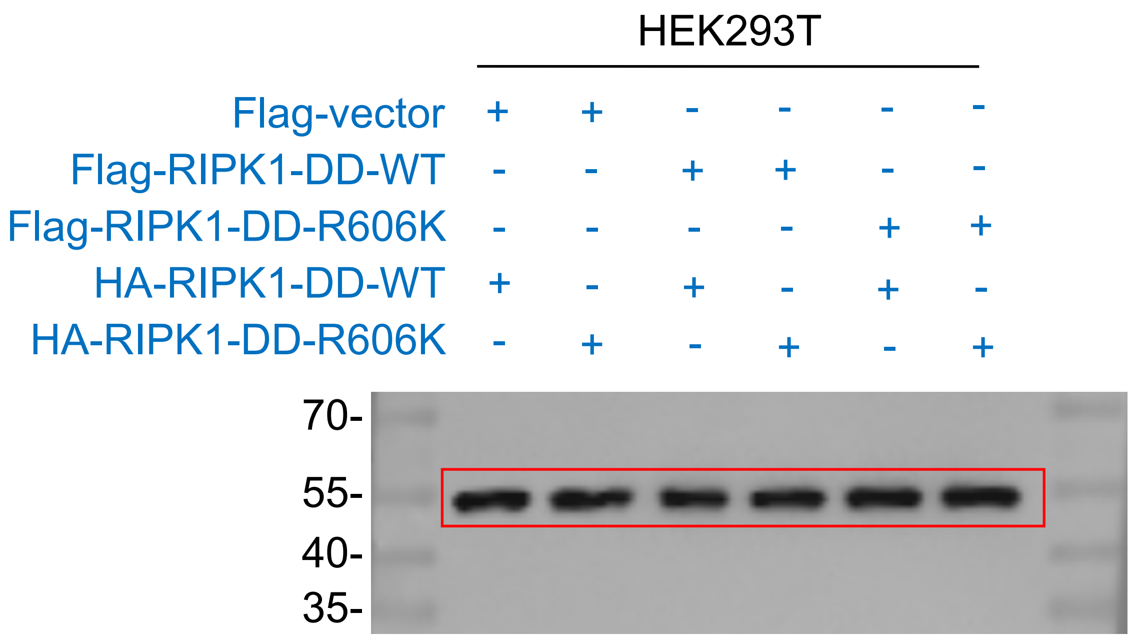

Uncropped blots_Figure 4

Panel F

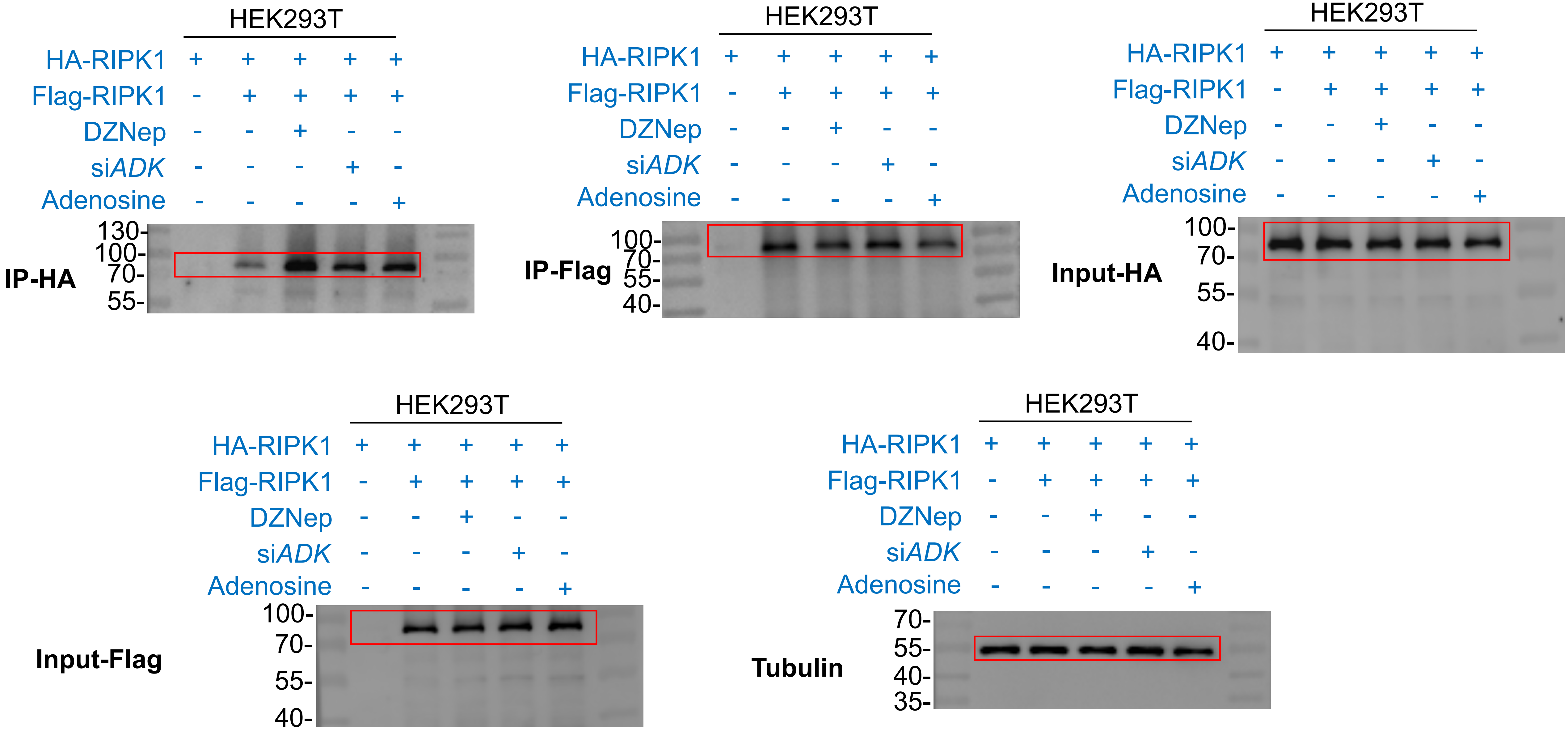

Uncropped blots_Figure 4

Panel G

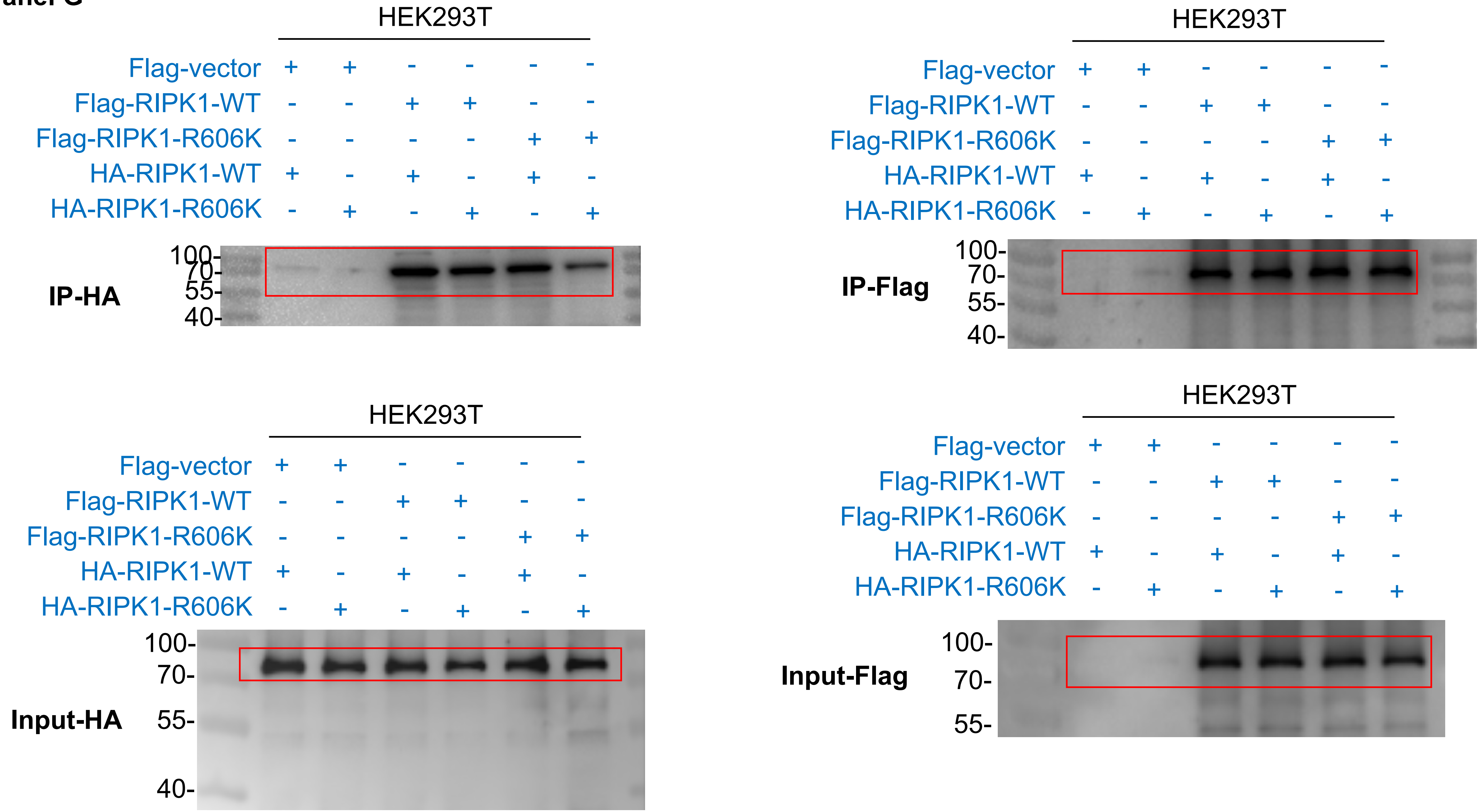

Uncropped blots_Figure 4

Panel G

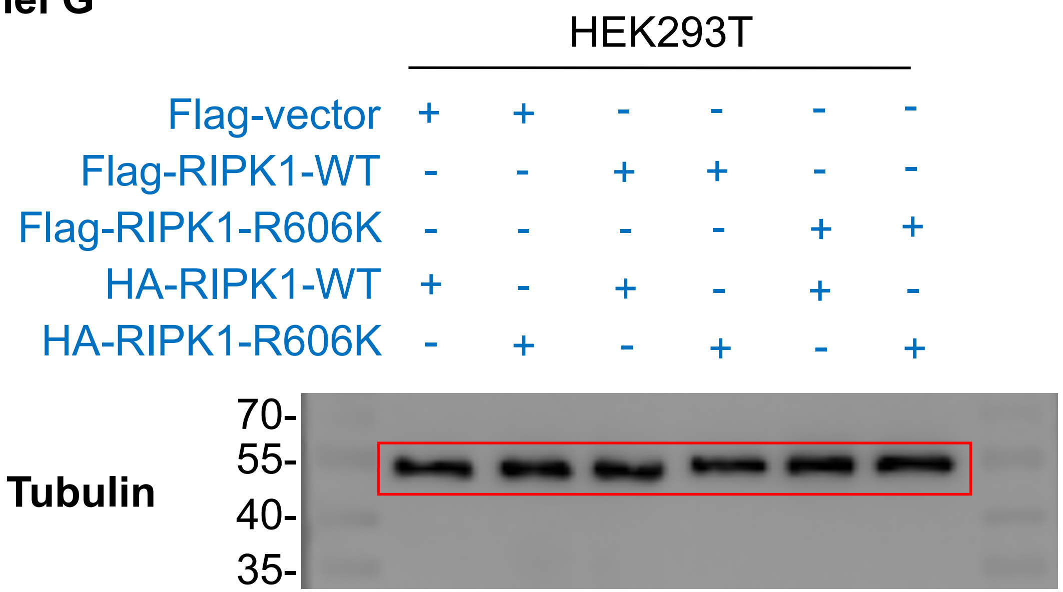

Uncropped blots_Figure 4

Panel H

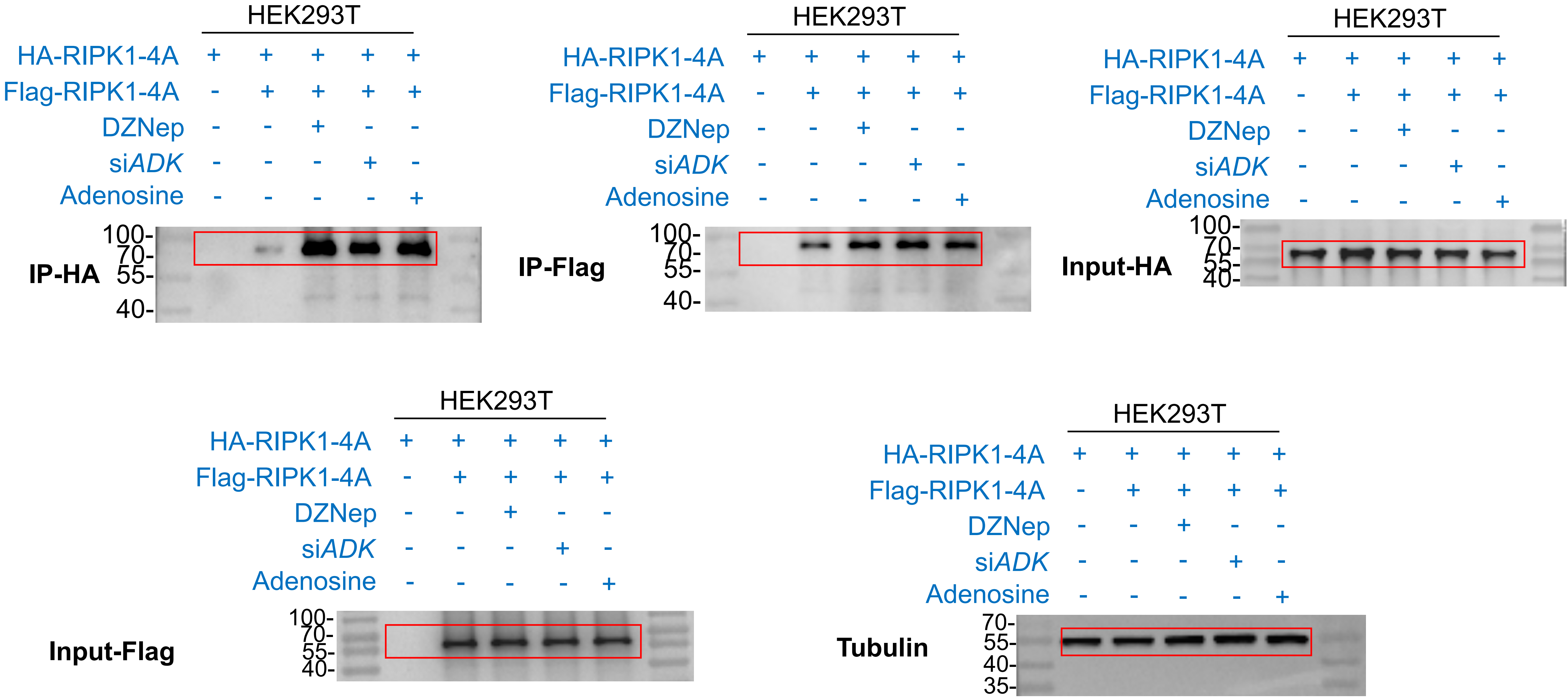

Uncropped blots_Figure 4

Panel I

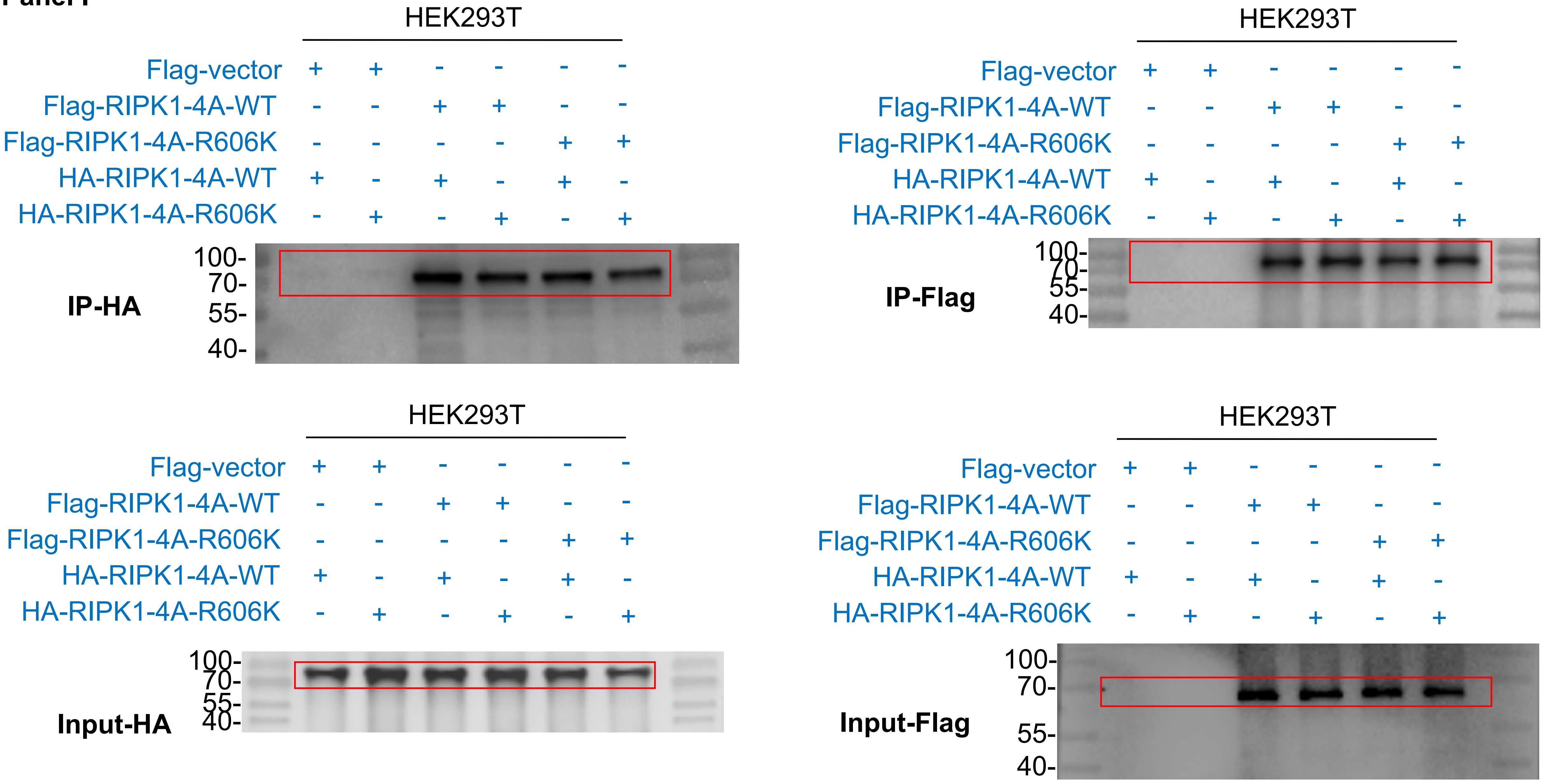

Uncropped blots_Figure 4

Panel I

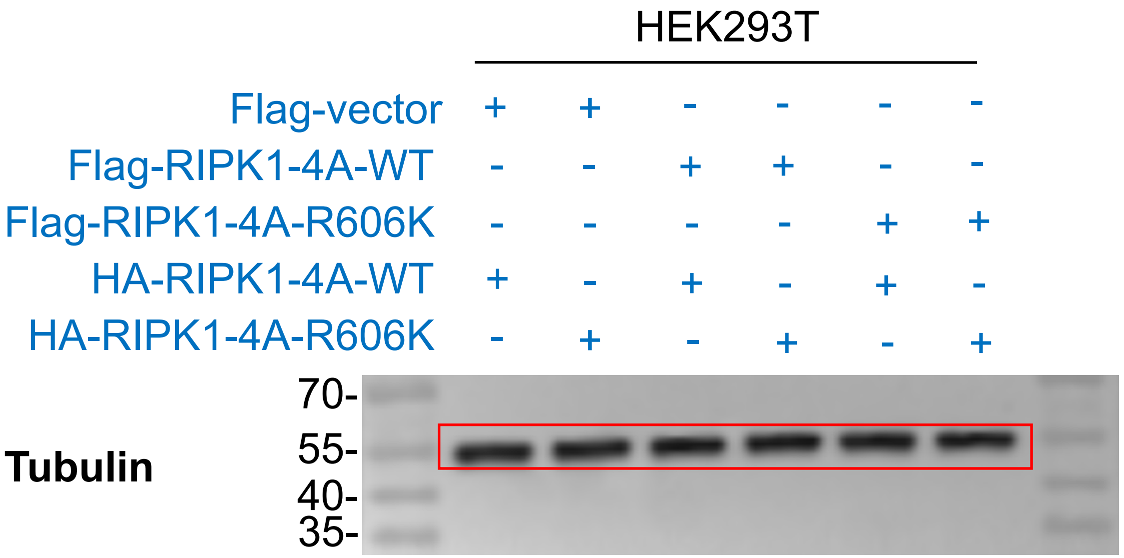

Uncropped blots_Figure 4

Panel J

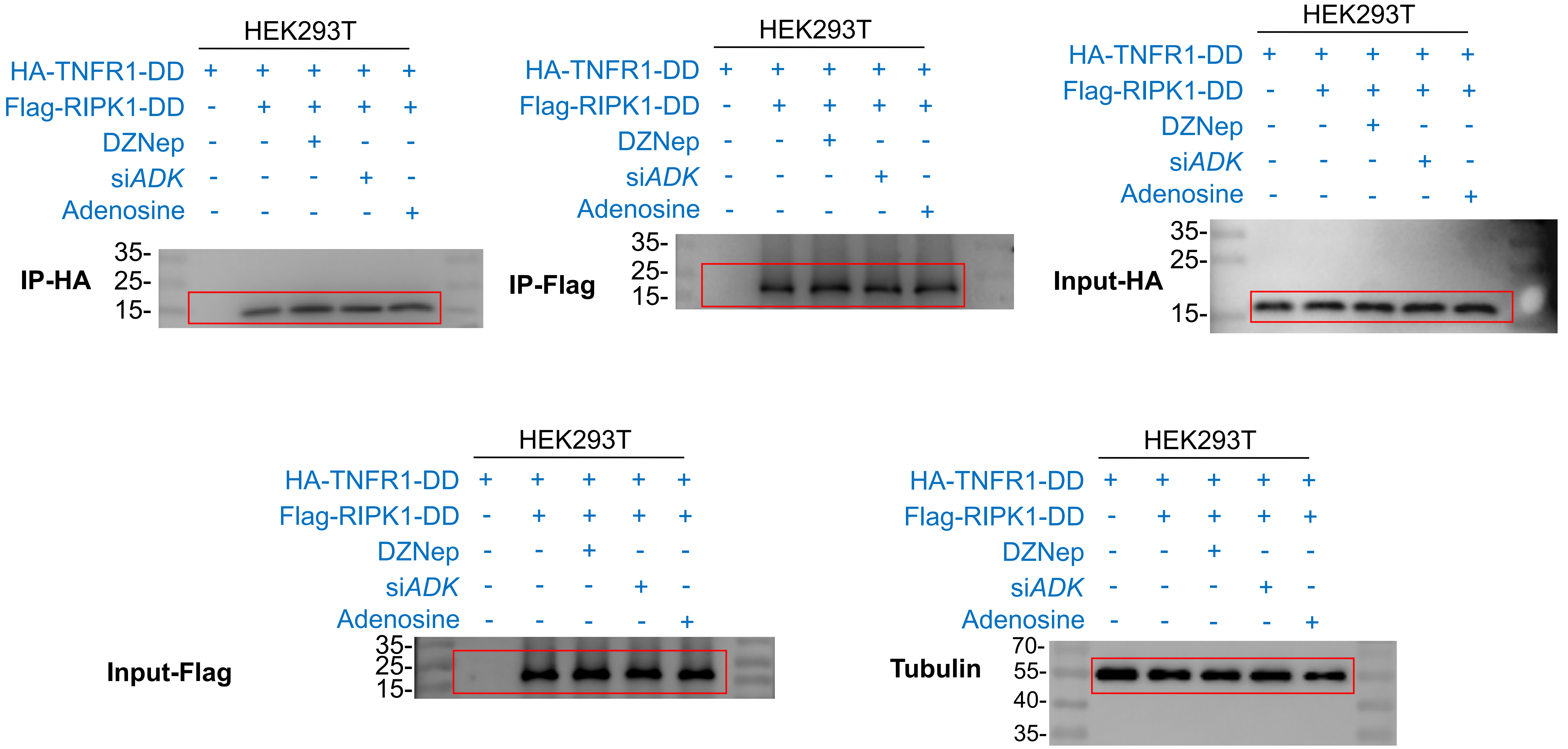

Uncropped blots_Figure 4

Panel K

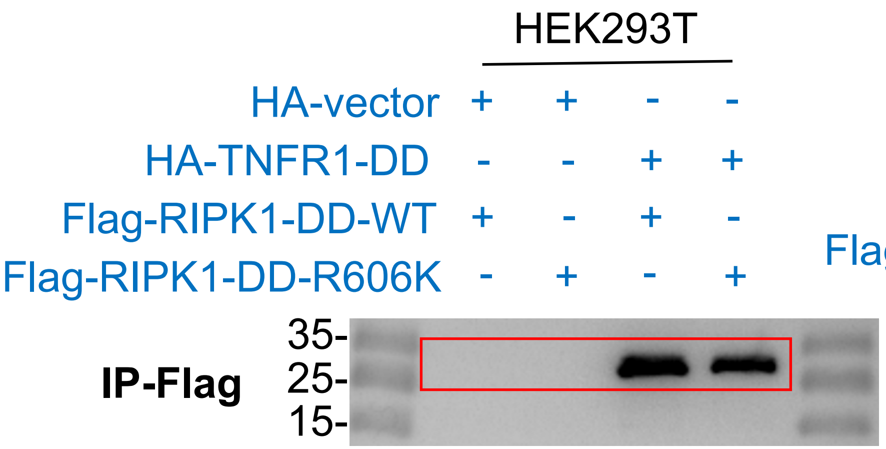

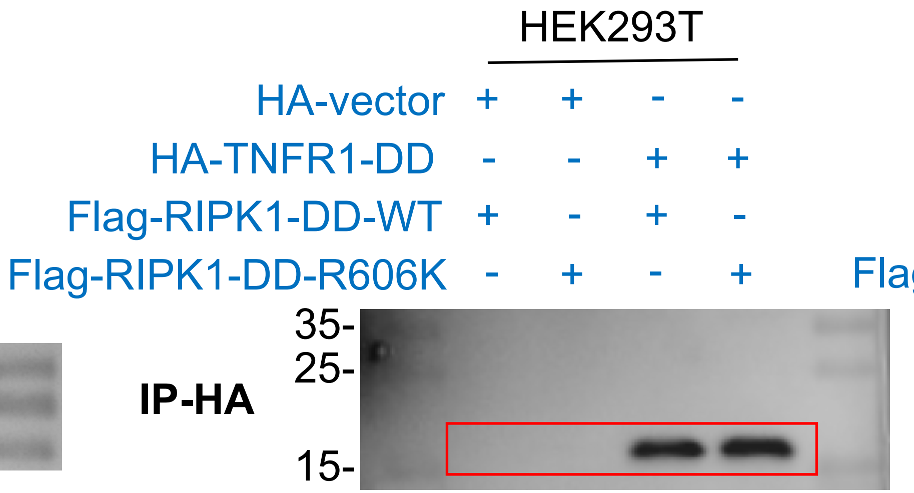

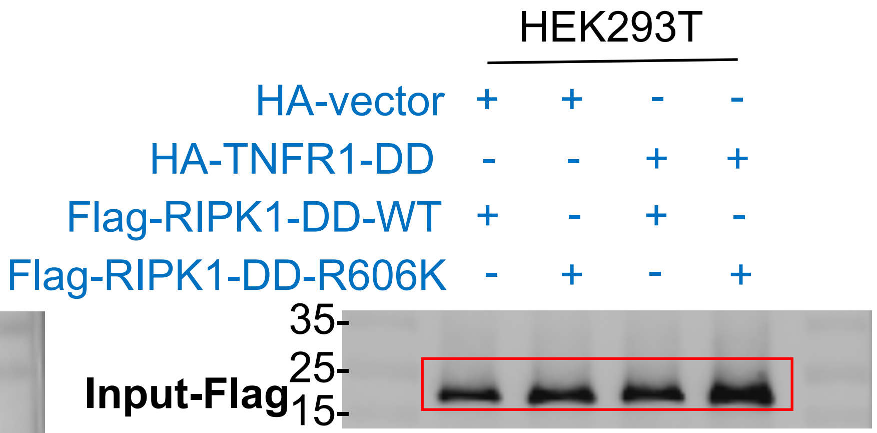

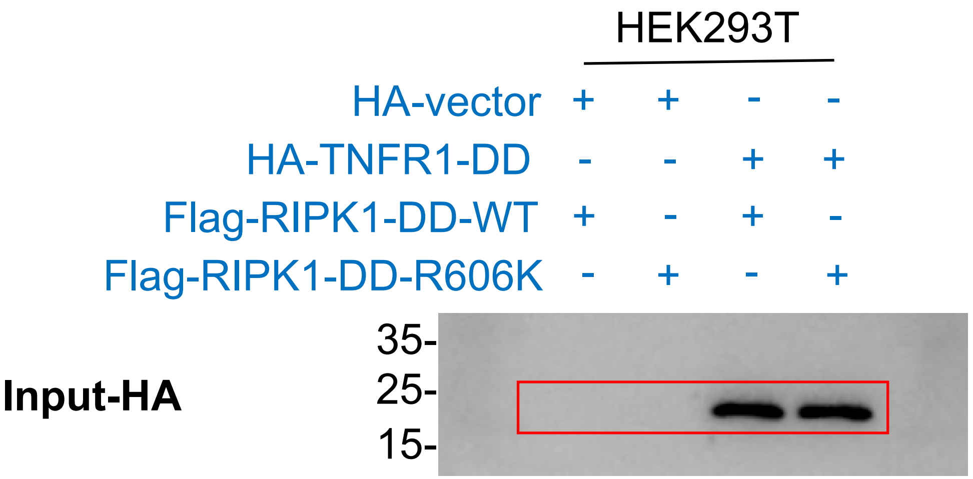

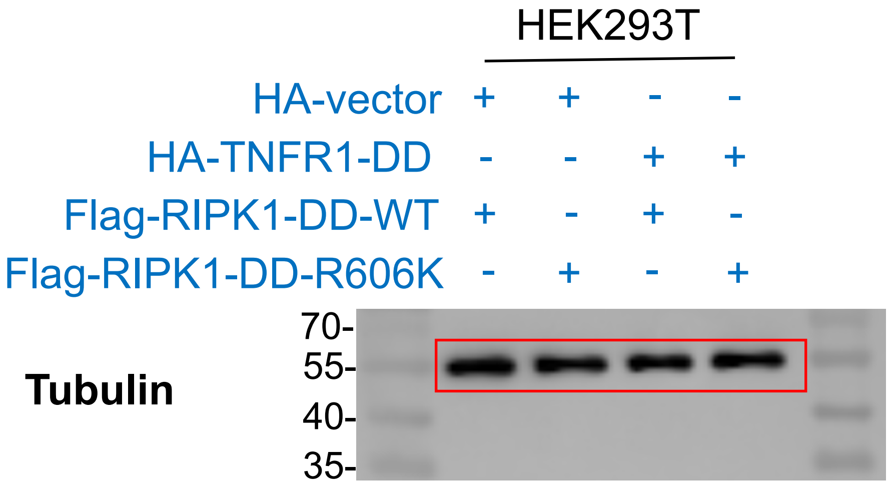

Uncropped blots_Figure 4

Panel L

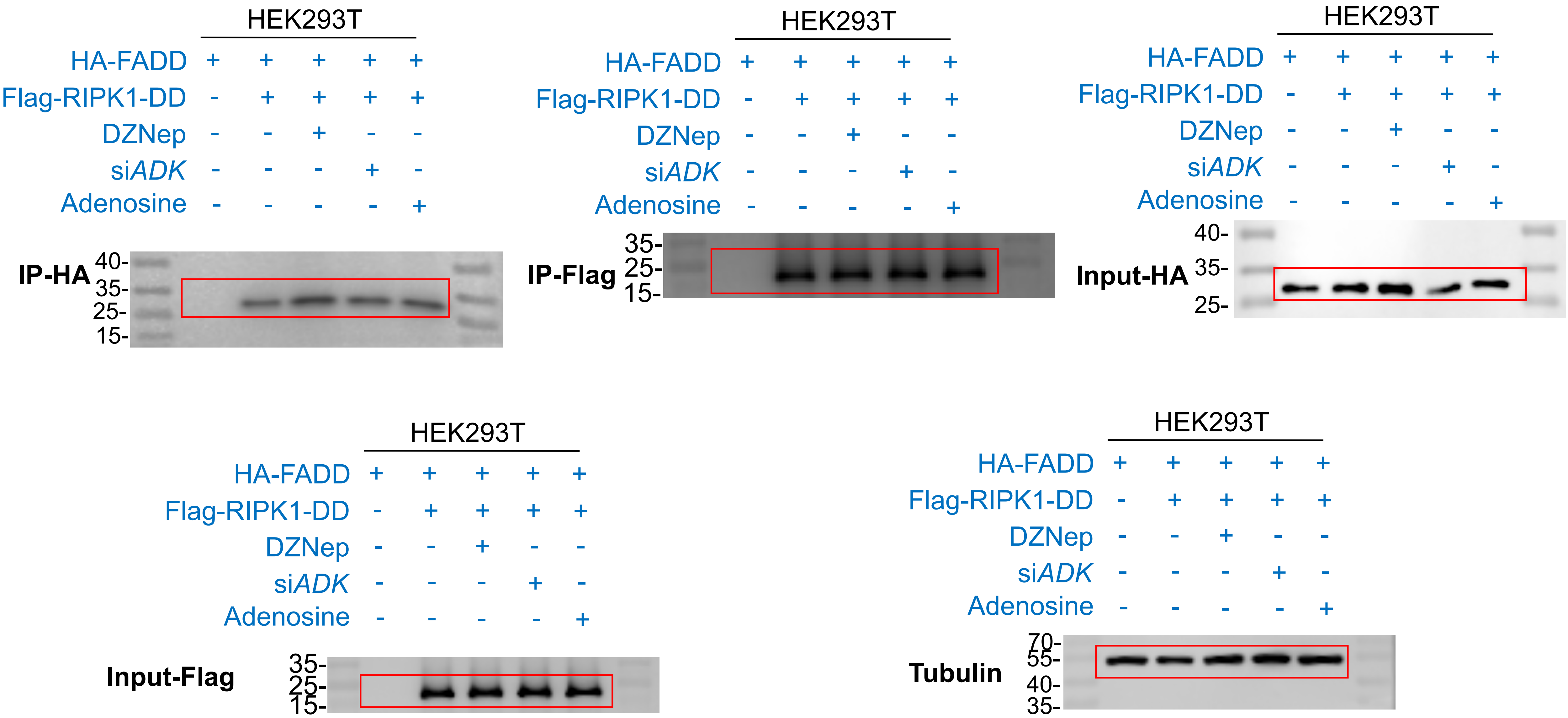

Uncropped blots_Figure 4

Panel M

HEK293T

HA-vector	+	+	-	-
HA-FADD	-	-	+	+
Flag-RIPK1-DD-WT	+	-	+	-
Flag-RIPK1-DD-R606K	-	+	-	+

IP-Flag

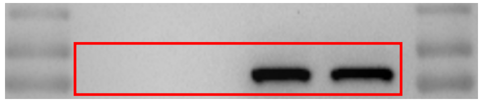

HEK293T

HA-vector	+	+	-	-
HA-FADD	-	-	+	+
Flag-RIPK1-DD-WT	+	-	+	-
Flag-RIPK1-DD-R606K	-	+	-	+

IP-HA

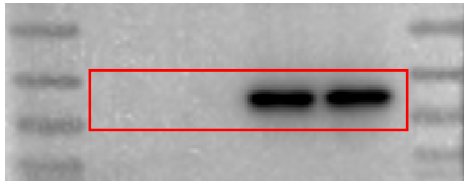

HEK293T

HA-vector	+	+	-	-
HA-FADD	-	-	+	+
Flag-RIPK1-DD-WT	+	-	+	-
Flag-RIPK1-DD-R606K	-	+	-	+

Input-Flag

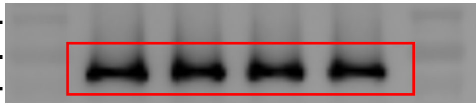

HEK293T

HA-vector	+	+	-	-
HA-FADD	-	-	+	+
Flag-RIPK1-DD-WT	+	-	+	-
Flag-RIPK1-DD-R606K	-	+	-	+

Input-HA

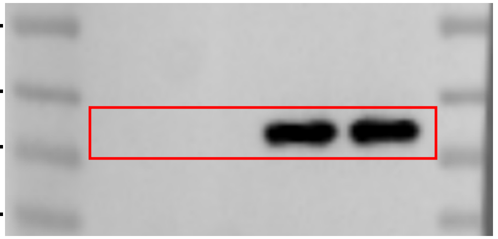

HEK293T

HA-vector	+	+	-	-
HA-FADD	-	-	+	+
Flag-RIPK1-DD-WT	+	-	+	-
Flag-RIPK1-DD-R606K	-	+	-	+

Tubulin

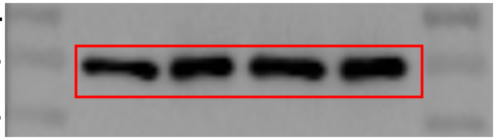

Uncropped blots_Figure 4

Panel N

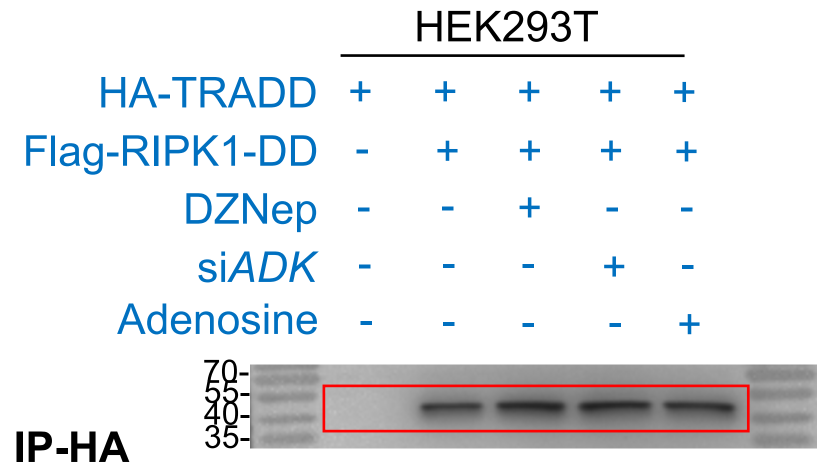

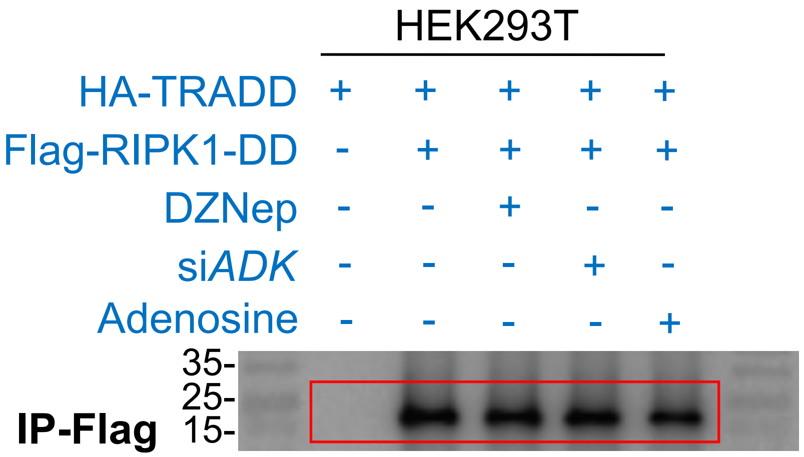

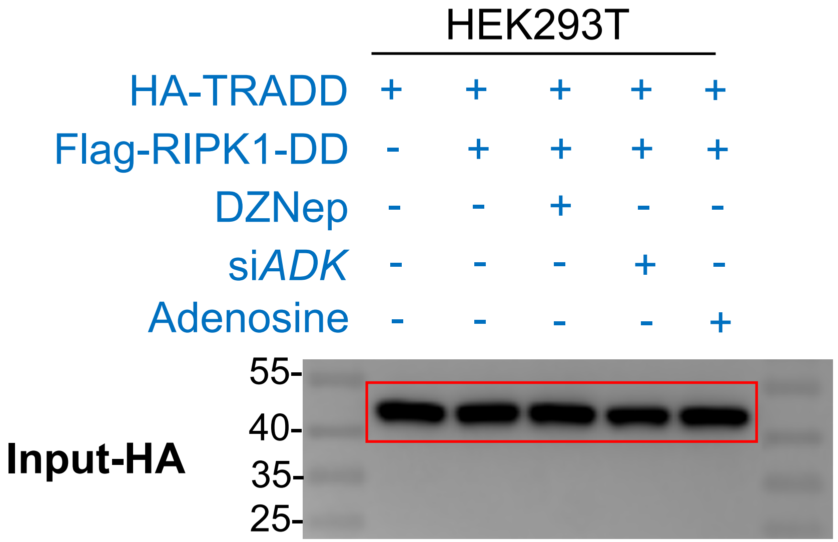

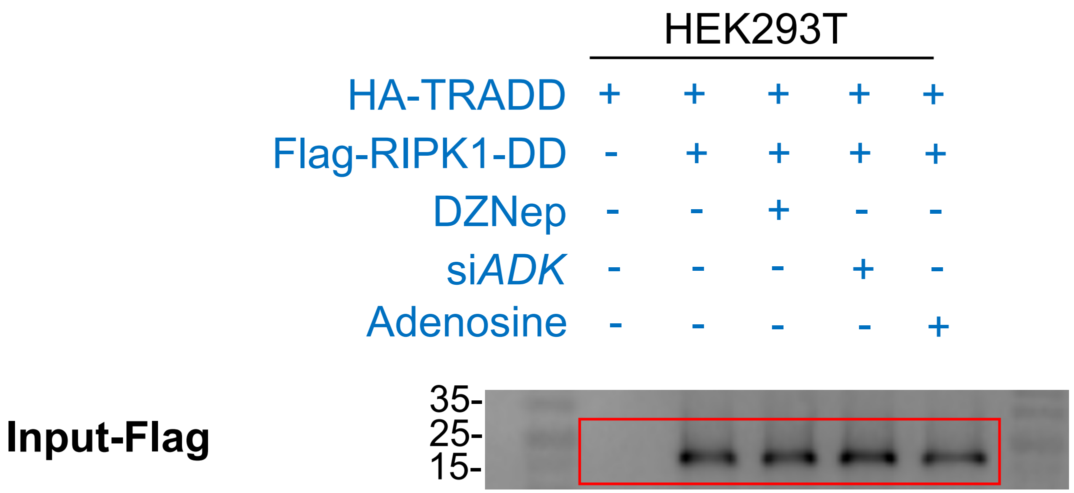

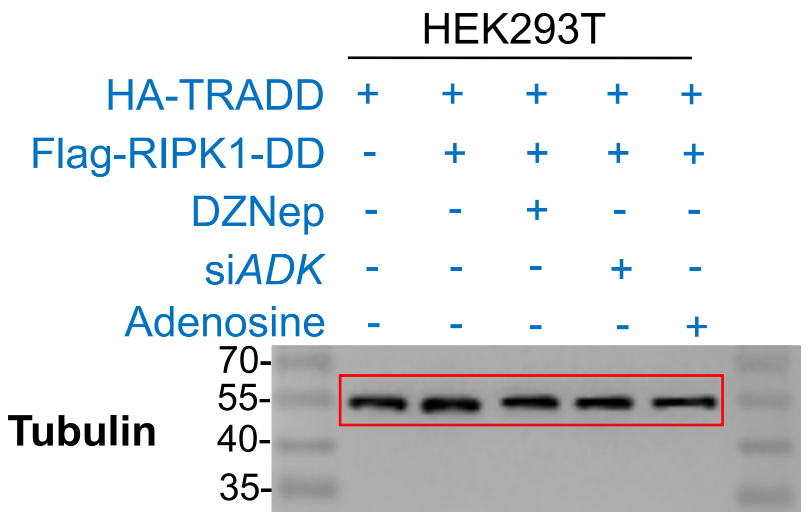

Uncropped blots_Figure 4

Panel O

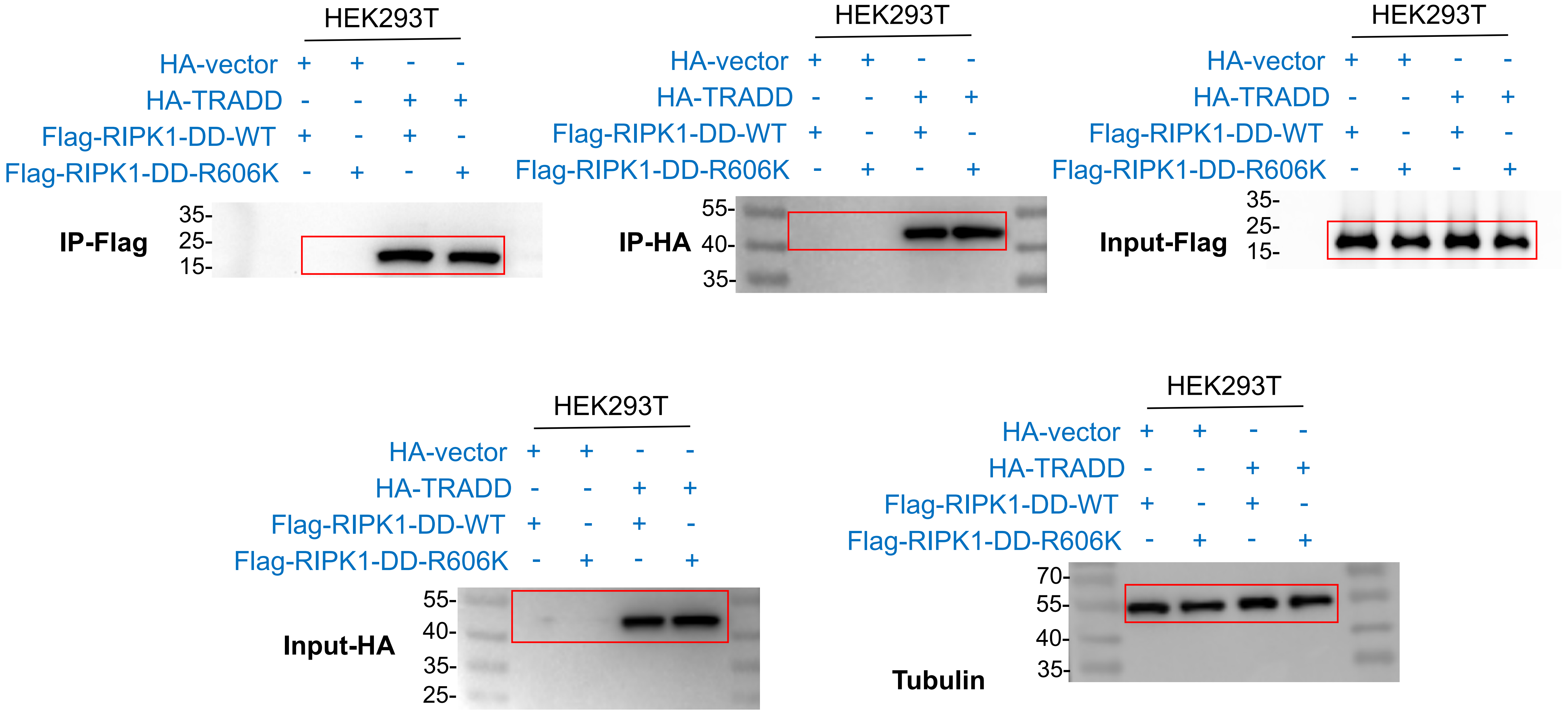

Supplement: SourceData F4 — is the source file for Fig. 4. [file jem_20250603_sourcedataf4.pdf]
