# Supplementary material for: Adenosine metabolic clearance maintains liver homeostasis by licensing arginine methylation of RIPK1
Source: J Exp Med. 2025 Oct 13;223(1):e20250603. doi: 10.1084/jem.20250603 (PMC12517274; doi:10.1084/jem.20250603)

Uncropped blots_Figure 5

Panel A

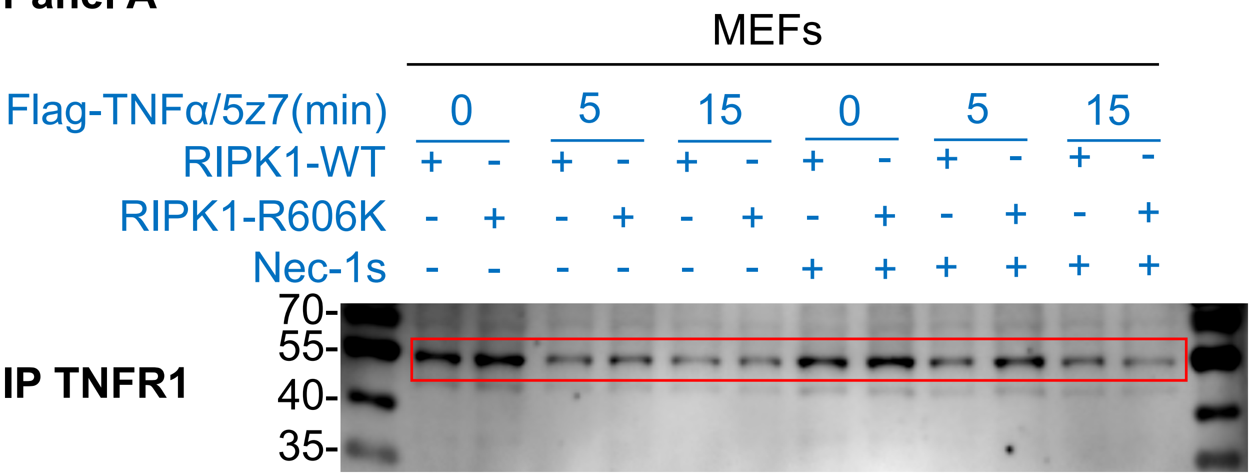

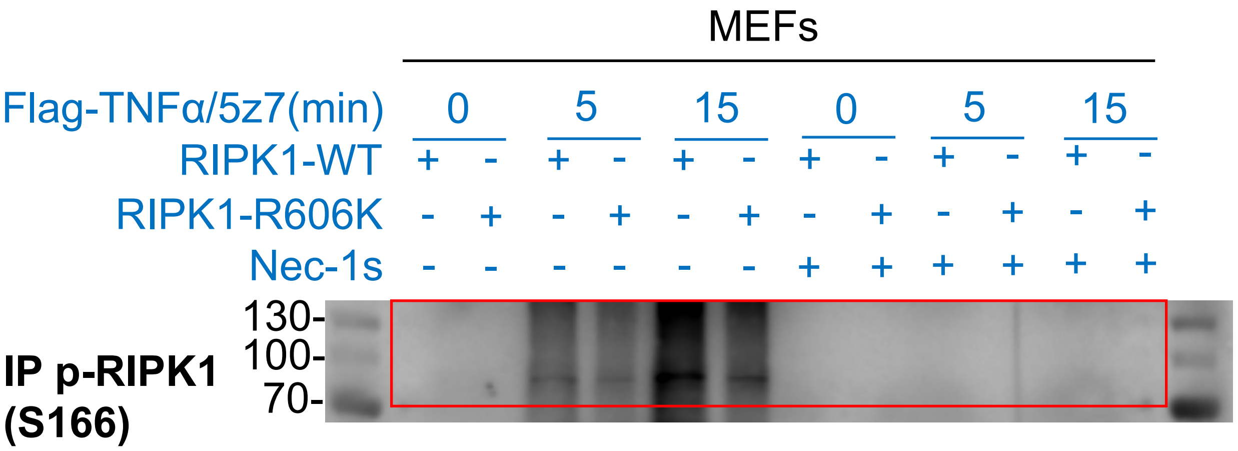

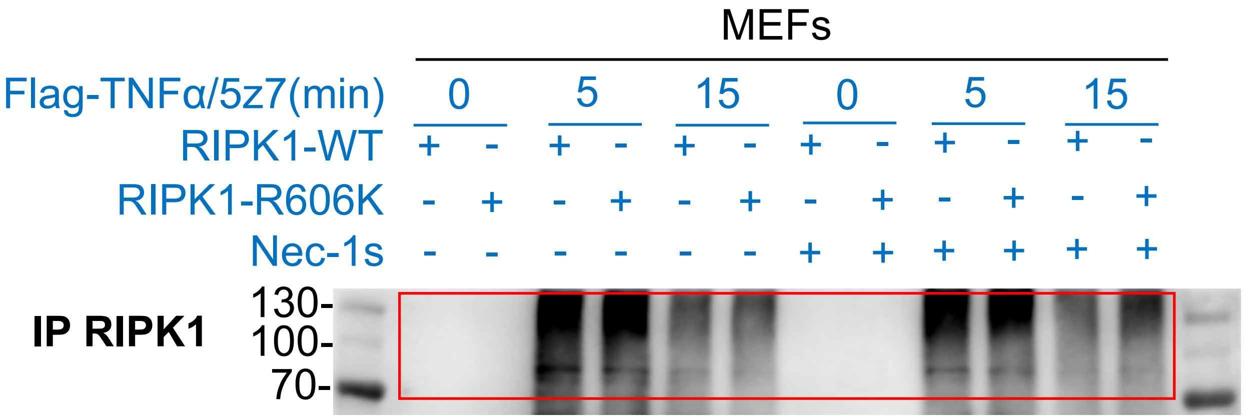

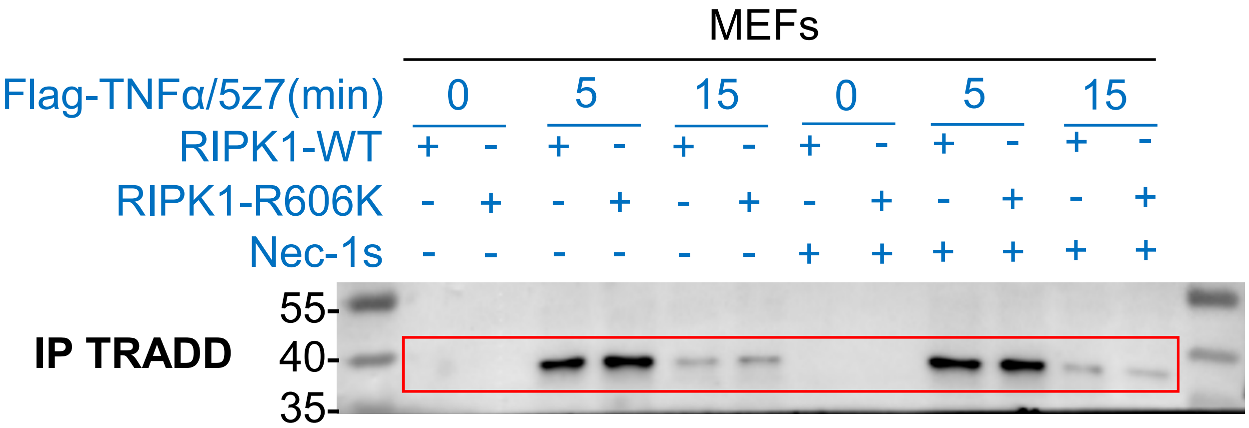

Uncropped blots_Figure 5

Panel A

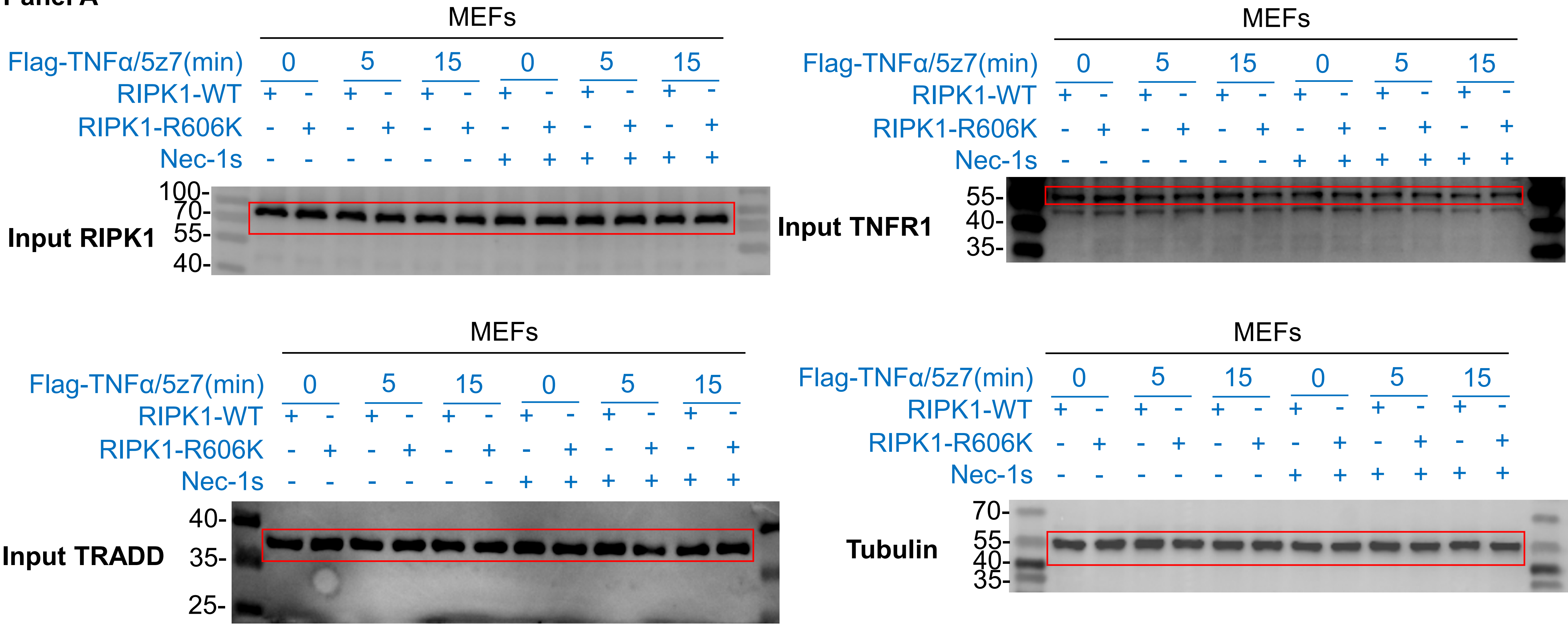

Uncropped blots_Figure 5

Panel B

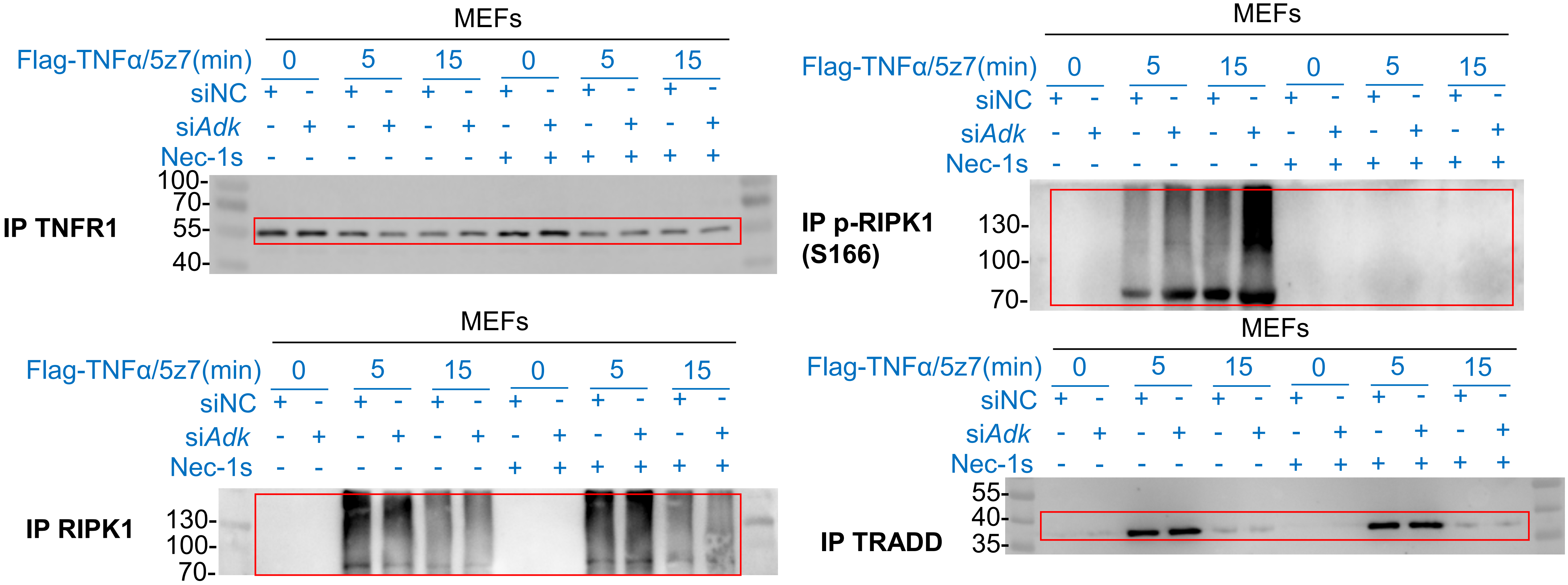

Uncropped blots_Figure 5

Panel B

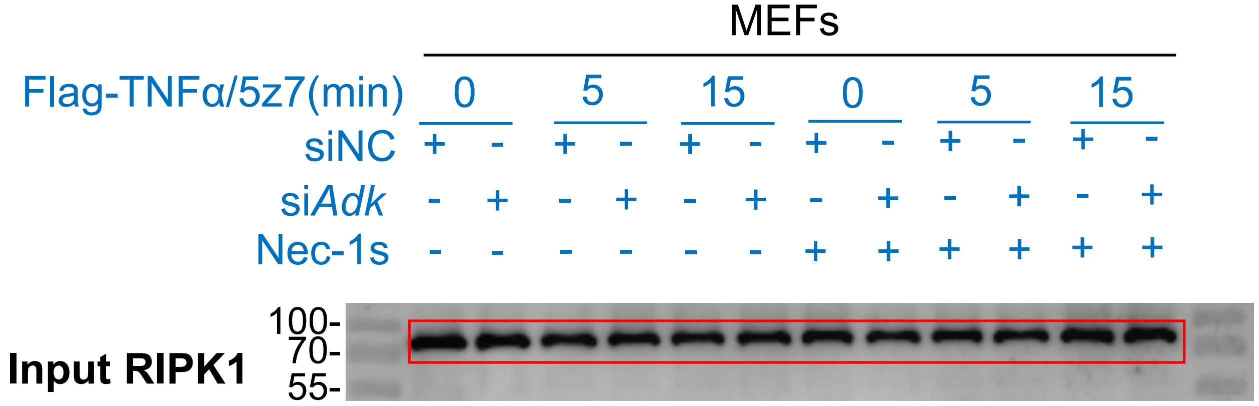

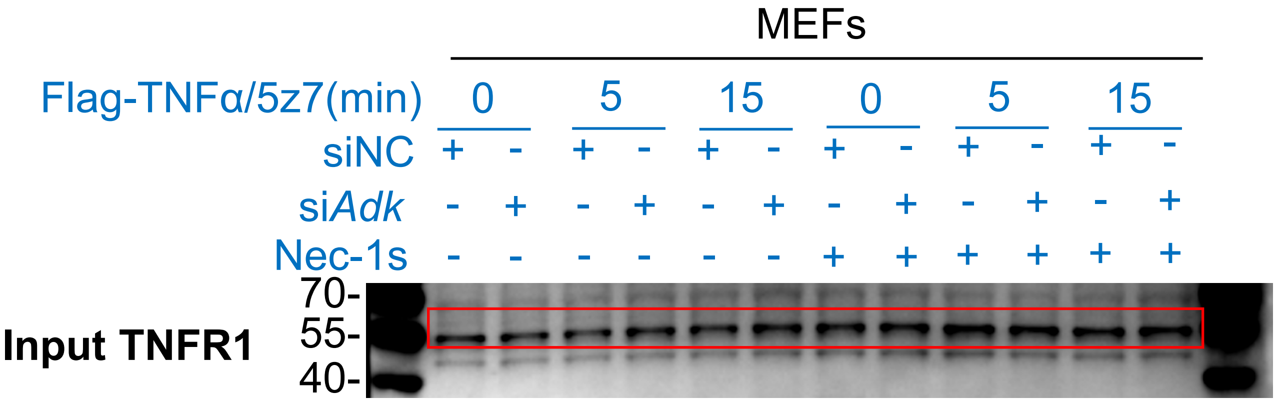

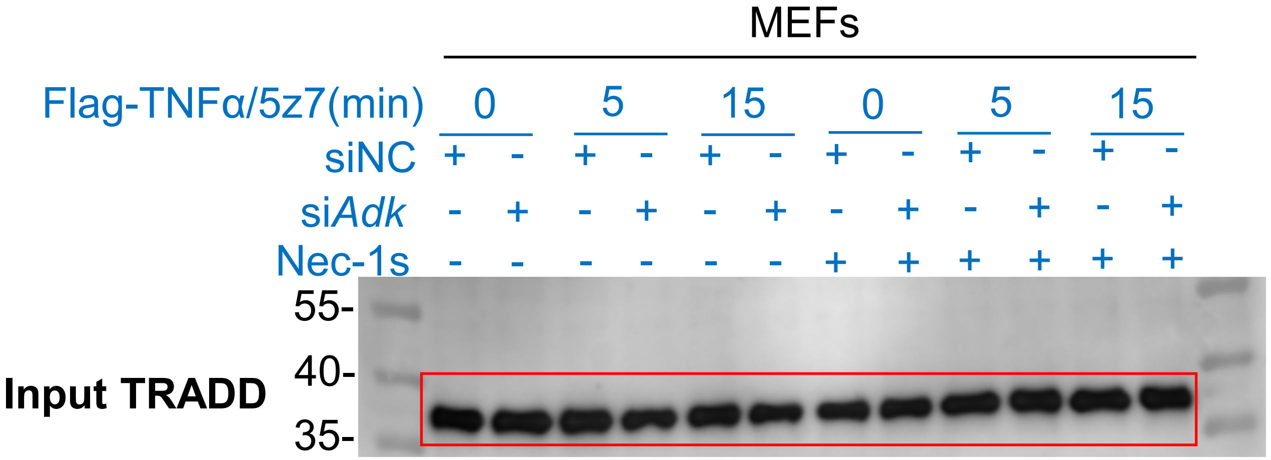

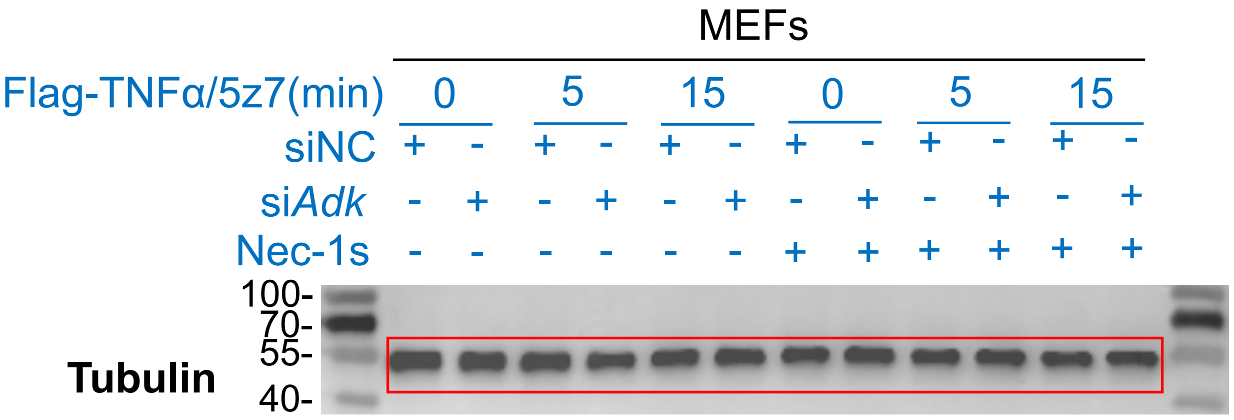

Uncropped blots_Figure 5

Panel C

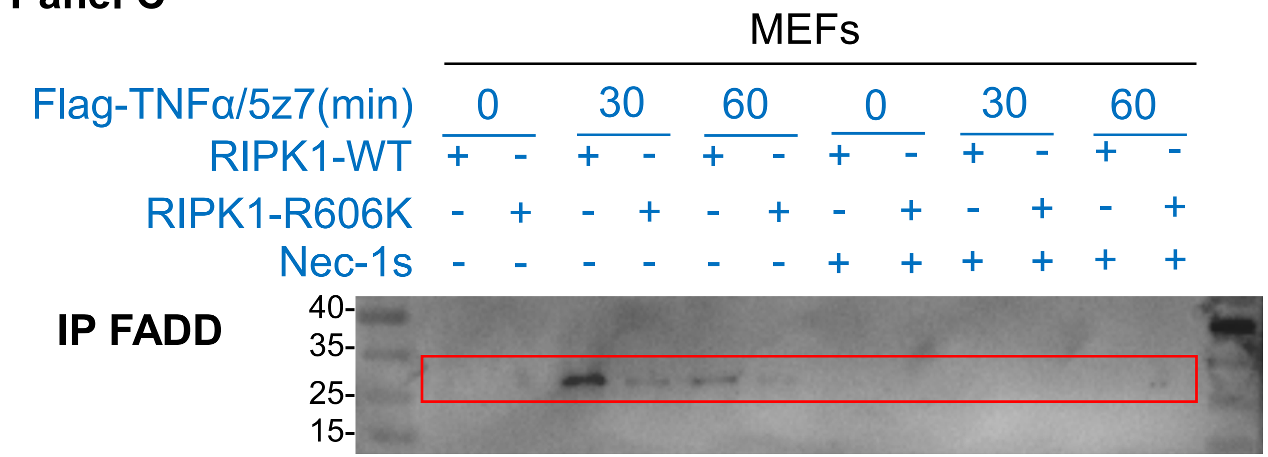

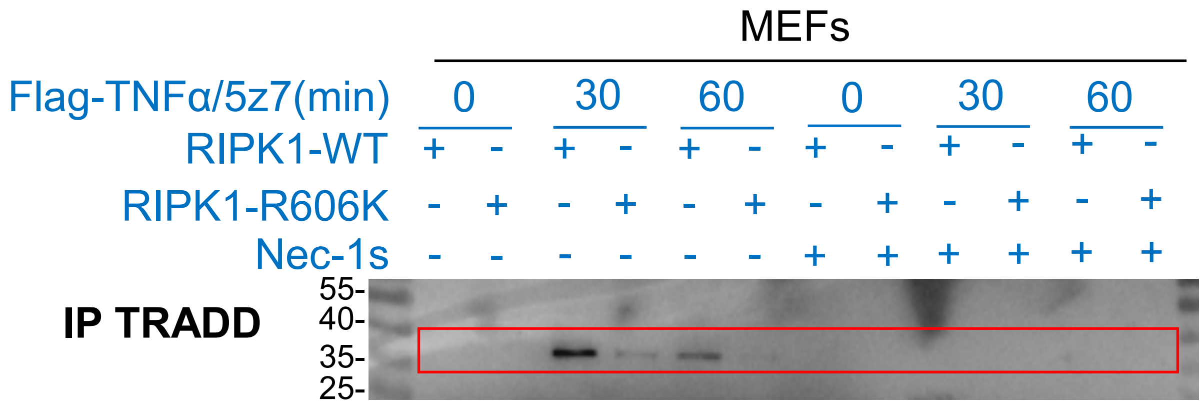

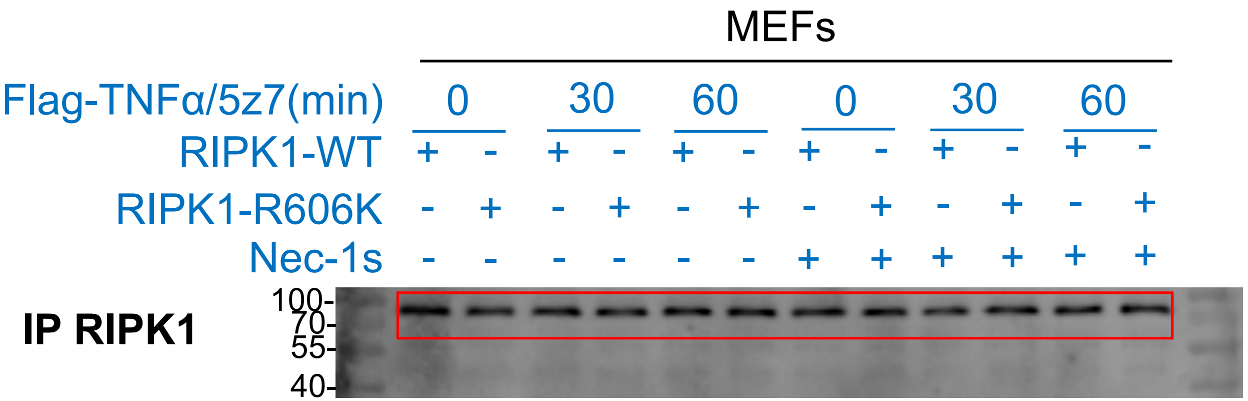

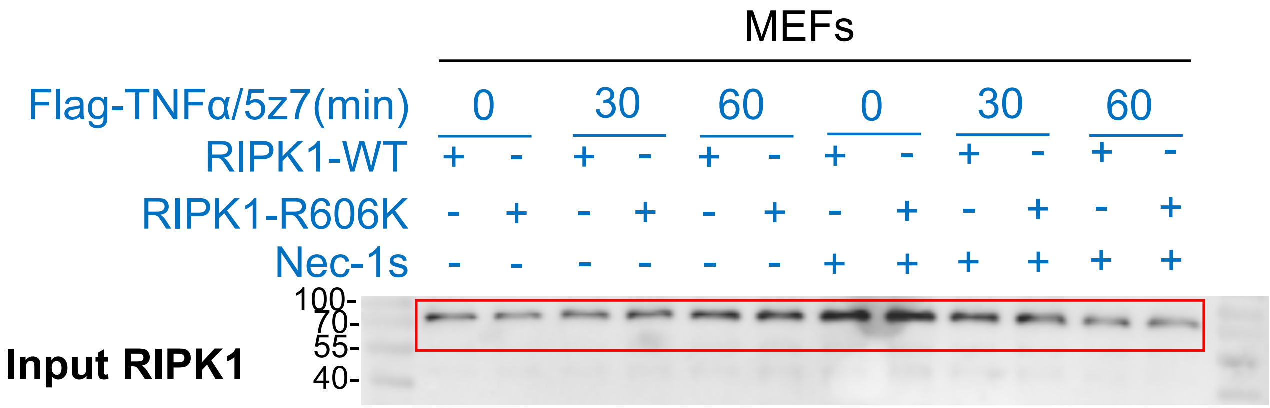

Uncropped blots_Figure 5

Panel C

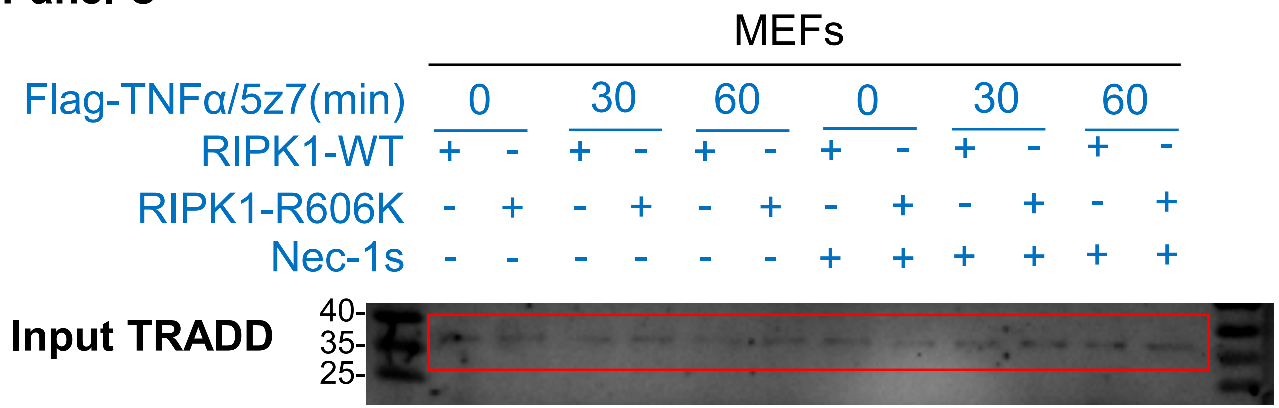

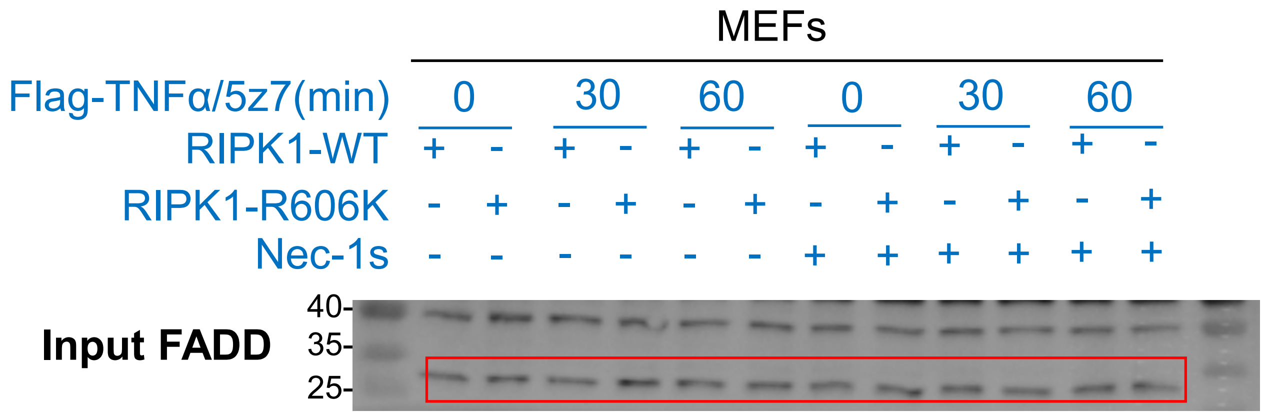

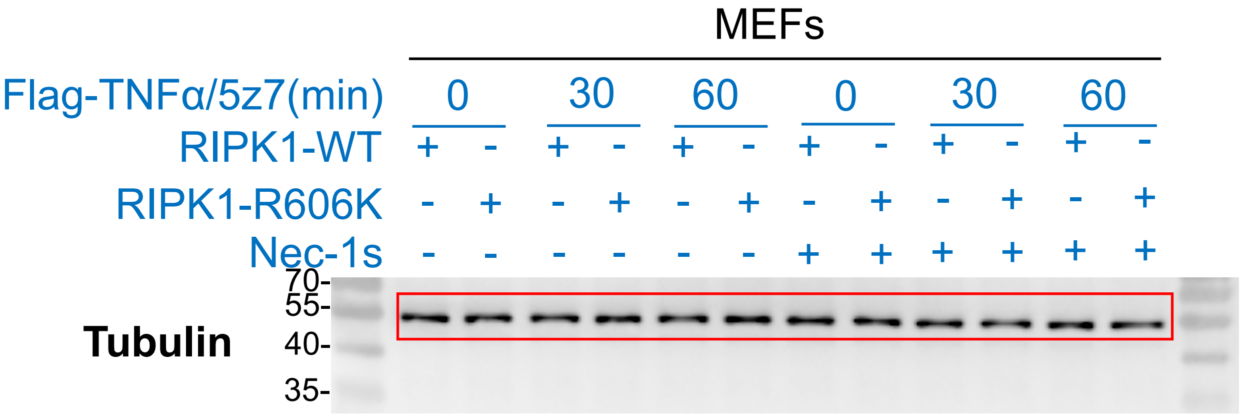

Uncropped blots_Figure 5

Panel D

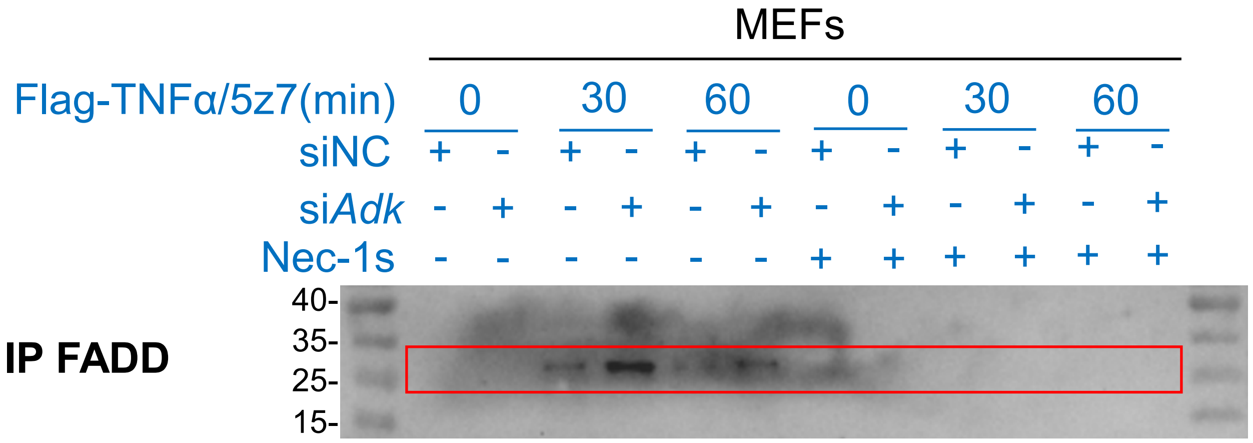

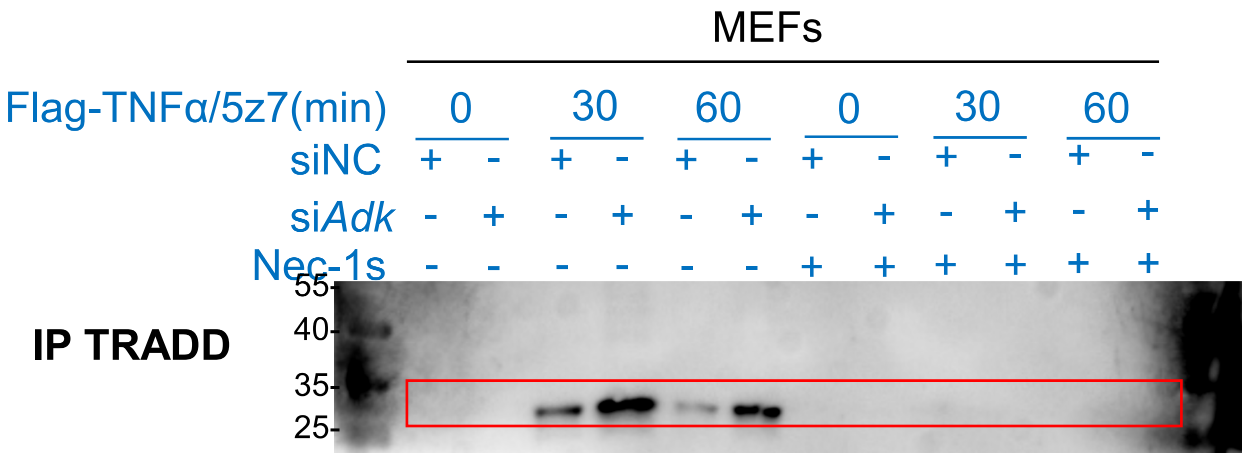

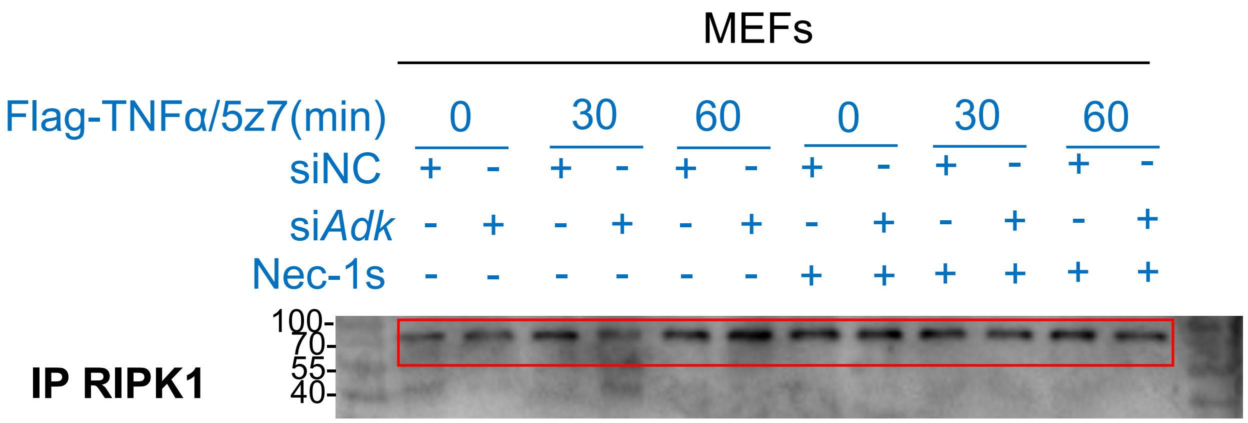

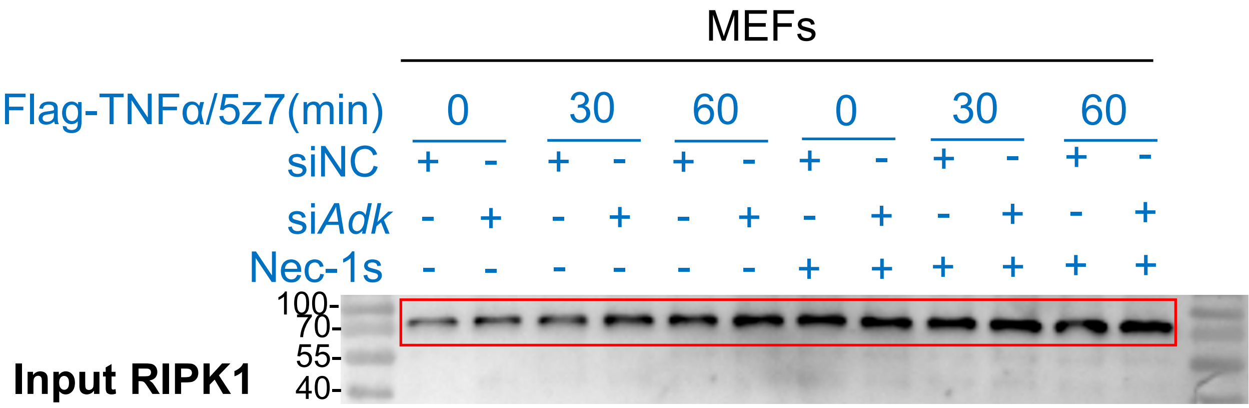

Uncropped blots_Figure 5

Panel D

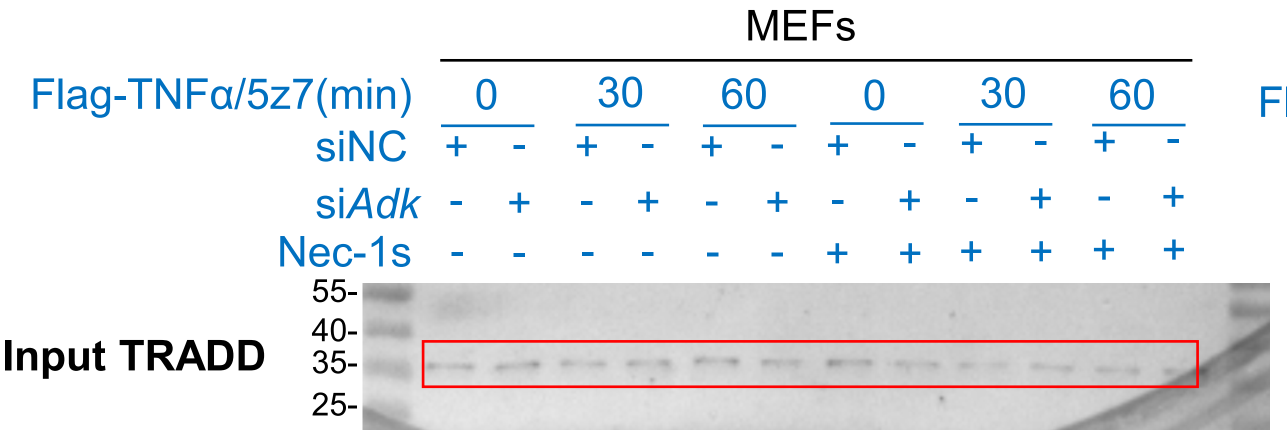

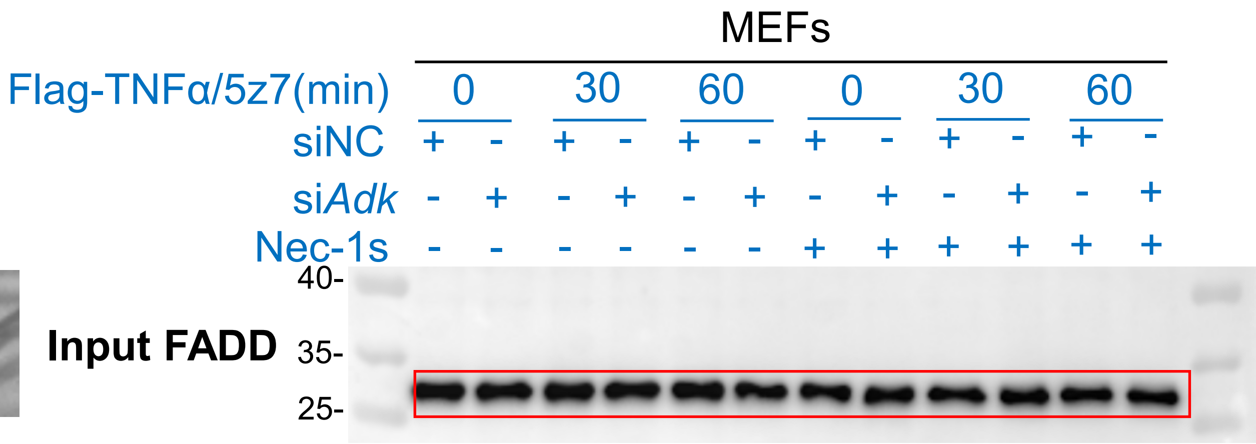

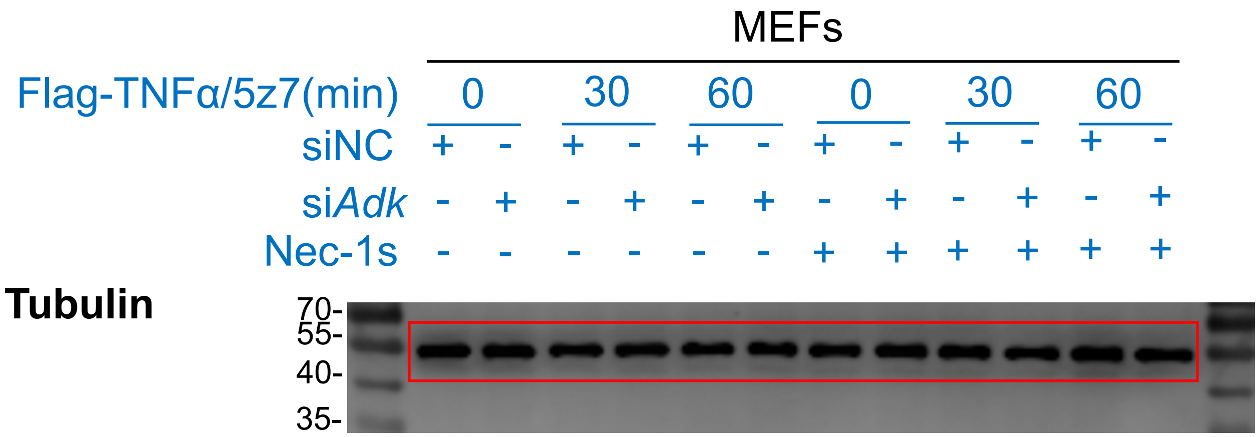

Uncropped blots_Figure 5

Panel F

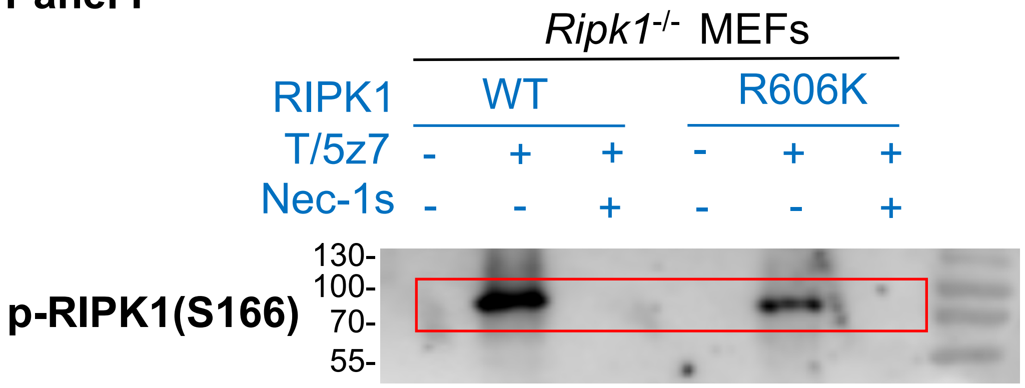

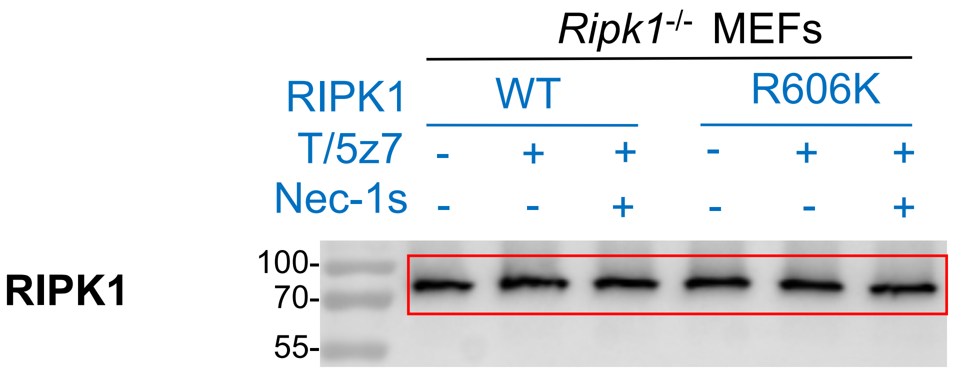

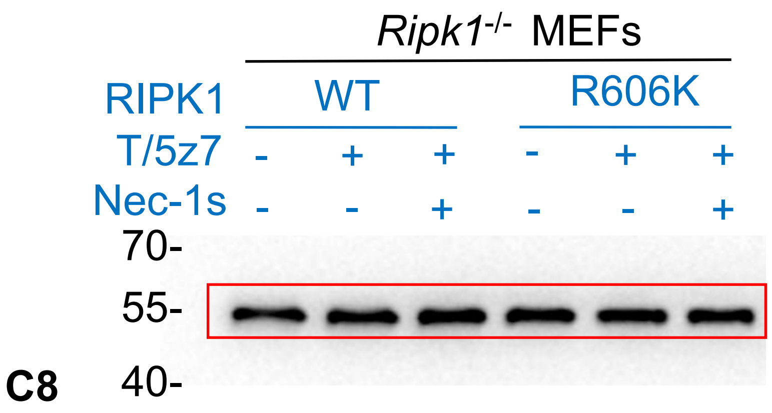

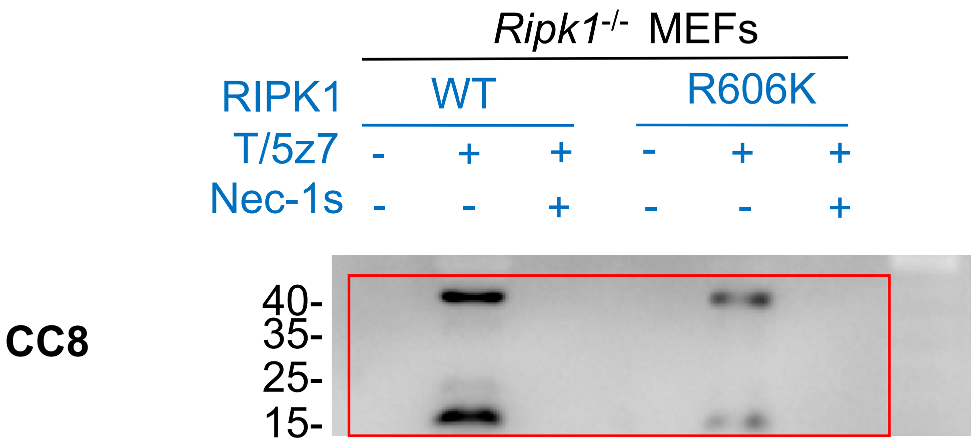

Uncropped blots_Figure 5

Panel F

C3

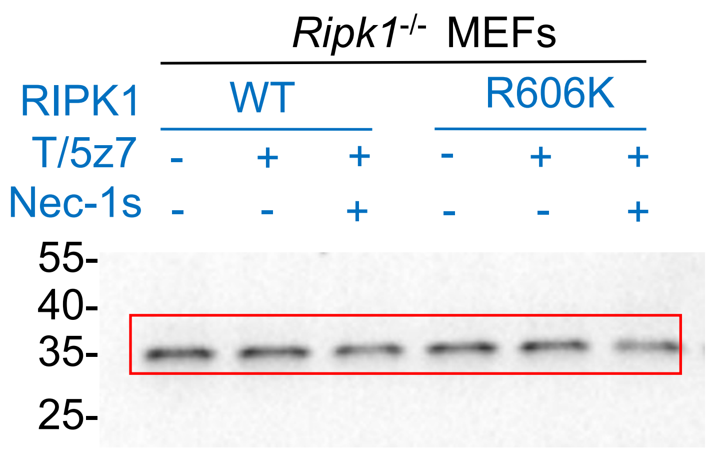

CC3

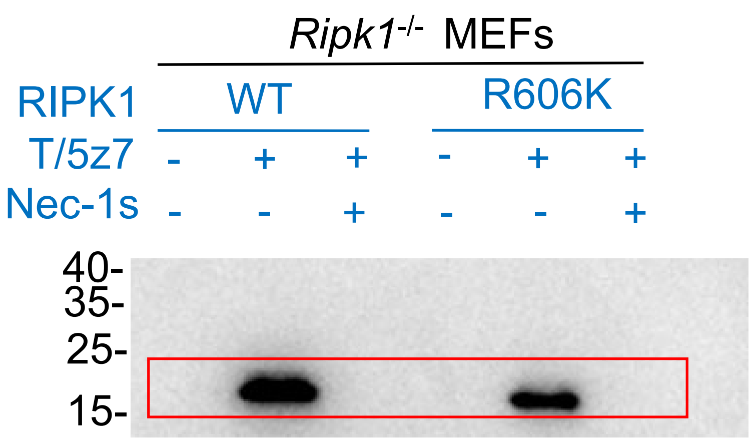

Tubulin

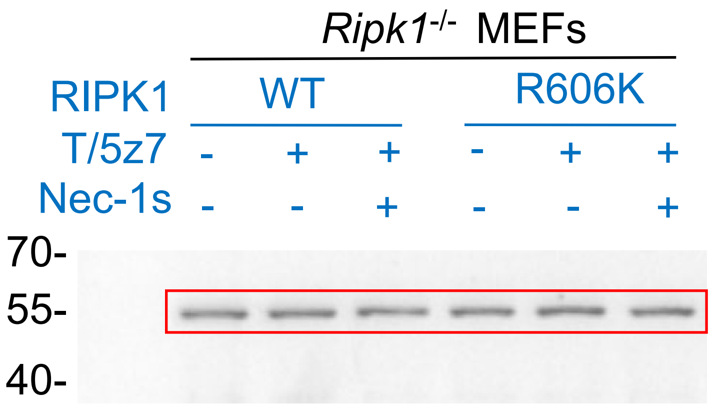

Uncropped blots_Figure 5

Panel H

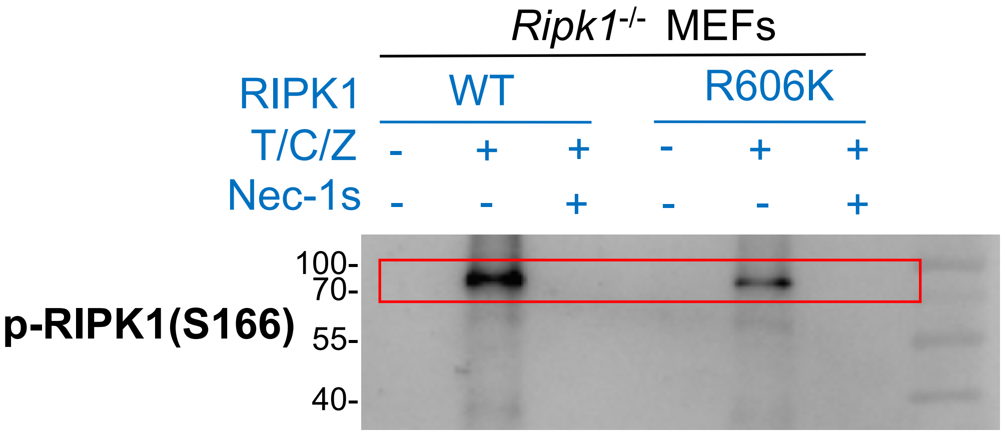

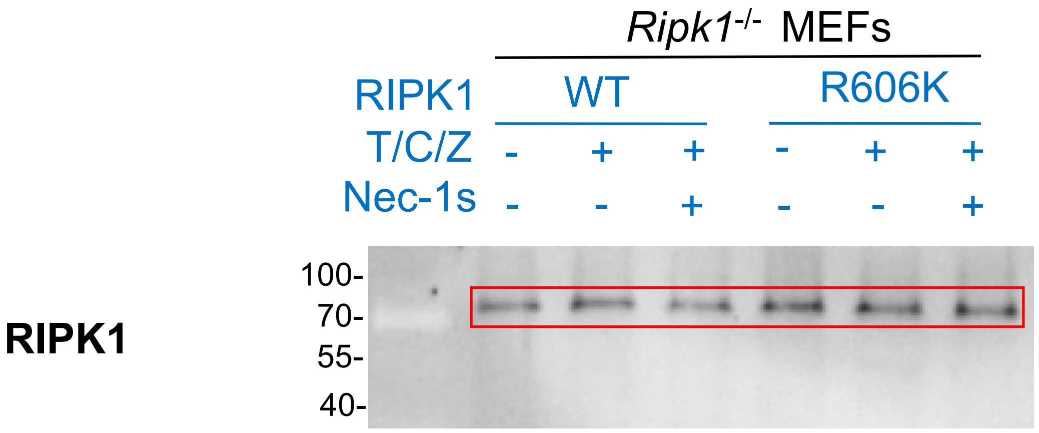

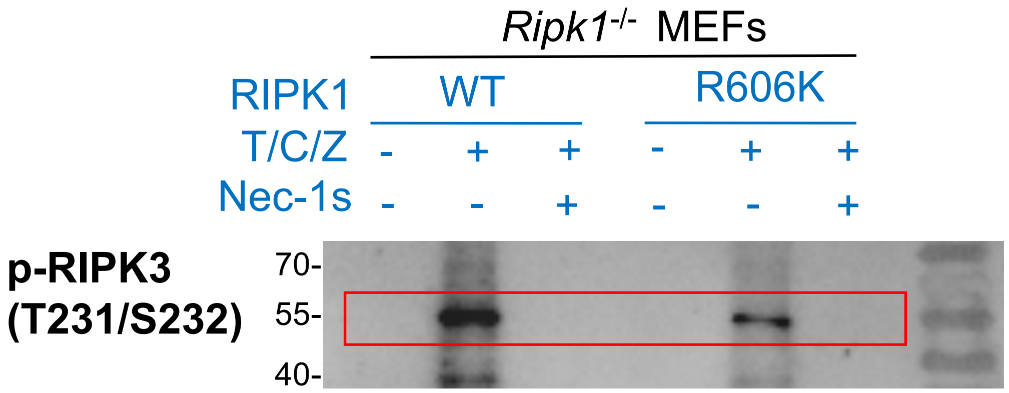

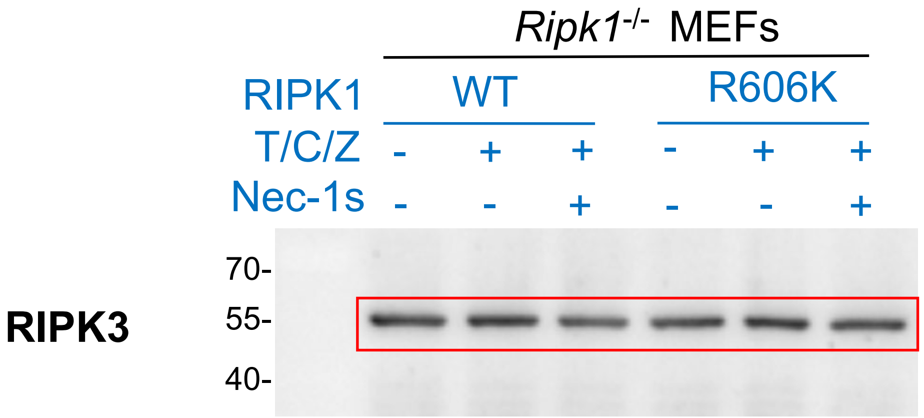

Uncropped blots_Figure 5

Panel H

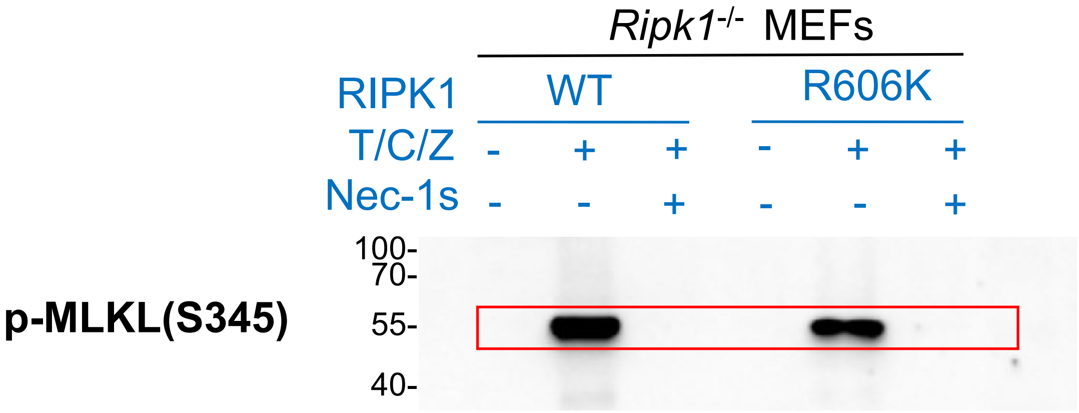

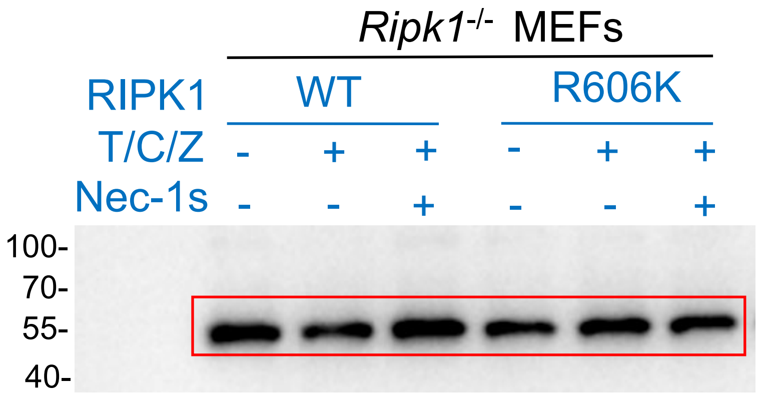

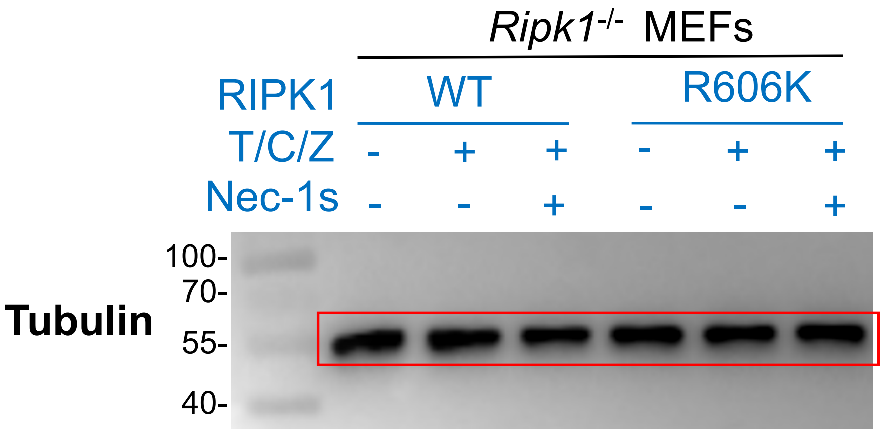

Supplement: SourceData F5 — is the source file for Fig. 5. [file jem_20250603_sourcedataf5.pdf]
