# Supplementary material for: Adenosine metabolic clearance maintains liver homeostasis by licensing arginine methylation of RIPK1
Source: J Exp Med. 2025 Oct 13;223(1):e20250603. doi: 10.1084/jem.20250603 (PMC12517274; doi:10.1084/jem.20250603)

Uncropped blots_Figure 6

Panel B

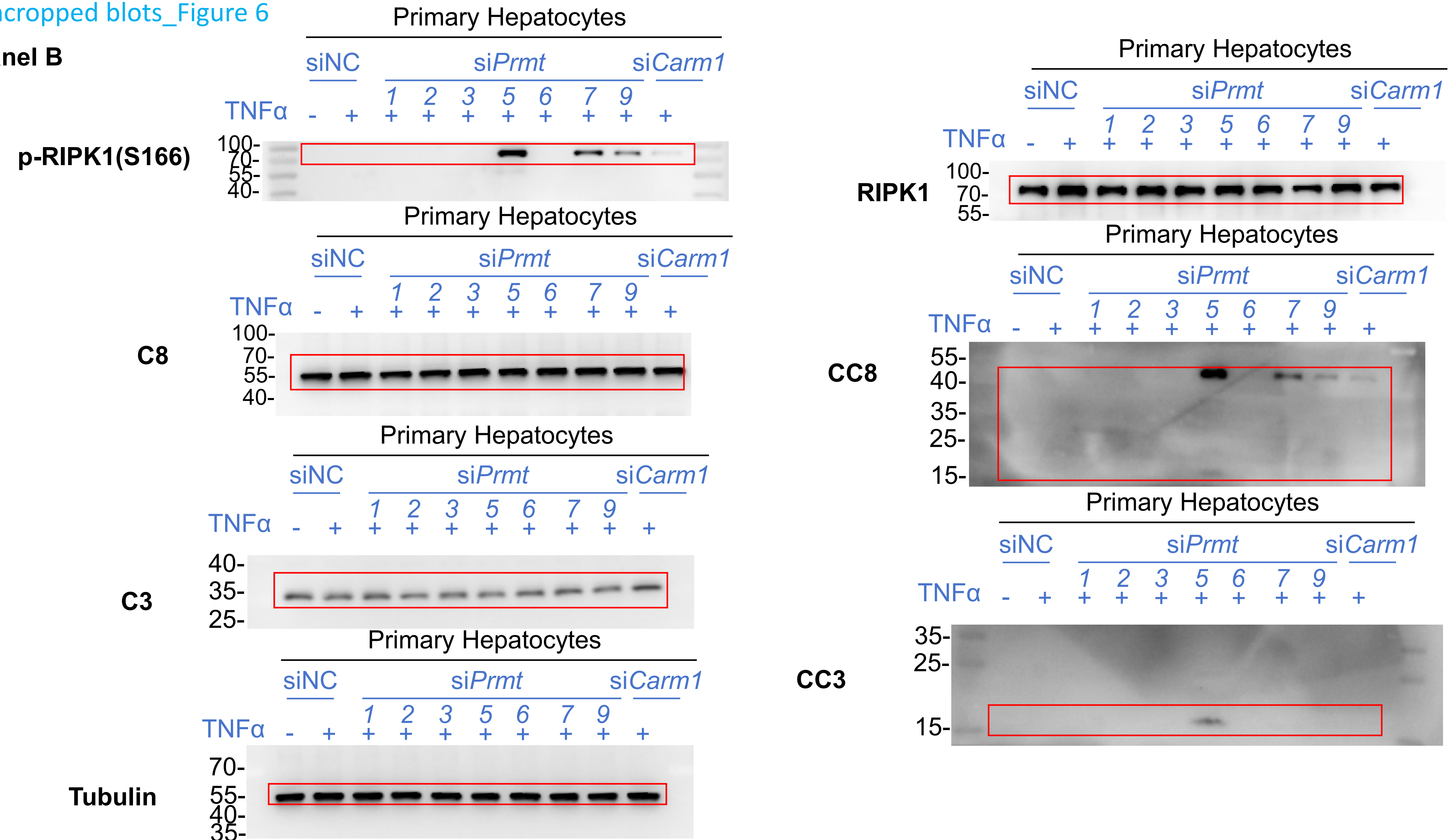

Uncropped blots_Figure 6

Panel C

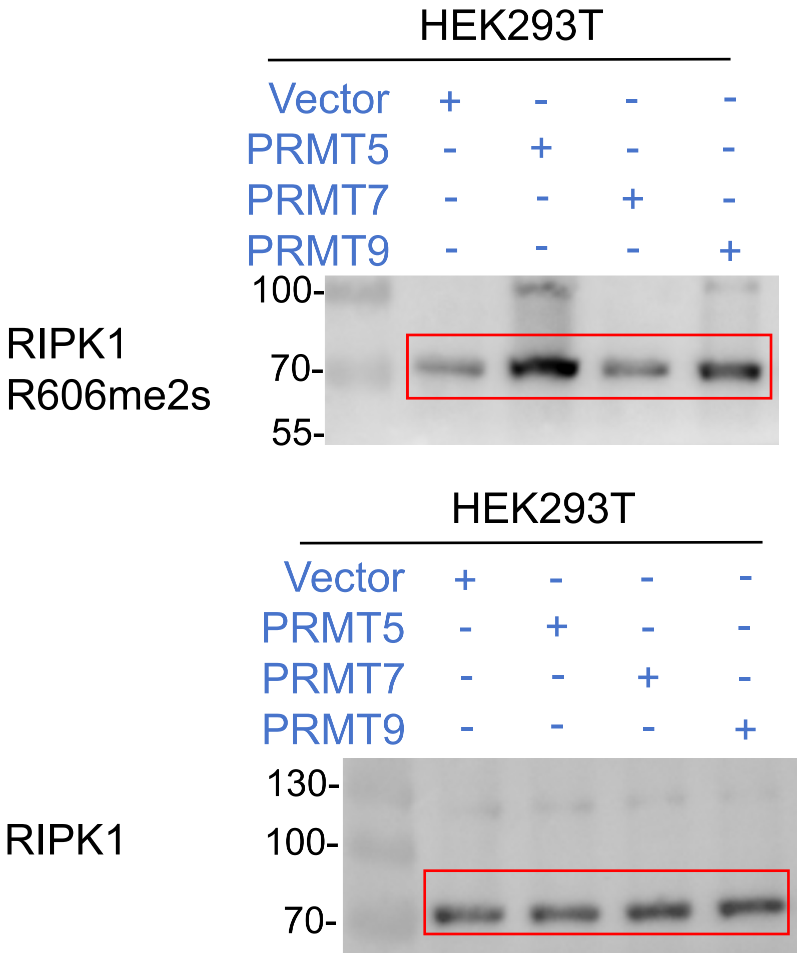

Panel D

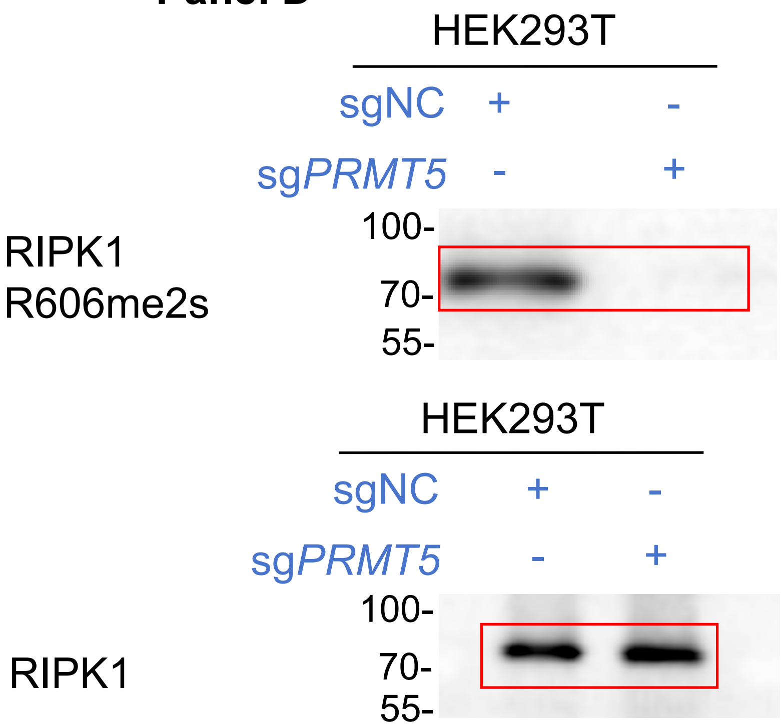

Uncropped blots_Figure 6

Panel E

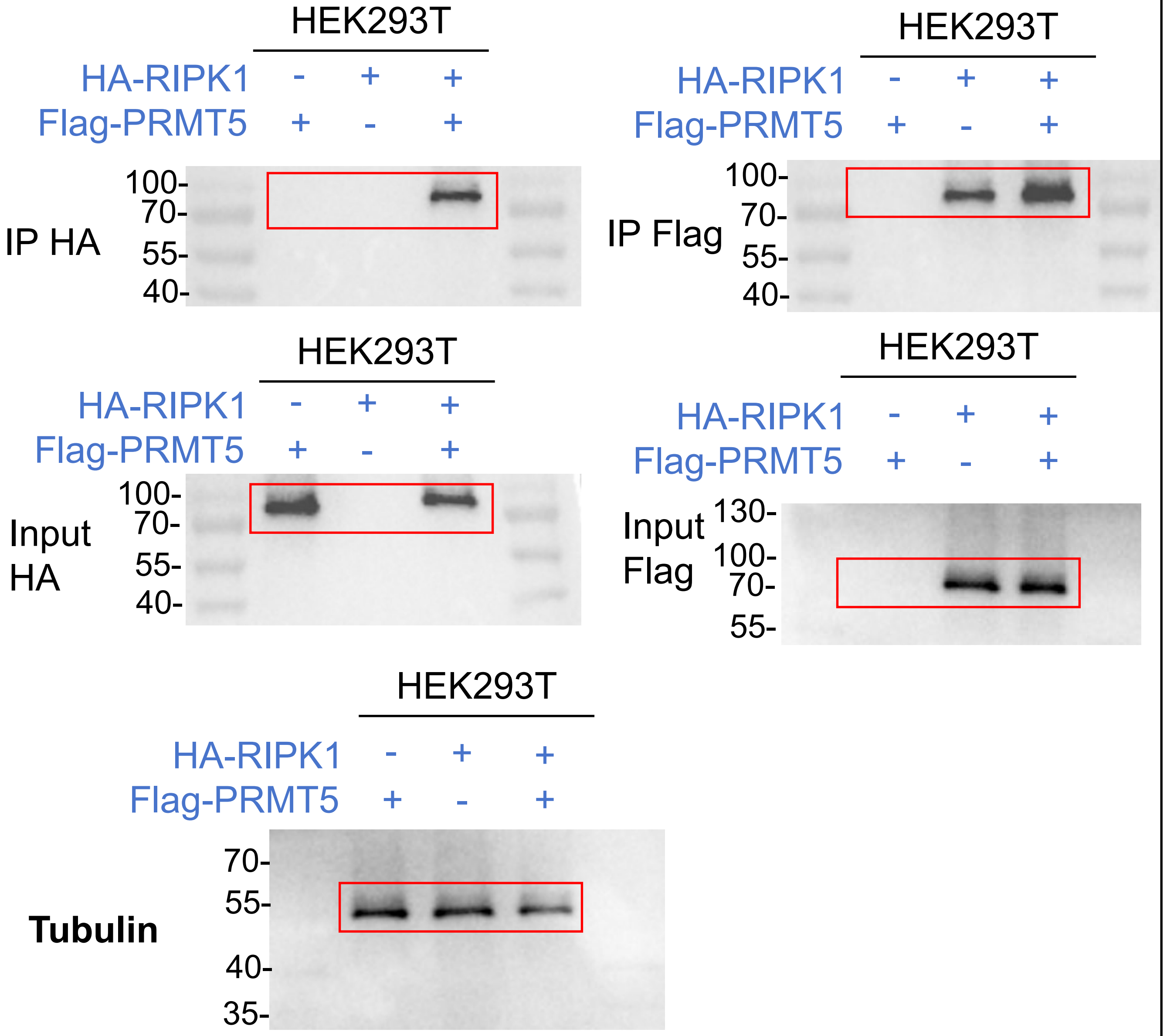

Panel F

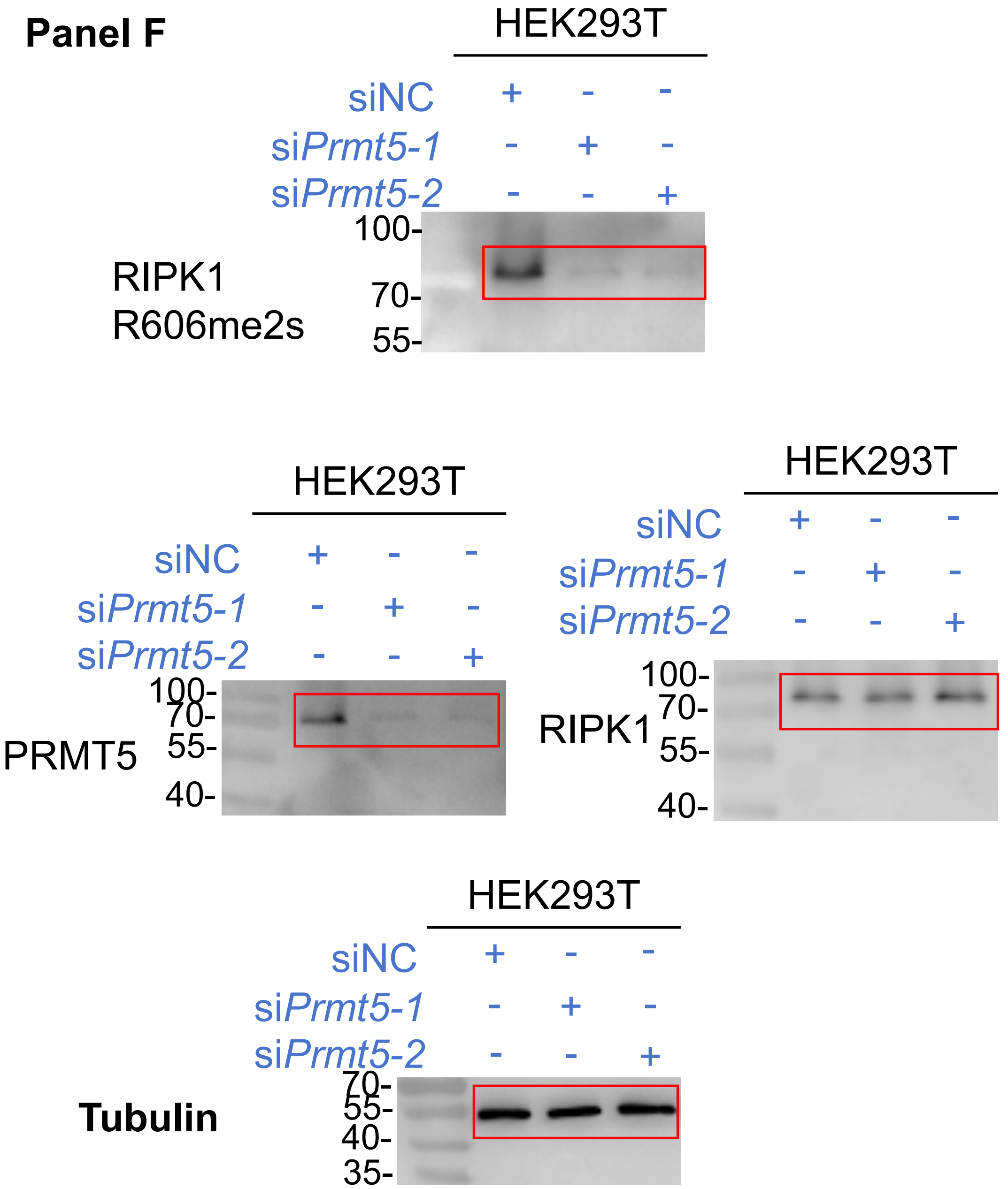

Uncropped blots_Figure 6

Panel G

PRMT5-WT	+	+	-	+	-	+	+
PRMT5-G367A/R368A	-	-	-	-	+	-	-
EPZ015666	-	-	-	-	-	+	-
MEP50	+	+	+	+	+	+	+
SAM	+	+	+	-	+	+	+
RIPK1-WT	-	+	+	+	+	+	-
RIPK1-R606K	-	-	-	-	-	-	+

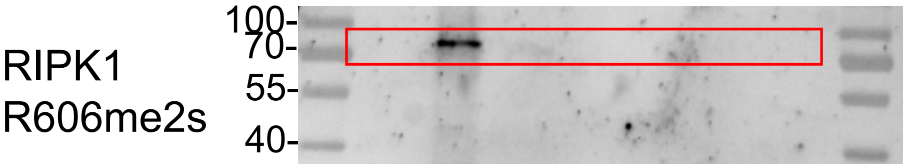

PRMT5-WT	+	+	-	+	-	+	+
PRMT5-G367A/R368A	-	-	-	-	+	-	-
EPZ015666	-	-	-	-	-	+	-
MEP50	+	+	+	+	+	+	+
SAM	+	+	+	-	+	+	+
RIPK1-WT	-	+	+	+	+	+	-
RIPK1-R606K	-	-	-	-	-	-	+

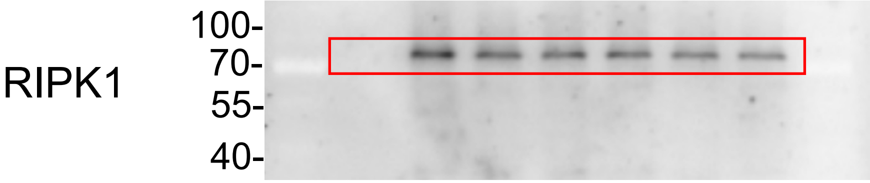

PRMT5-WT	+	+	-	+	-	+	+
PRMT5-G367A/R368A	-	-	-	-	+	-	-
EPZ015666	-	-	-	-	-	+	-
MEP50	+	+	+	+	+	+	+
SAM	+	+	+	-	+	+	+
RIPK1-WT	-	+	+	+	+	+	-
RIPK1-R606K	-	-	-	-	-	-	+

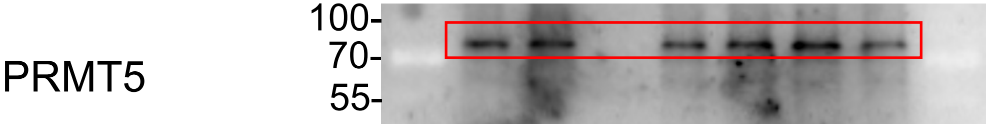

Uncropped blots_Figure 6

Panel I

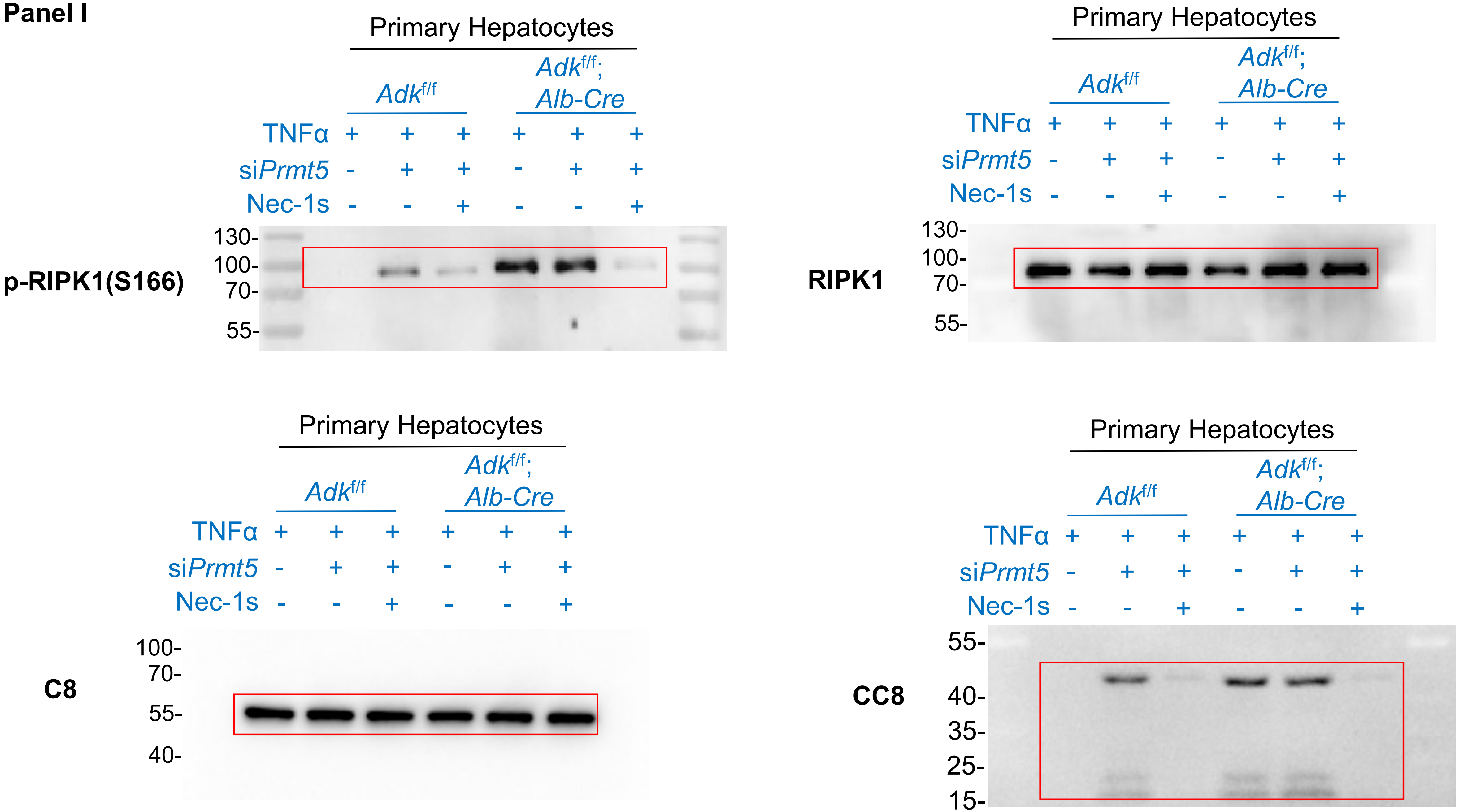

Uncropped blots_Figure 6

Panel I

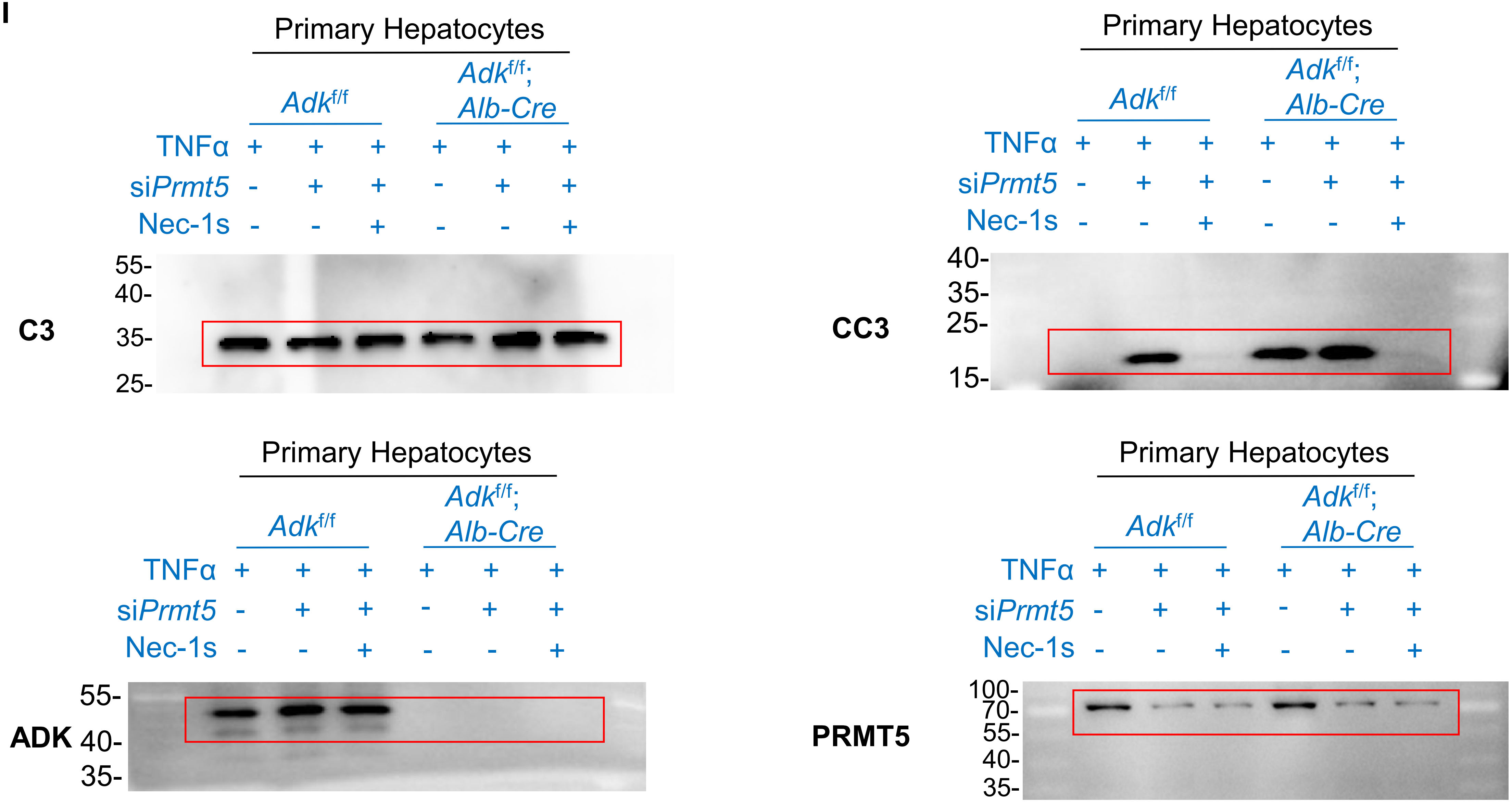

Uncropped blots_Figure 6

Panel I

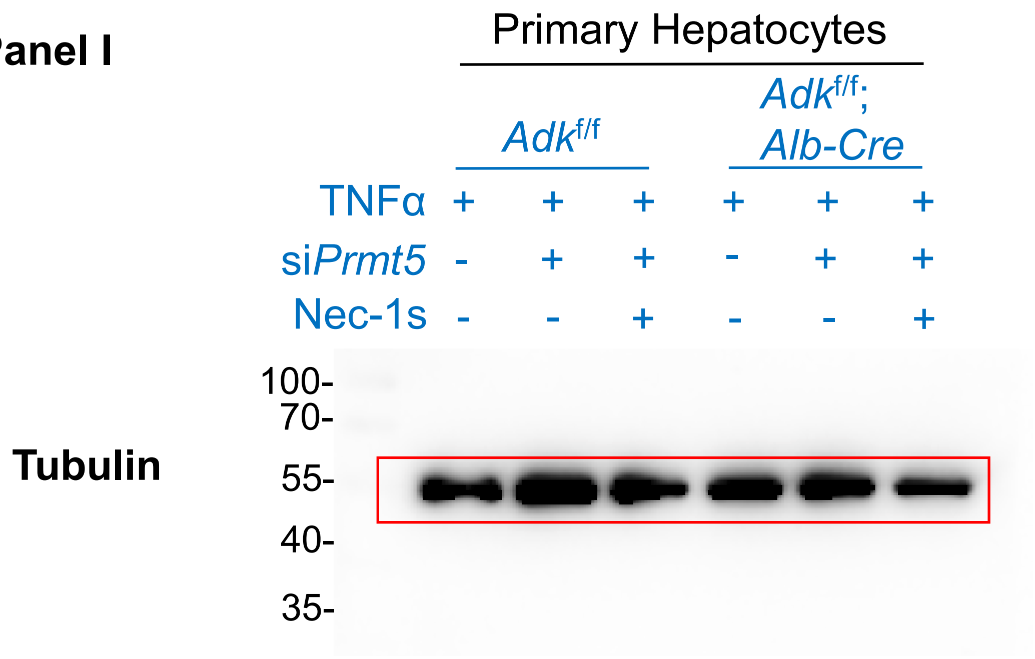

Uncropped blots_Figure 6

Panel K

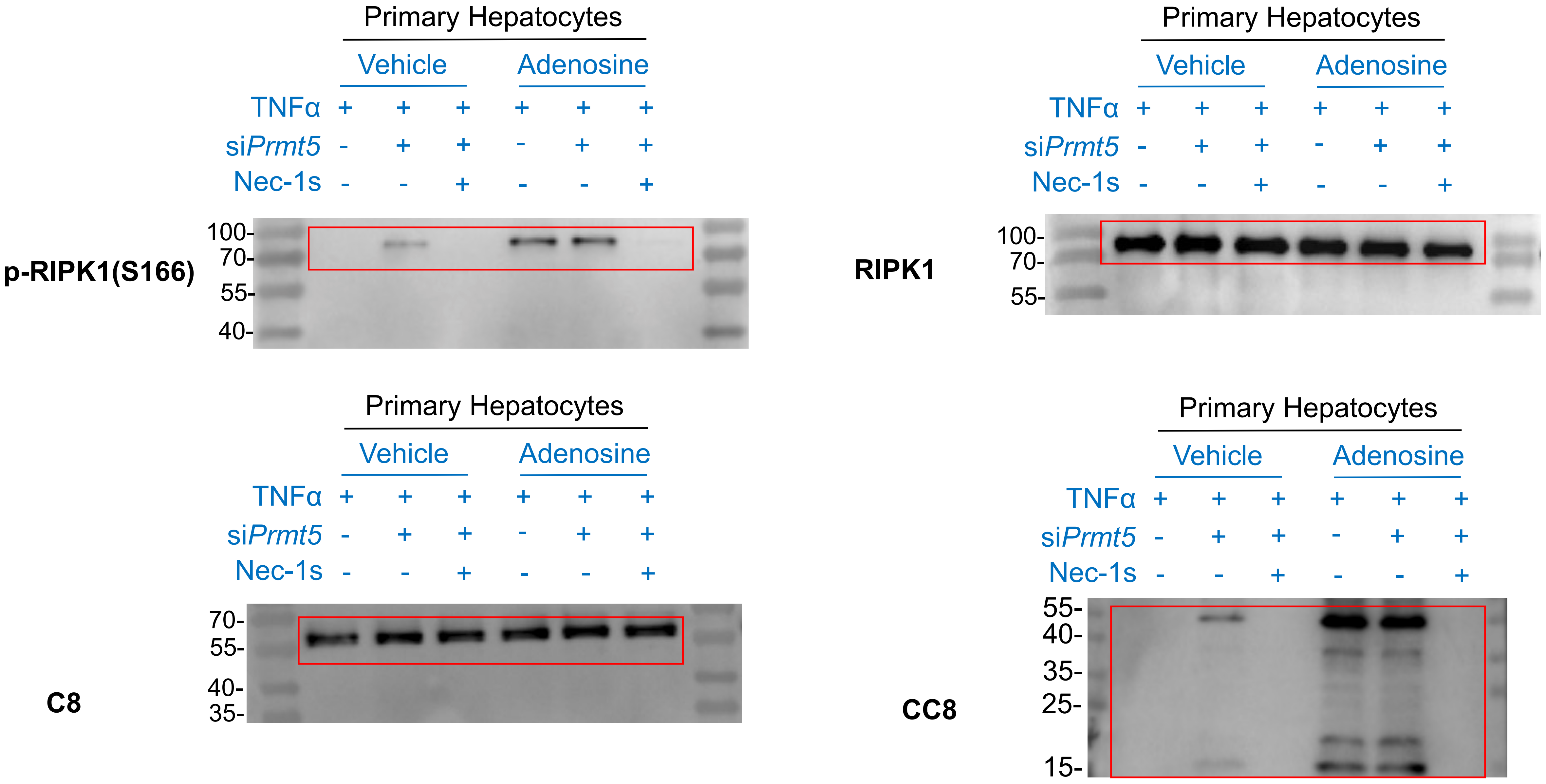

Uncropped blots_Figure 6

Panel K

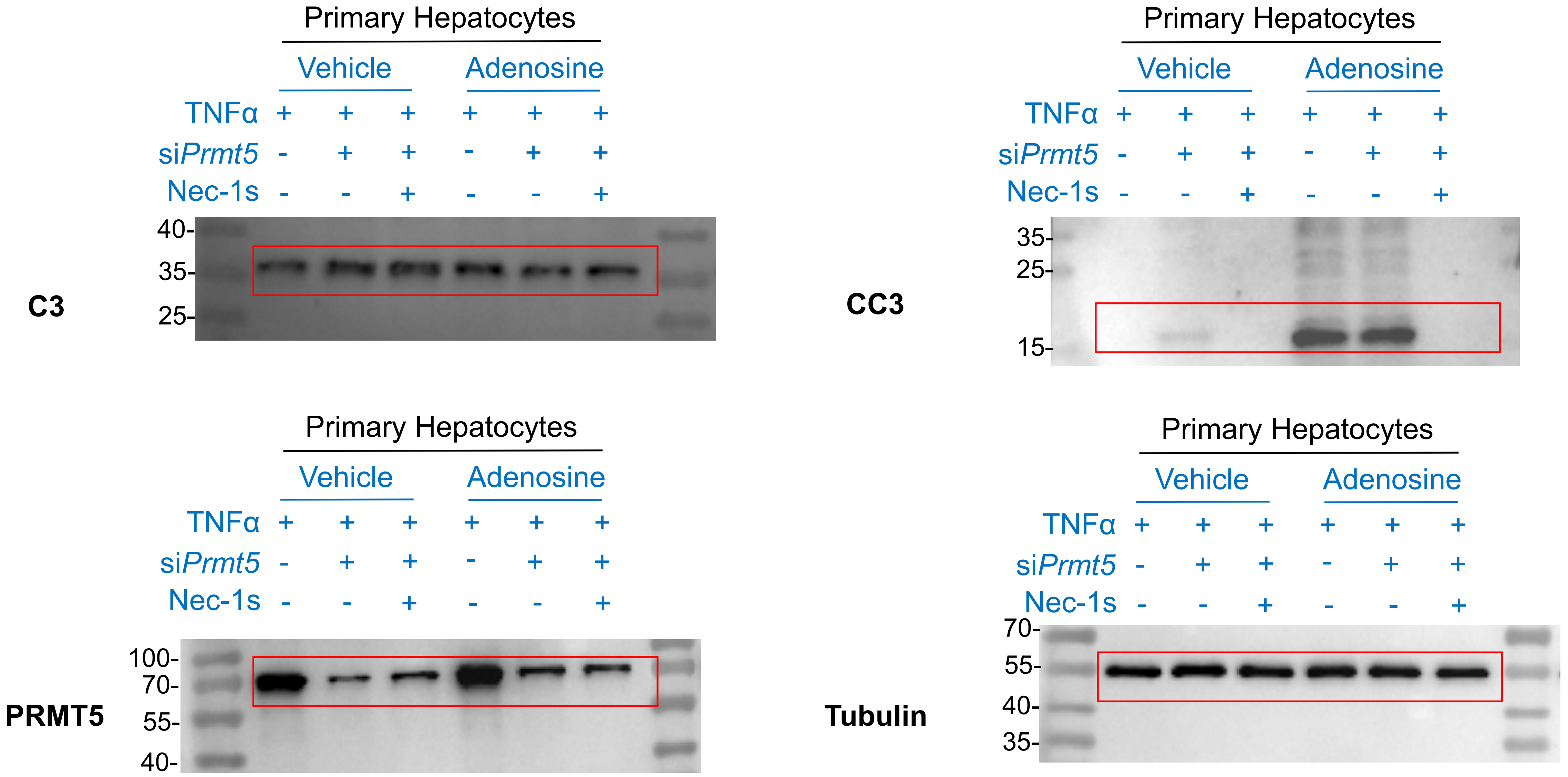

Supplement: SourceData F6 — is the source file for Fig. 6. [file jem_20250603_sourcedataf6.pdf]
