# Supplementary figures and images for: Adenosine metabolic clearance maintains liver homeostasis by licensing arginine methylation of RIPK1
Source: J Exp Med. 2025 Oct 13;223(1):e20250603. doi: 10.1084/jem.20250603 (PMC12517274; doi:10.1084/jem.20250603)

Panel A

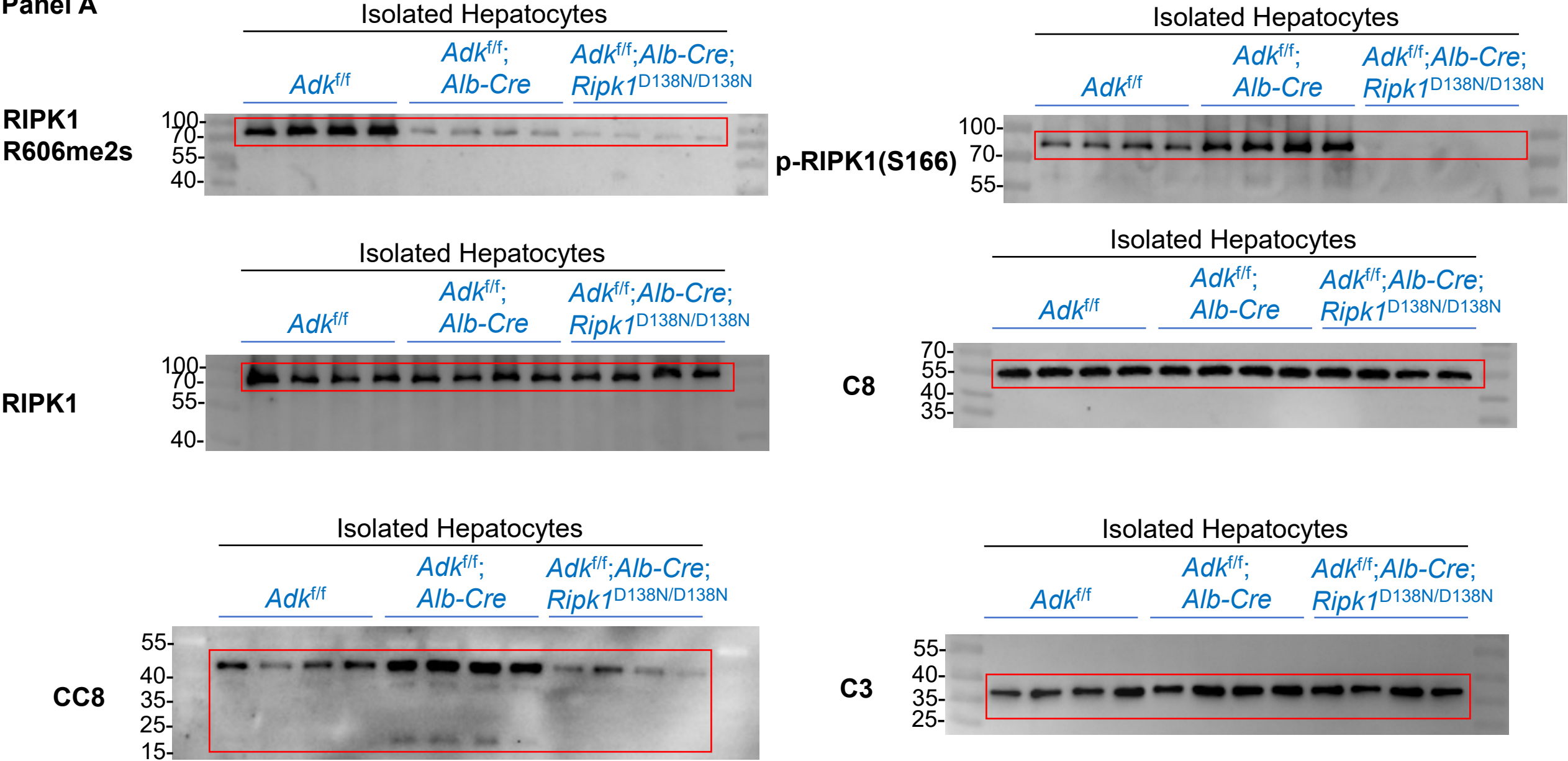

Panel A

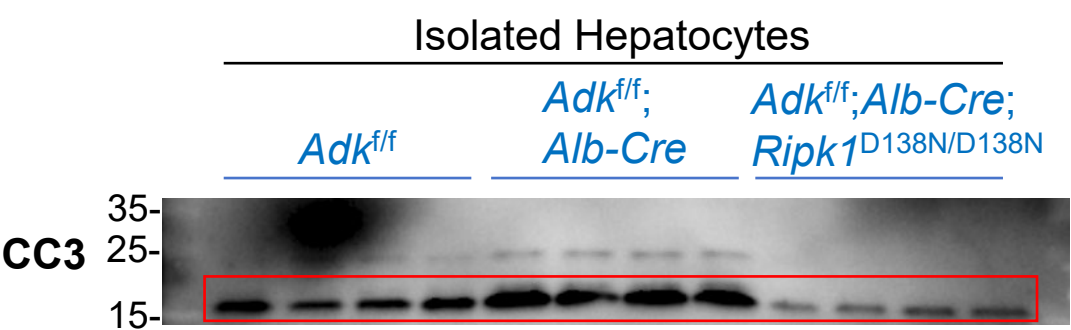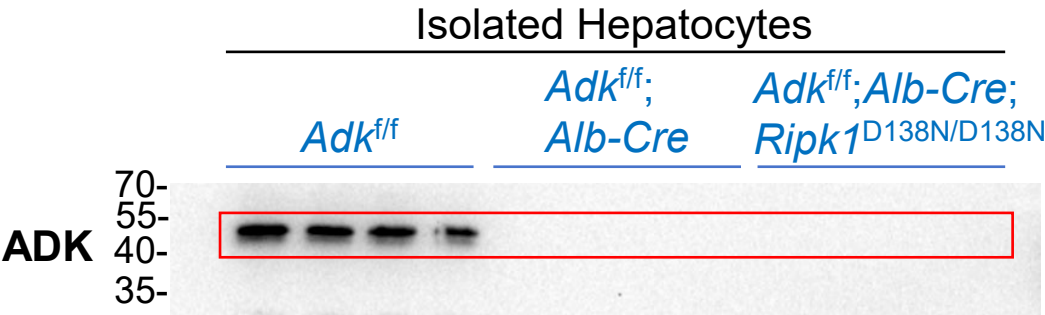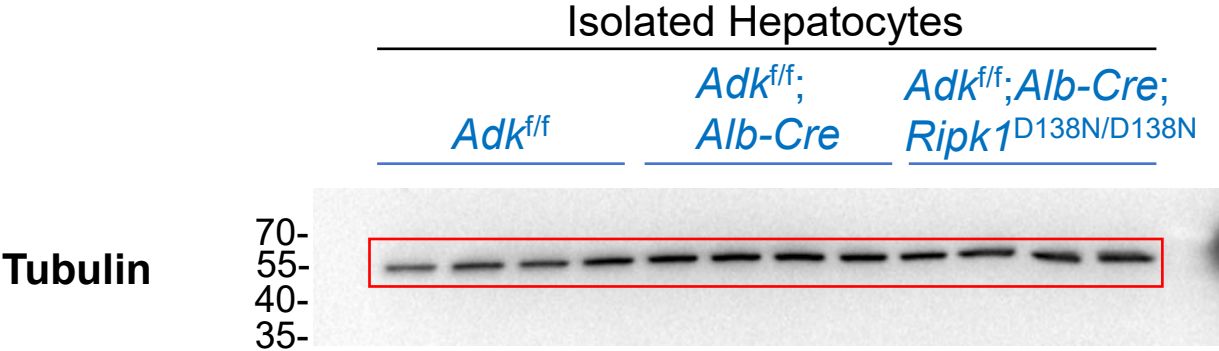

Panel E

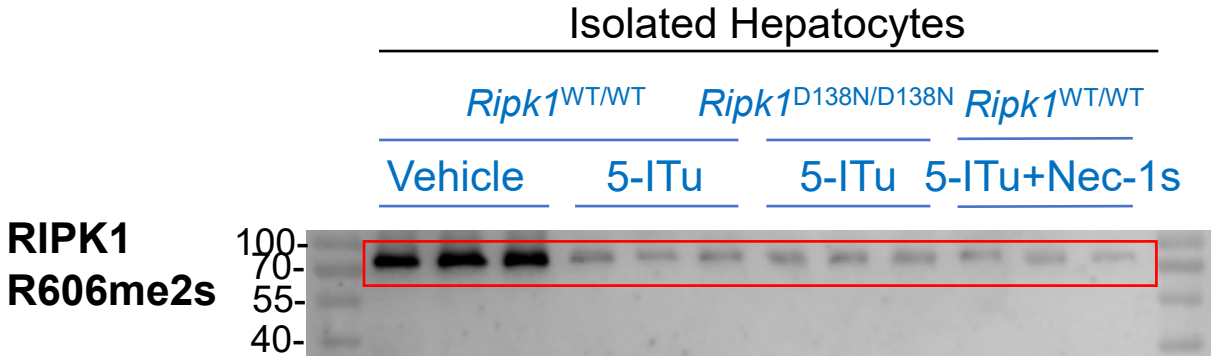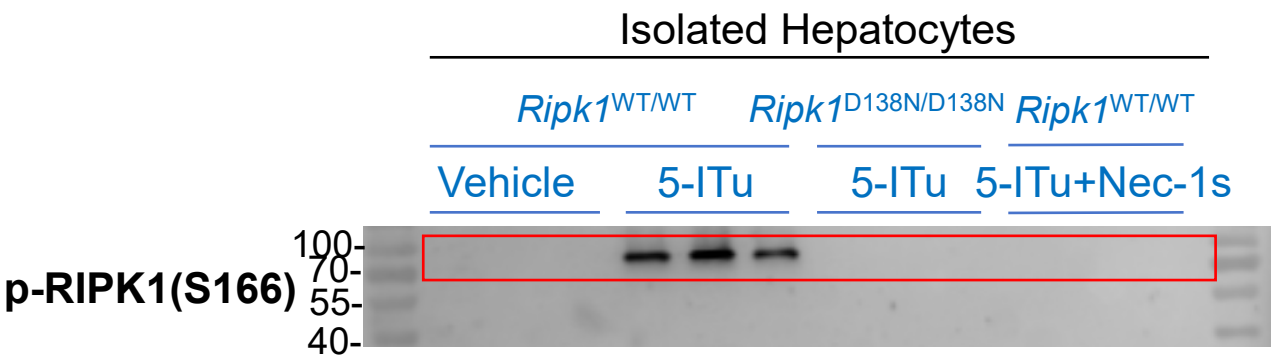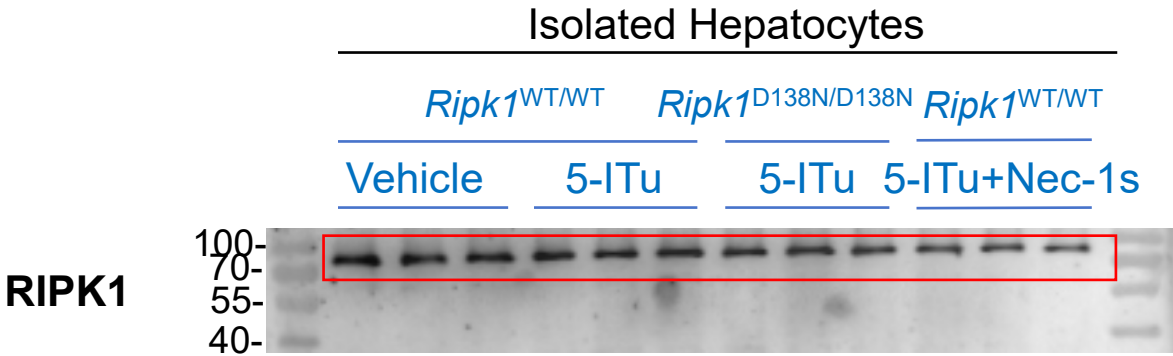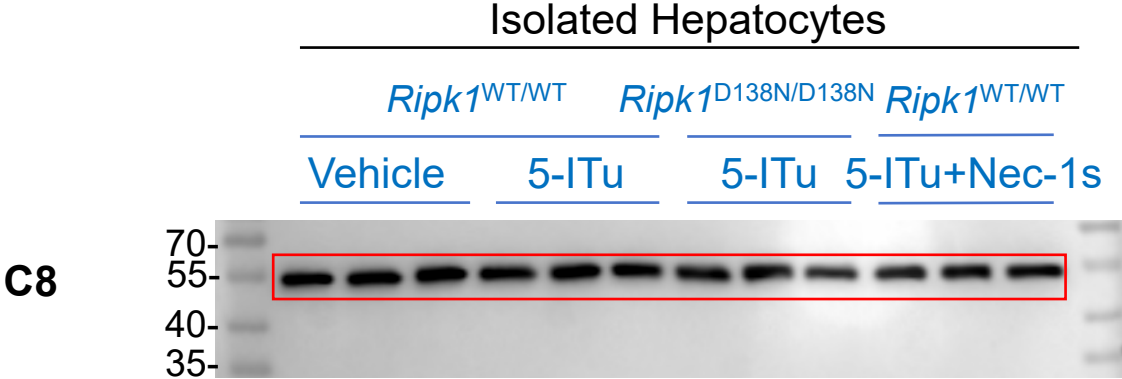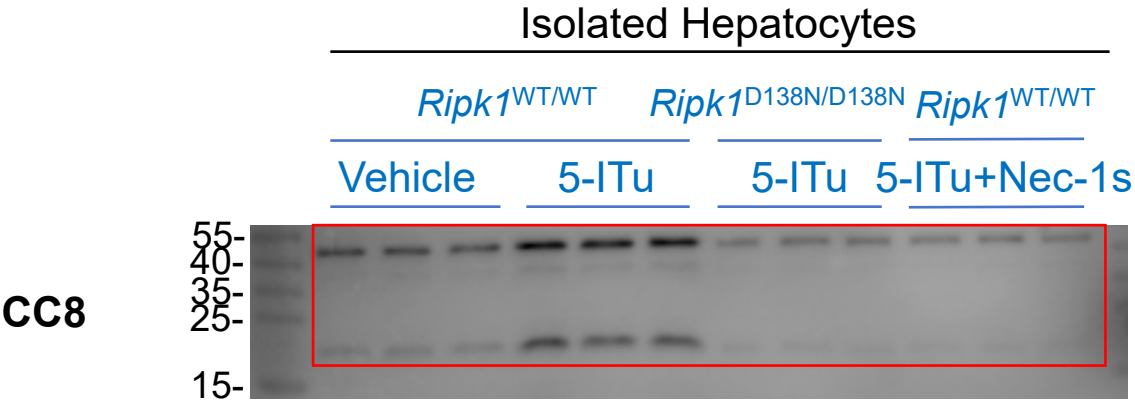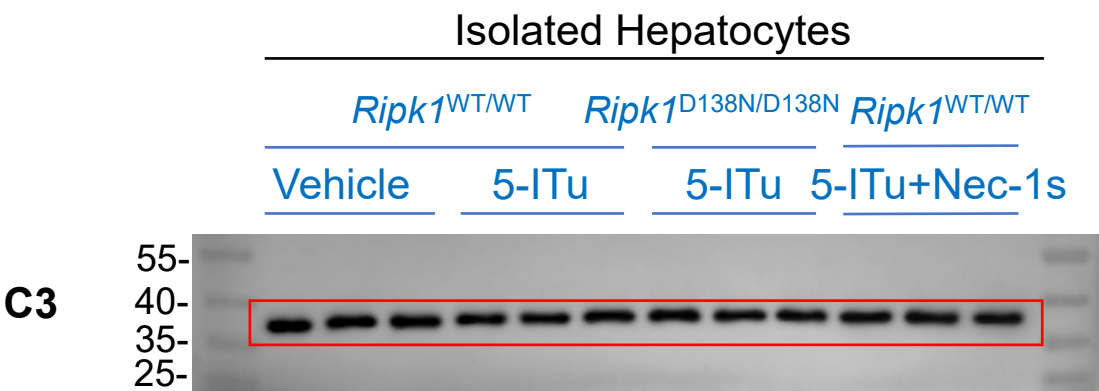

Panel E

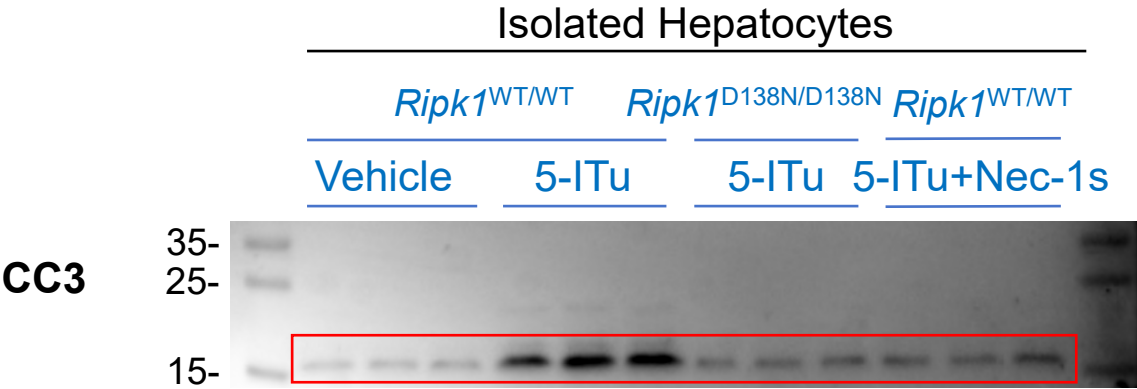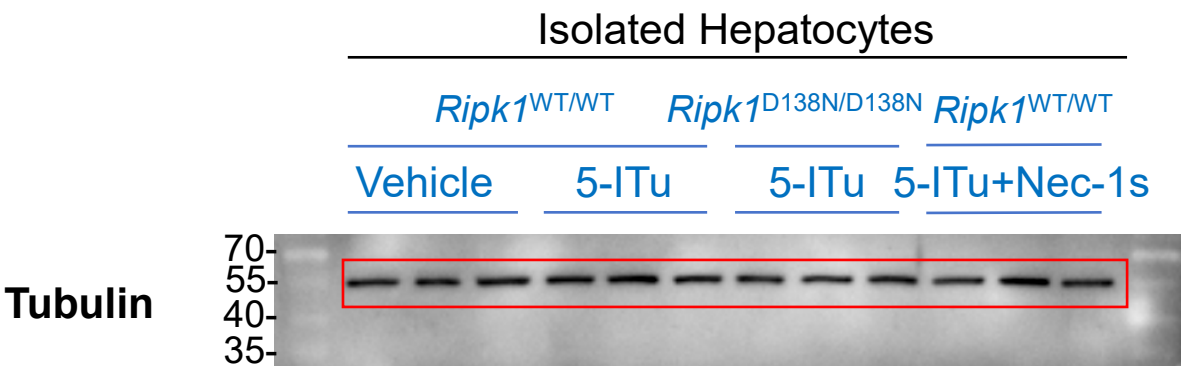

Supplement: SourceData F7 — is the source file for Fig. 7. [file jem_20250603_sourcedataf7.pdf]

Panel C

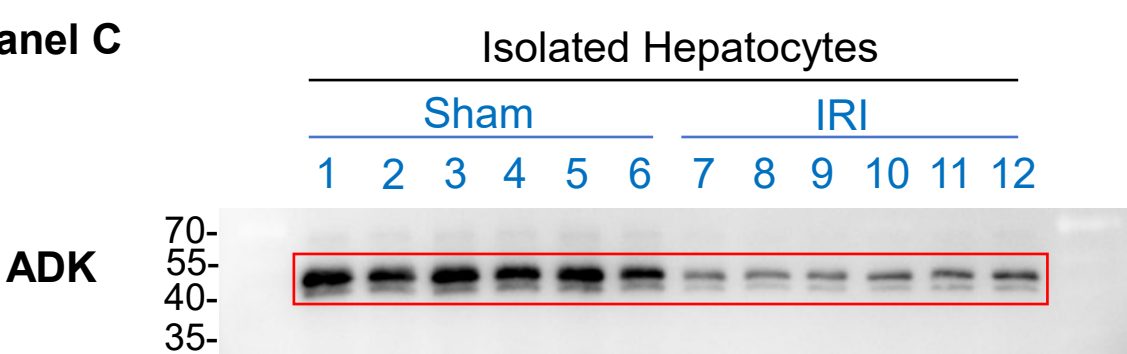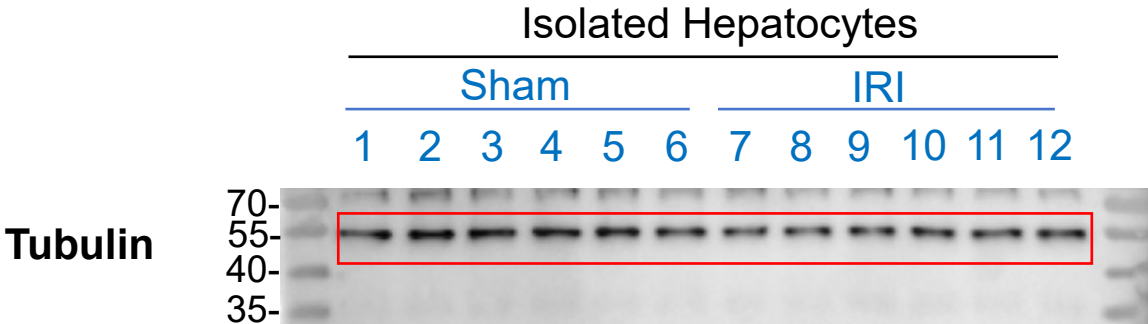

Panel E

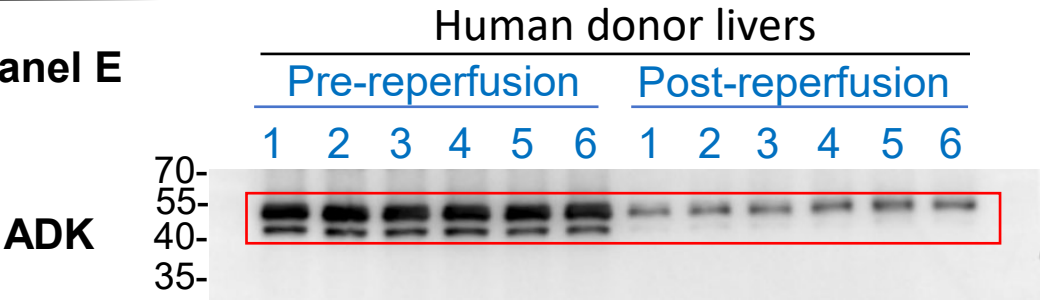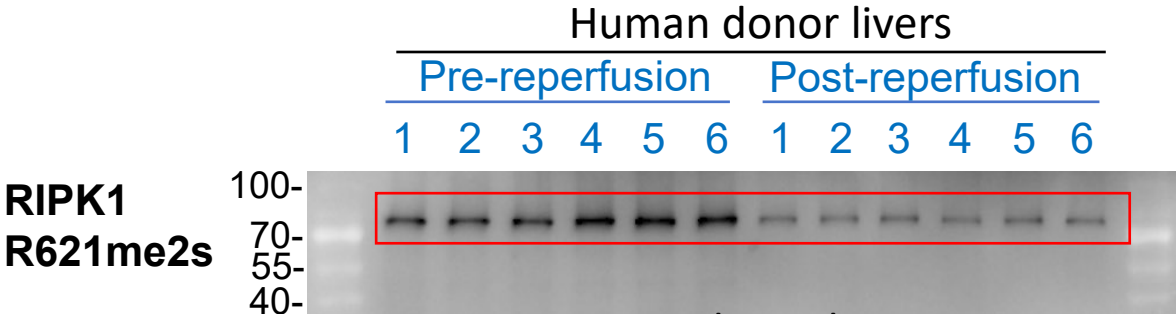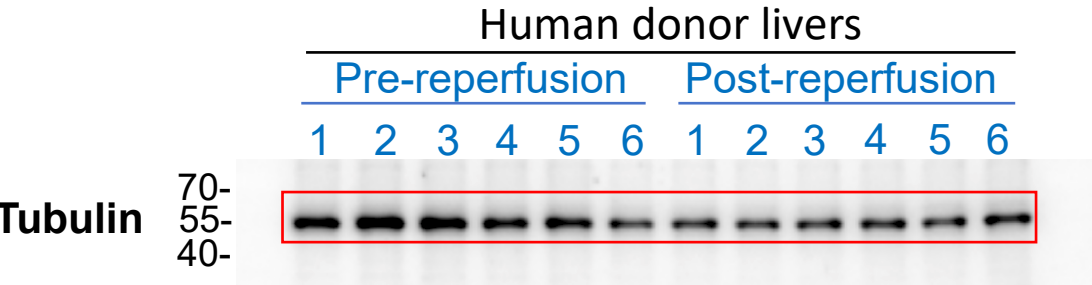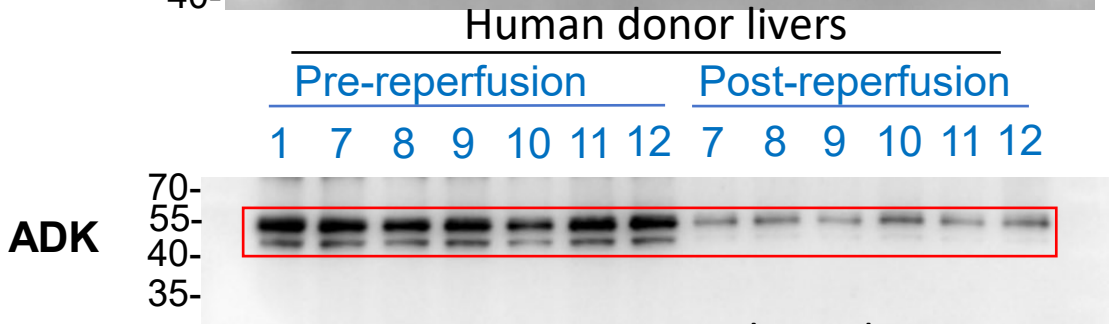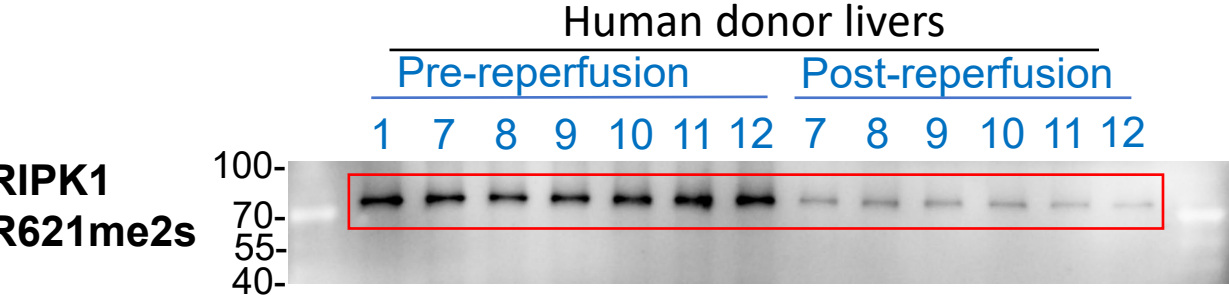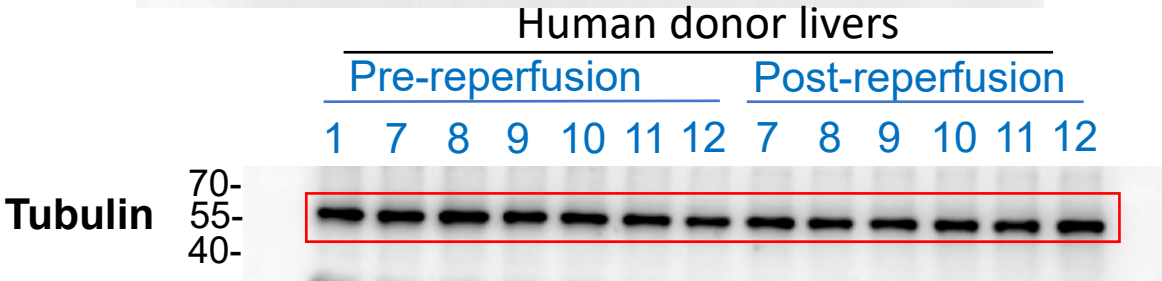

Panel E

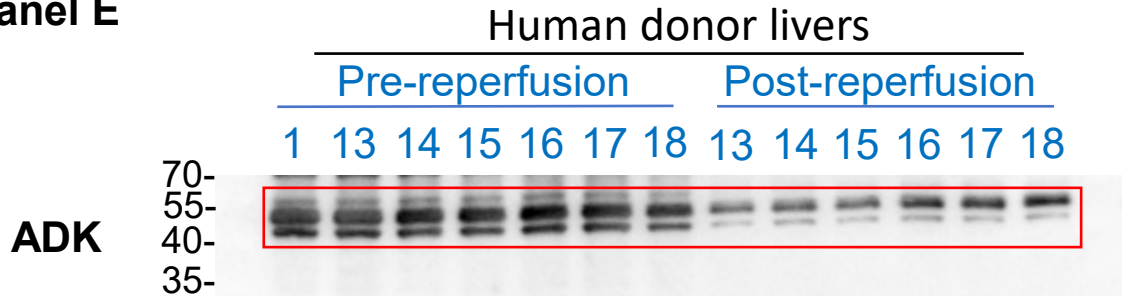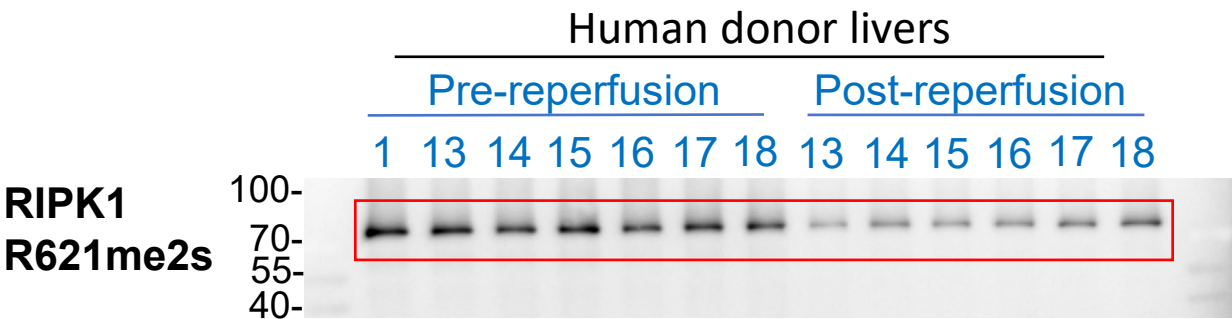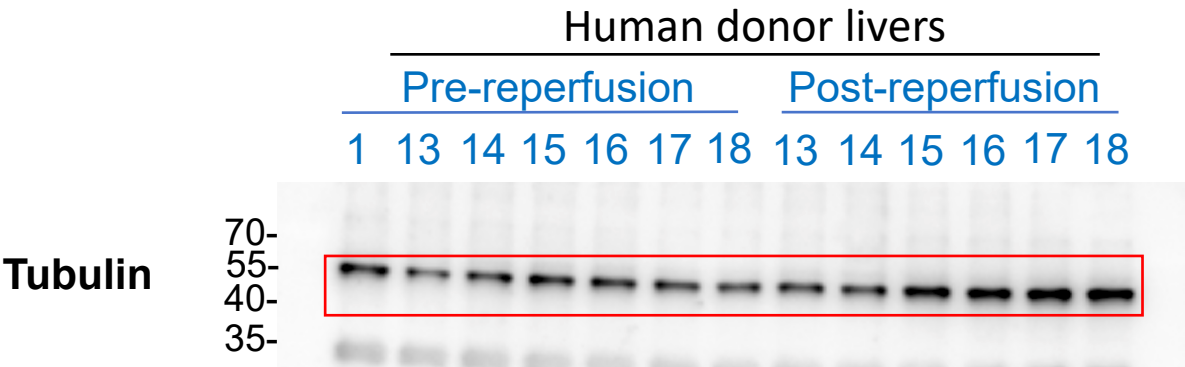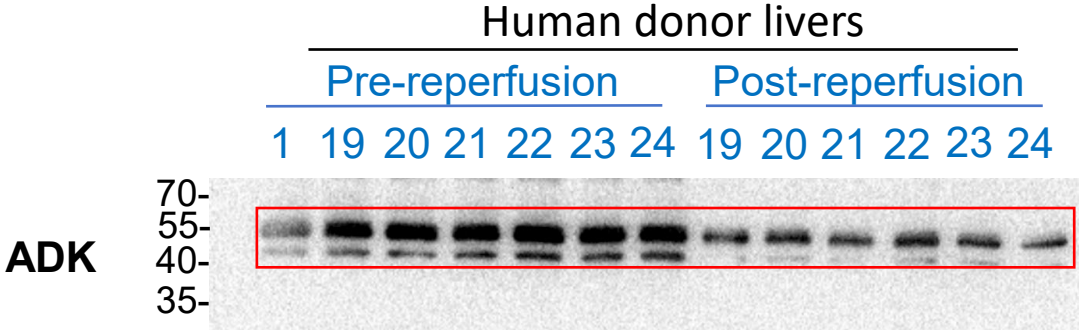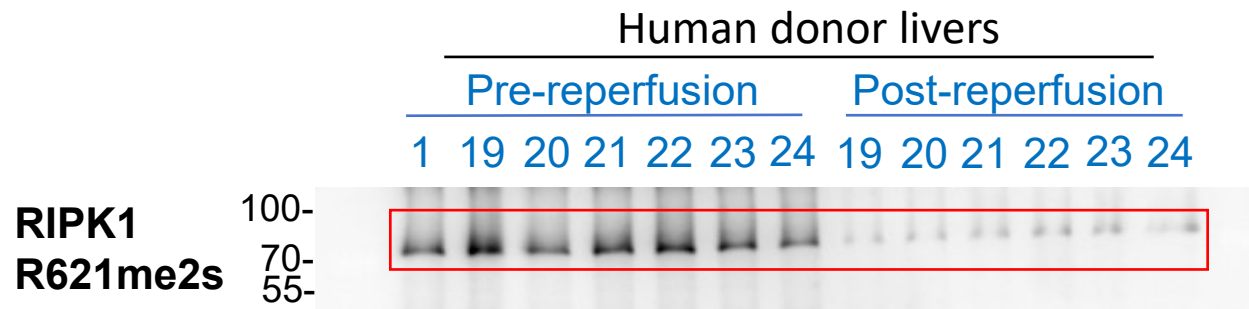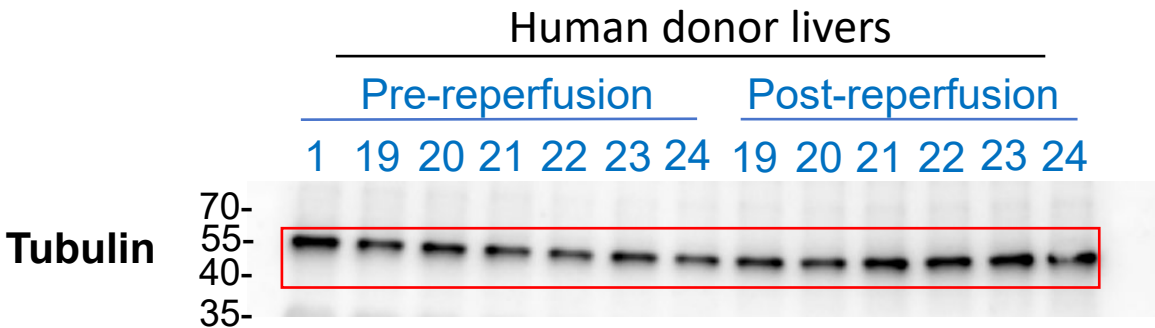

Panel G

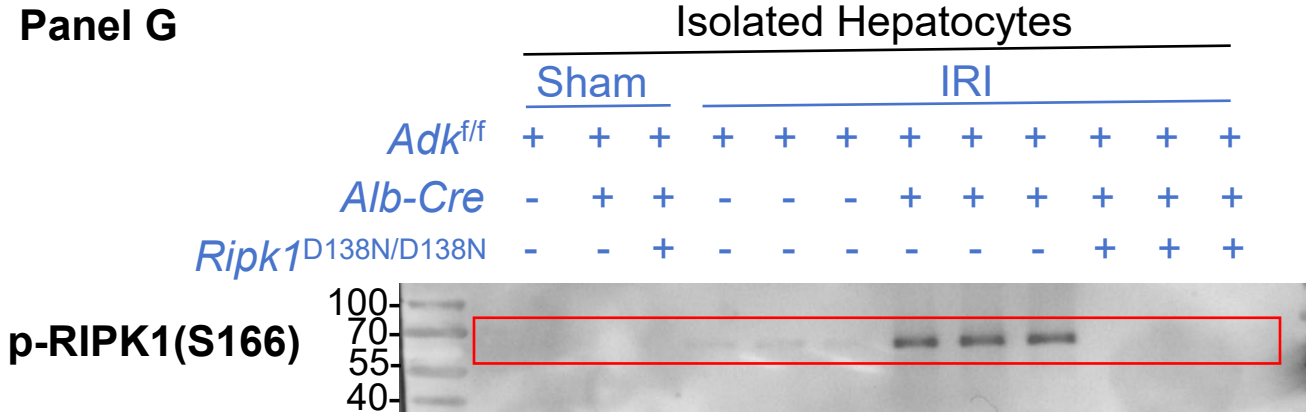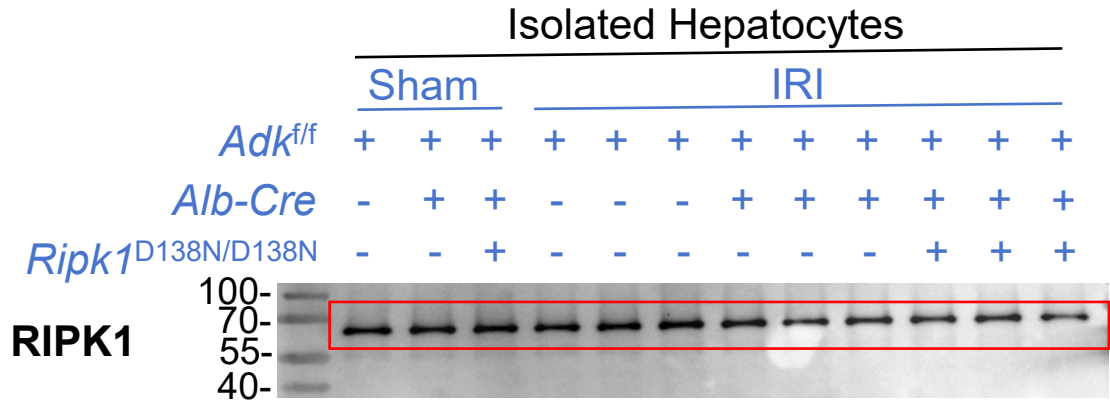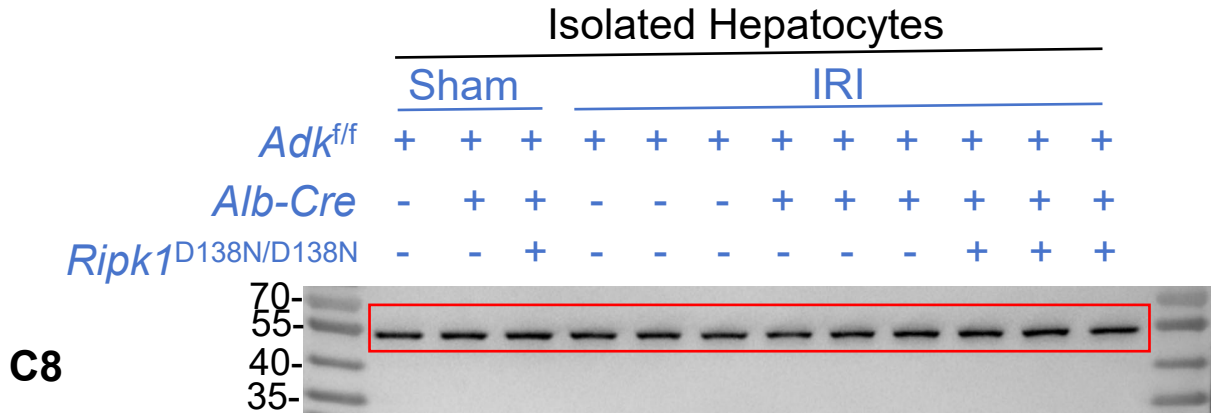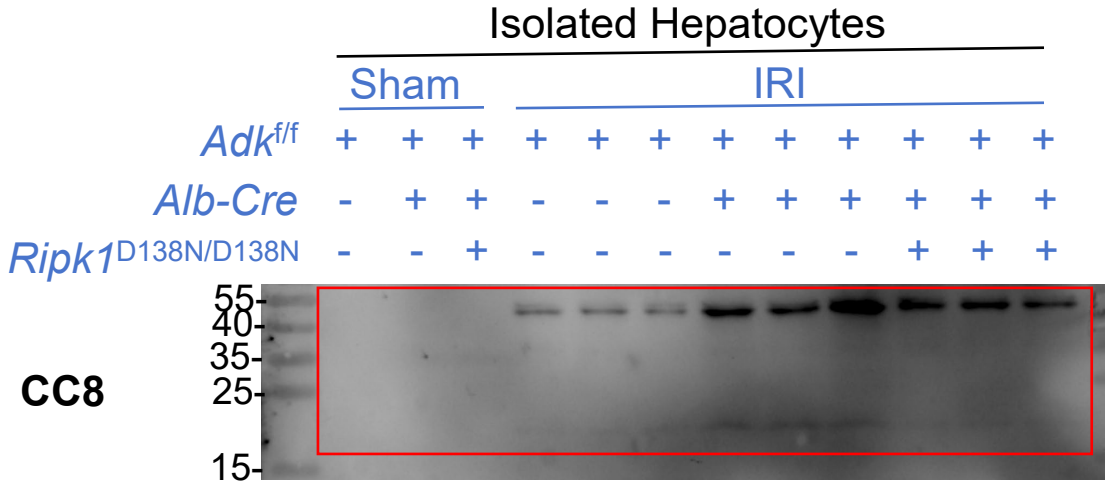

Panel G

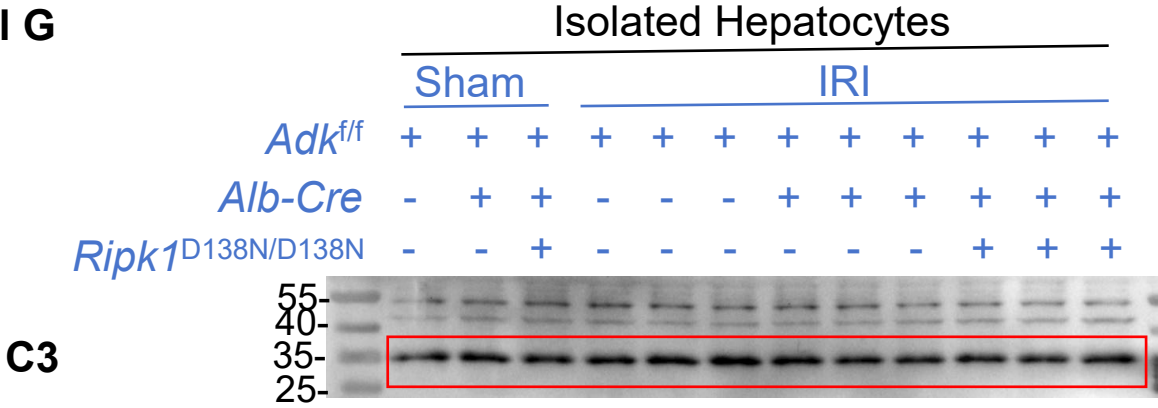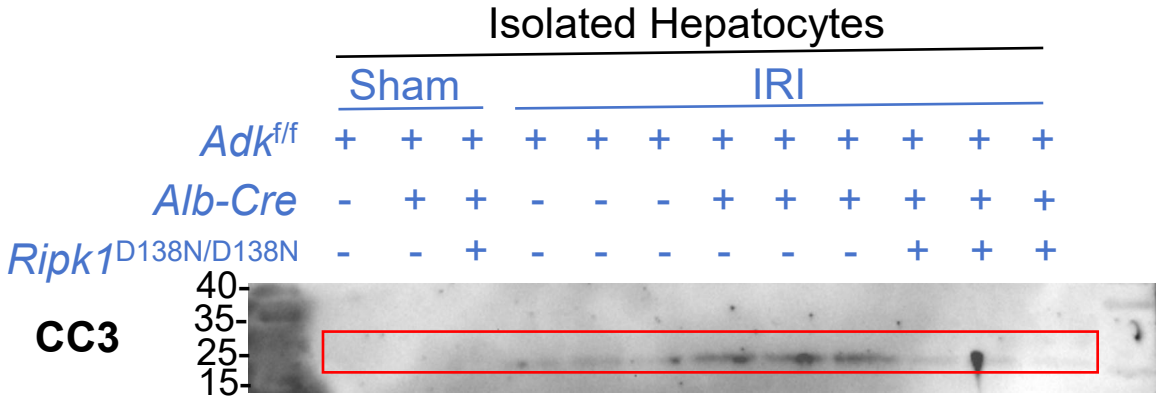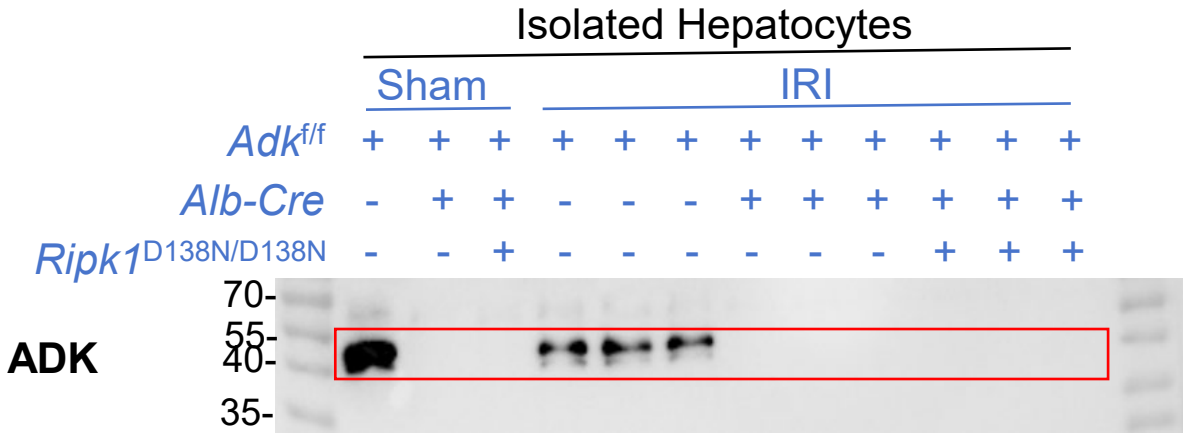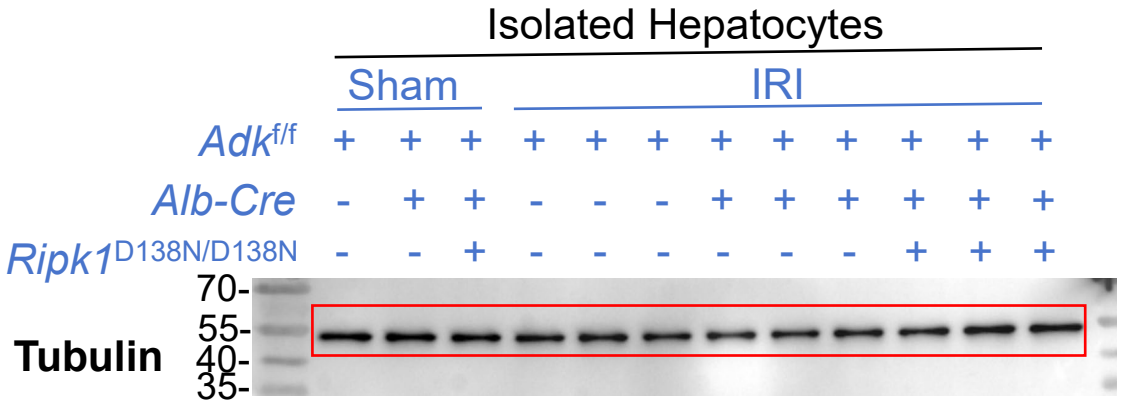

Panel J

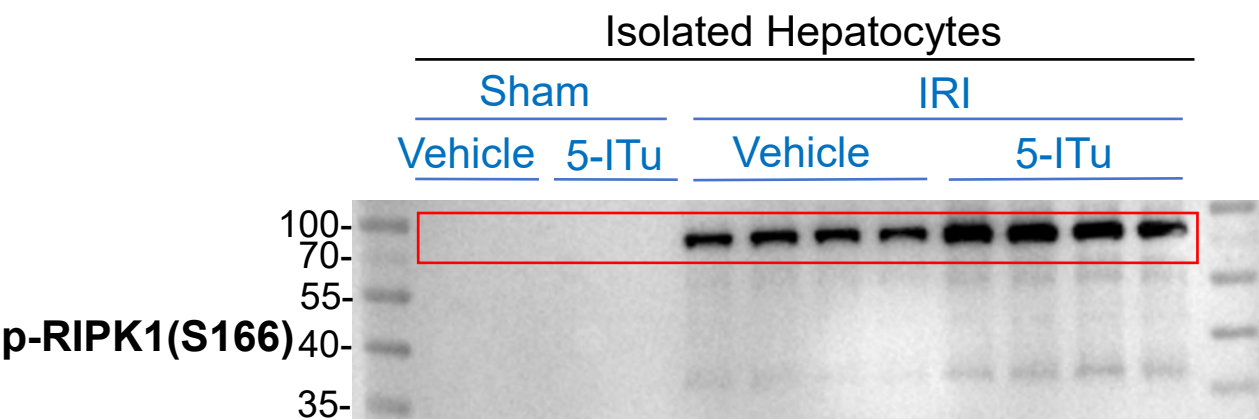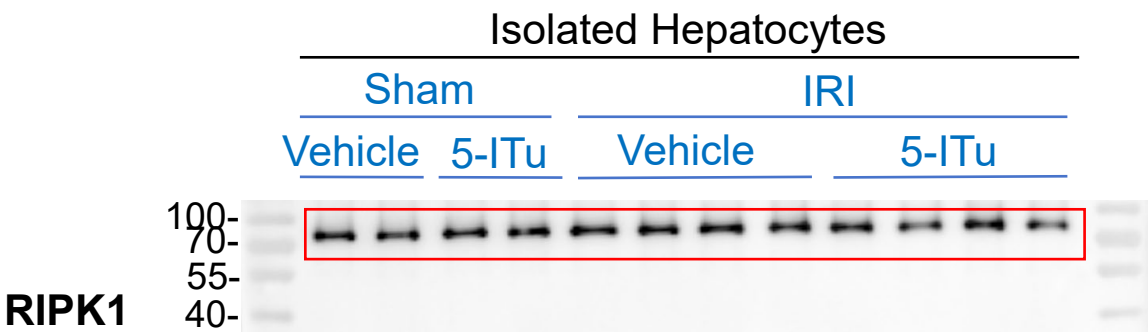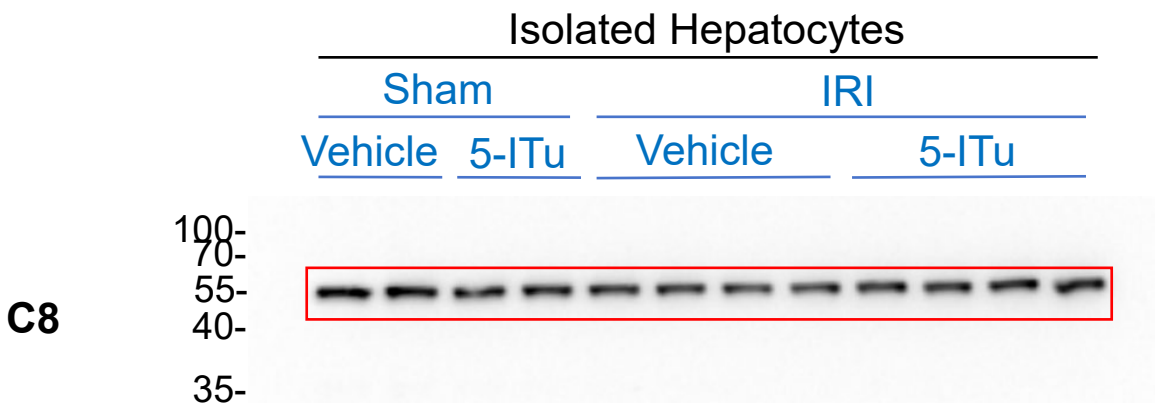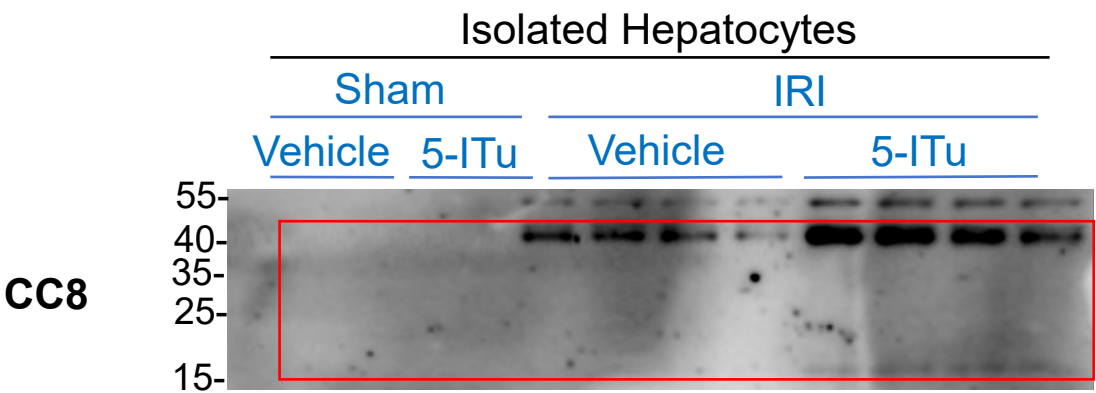

Panel J

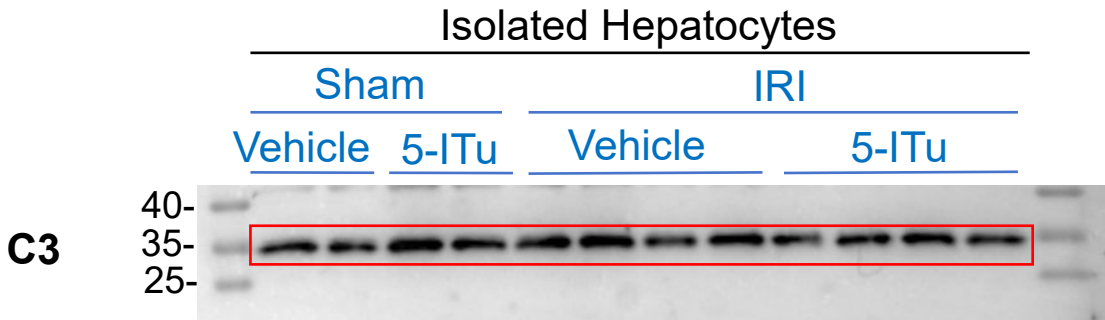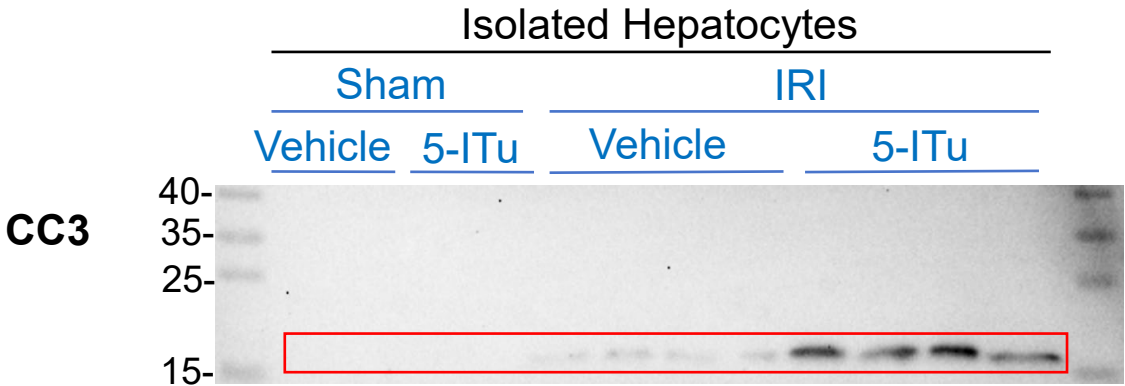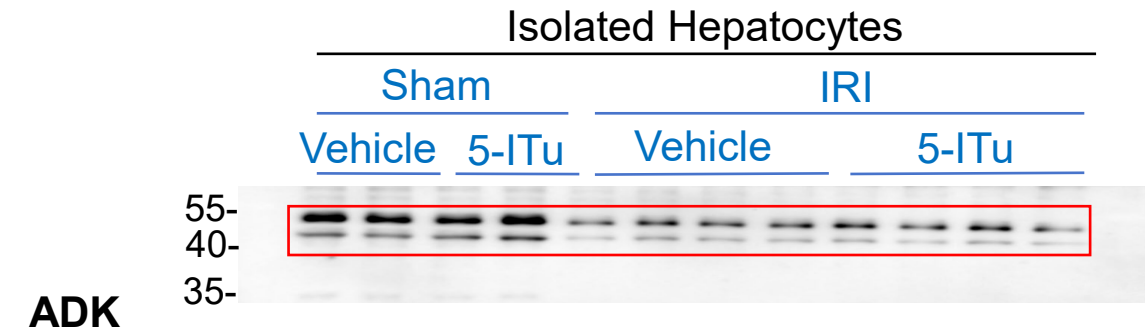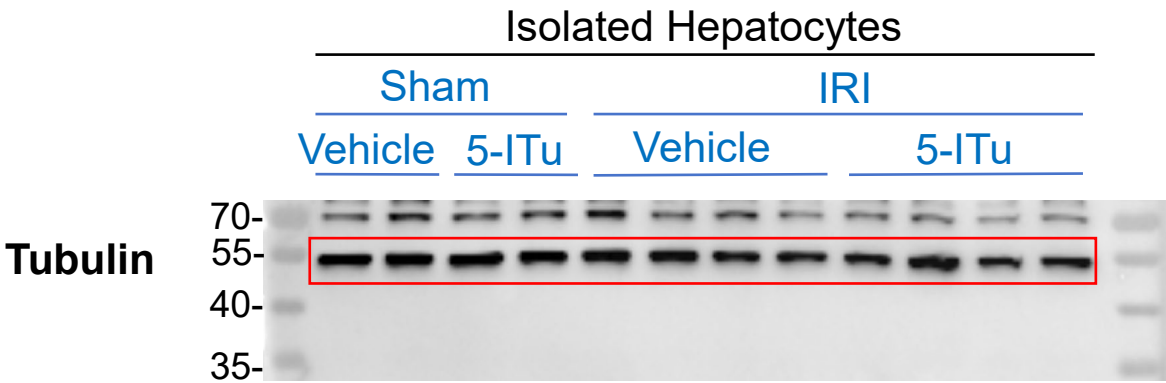

Panel M

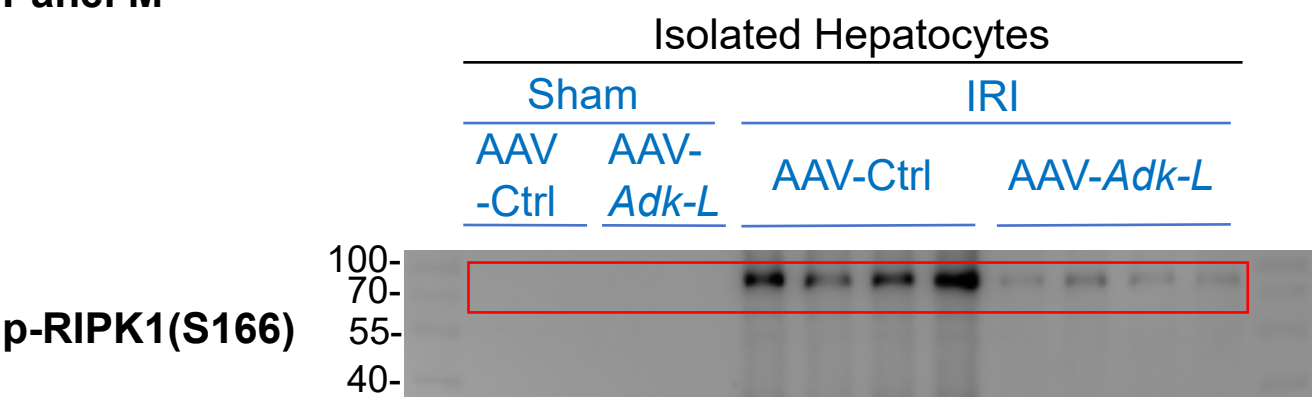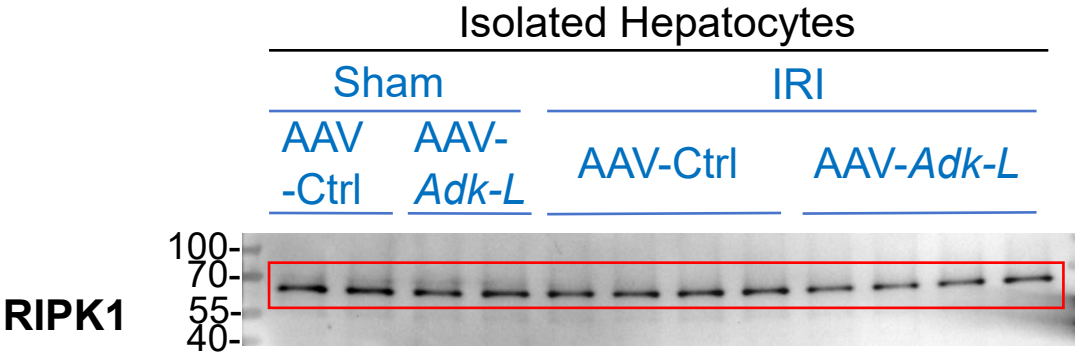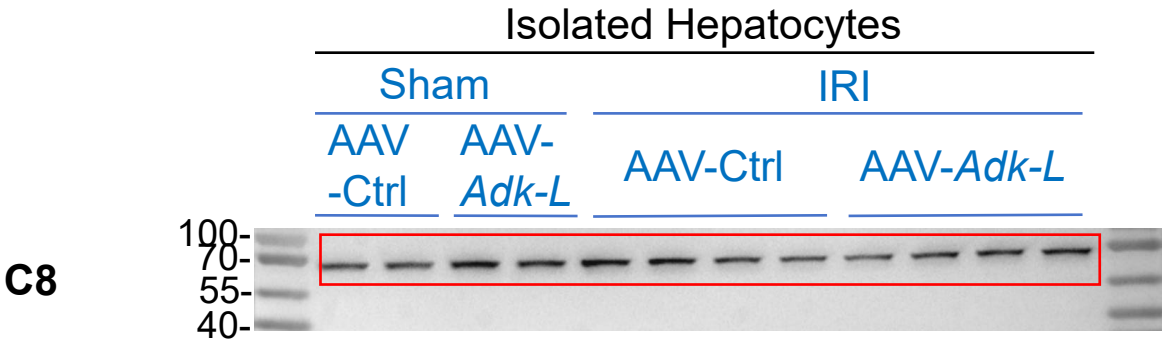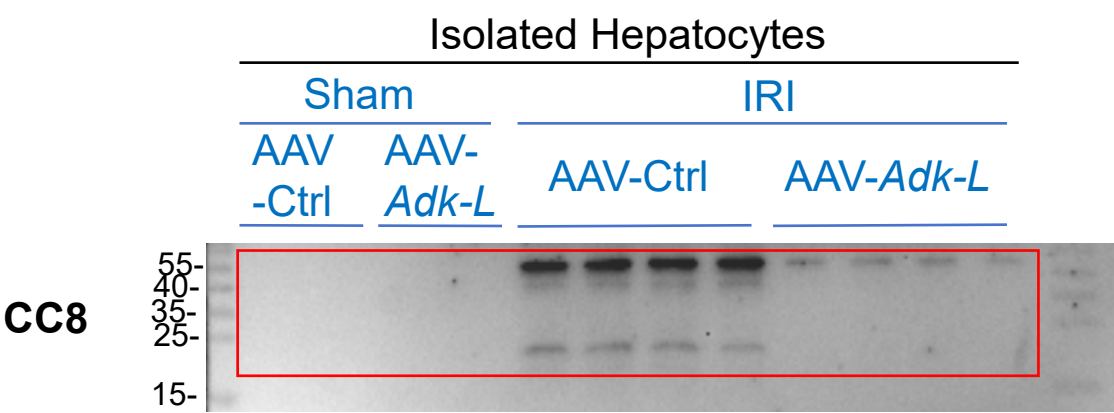

Panel M

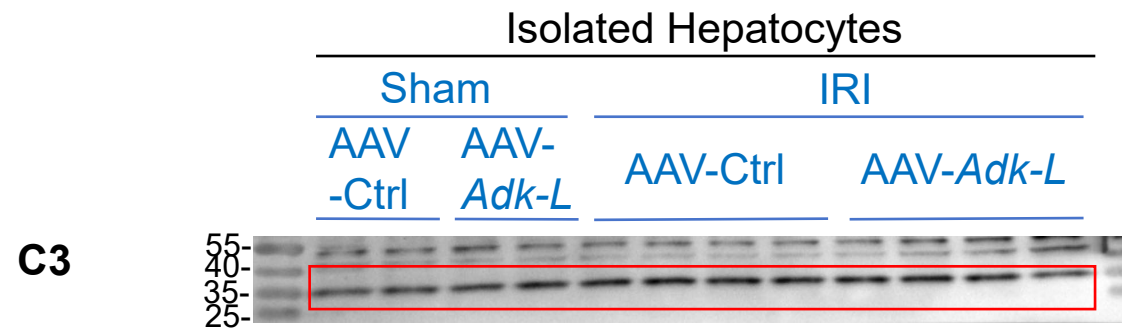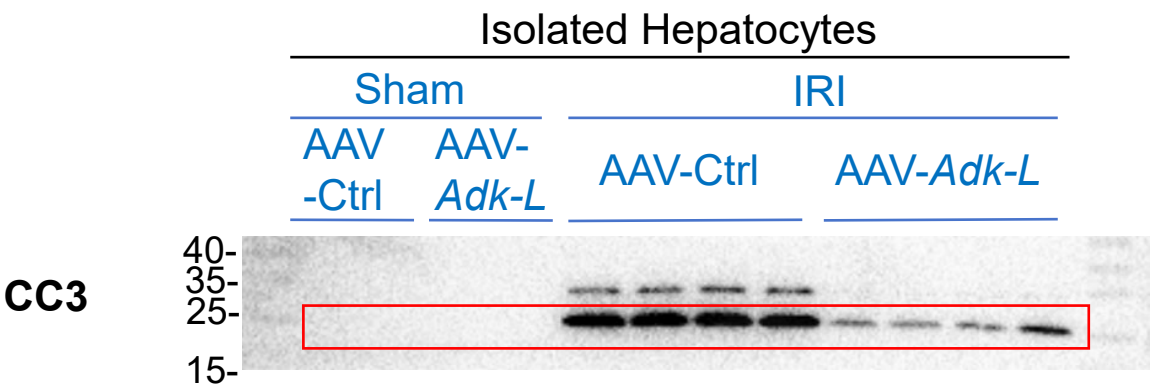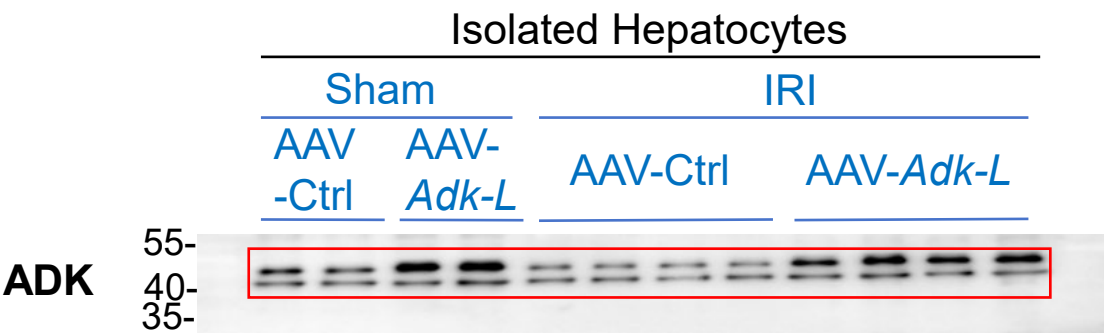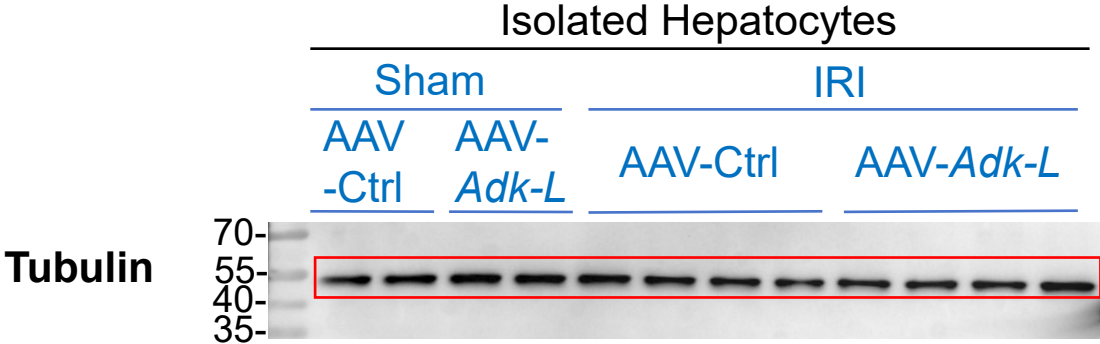

Supplement: SourceData F8 — is the source file for Fig. 8. [file jem_20250603_sourcedataf8.pdf]

Panel E

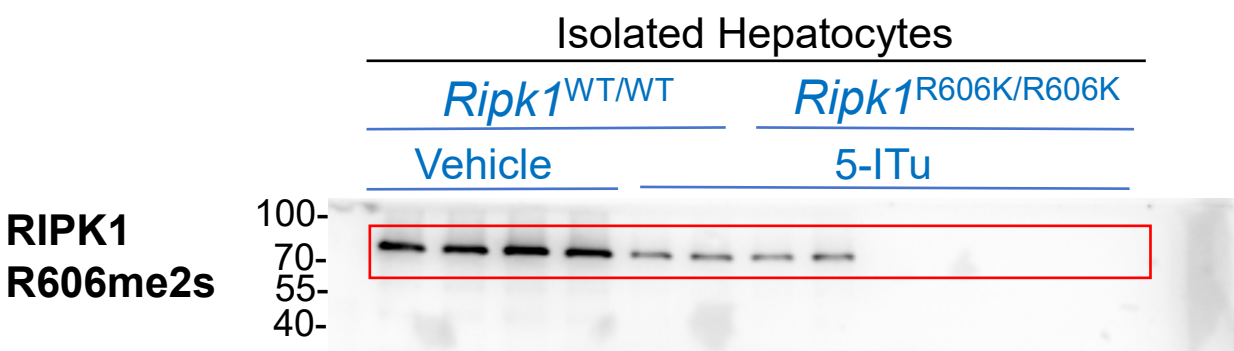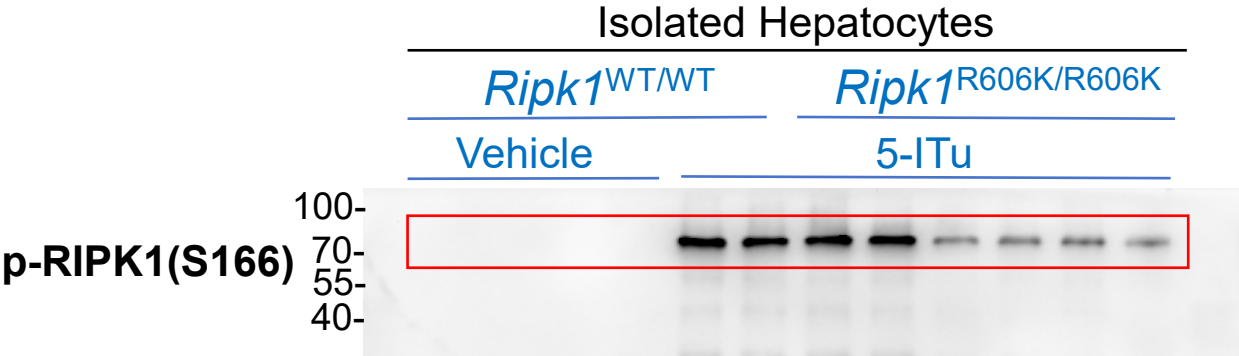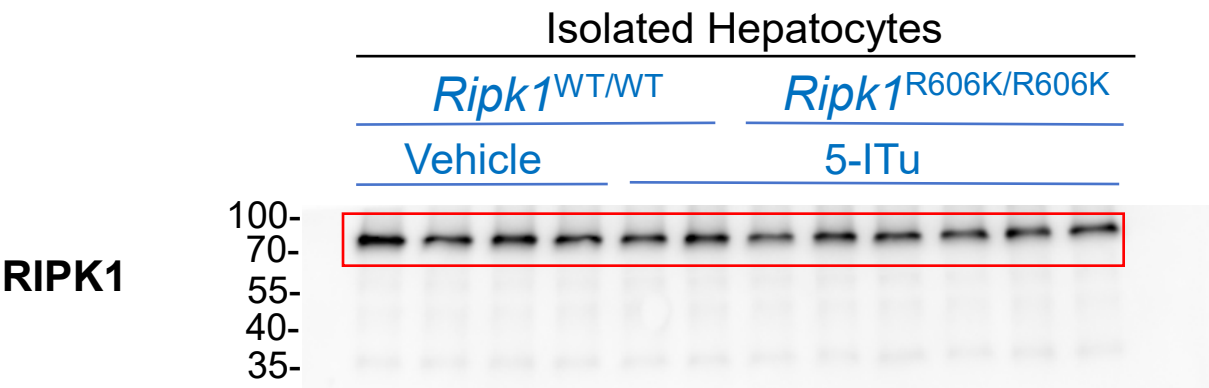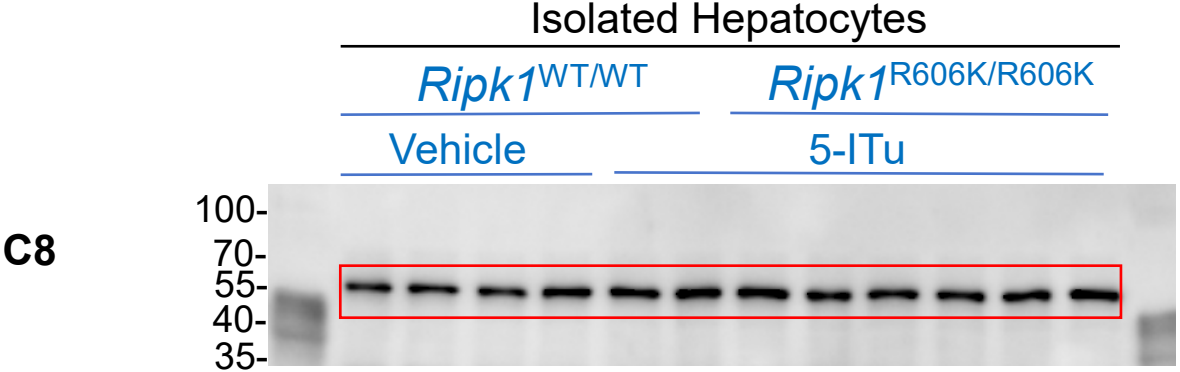

Panel E

CC8

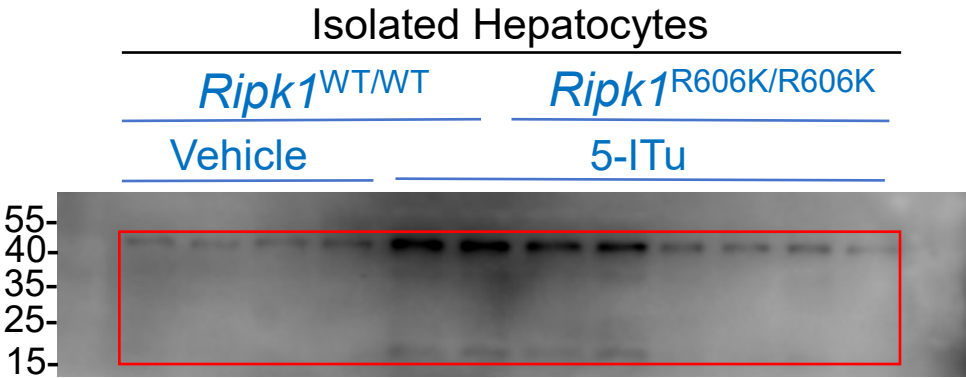

C3

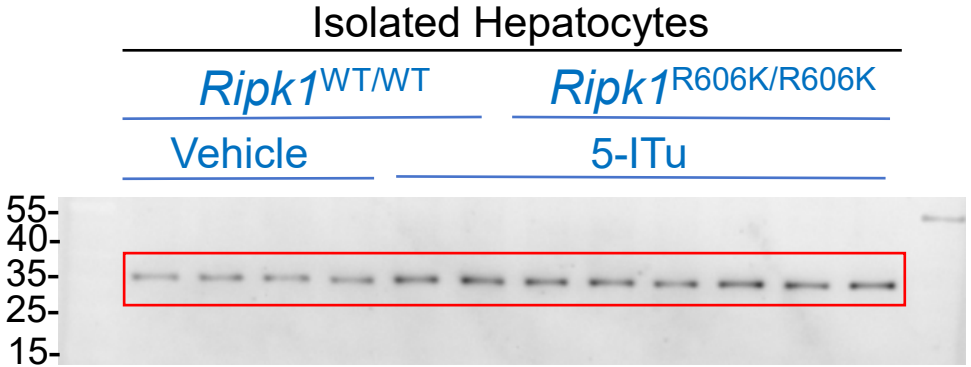

CC3

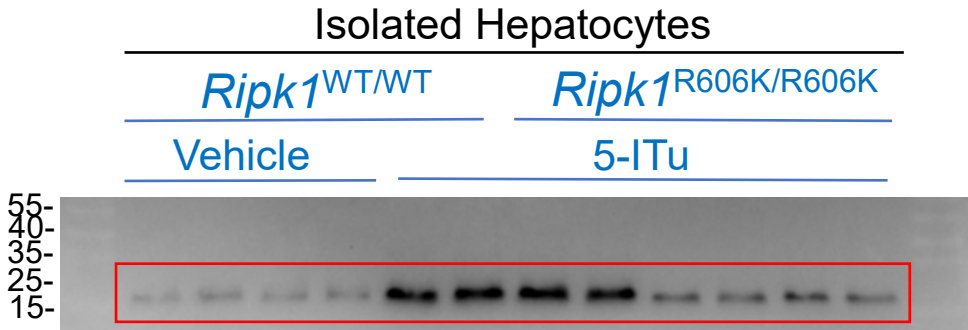

Tubulin

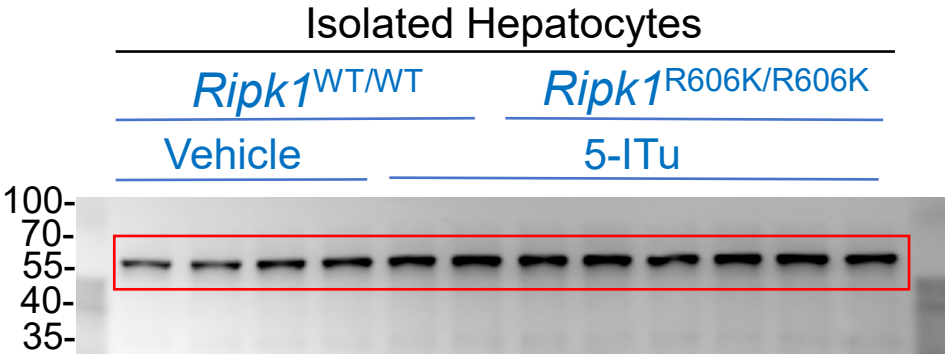

Supplement: SourceData F9 — is the source file for Fig. 9. [file jem_20250603_sourcedataf9.pdf]

Panel A

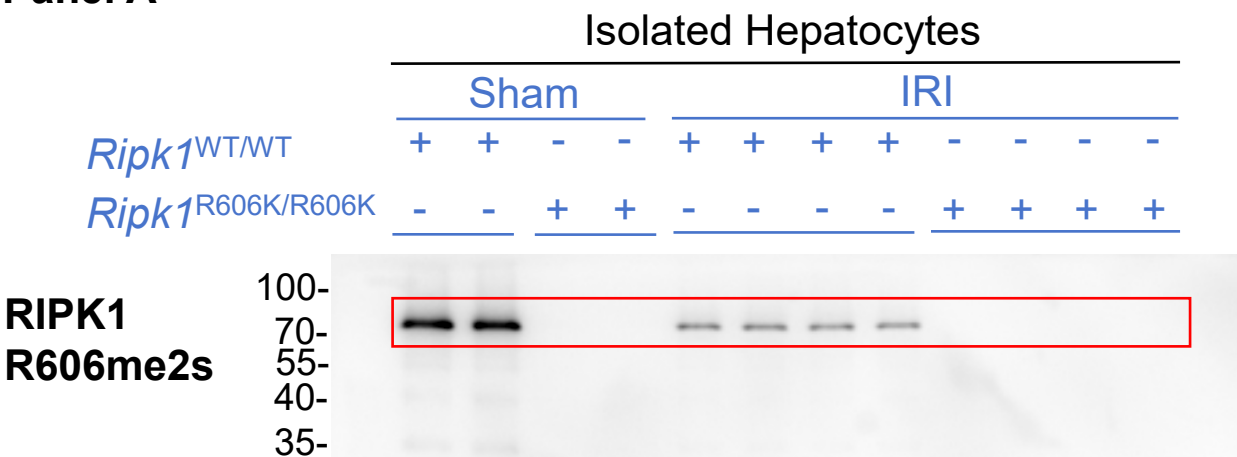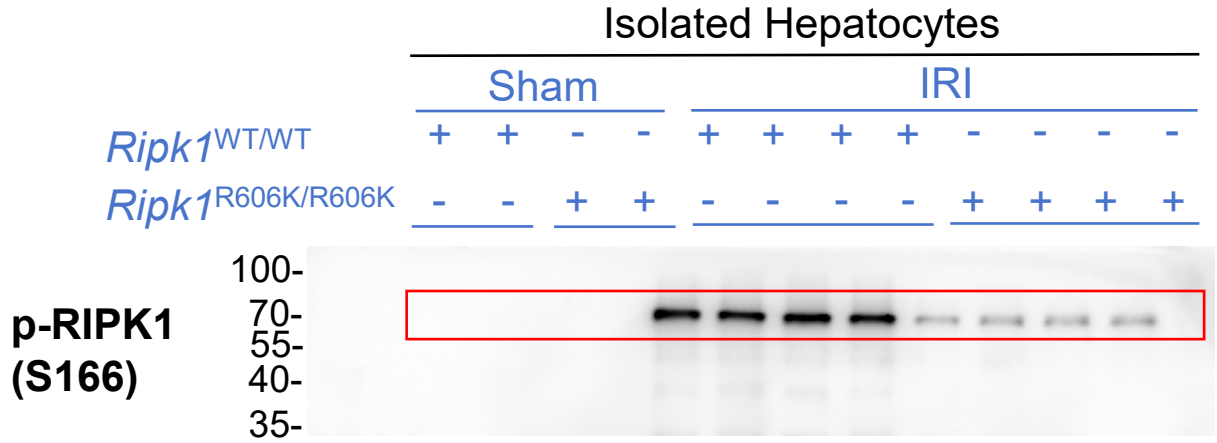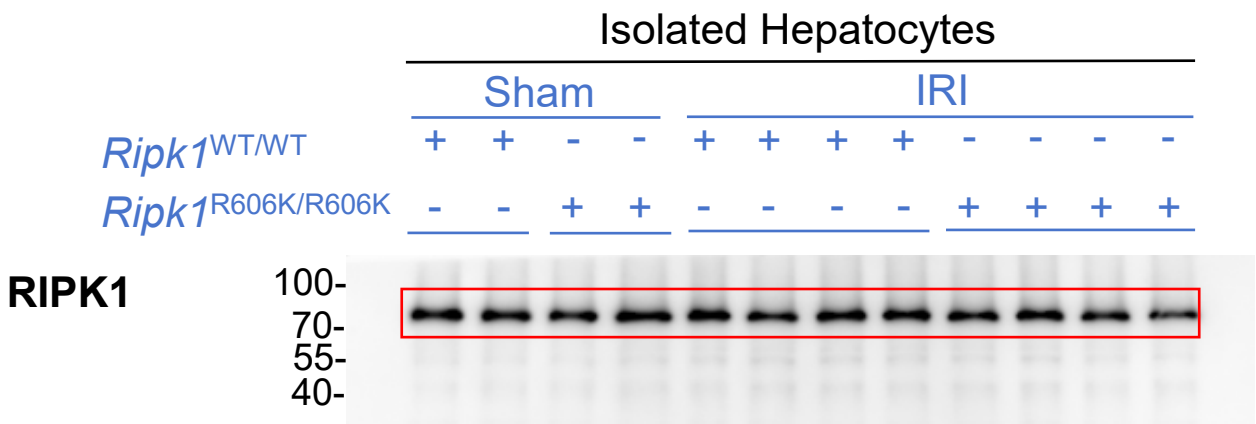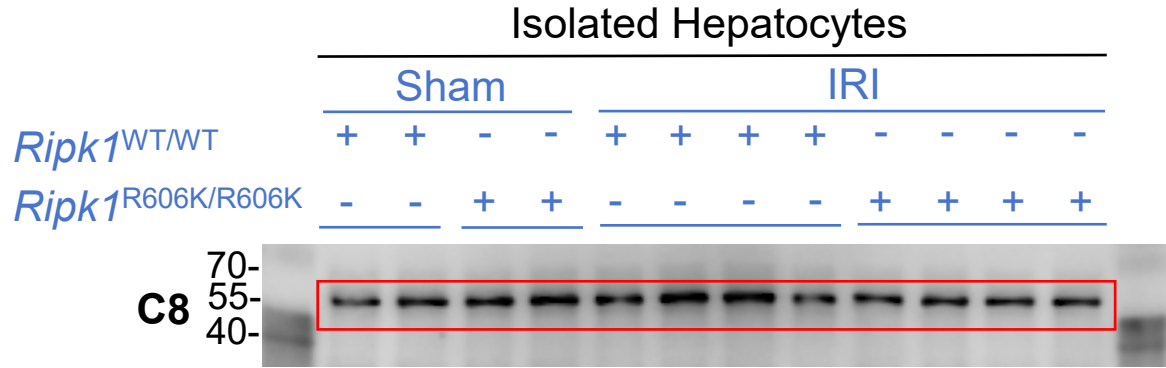

Panel A

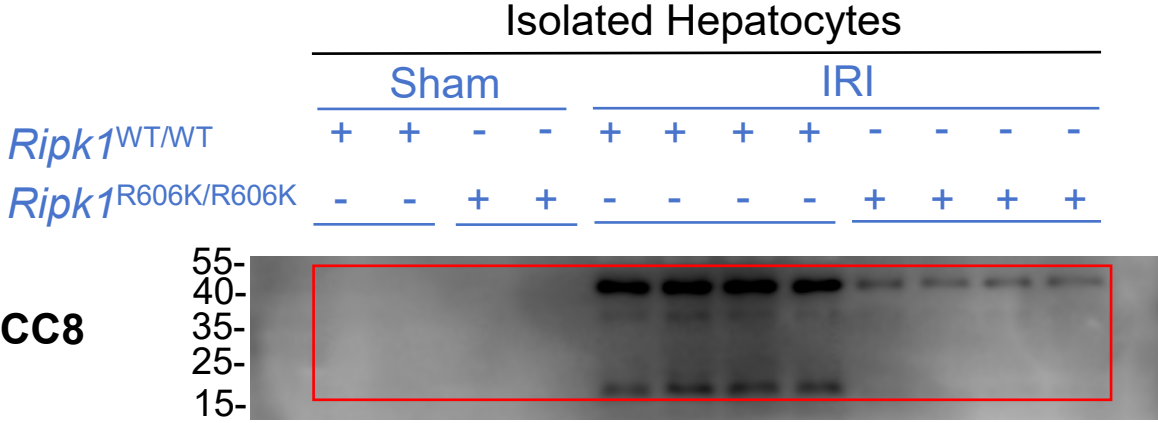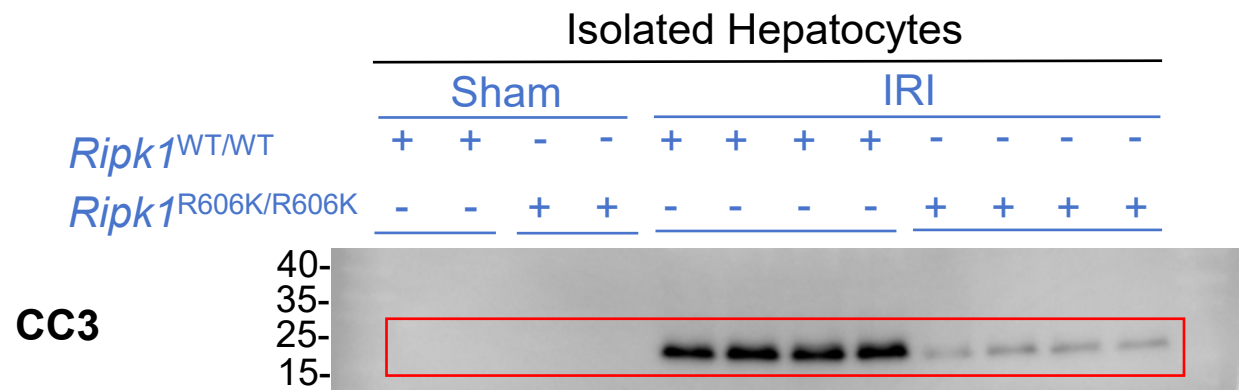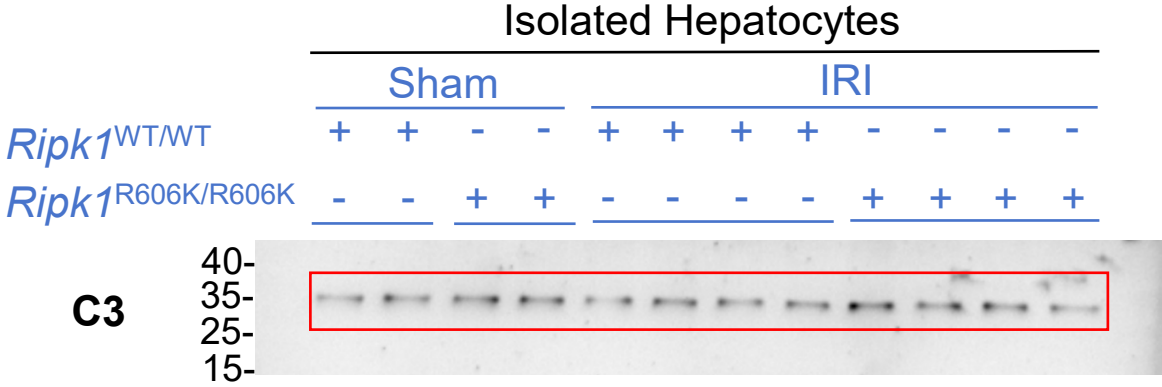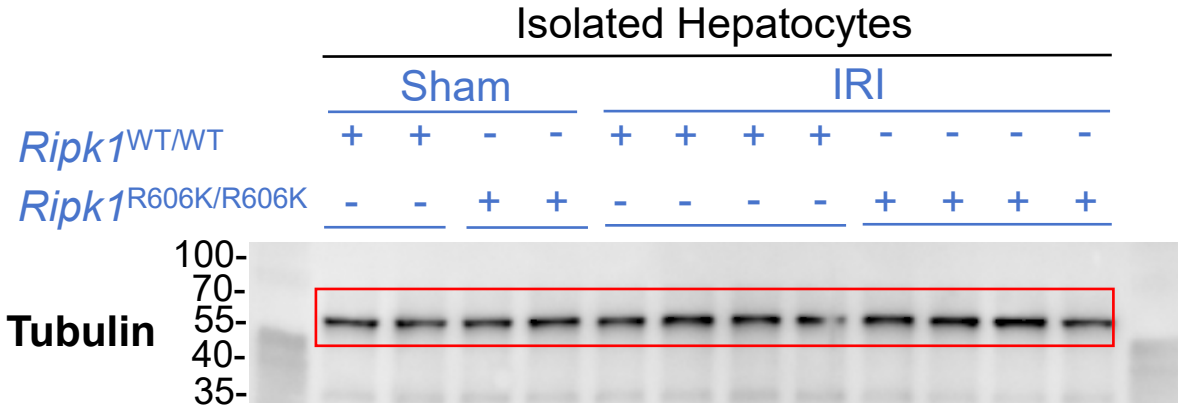

Panel J

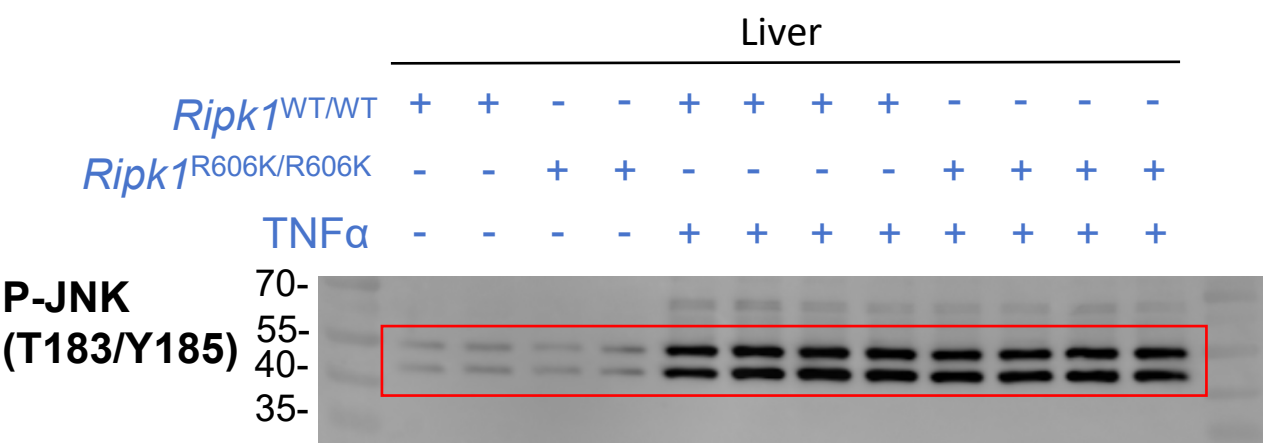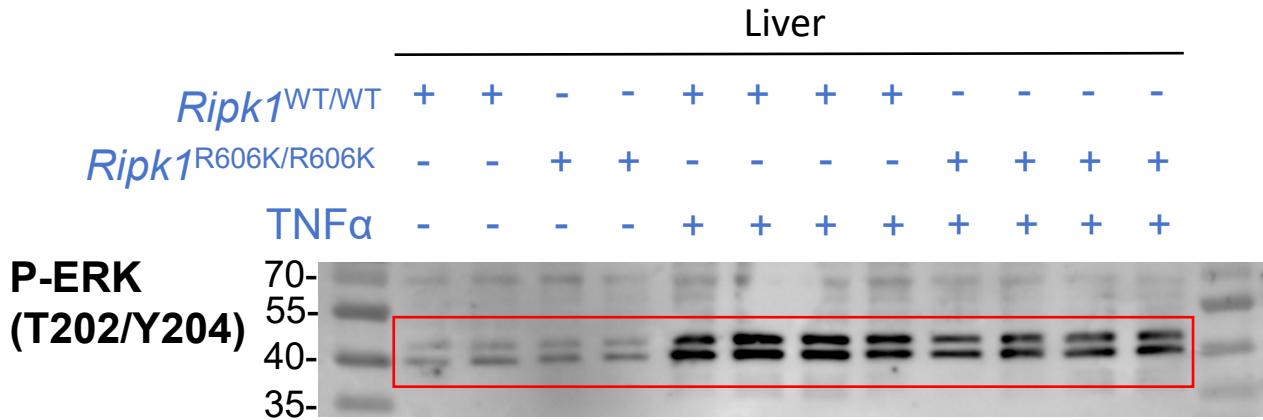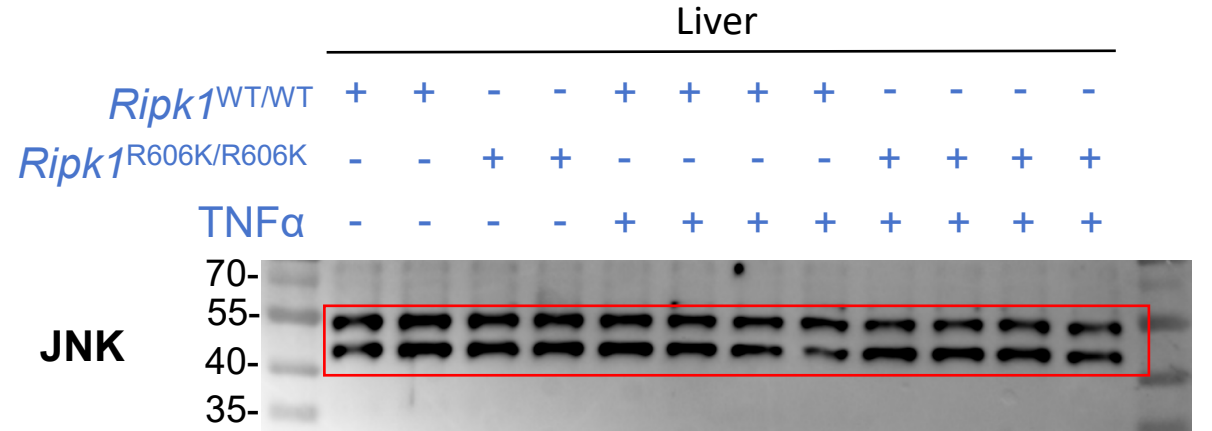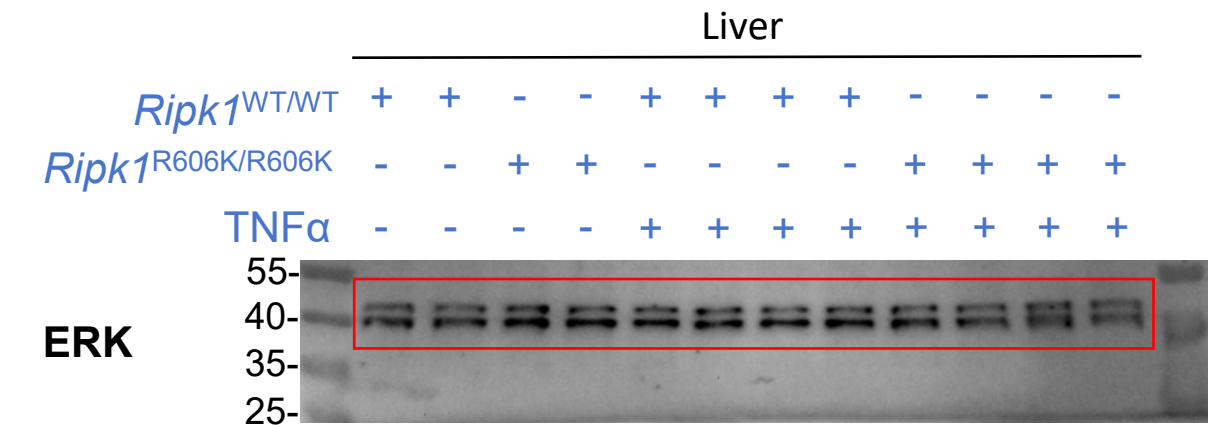

Panel J

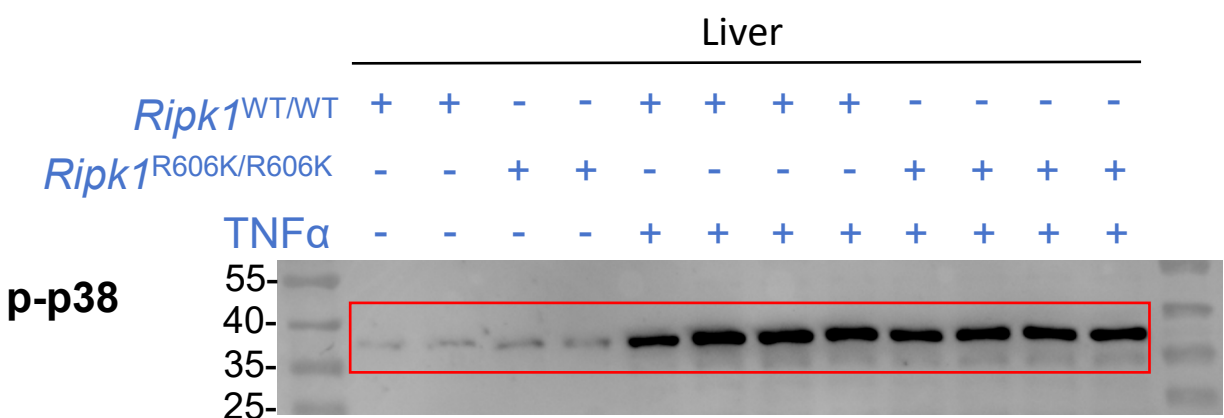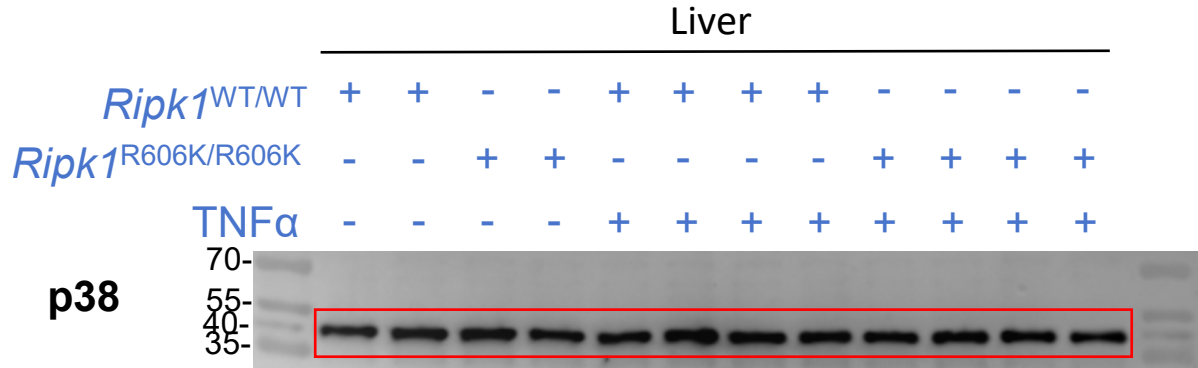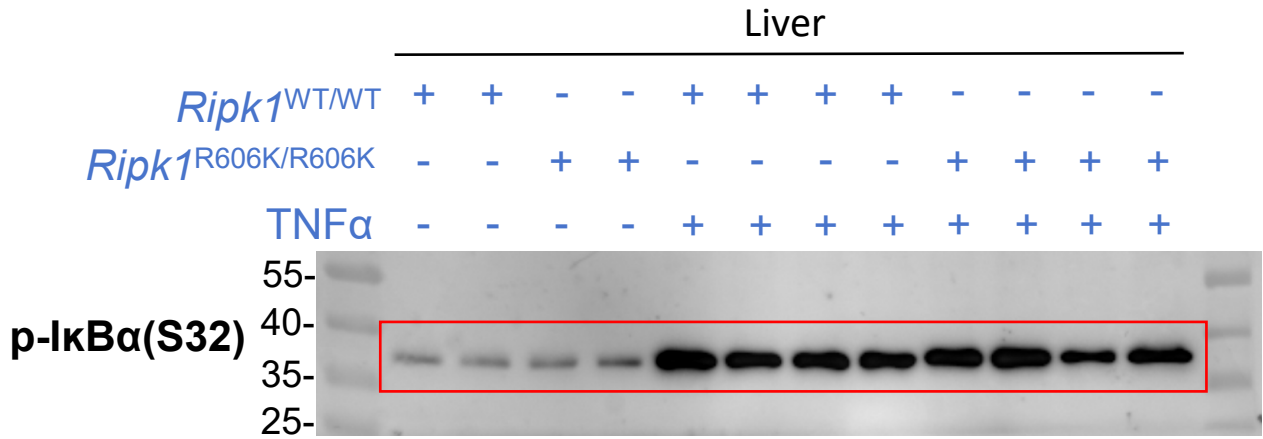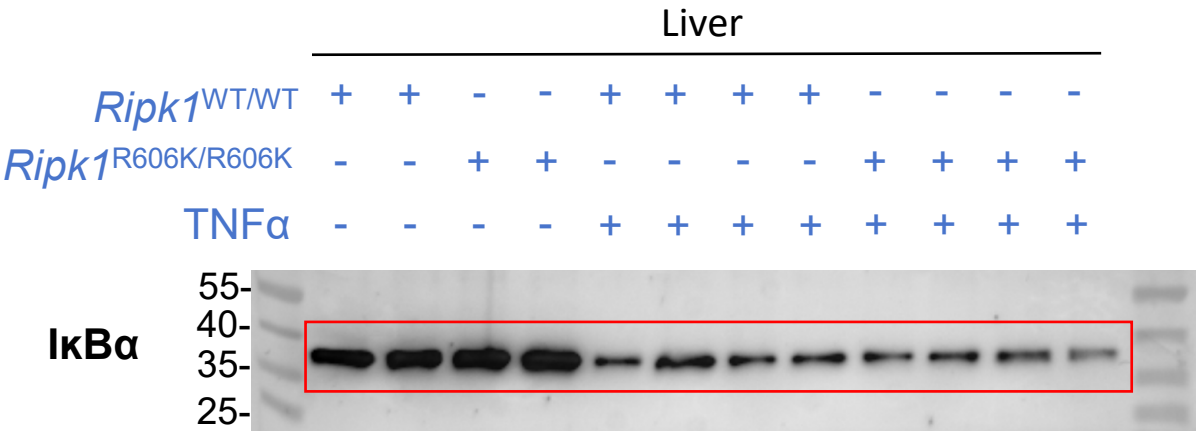

Panel J

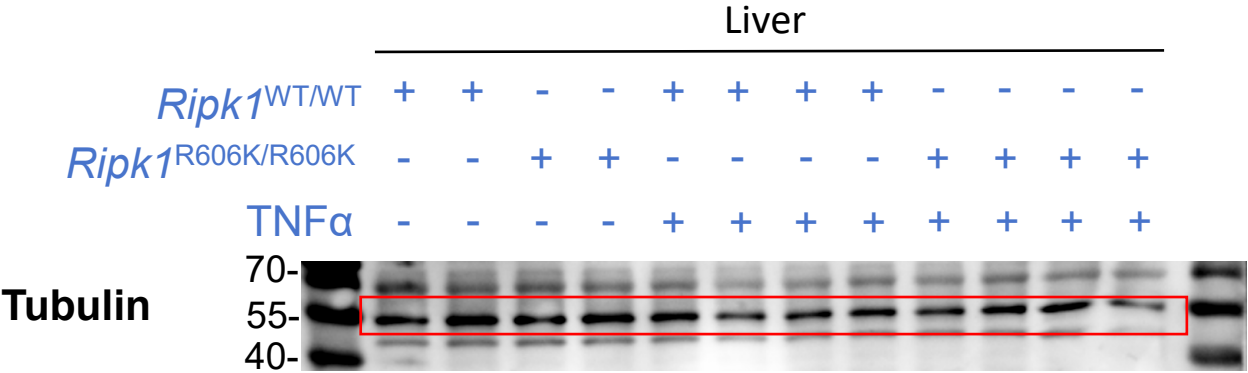

Supplement: SourceData F10 — is the source file for Fig. 10. [file jem_20250603_sourcedataf10.pdf]

Panel B

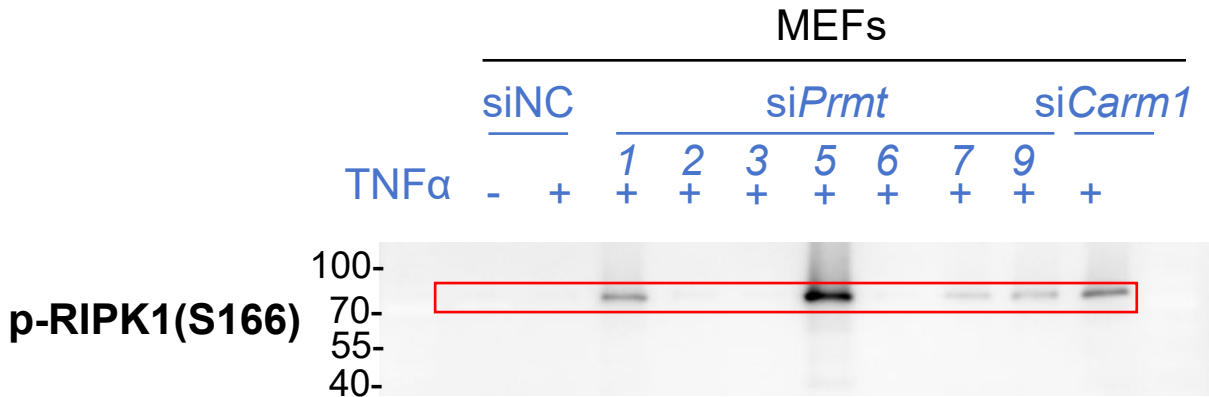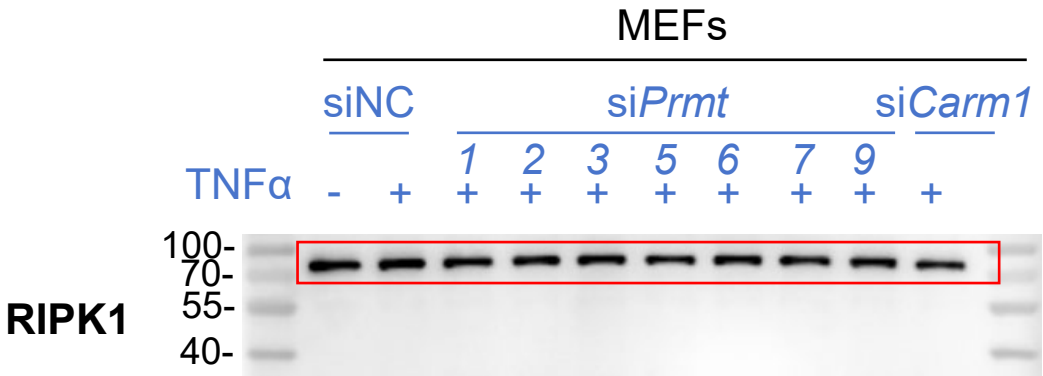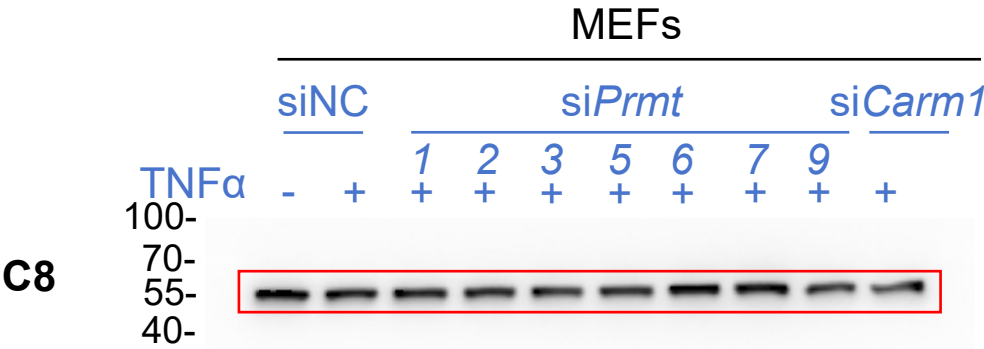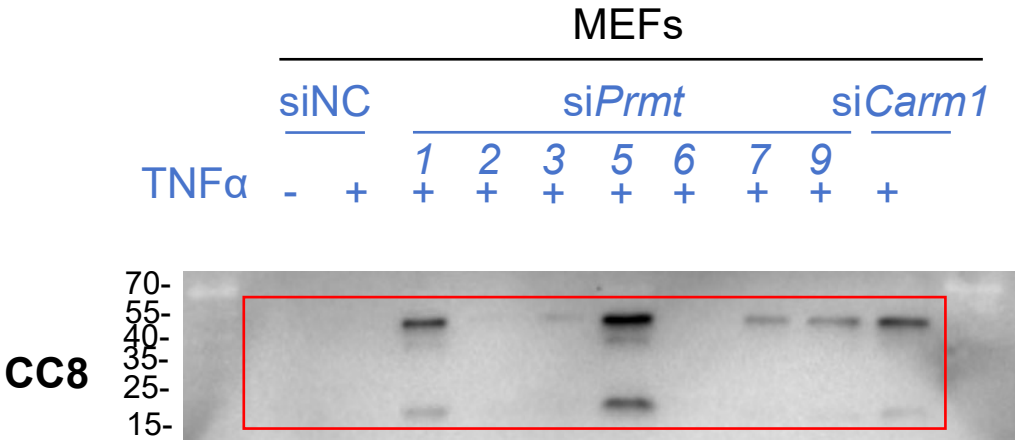

Panel B

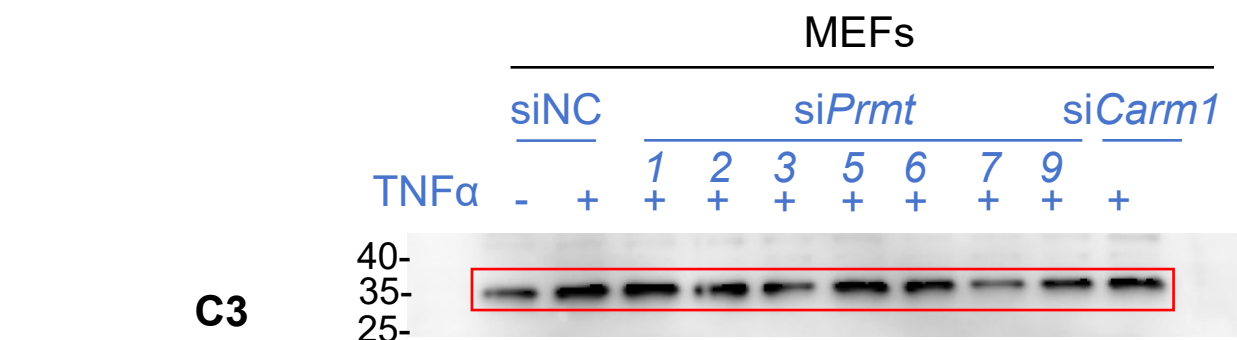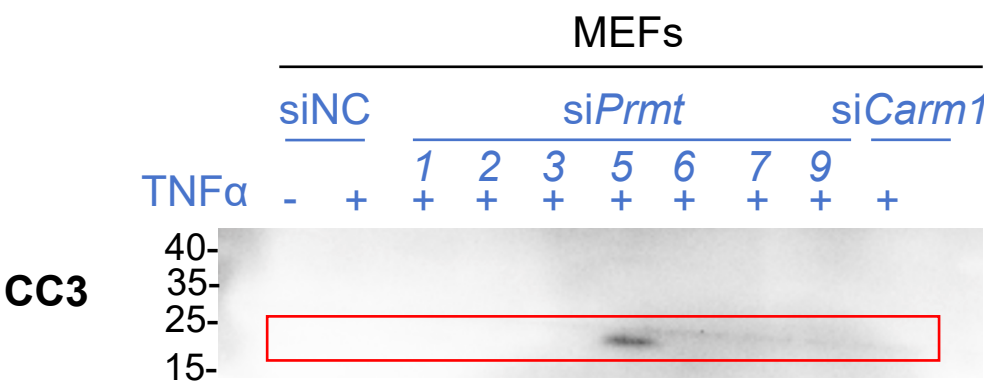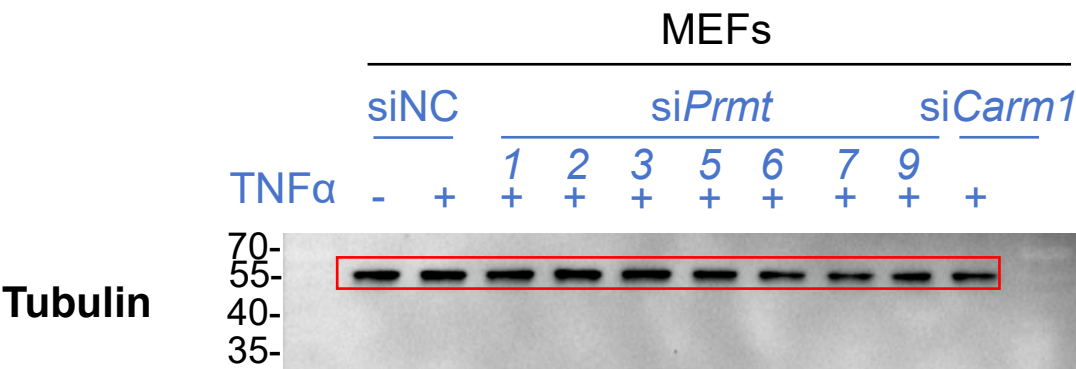

Panel D

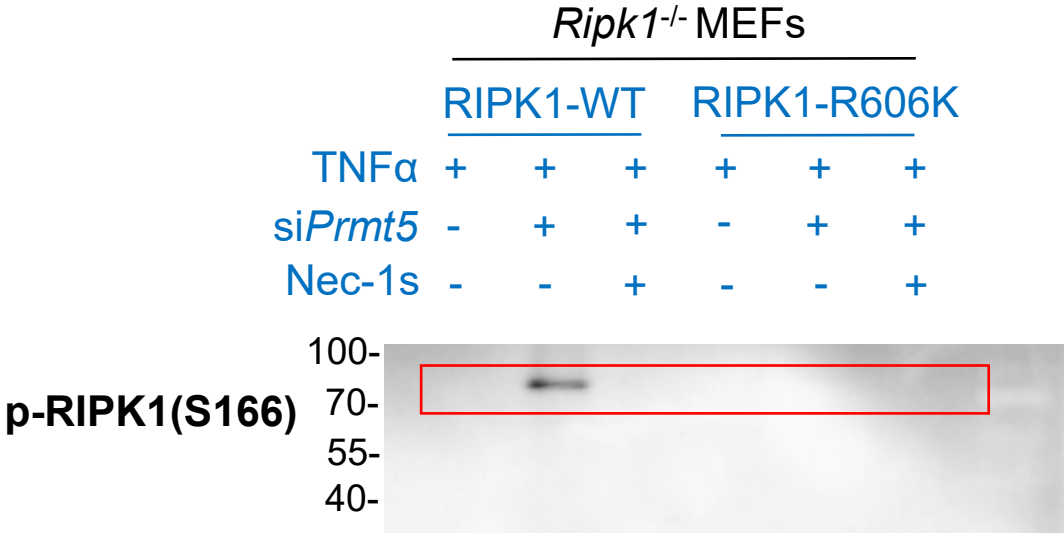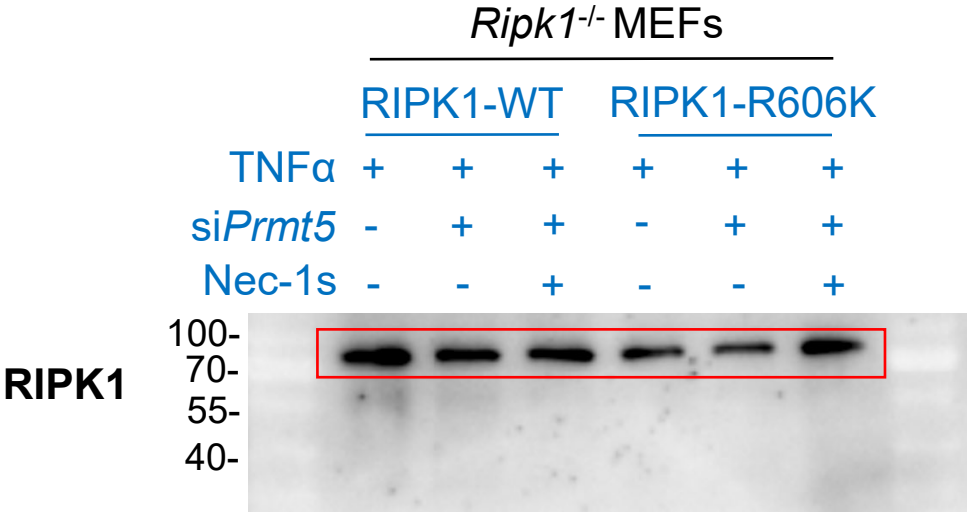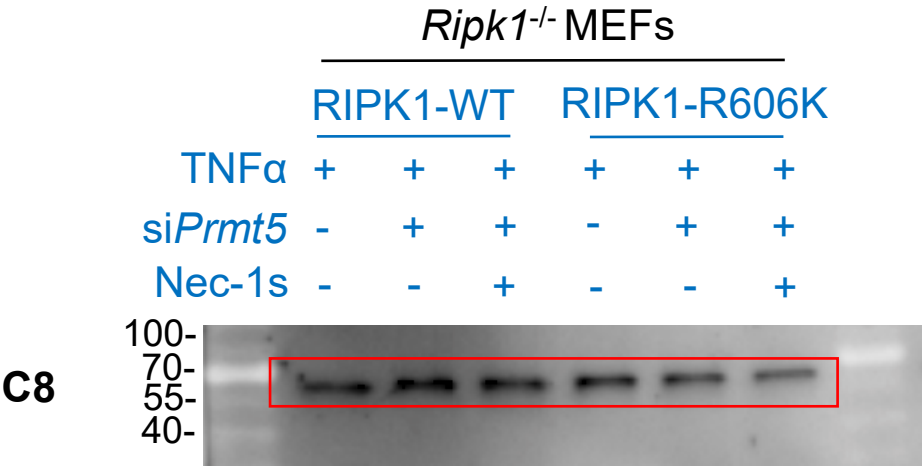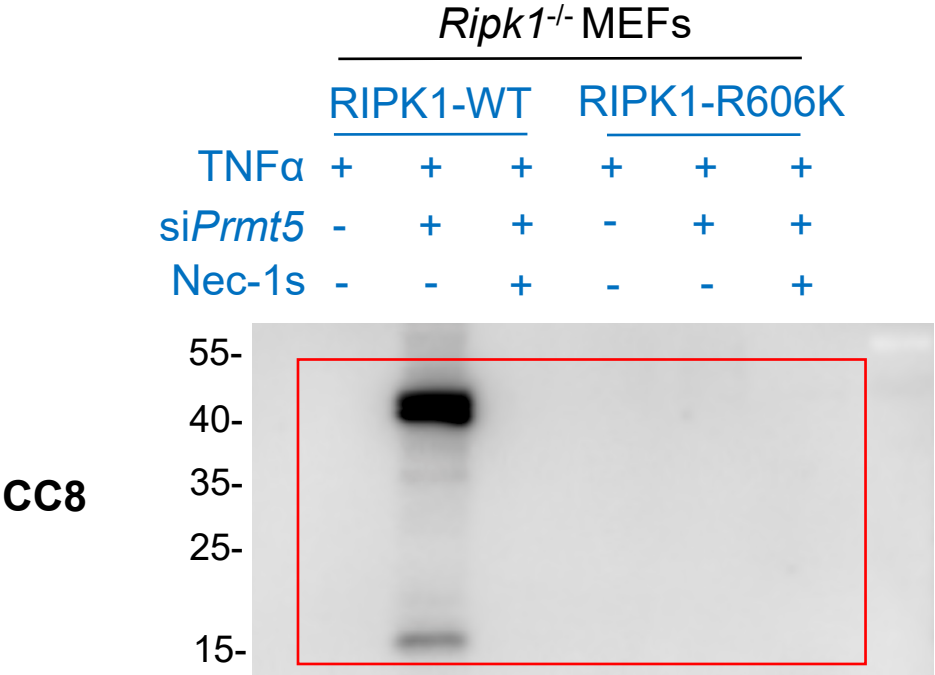

Panel D

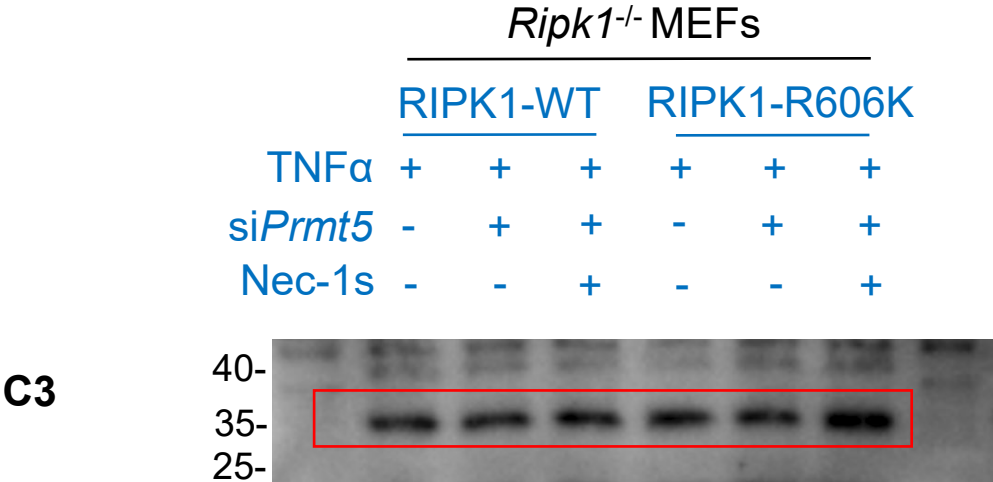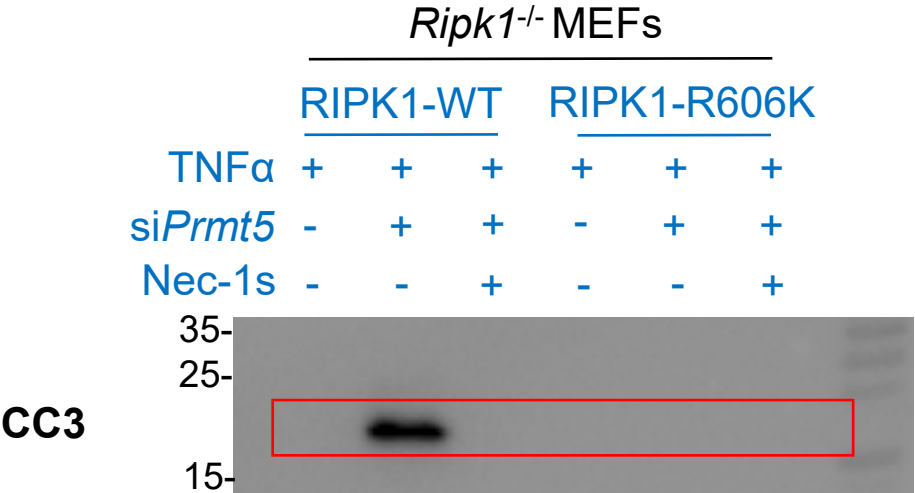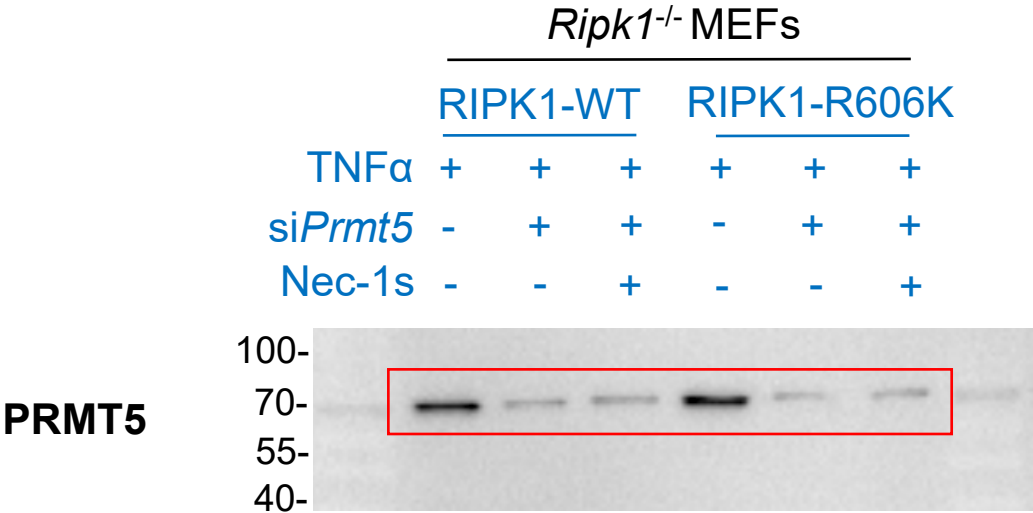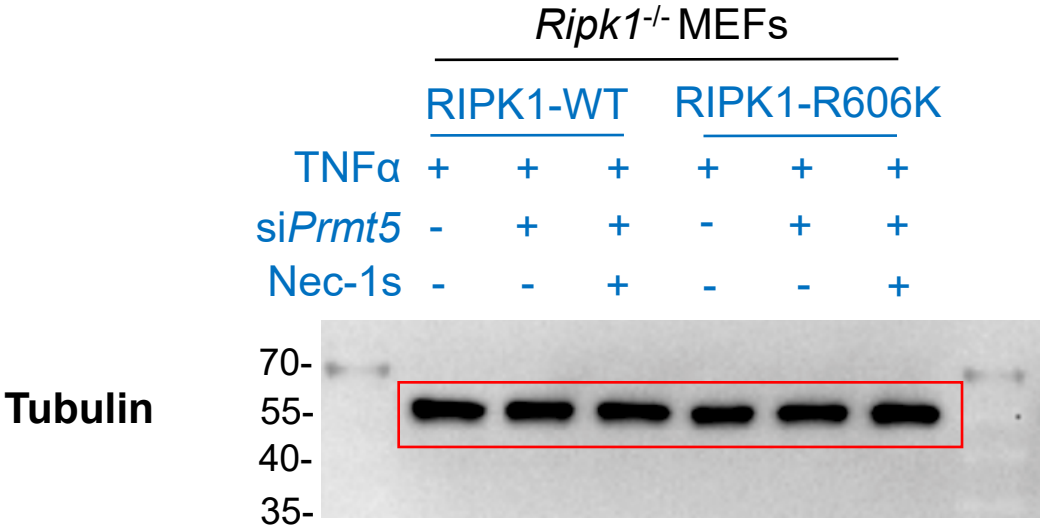

Panel F

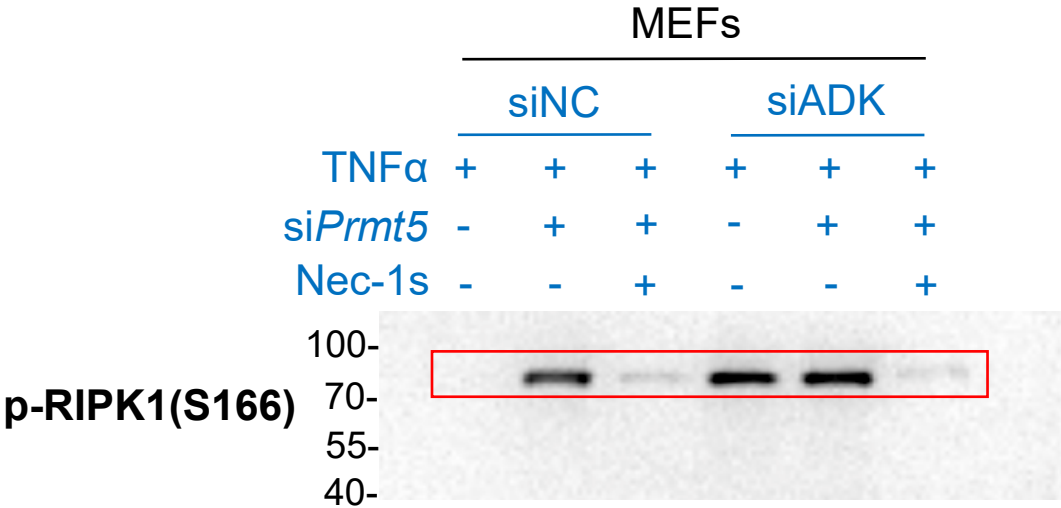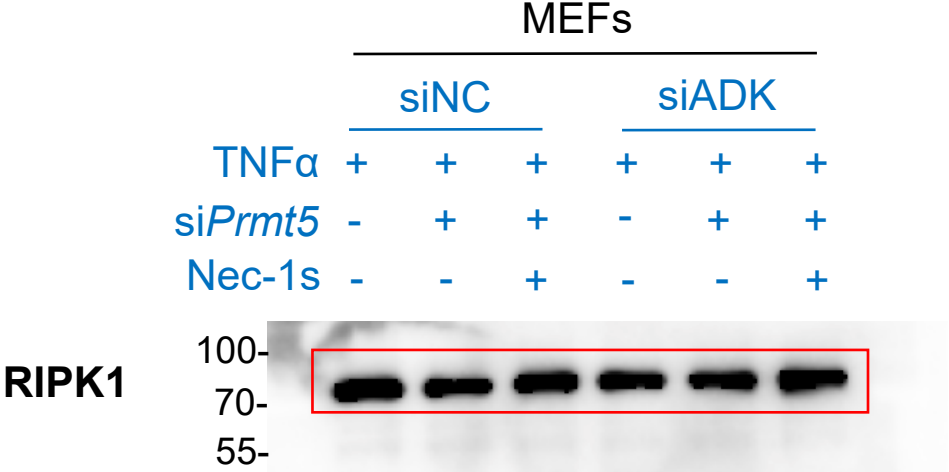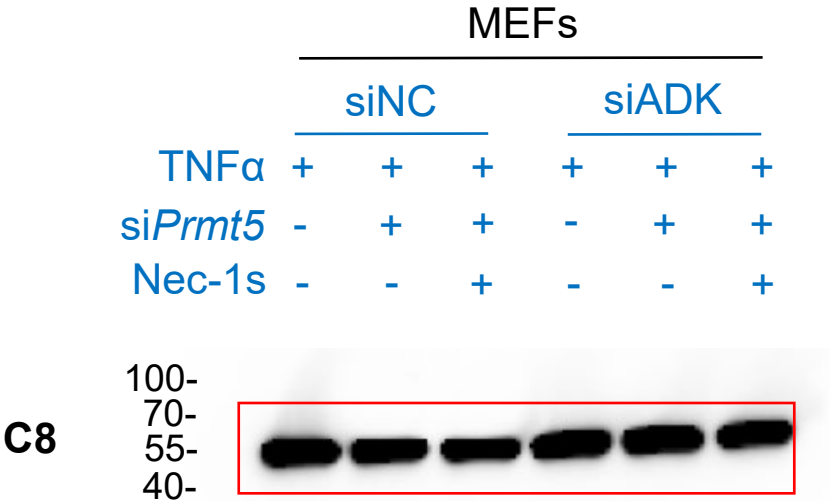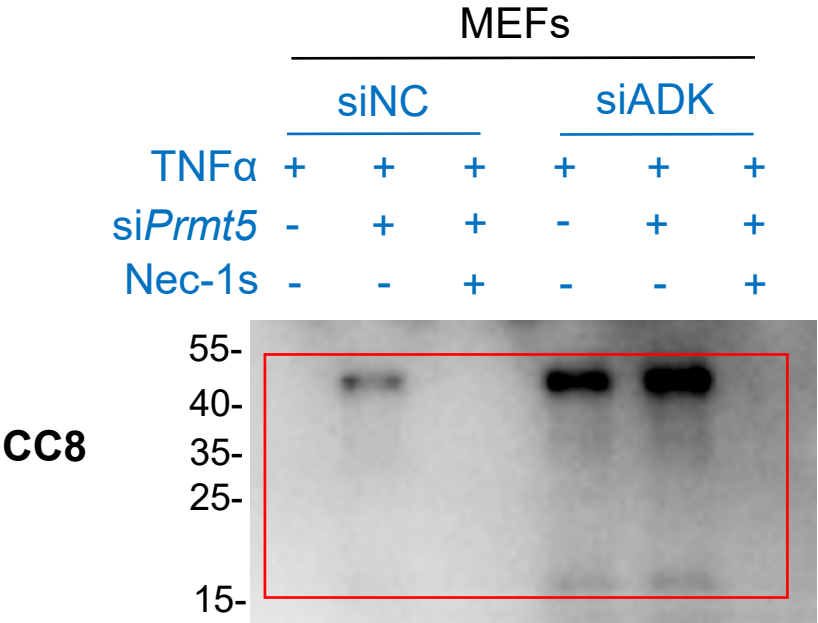

Panel F

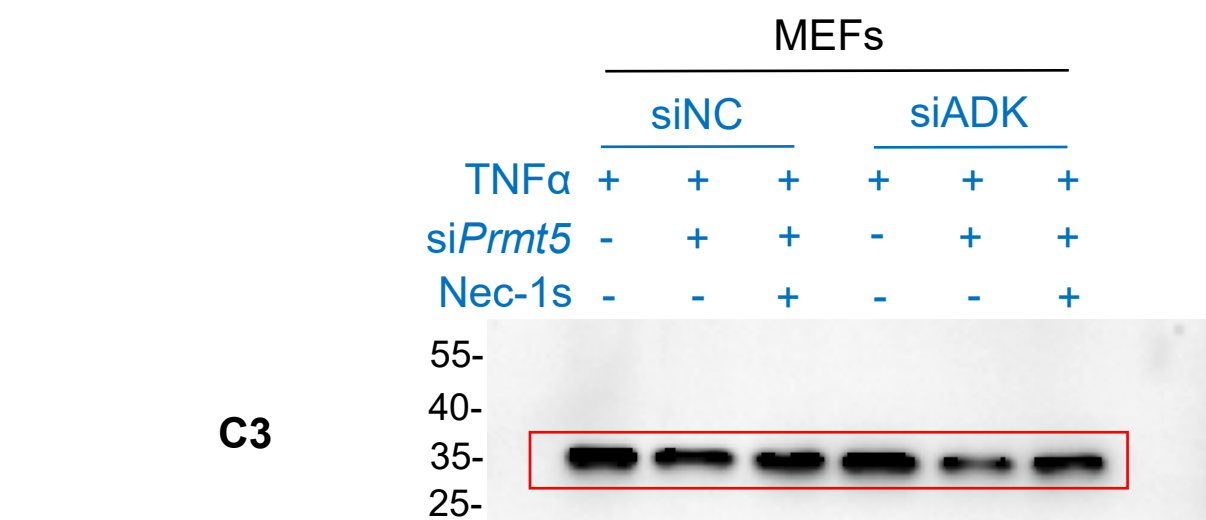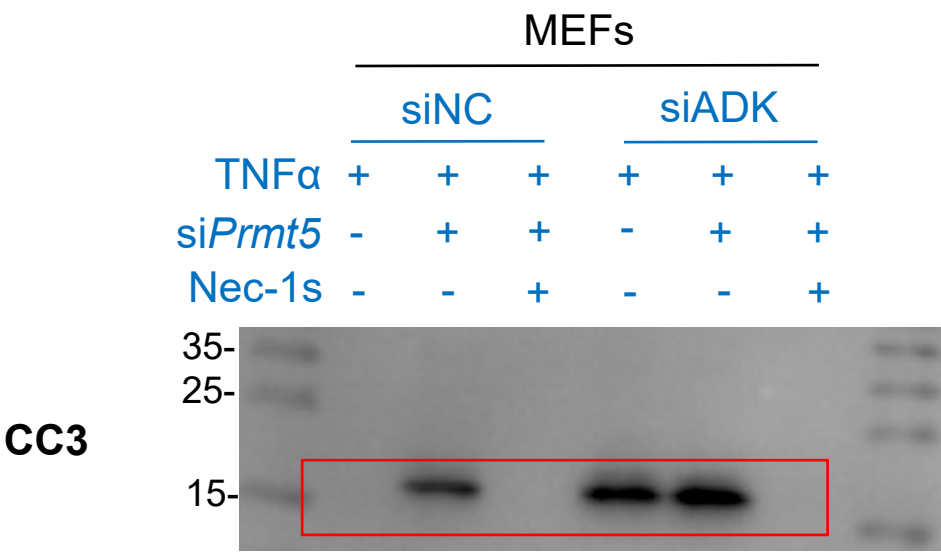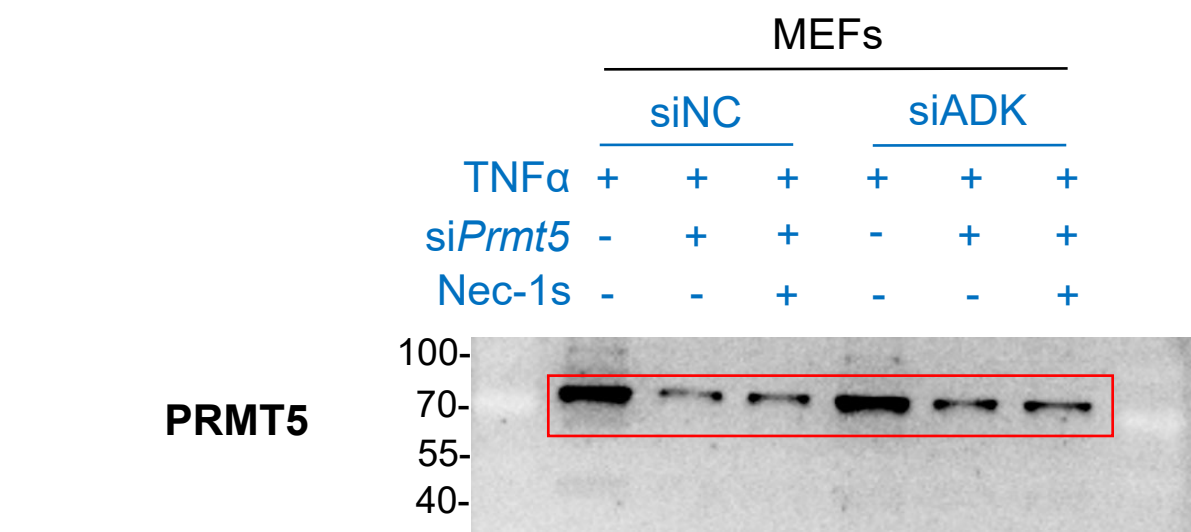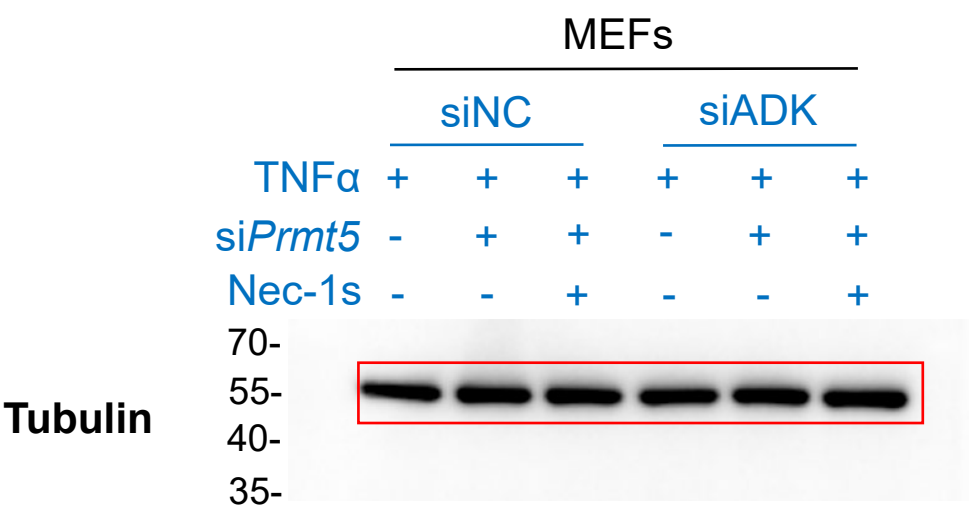

Panel H

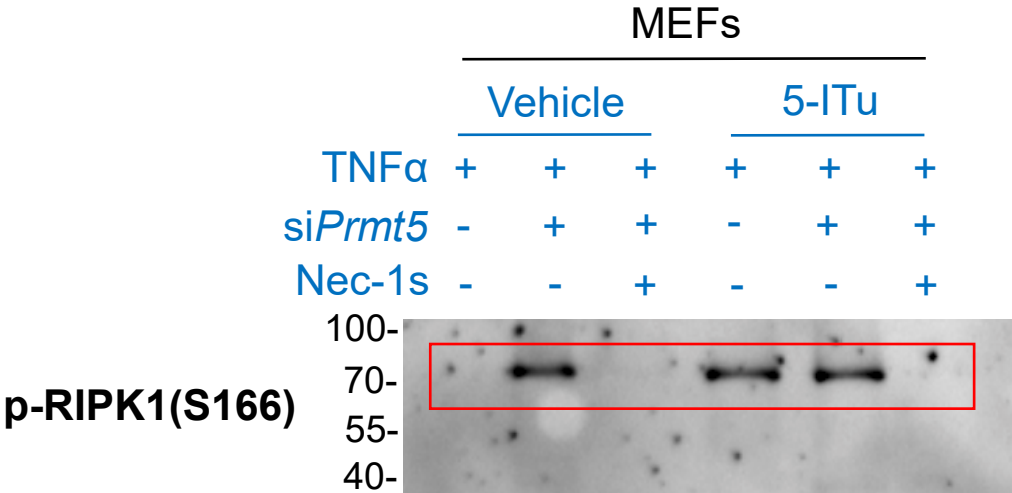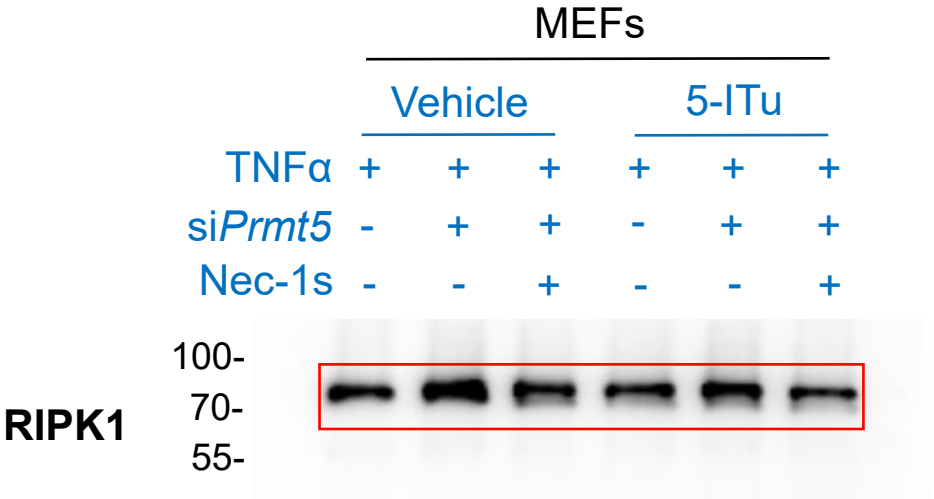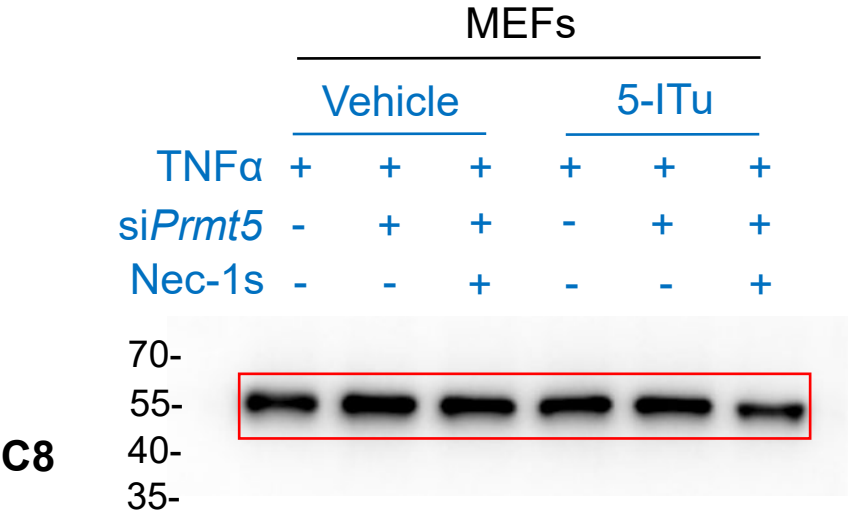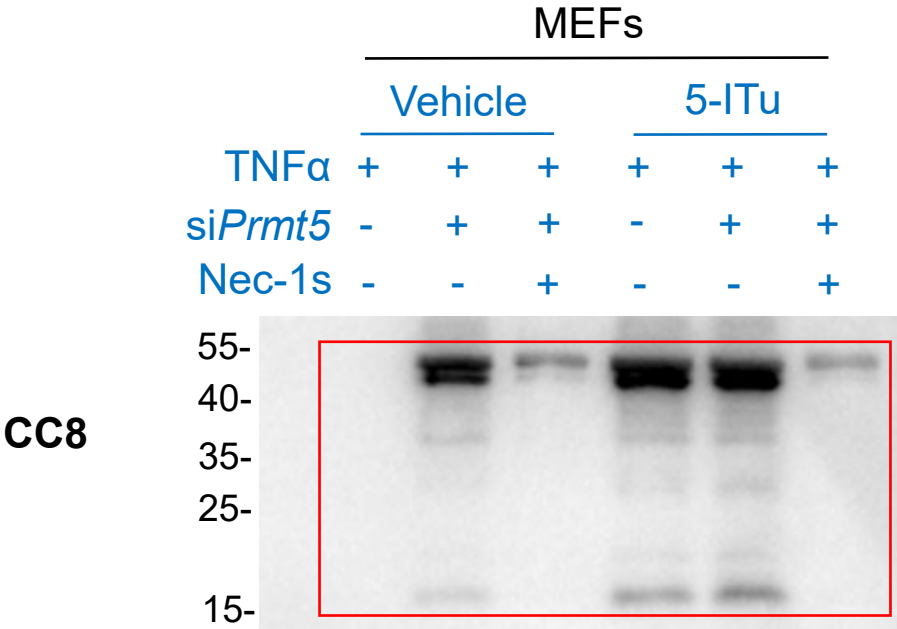

Panel H

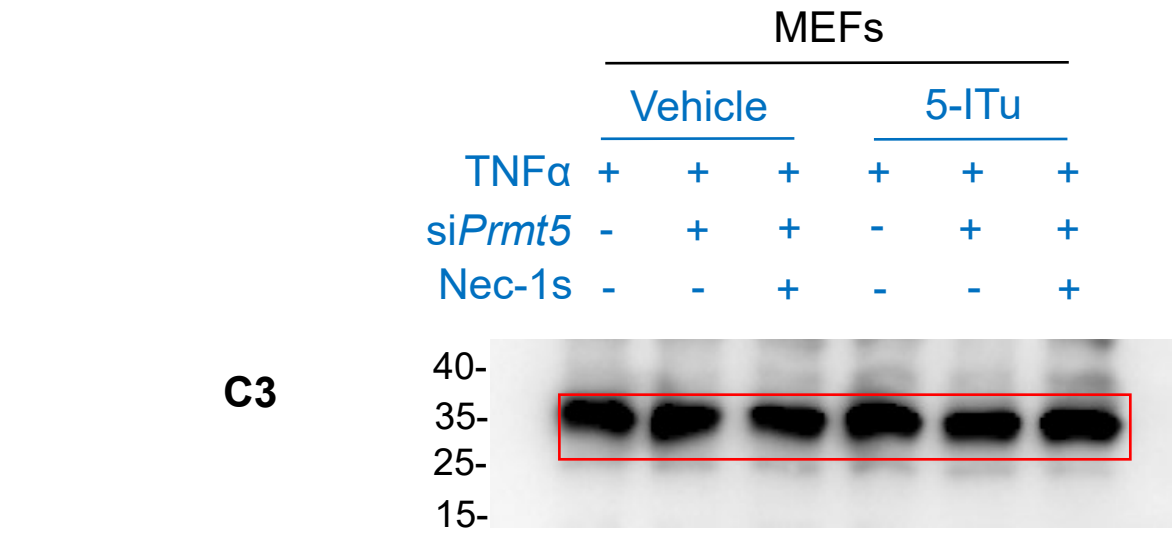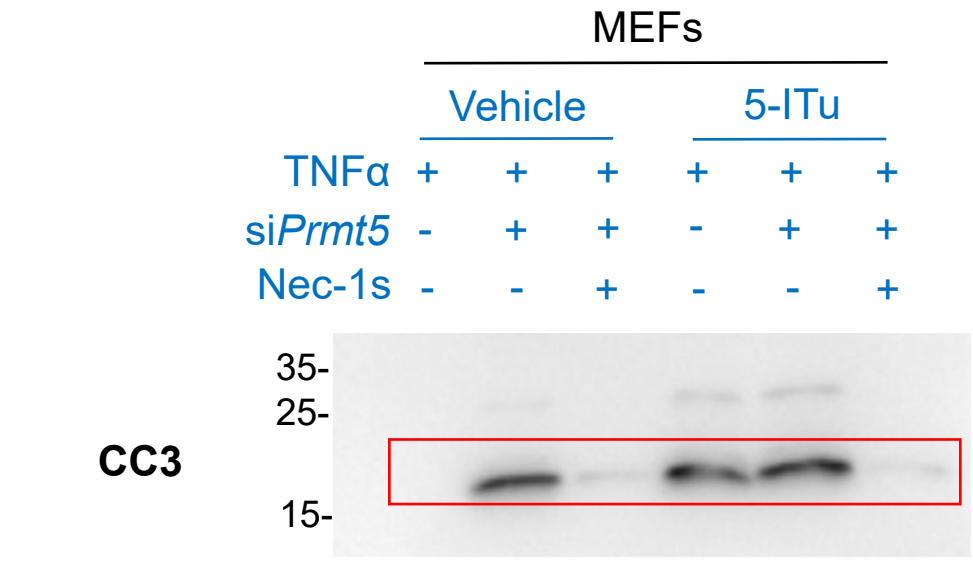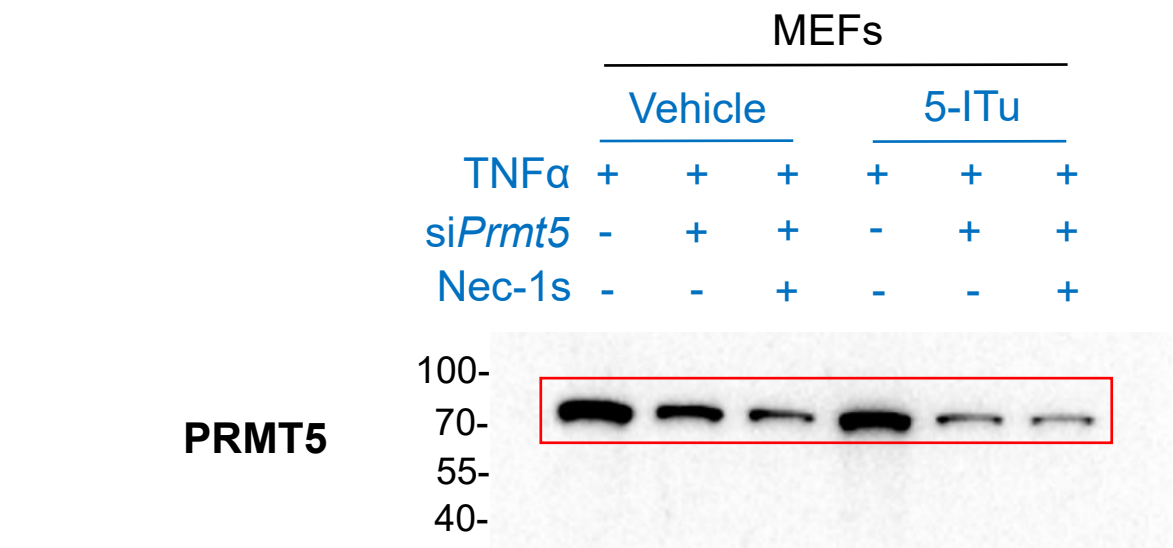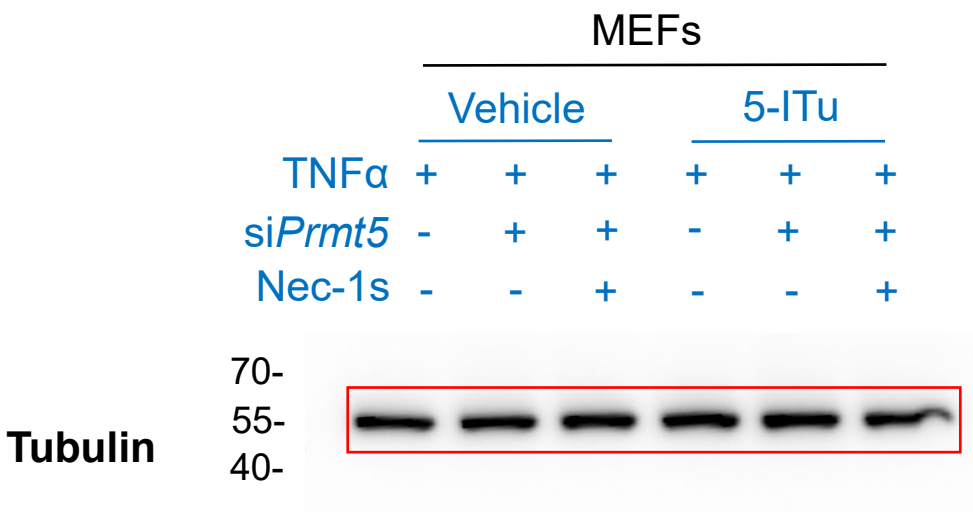

Panel J

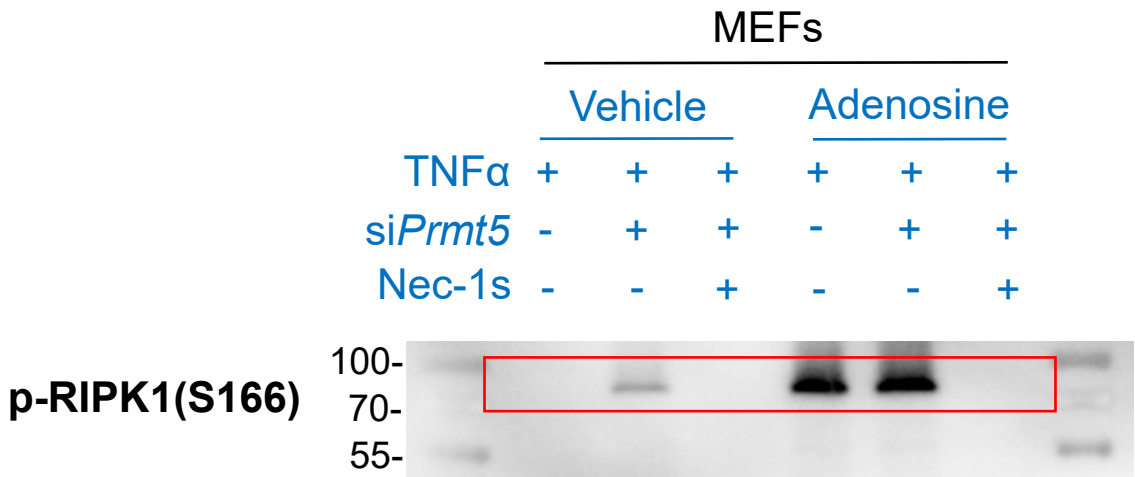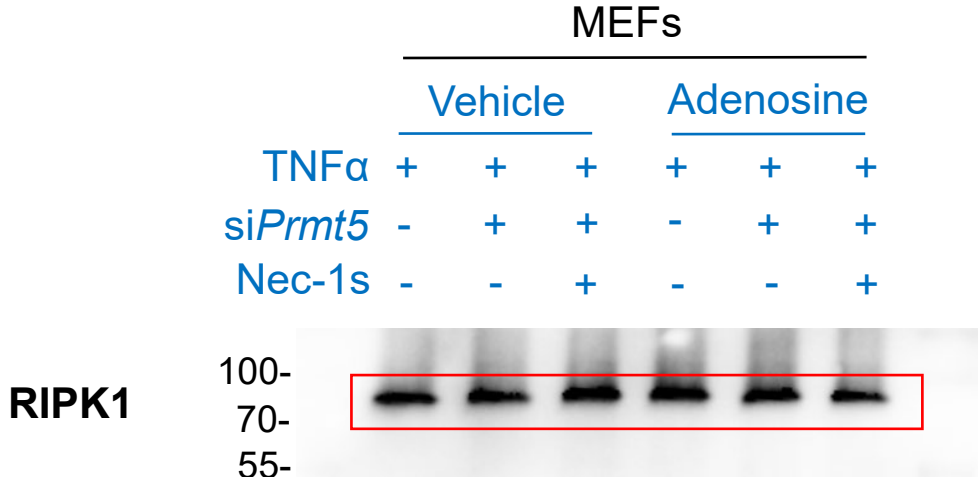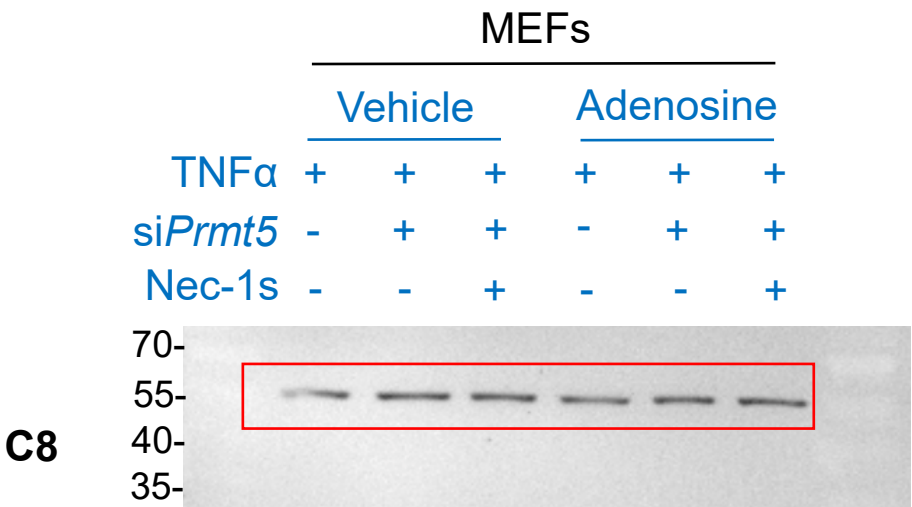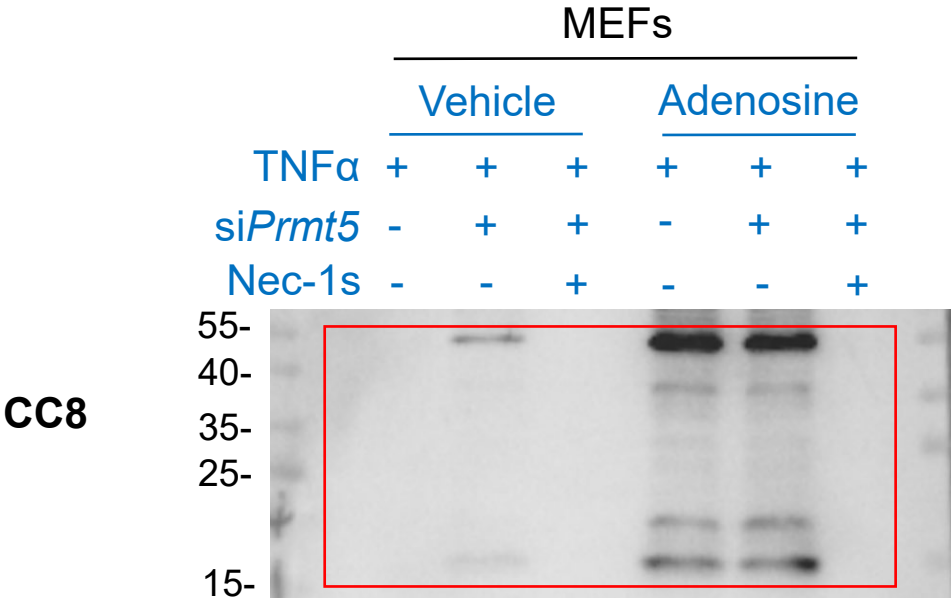

Panel J

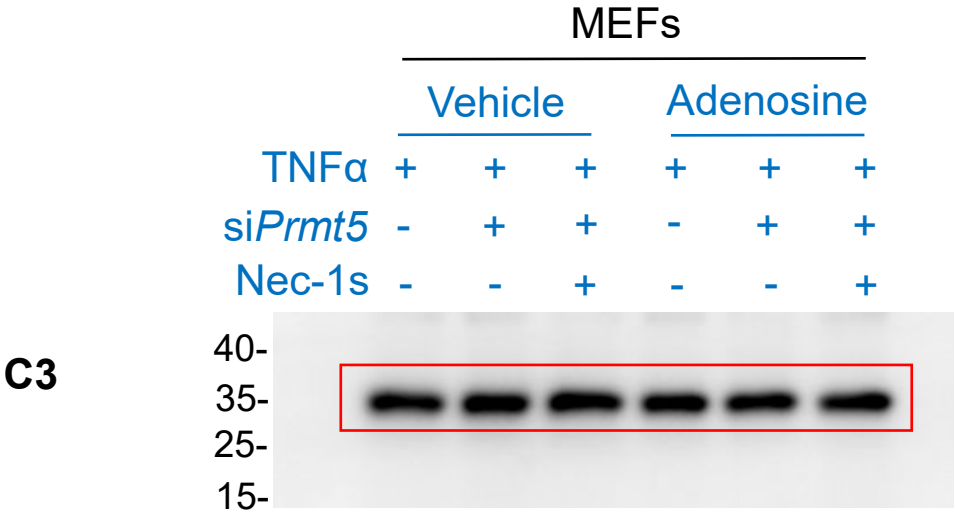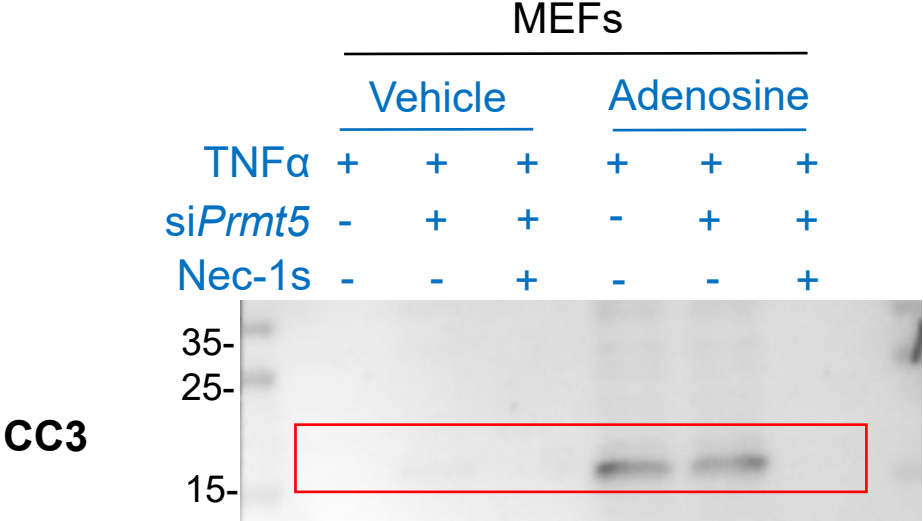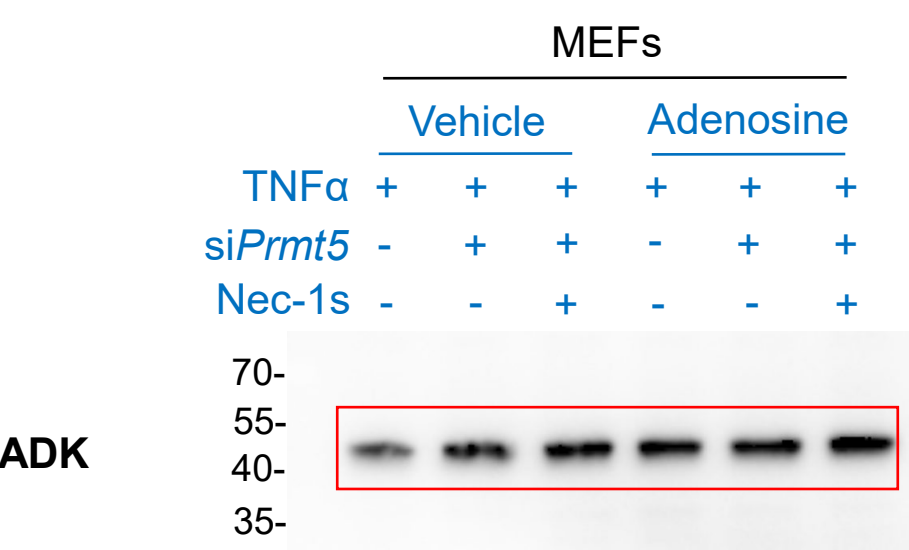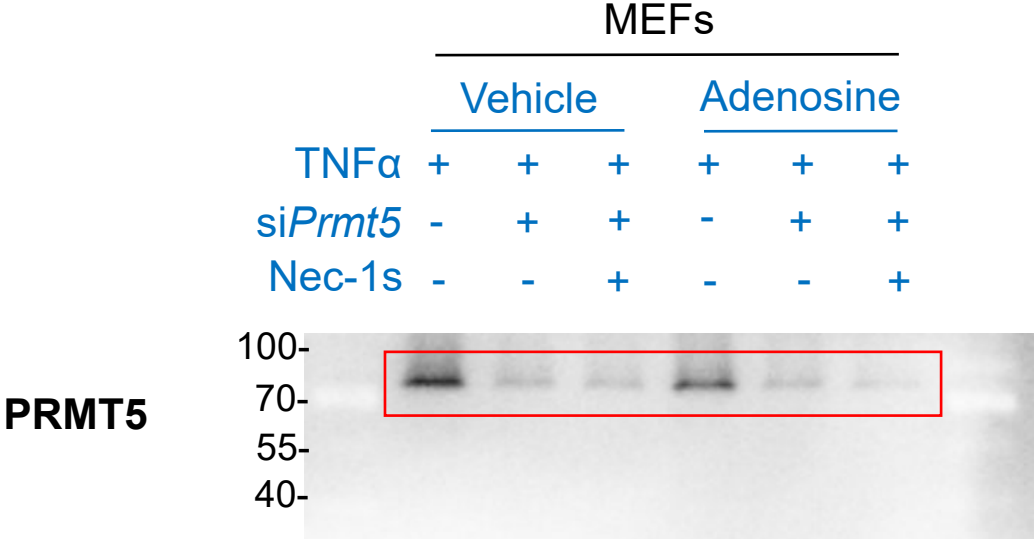

Panel J

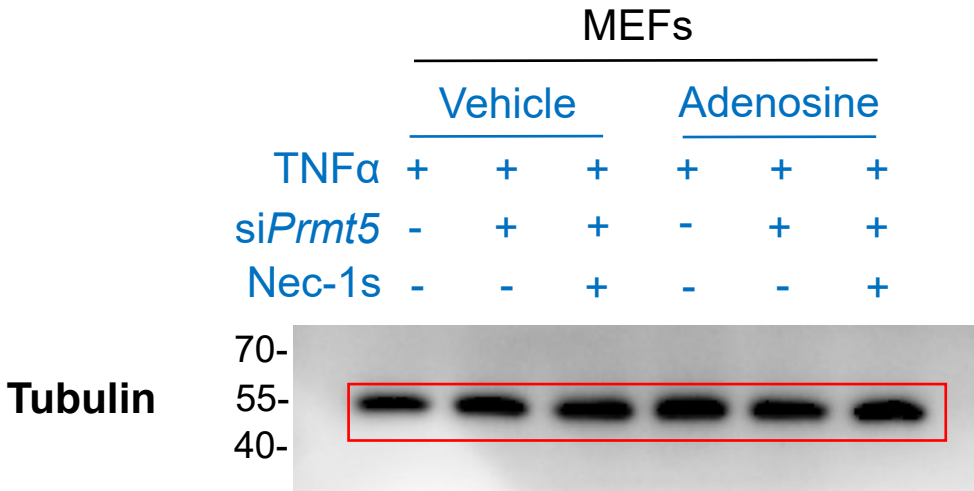

Supplement: SourceData FS3 — is the source file for Fig. S3. [file jem_20250603_sourcedatafs3.pdf]

Panel B

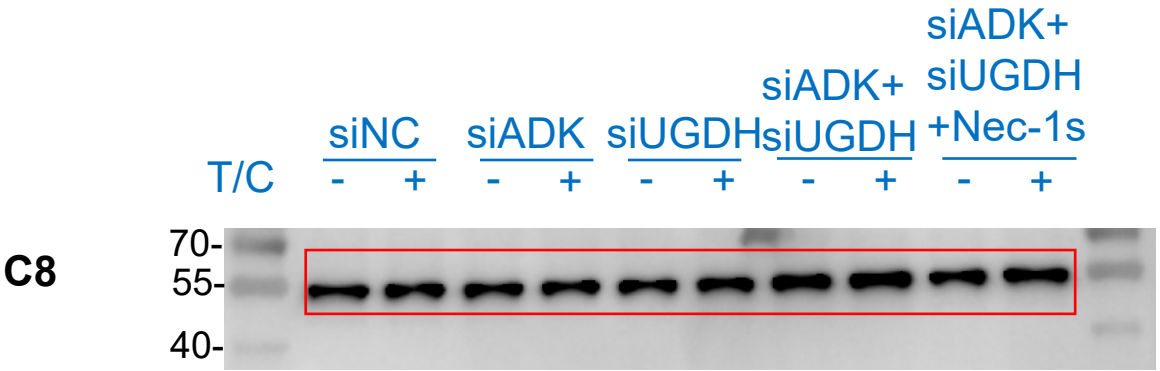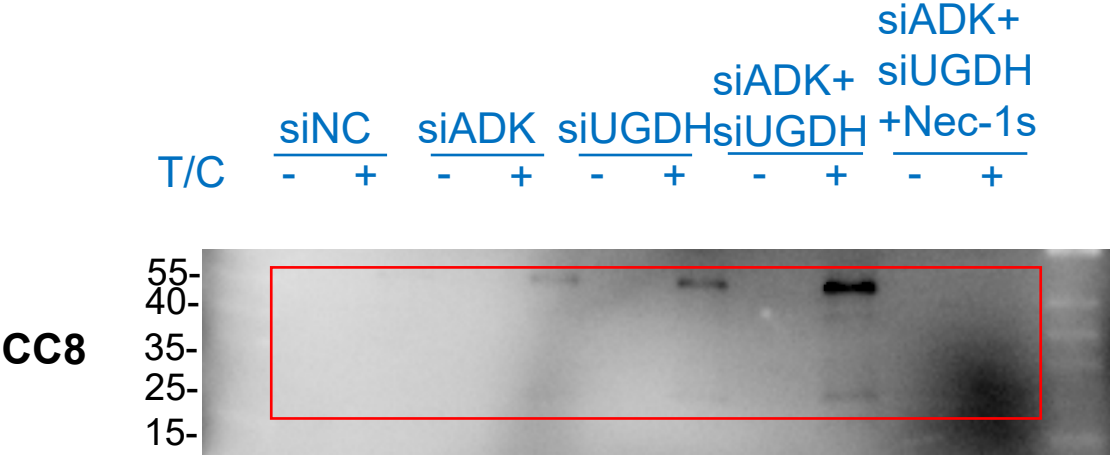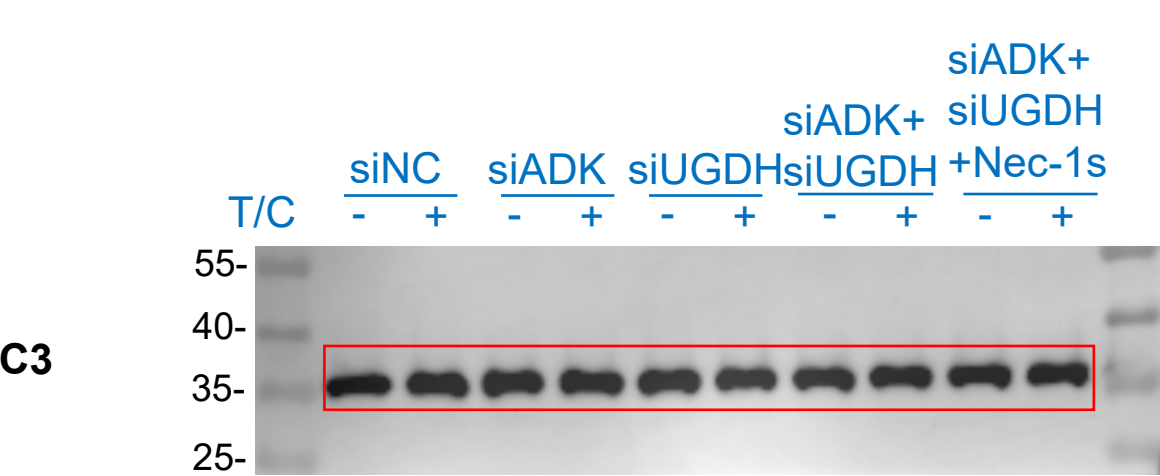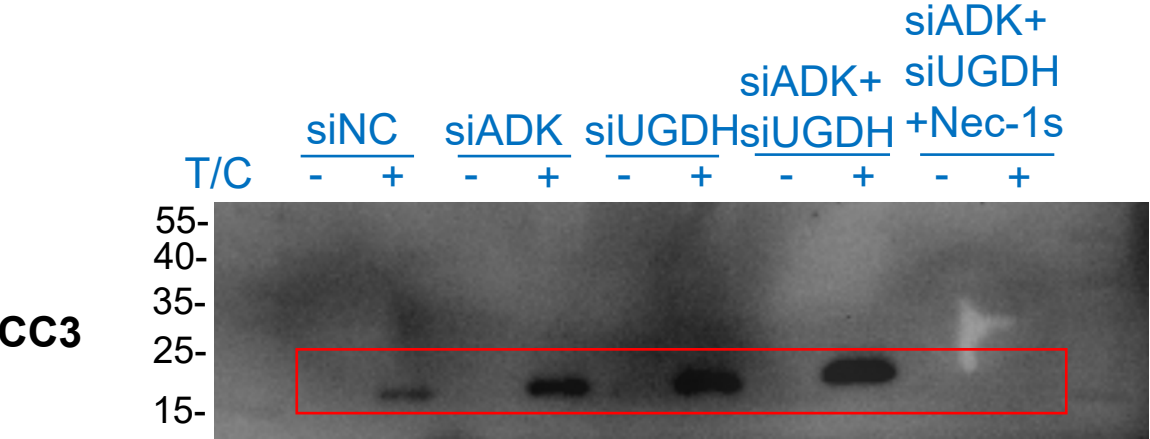

Panel B

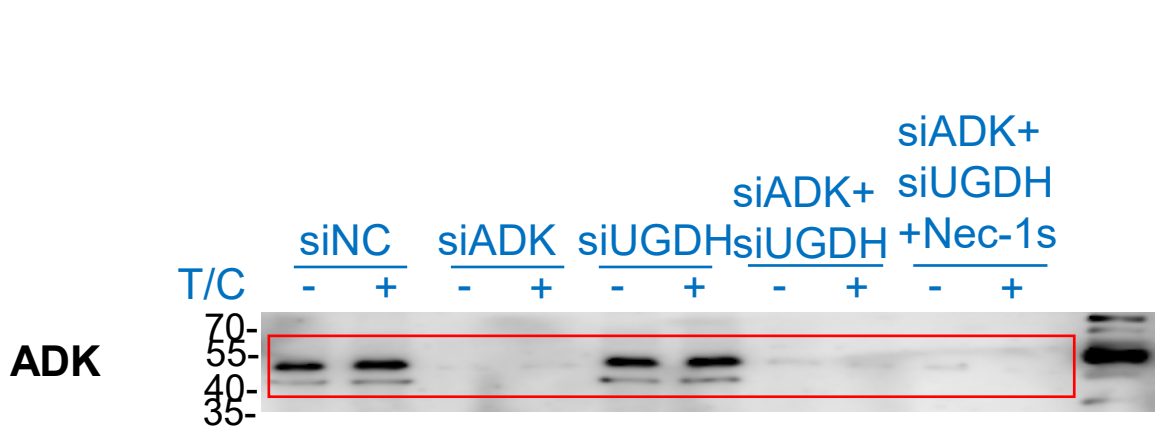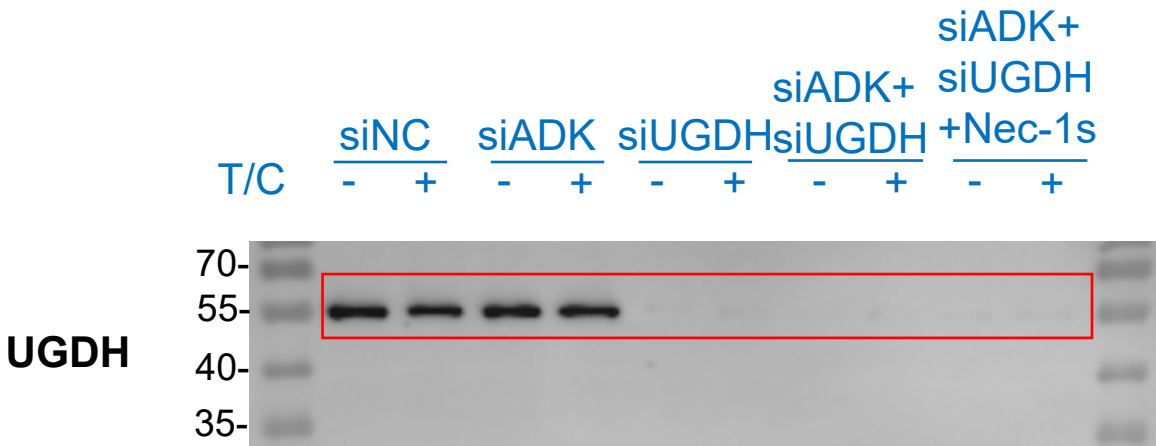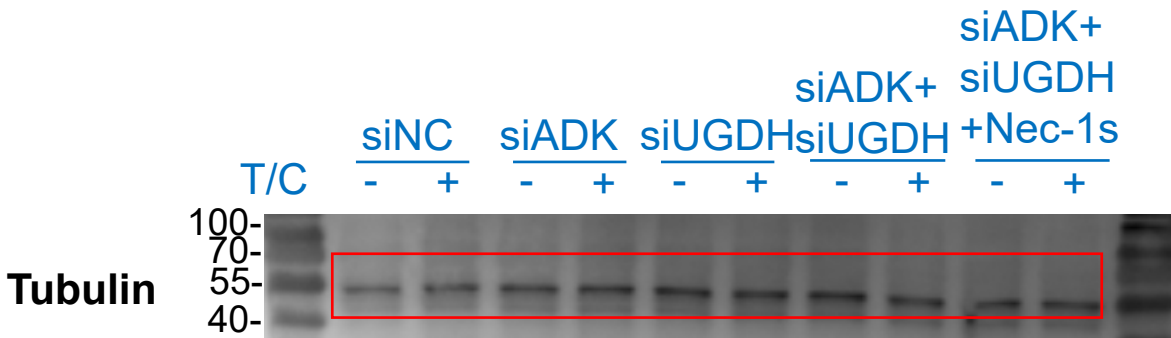

Panel H

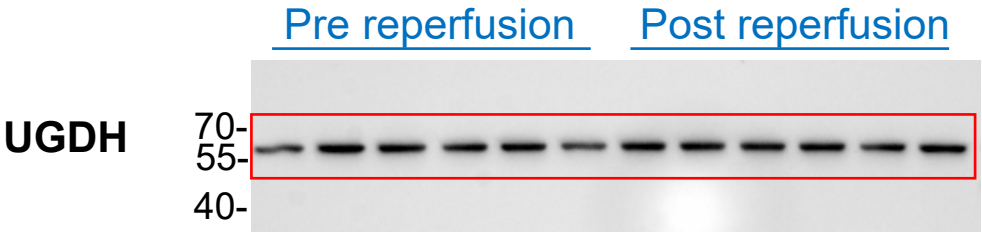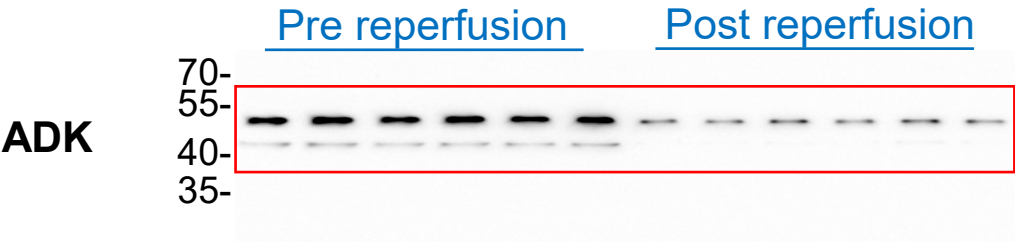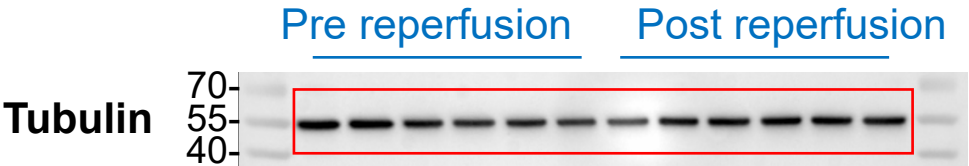

Panel N

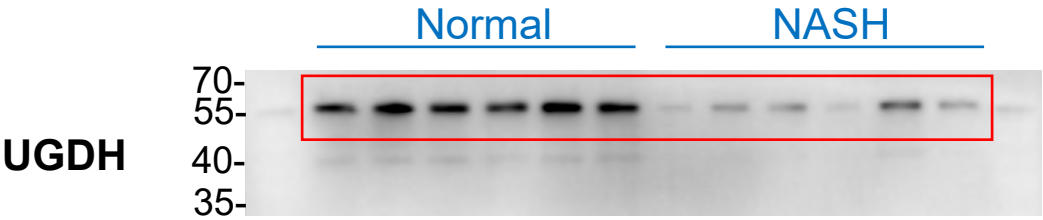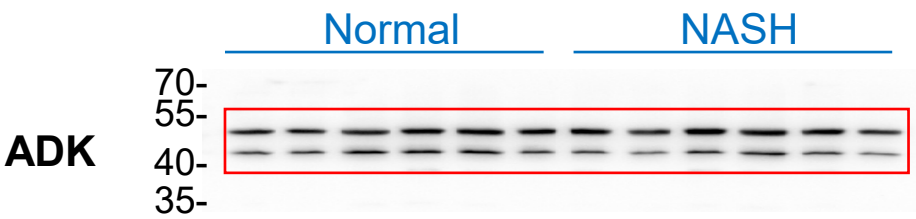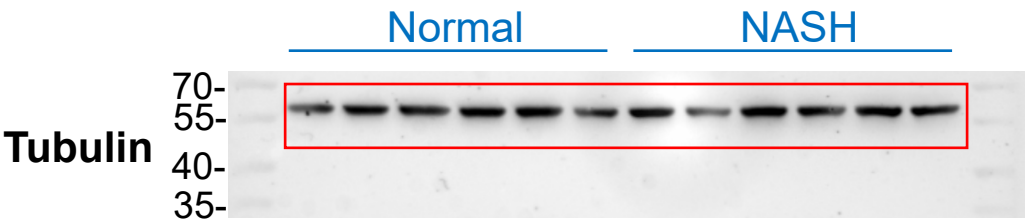

Supplement: SourceData FS5 — is the source file for Fig. S5. [file jem_20250603_sourcedatafs5.pdf]
