# Supplementary material for: Adenosine metabolic clearance maintains liver homeostasis by licensing arginine methylation of RIPK1
Source: J Exp Med. 2025 Oct 13;223(1):e20250603. doi: 10.1084/jem.20250603 (PMC12517274; doi:10.1084/jem.20250603)

Uncropped blots_Supplemental Figure 1

Panel B

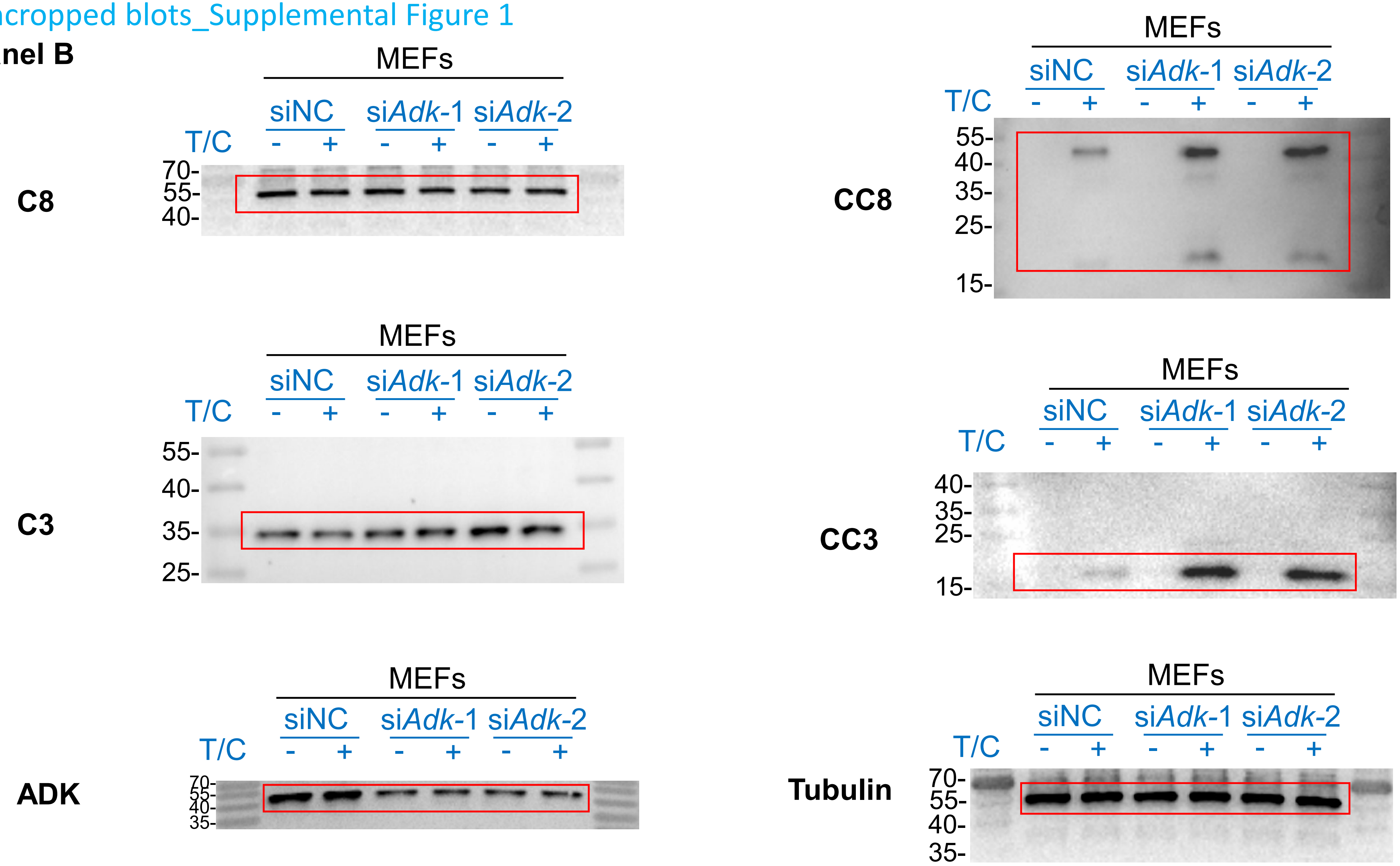

Uncropped blots_Supplemental Figure 1

Panel D

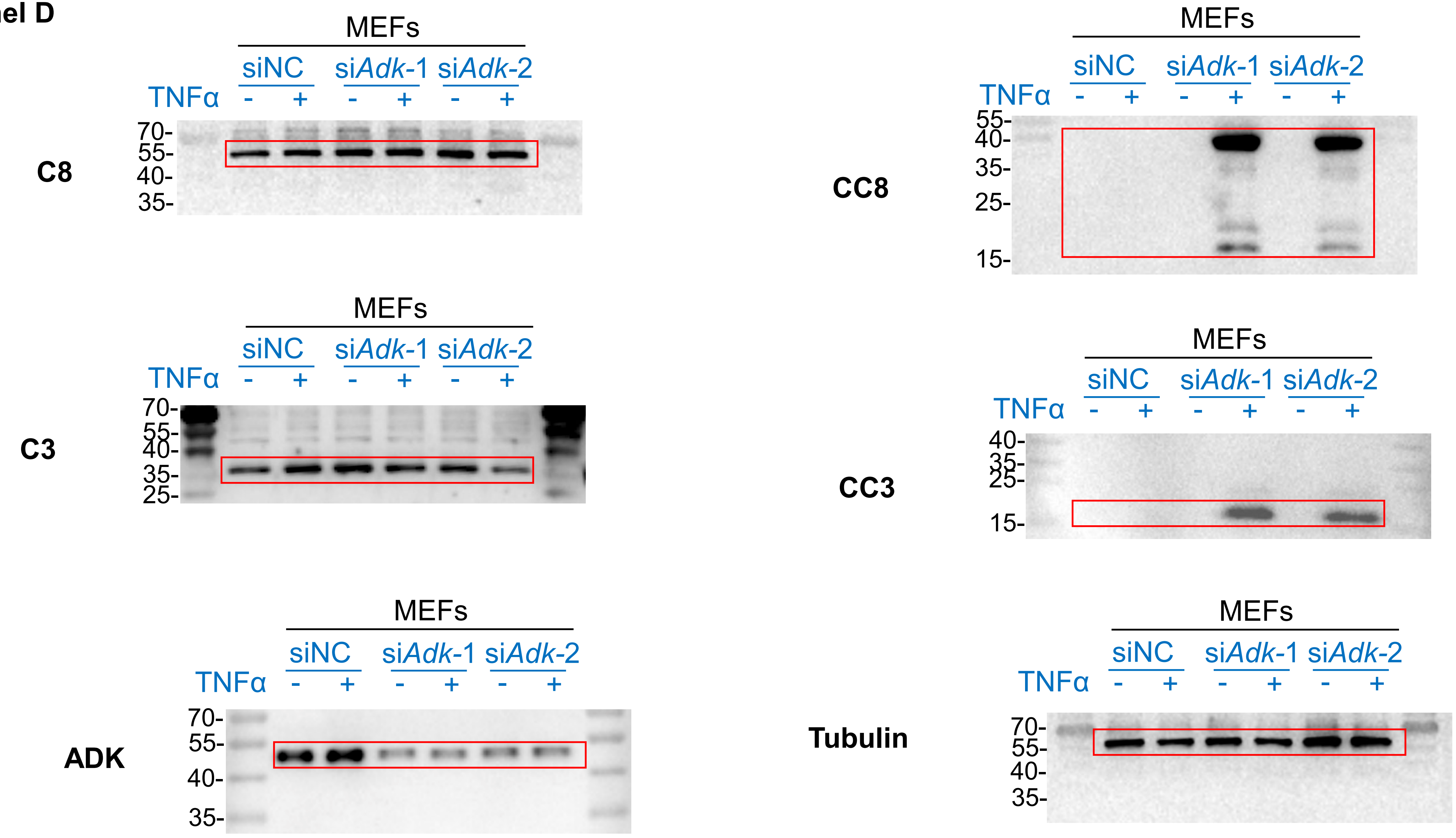

Uncropped blots_Supplemental Figure 1

Panel E

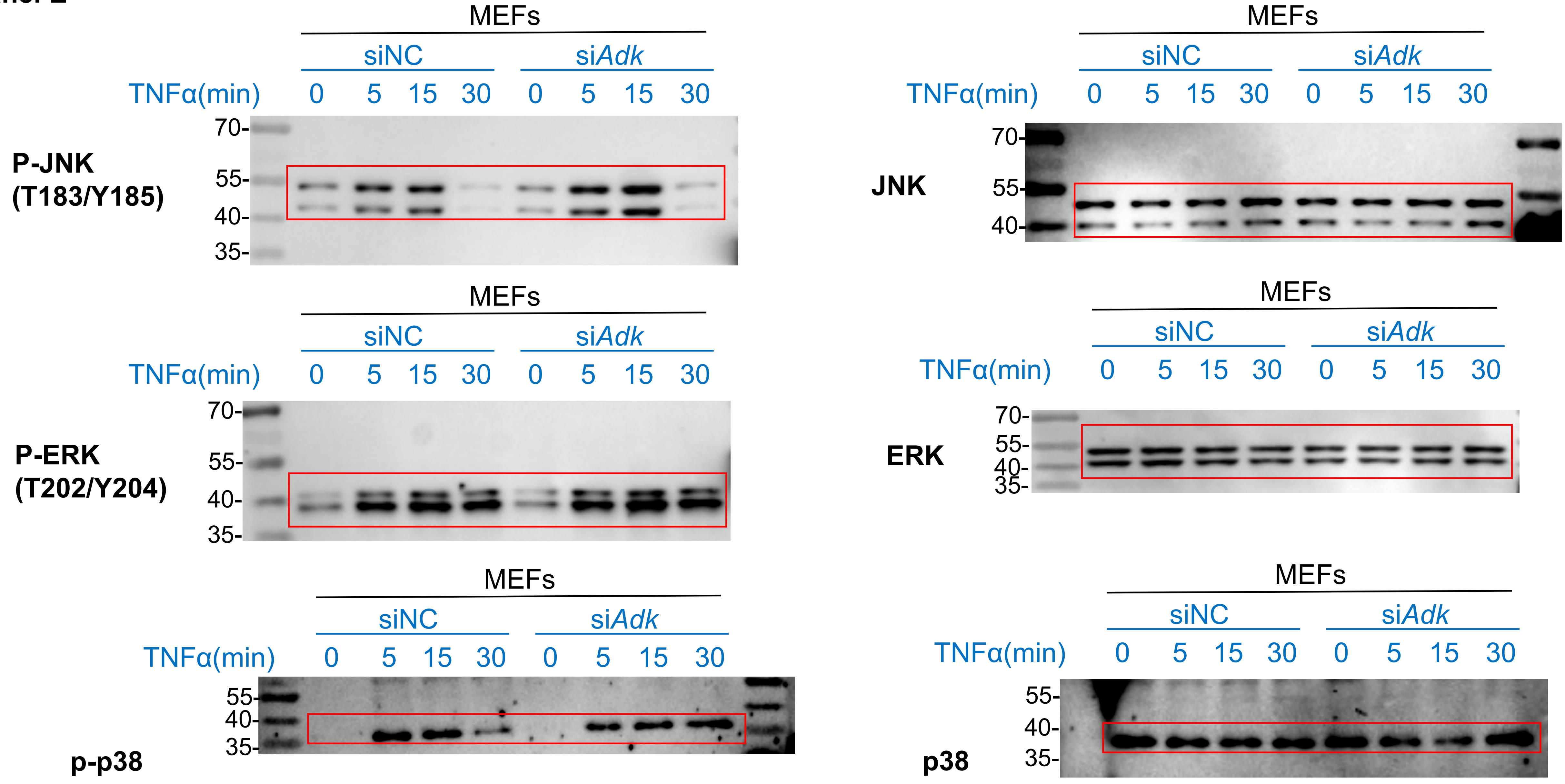

Uncropped blots_Supplemental Figure 1

Panel E

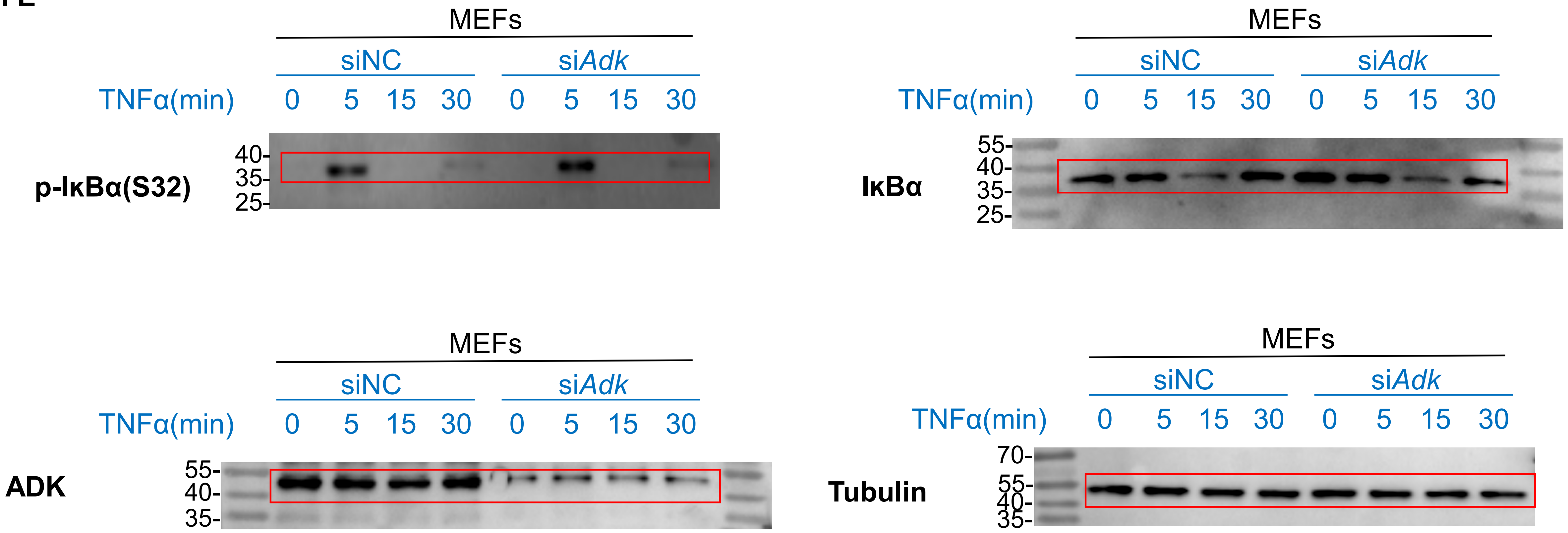

Uncropped blots_Supplemental Figure 1

Panel H

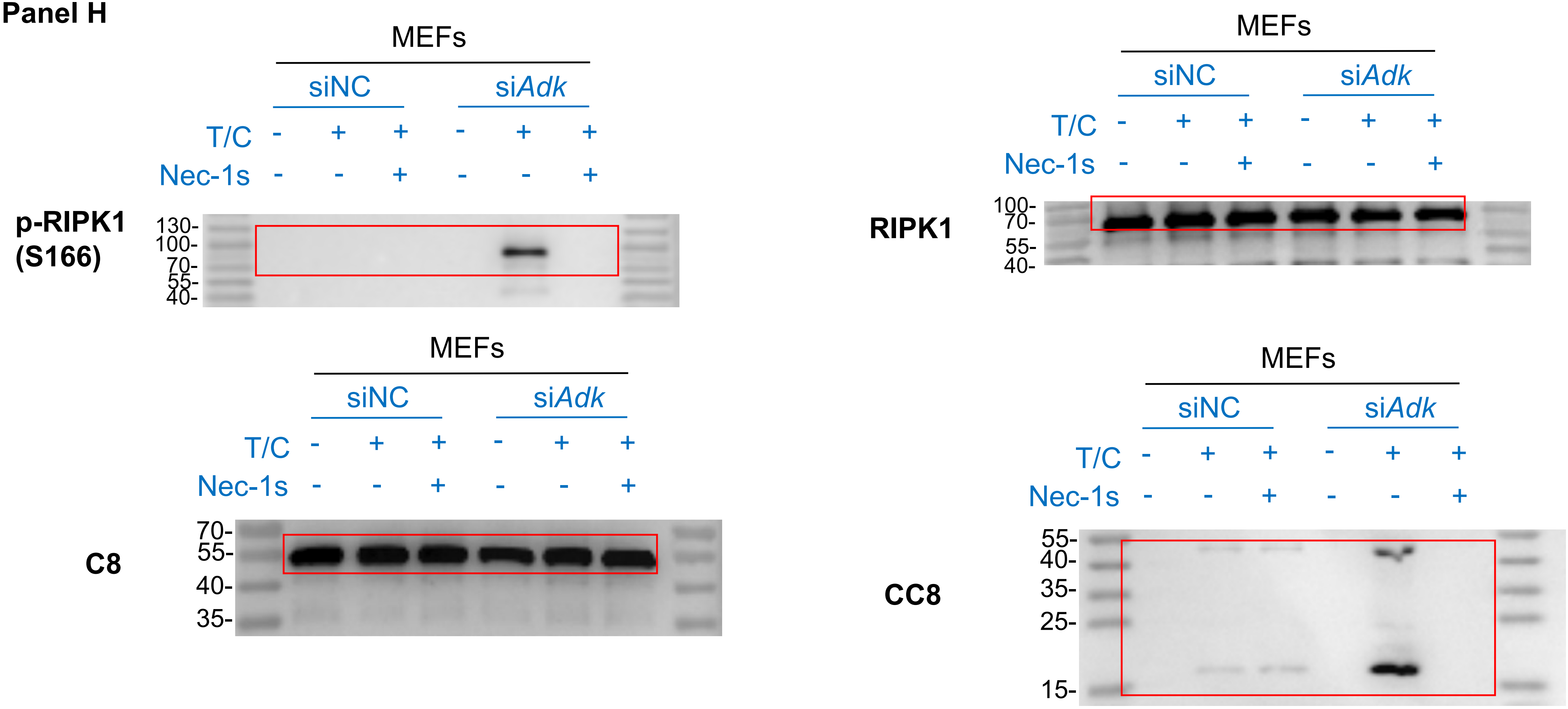

Uncropped blots_Supplemental Figure 1

Panel H

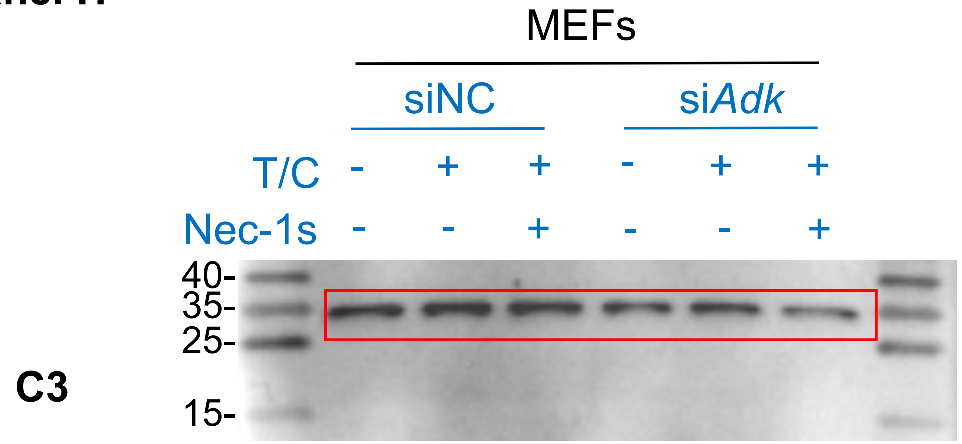

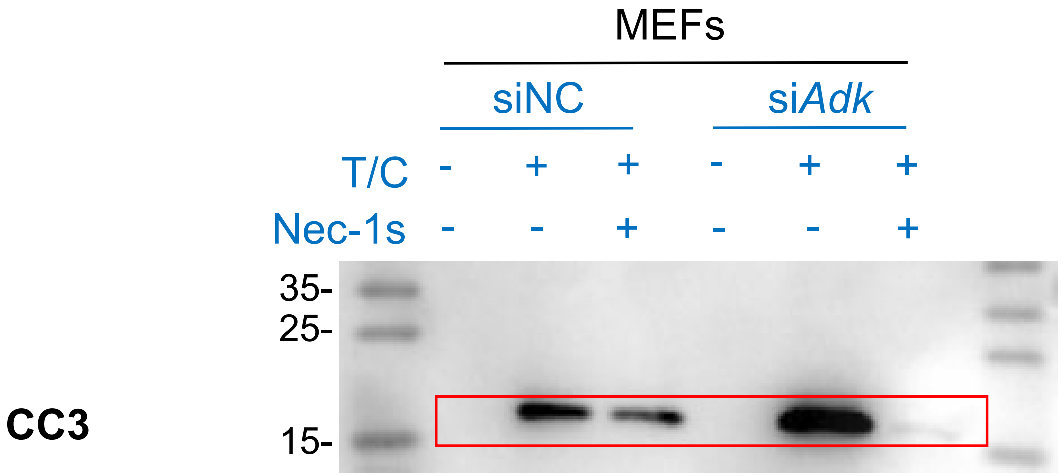

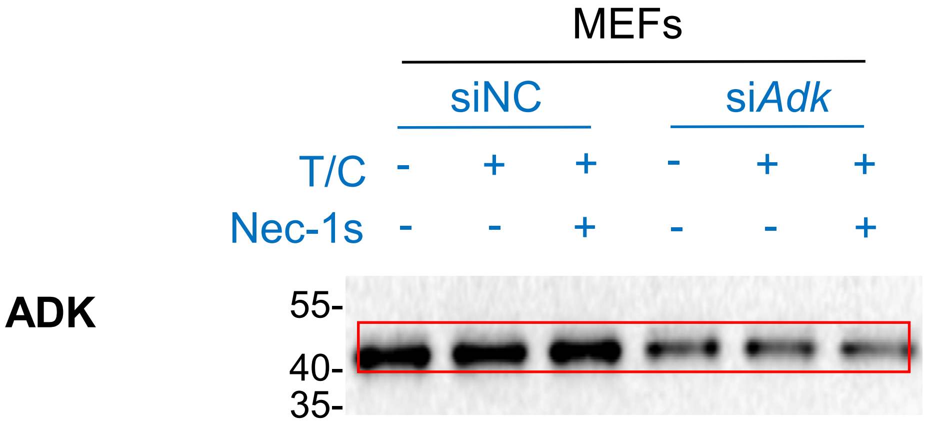

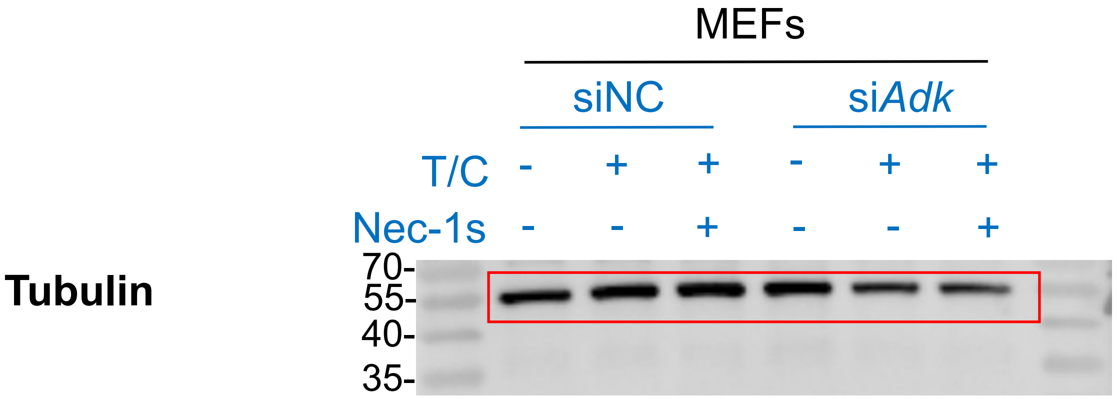

Uncropped blots_Supplemental Figure 1

Panel J

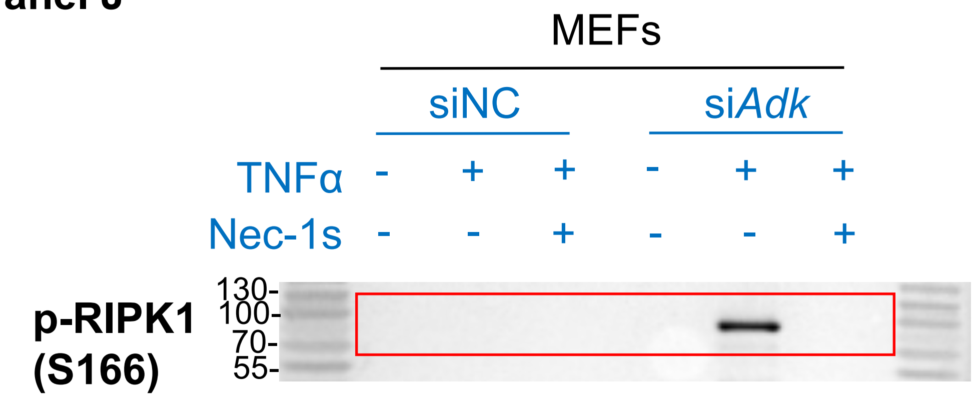

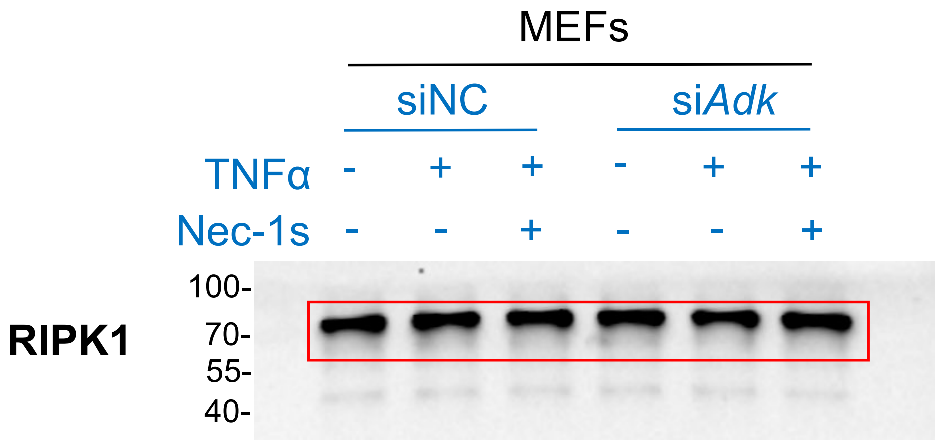

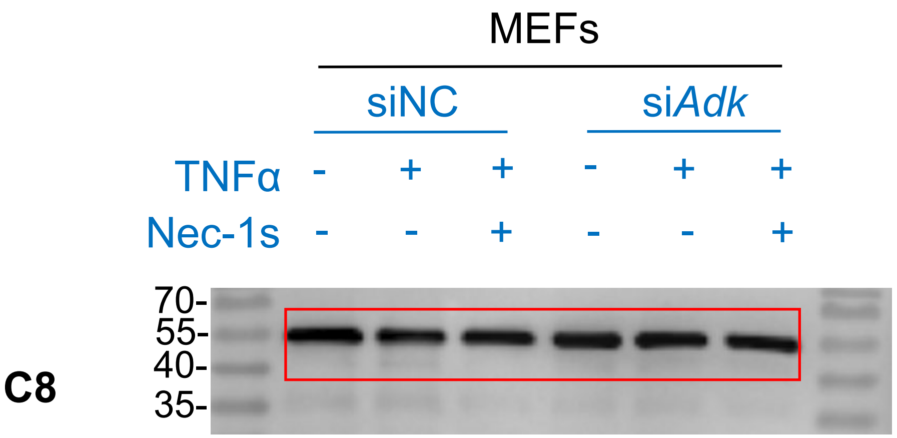

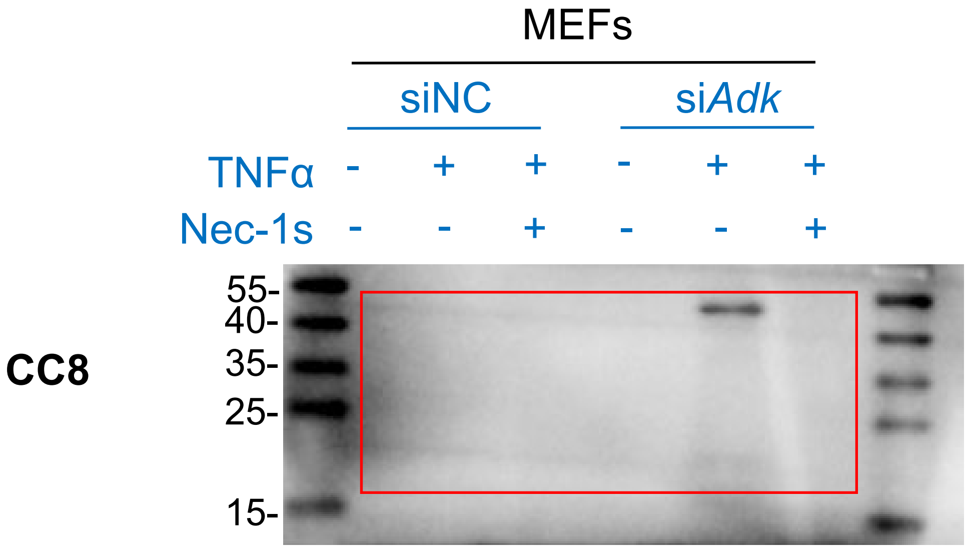

Uncropped blots_Supplemental Figure 1

Panel J

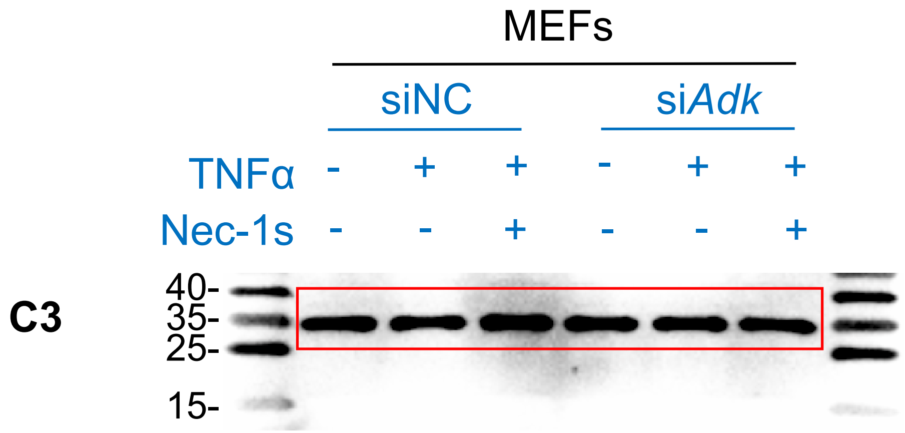

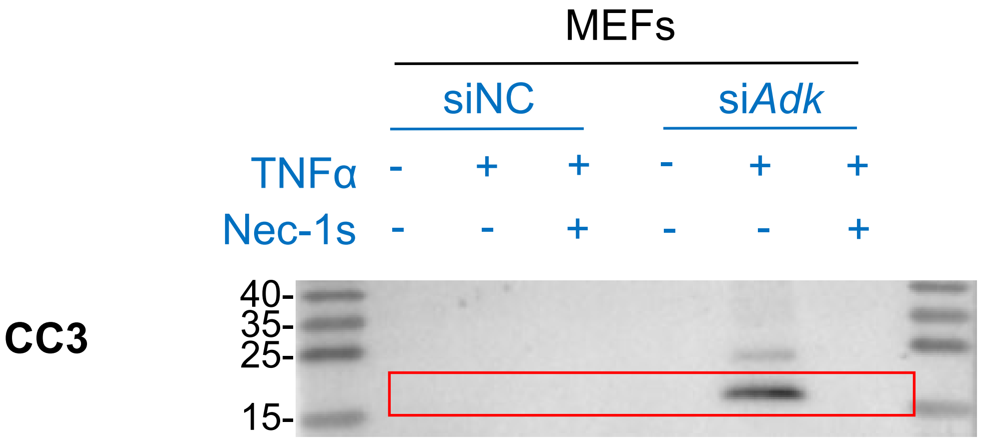

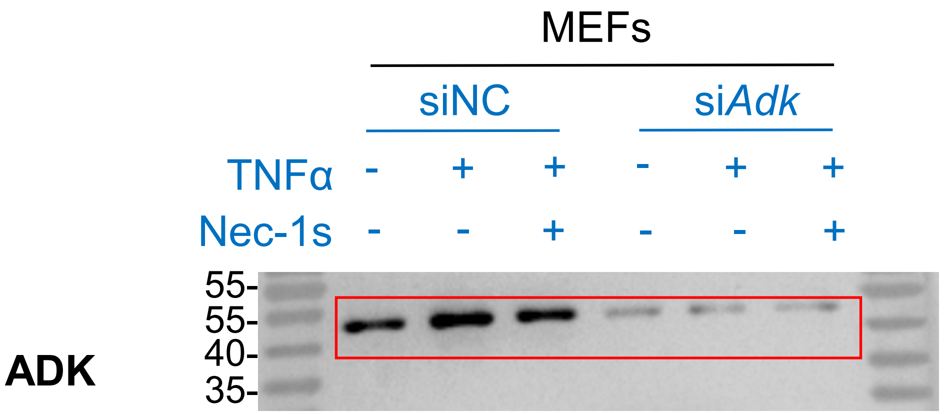

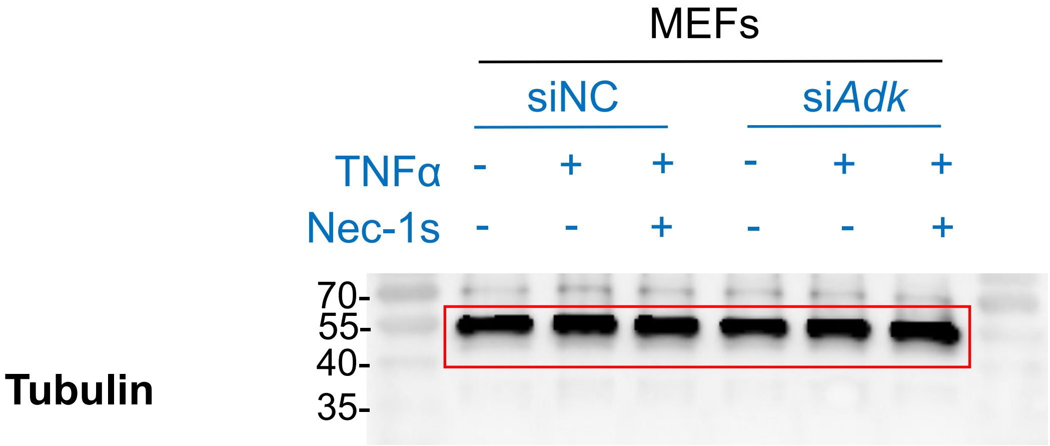

Uncropped blots_Supplemental Figure 1

Panel L

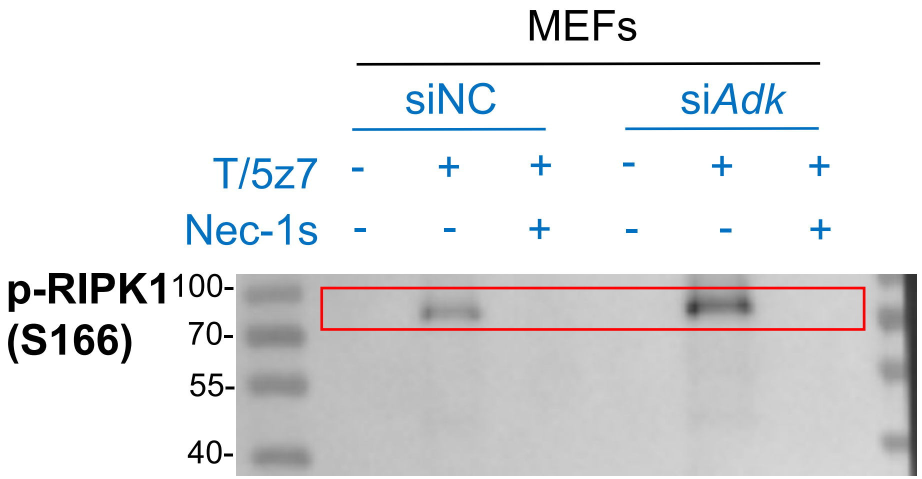

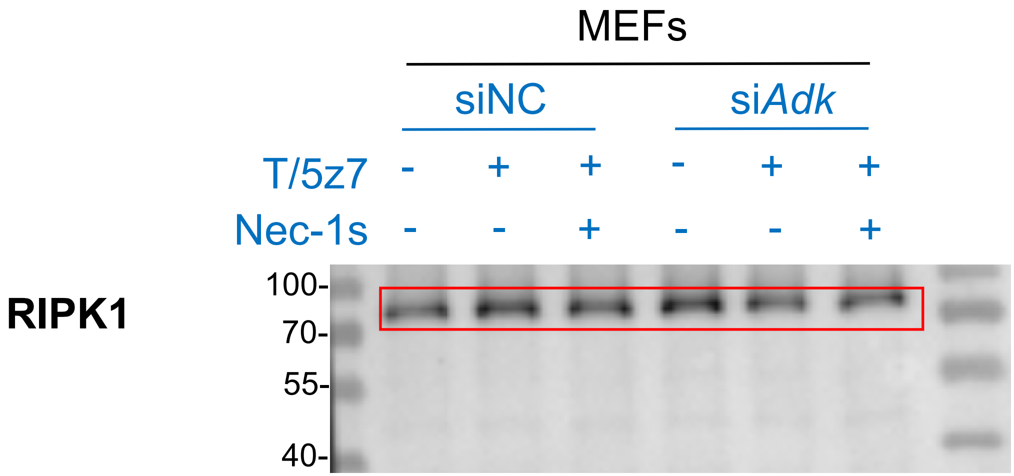

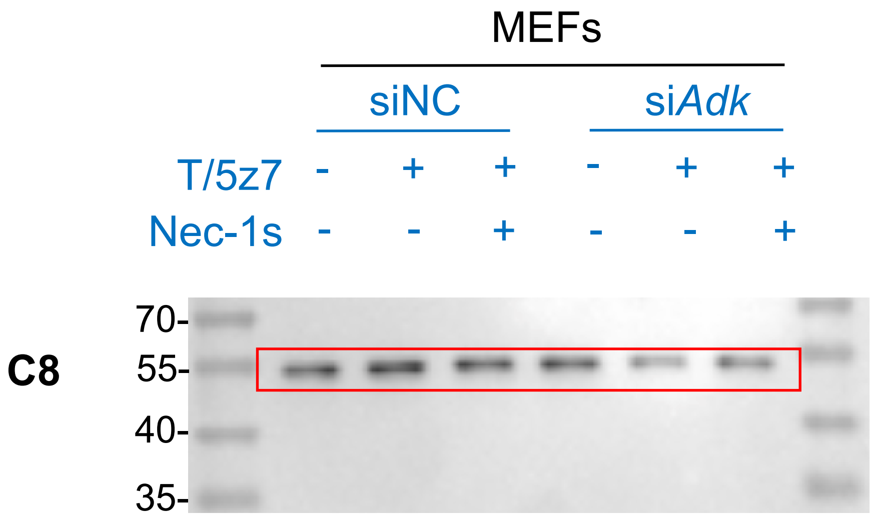

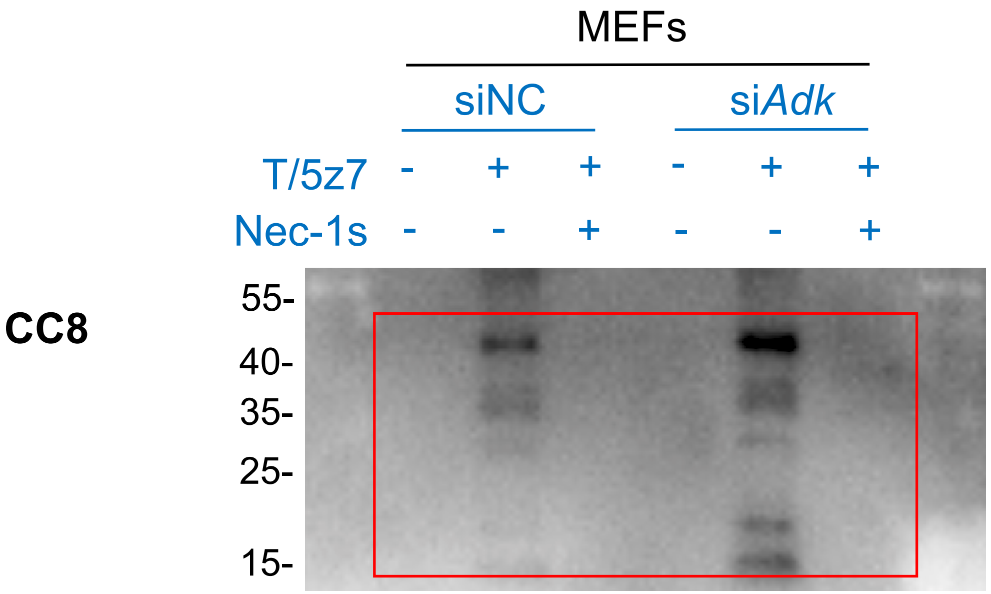

Uncropped blots_Supplemental Figure 1

Panel L

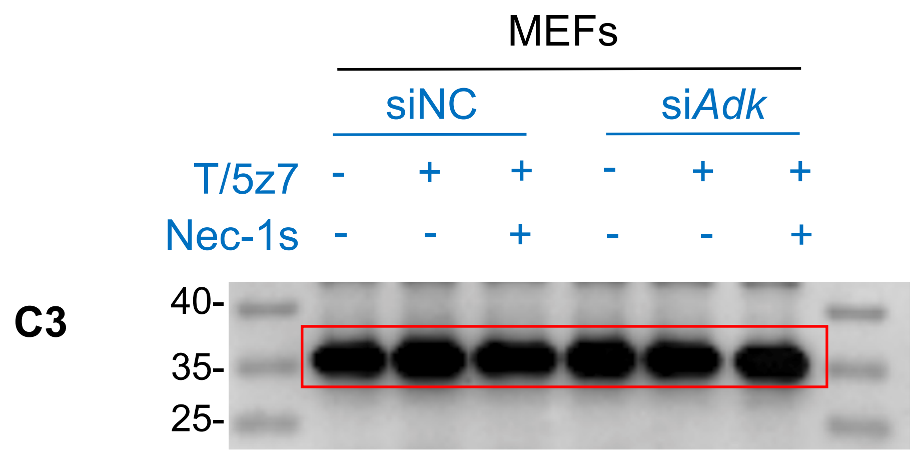

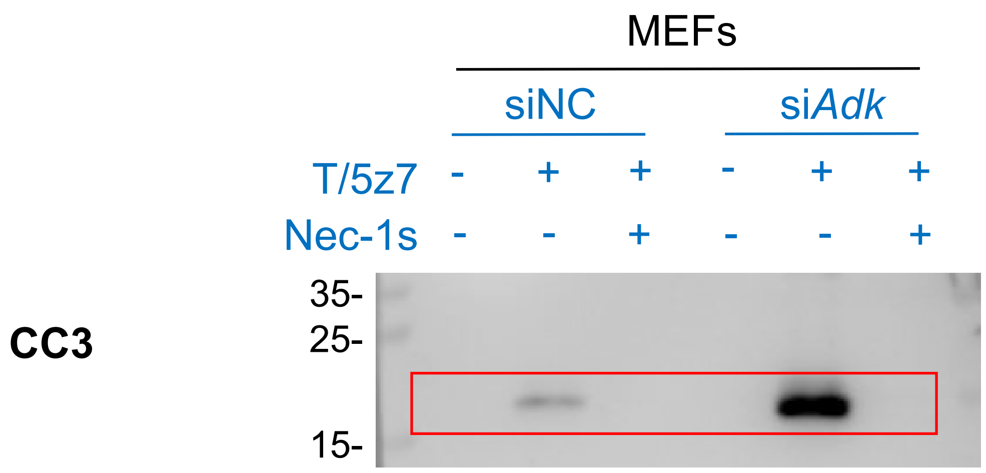

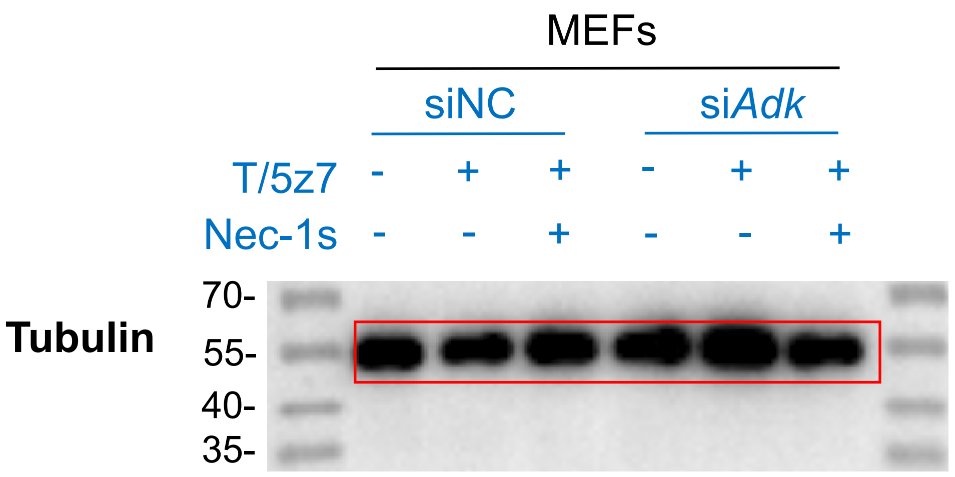

Uncropped blots_Supplemental Figure 1

Panel N

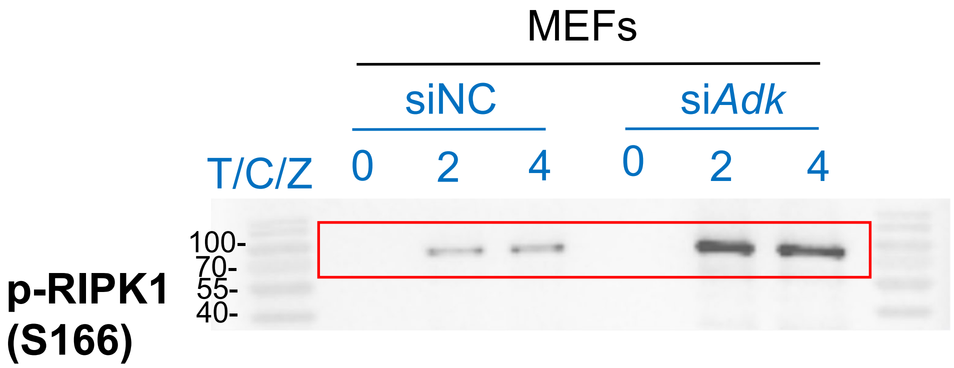

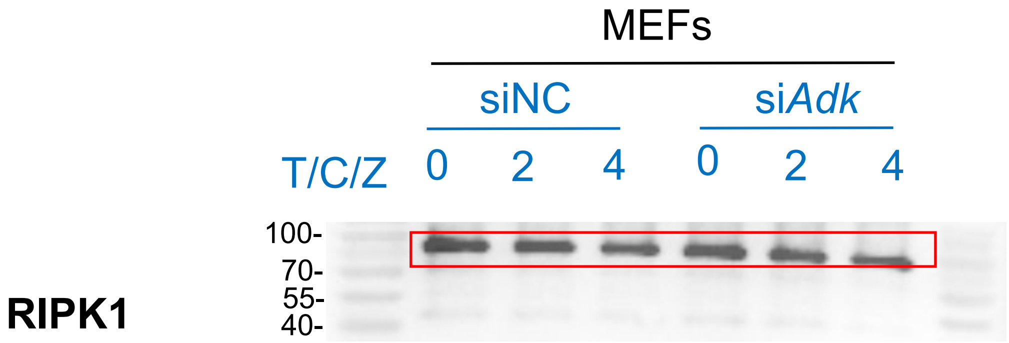

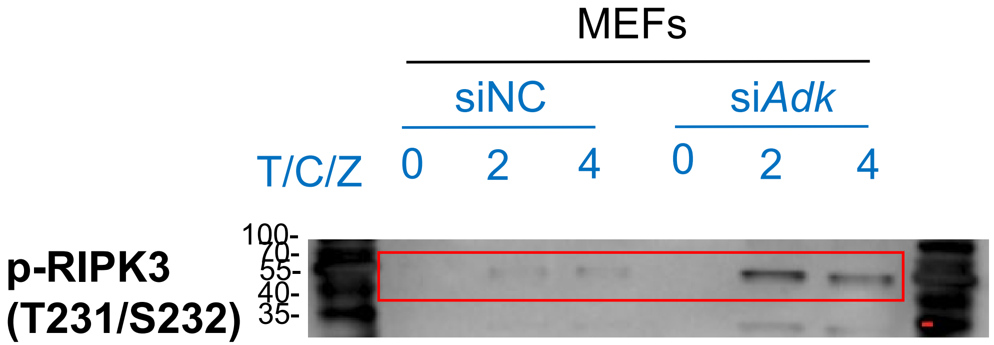

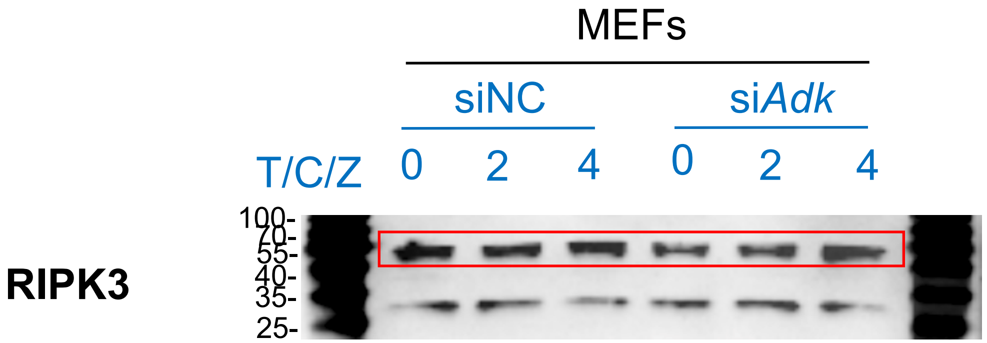

Uncropped blots_Supplemental Figure 1

Panel N

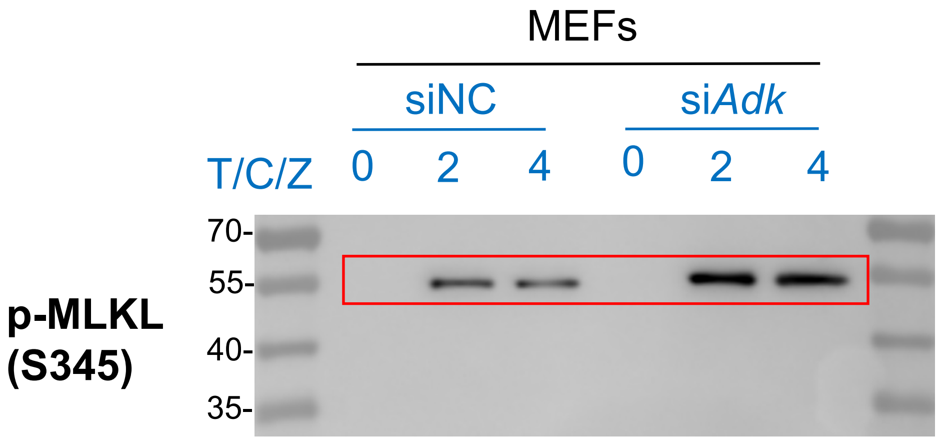

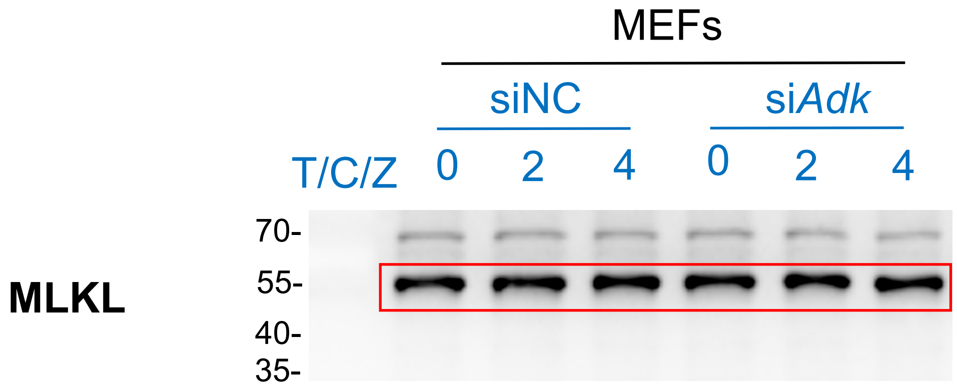

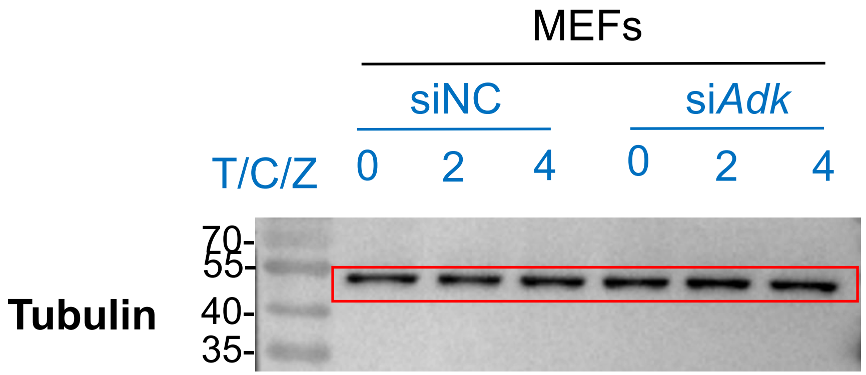

Supplement: SourceData FS1 — is the source file for Fig. S1. [file jem_20250603_sourcedatafs1.pdf]
