# Supplementary material for: Adenosine metabolic clearance maintains liver homeostasis by licensing arginine methylation of RIPK1
Source: J Exp Med. 2025 Oct 13;223(1):e20250603. doi: 10.1084/jem.20250603 (PMC12517274; doi:10.1084/jem.20250603)

Uncropped blots_Supplemental Figure 2

Panel F

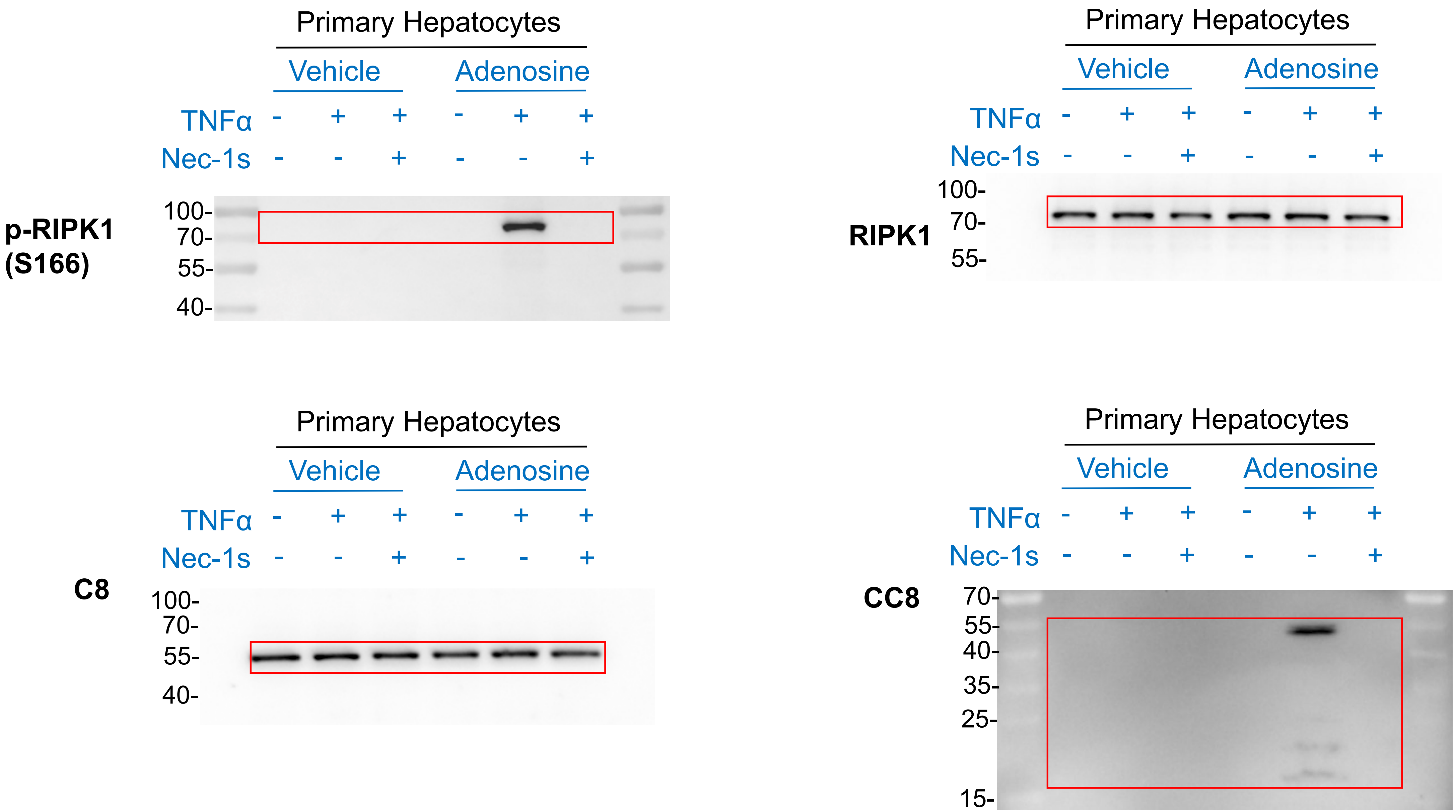

Uncropped blots_Supplemental Figure 2

Panel F

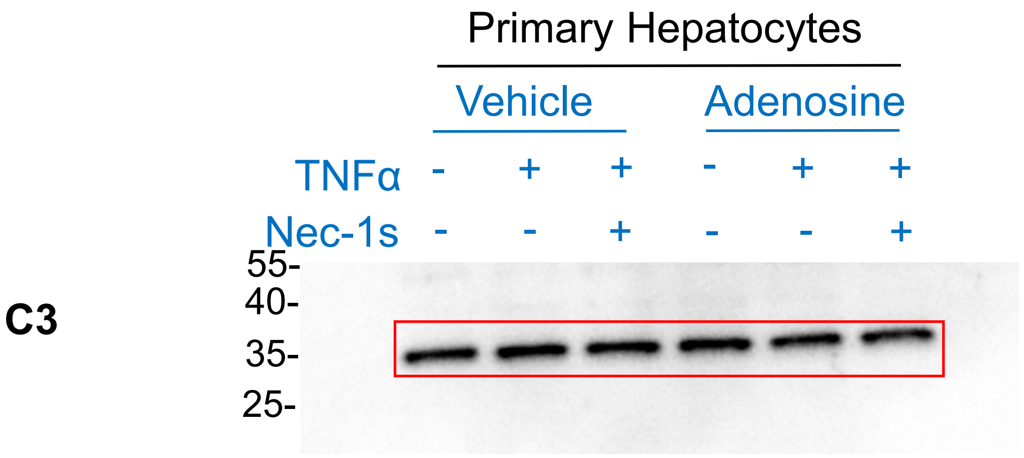

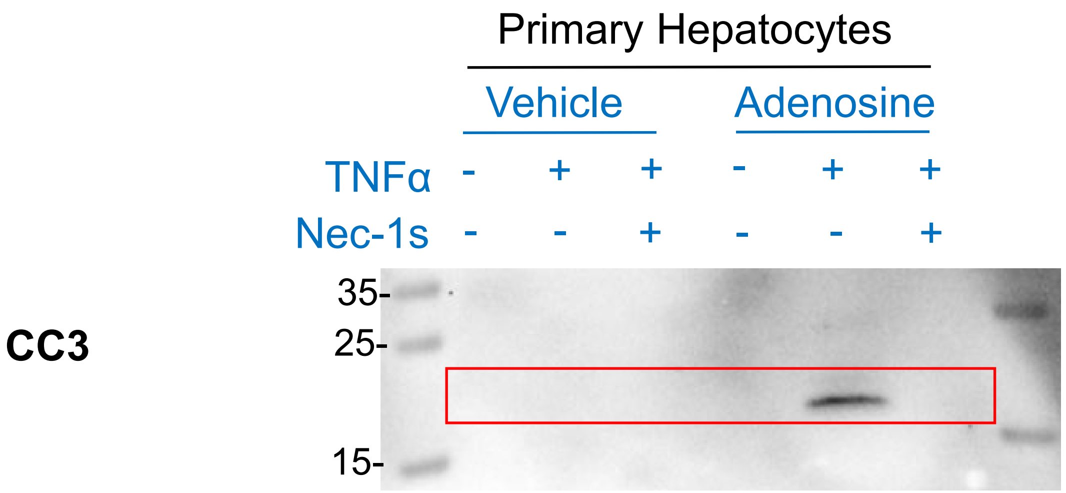

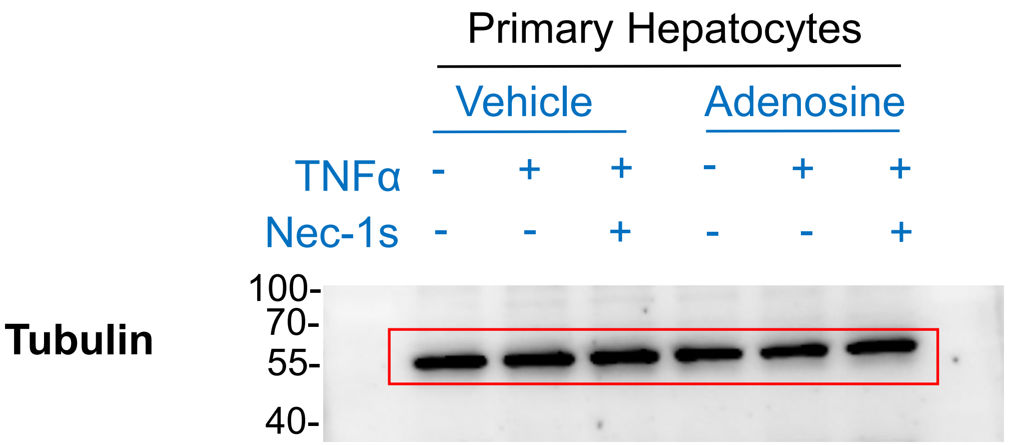

Uncropped blots_Supplemental Figure 2

Panel H

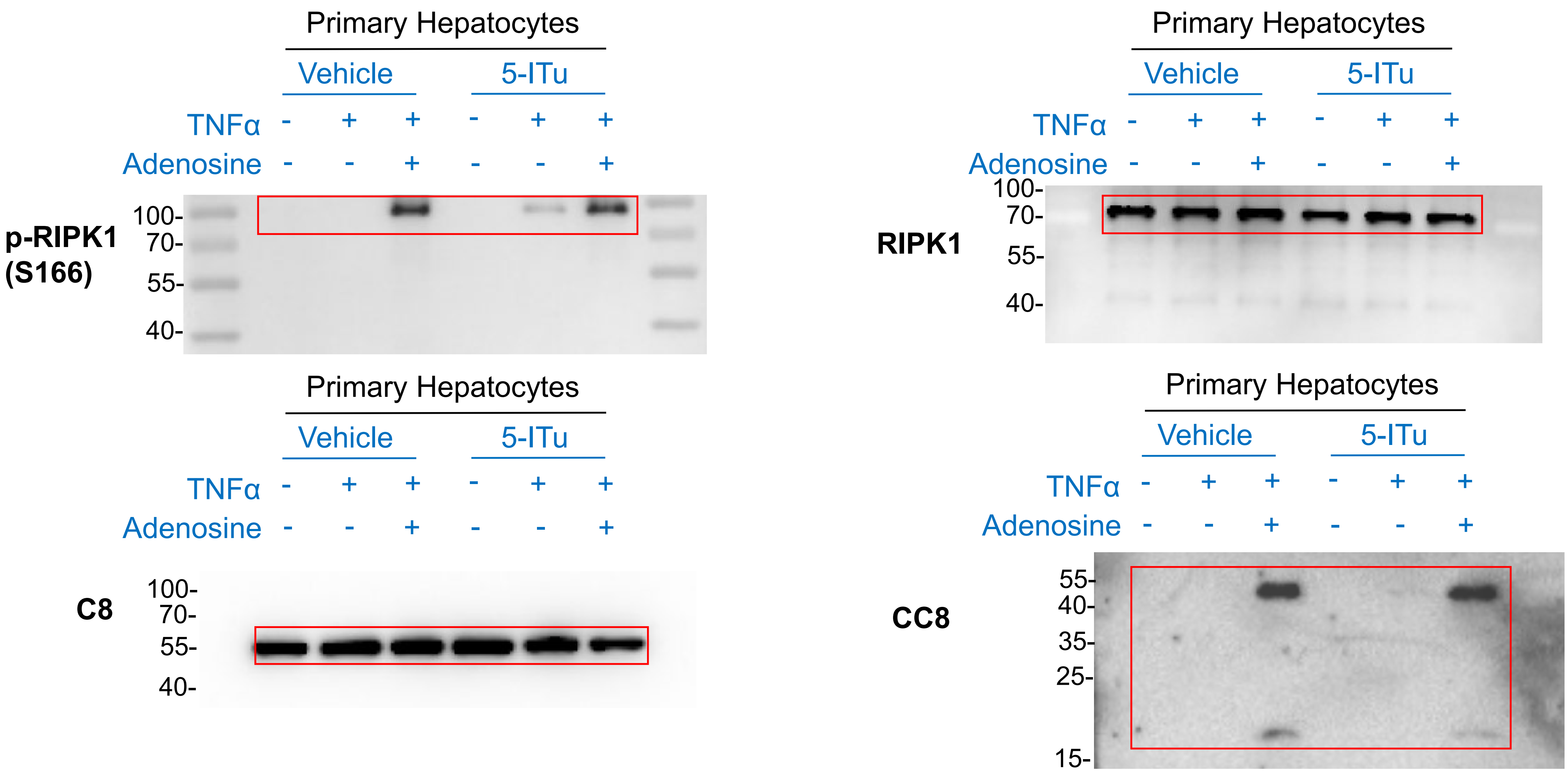

Uncropped blots_Supplemental Figure 2

Panel H

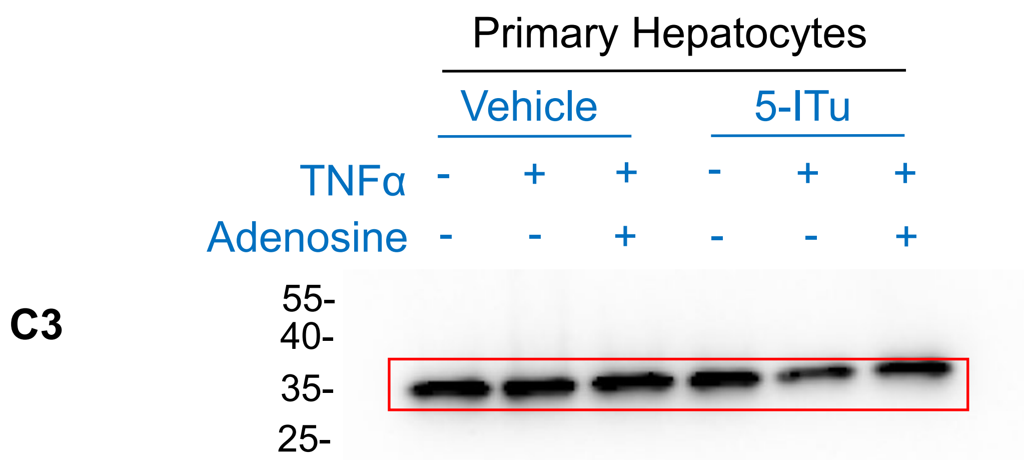

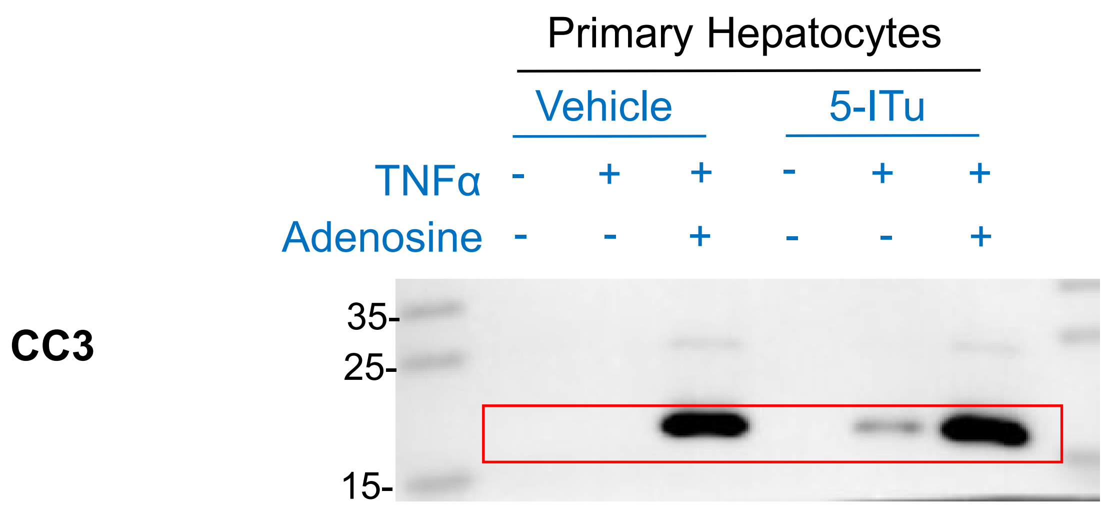

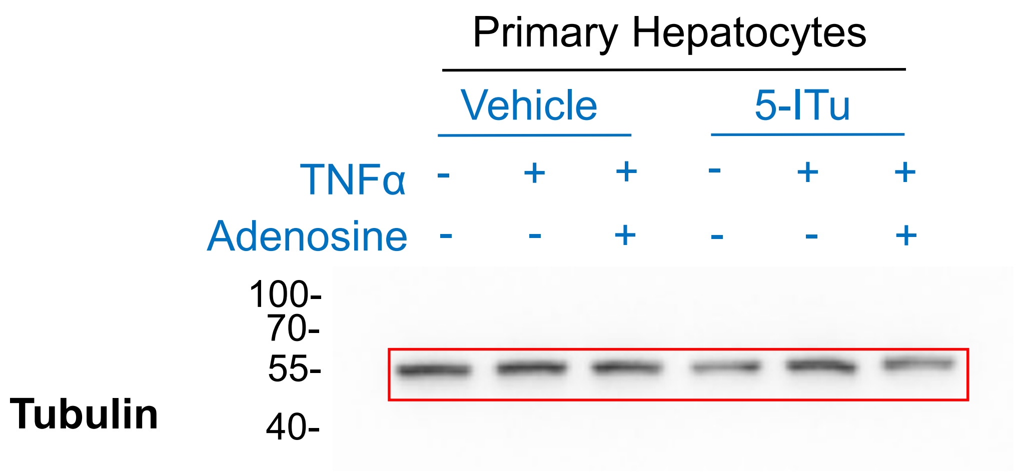

Uncropped blots_Supplemental Figure 2

Panel J

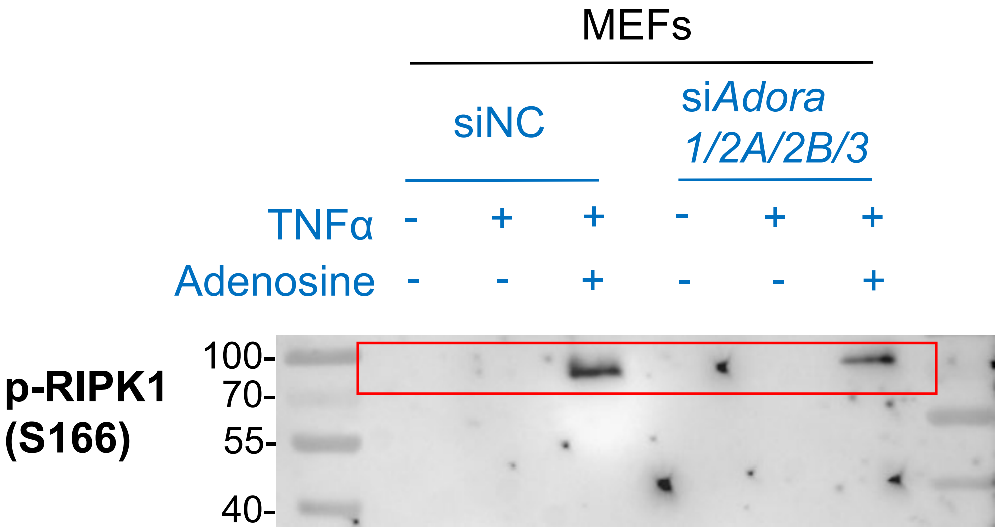

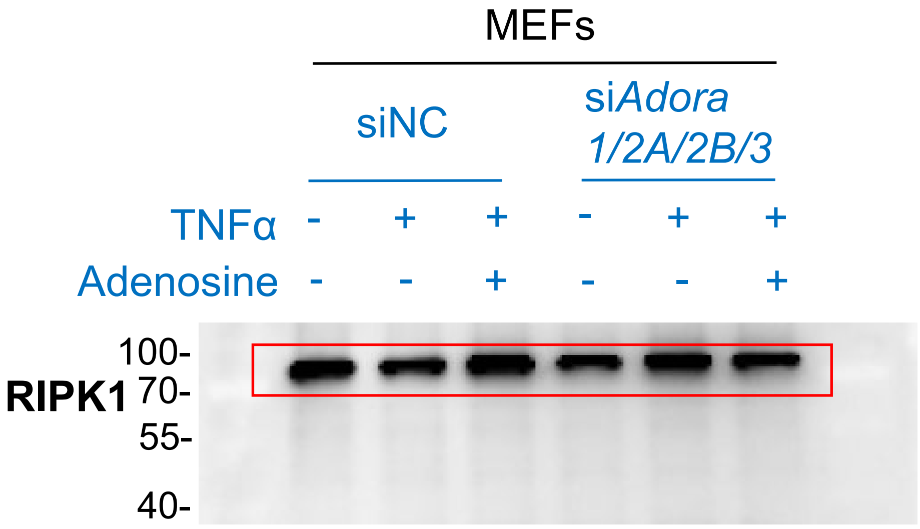

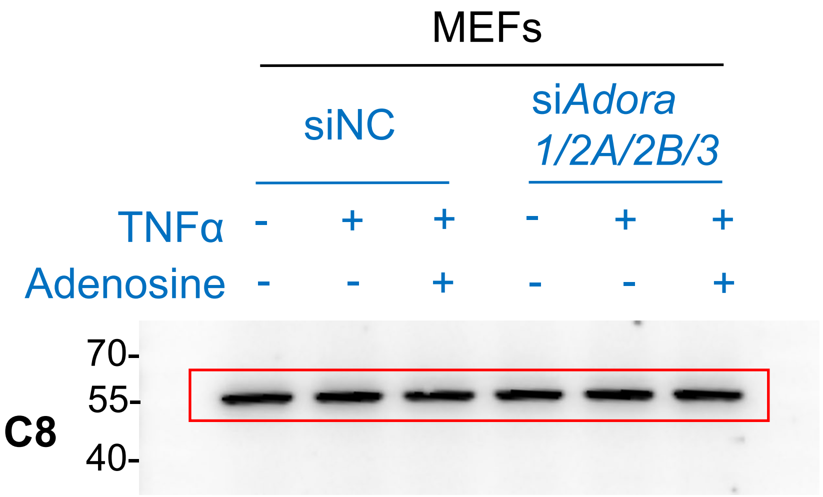

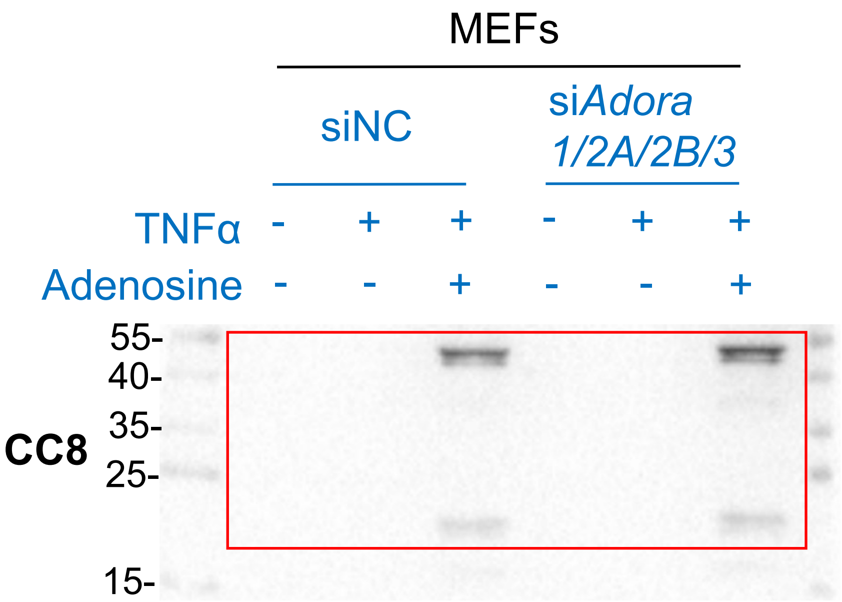

Uncropped blots_Supplemental Figure 2

Panel J

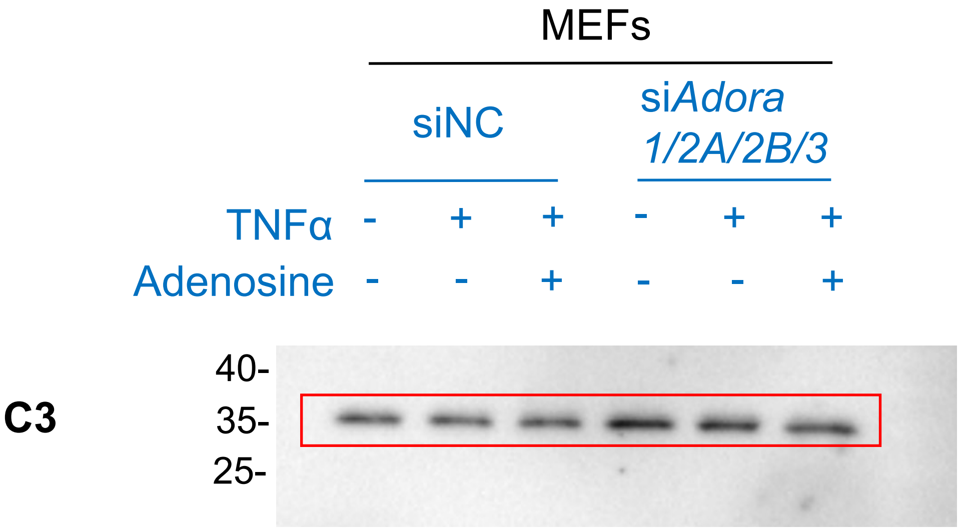

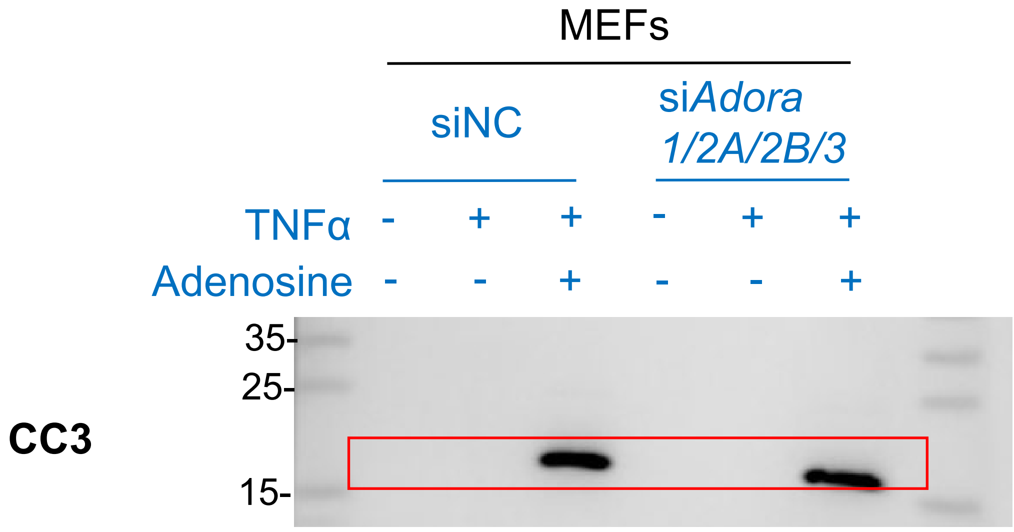

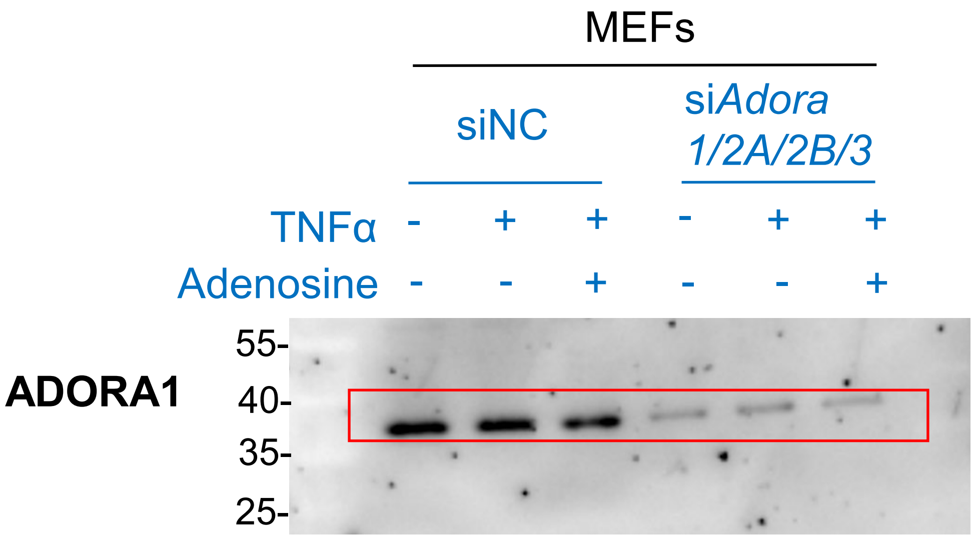

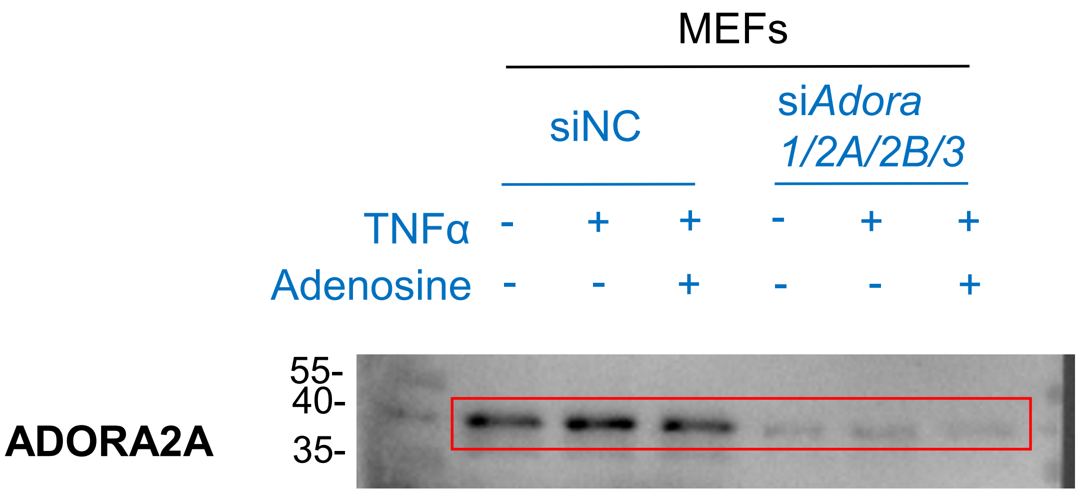

Uncropped blots_Supplemental Figure 2

Panel J

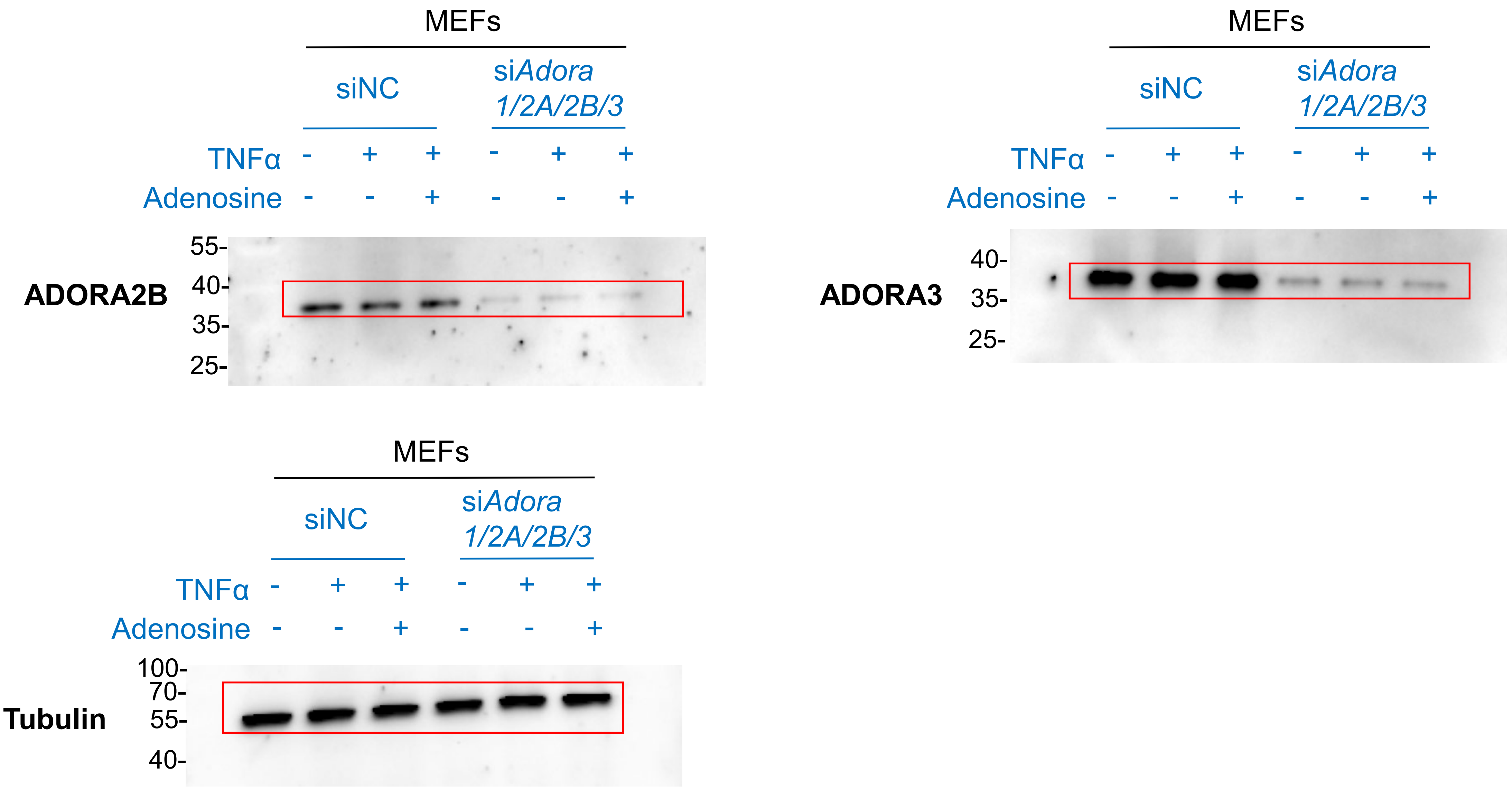

Uncropped blots_Supplemental Figure 2

Panel O

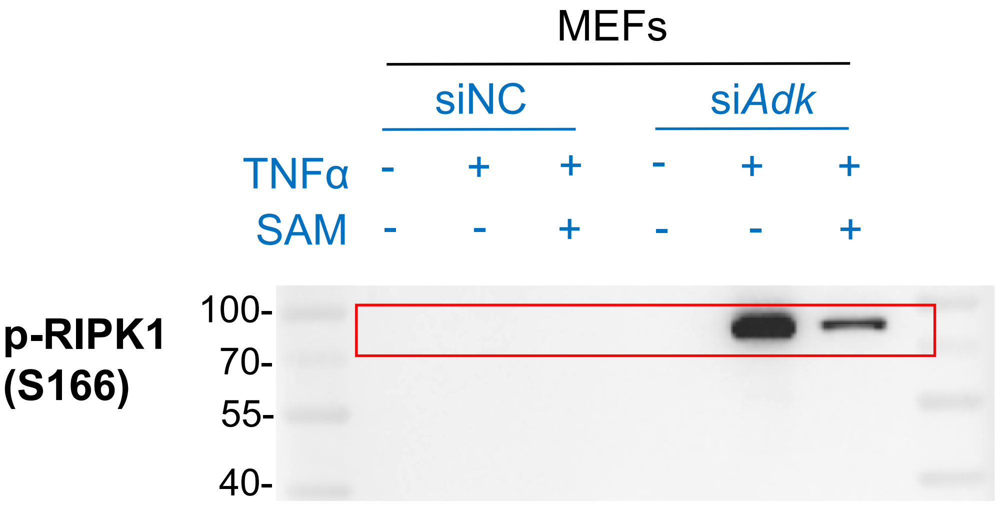

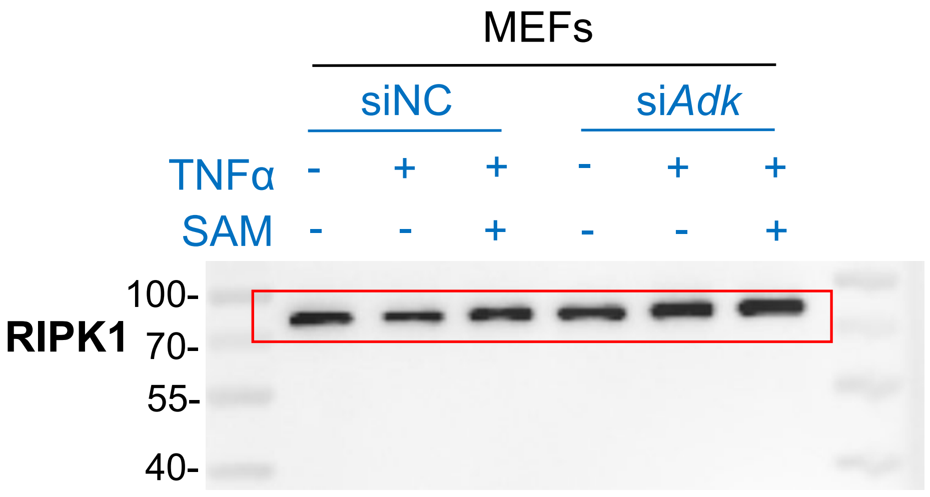

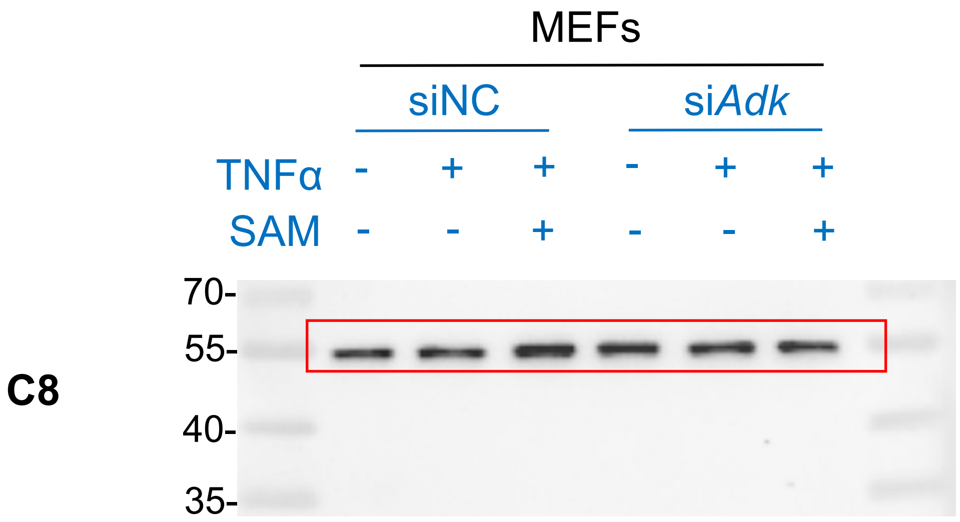

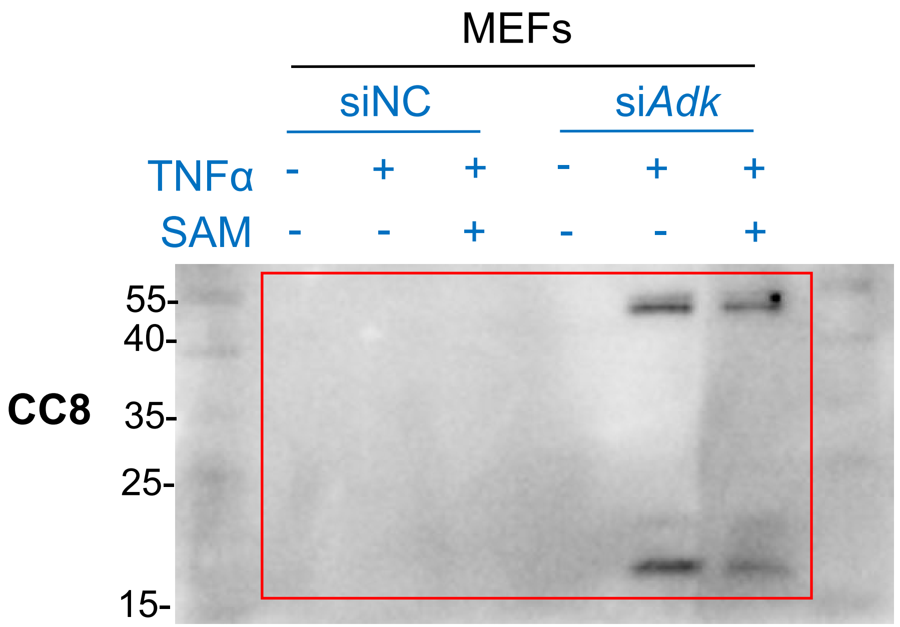

Uncropped blots_Supplemental Figure 2

Panel O

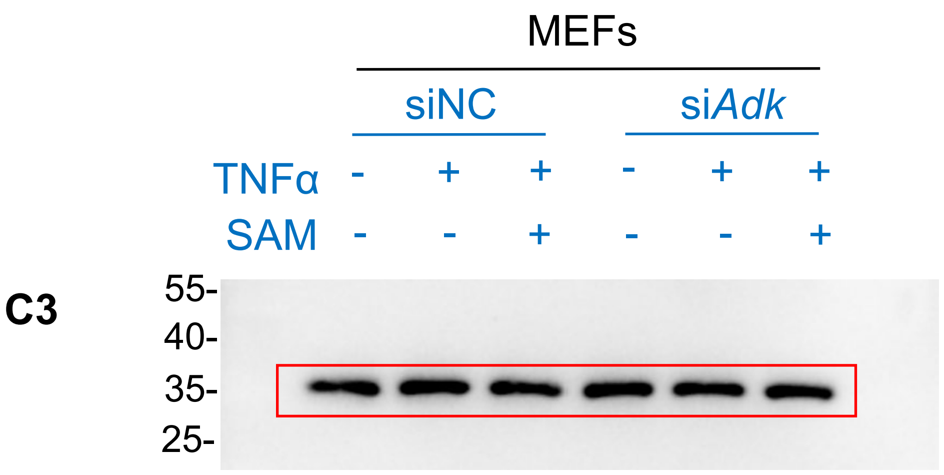

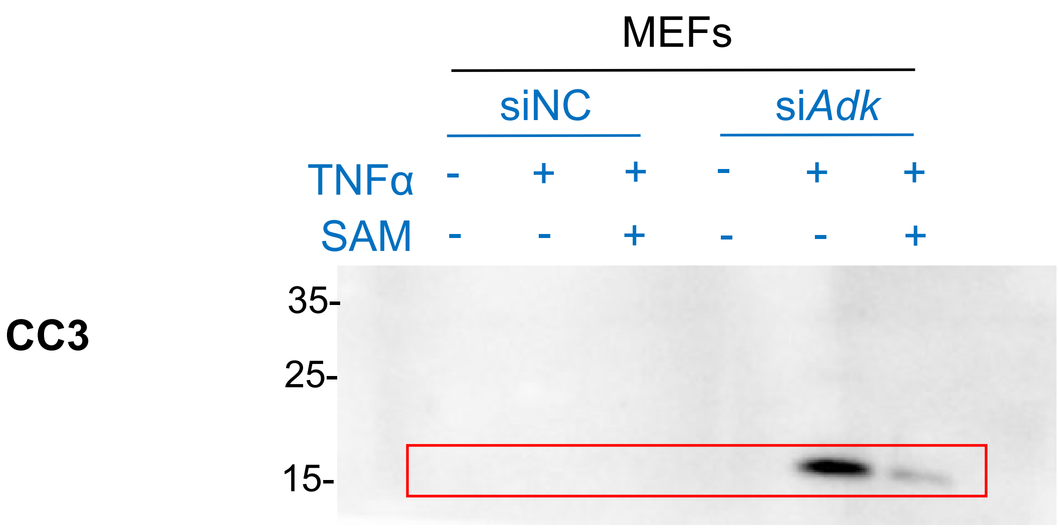

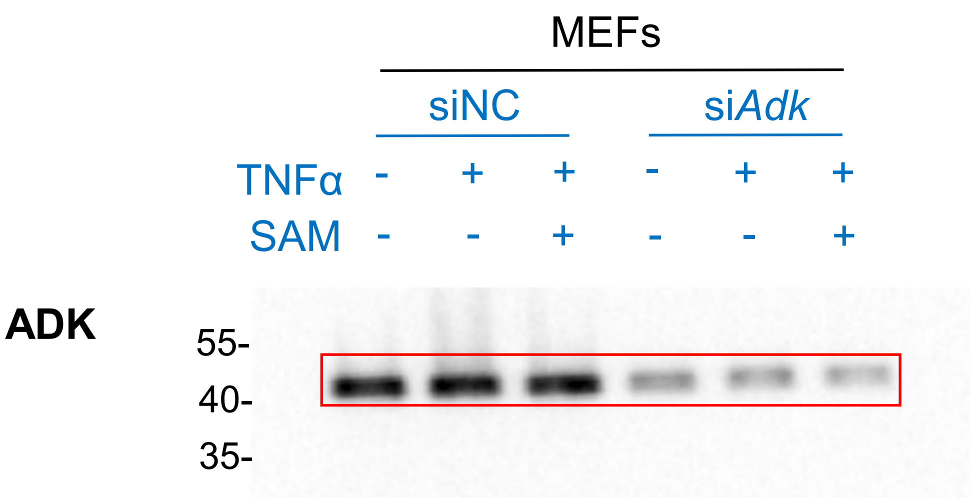

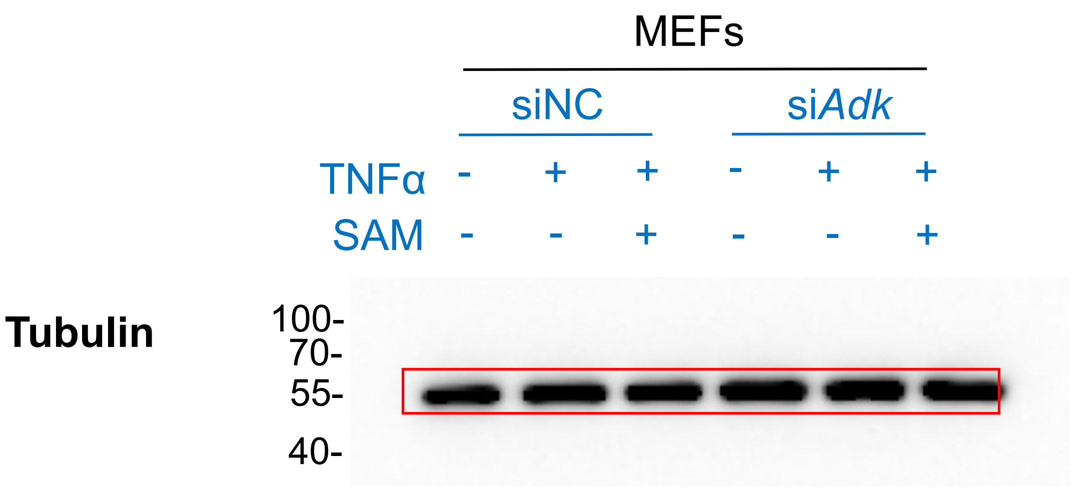

Uncropped blots_Supplemental Figure 2

Panel Q

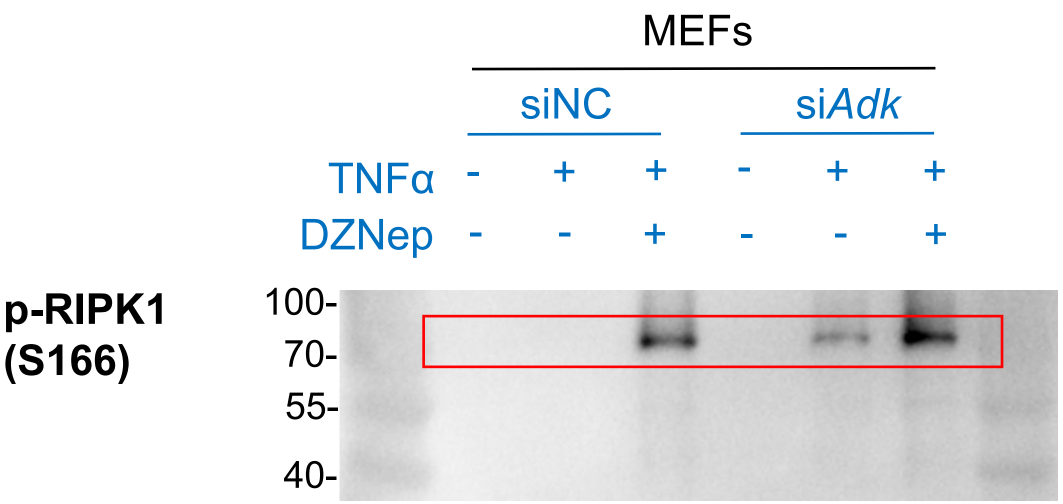

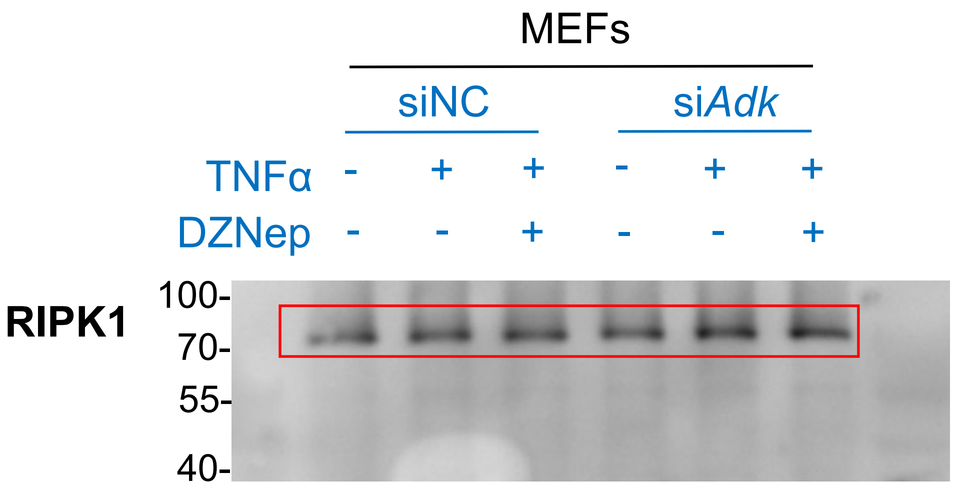

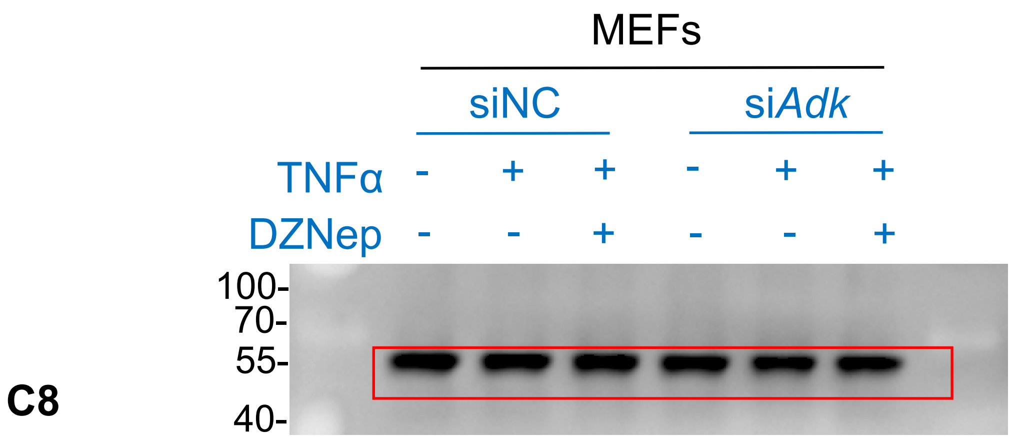

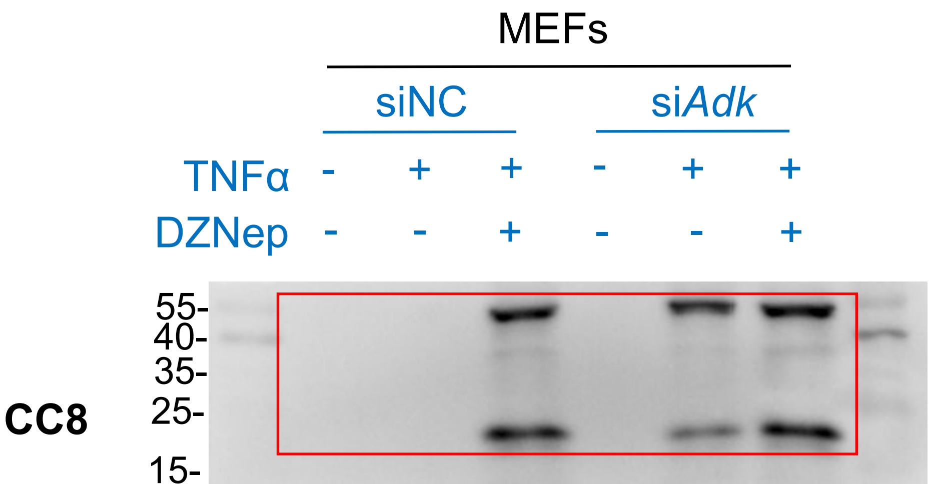

Uncropped blots_Supplemental Figure 2

Panel Q

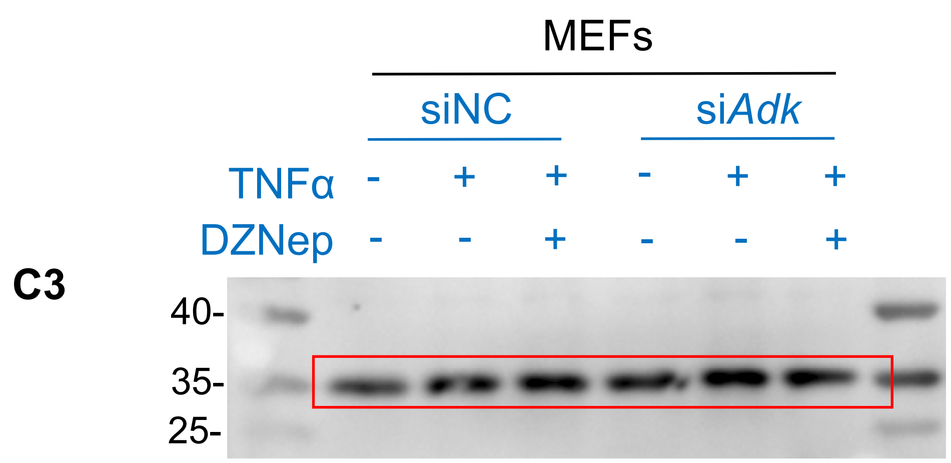

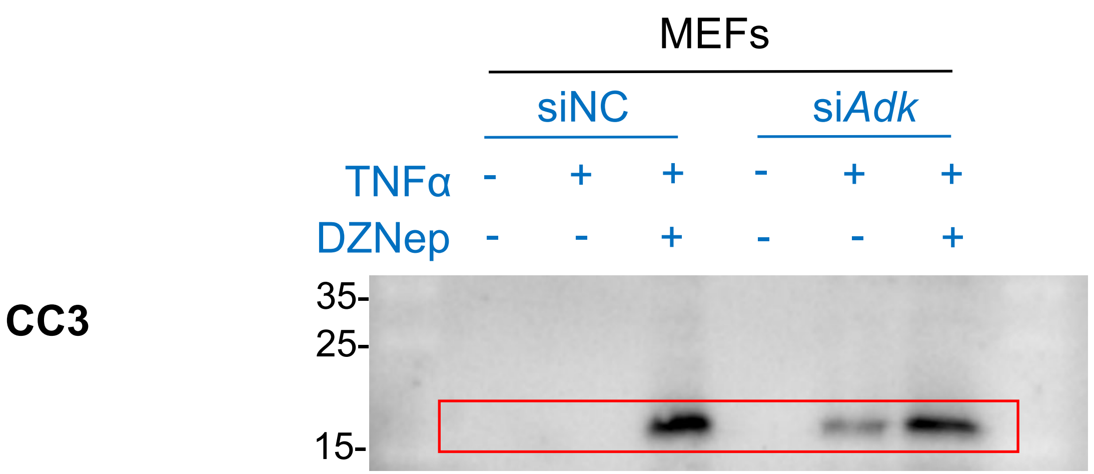

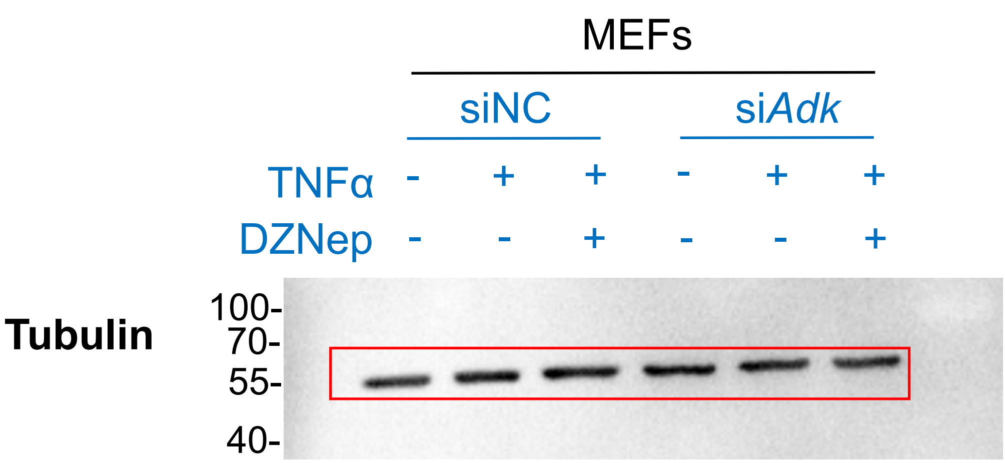

Supplement: SourceData FS2 — is the source file for Fig. S2. [file jem_20250603_sourcedatafs2.pdf]
